# Supplementary figures and images for: Human fetal kidney organoids model early human nephrogenesis and Notch-driven cell fate (part 1 of 2)
Source: EMBO J. 2025 Jul 21;44(17):4681–719. doi: 10.1038/s44318-025-00504-2 (PMC12402132; doi:10.1038/s44318-025-00504-2)

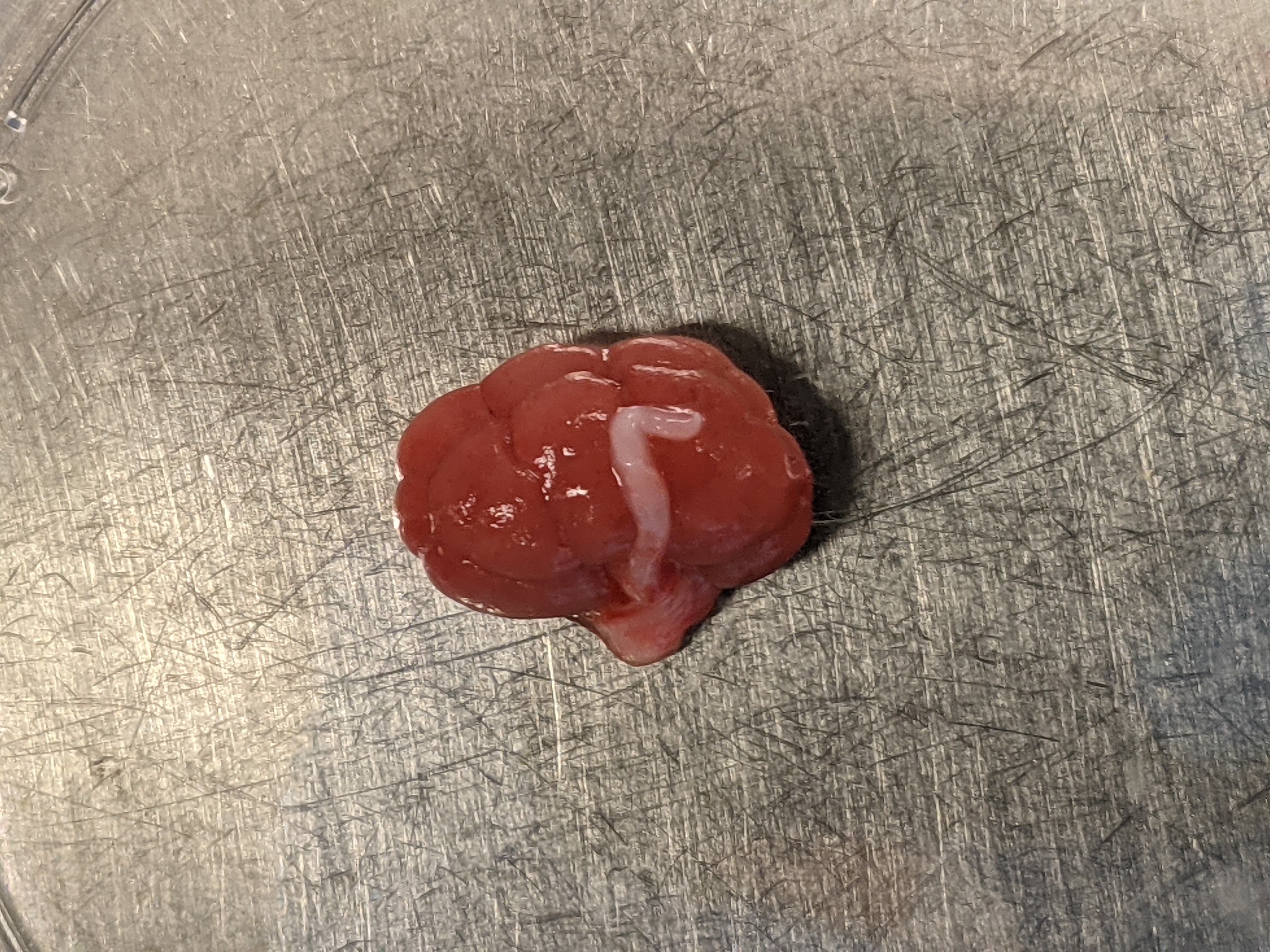

Supplement: Supplementary file 3 — Source data Fig. 1 [file 44318_2025_504_MOESM3_ESM.zip › Figure 1/1A/Human Fetal kidney.jpg]

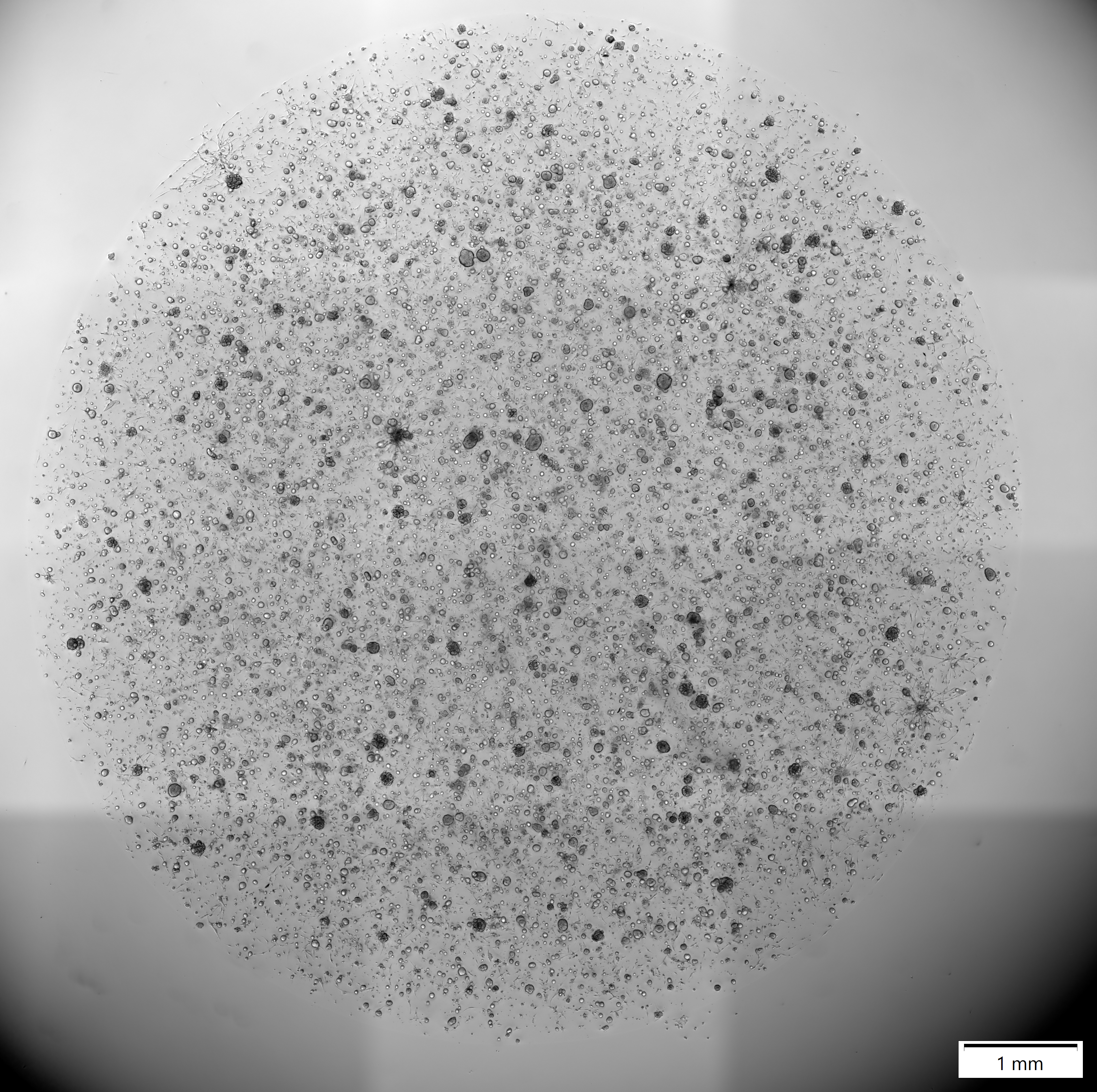

Supplement: Supplementary file 3 — Source data Fig. 1 [file 44318_2025_504_MOESM3_ESM.zip › Figure 1/1A/widefield-P0-hFKOs after seeding.tif]

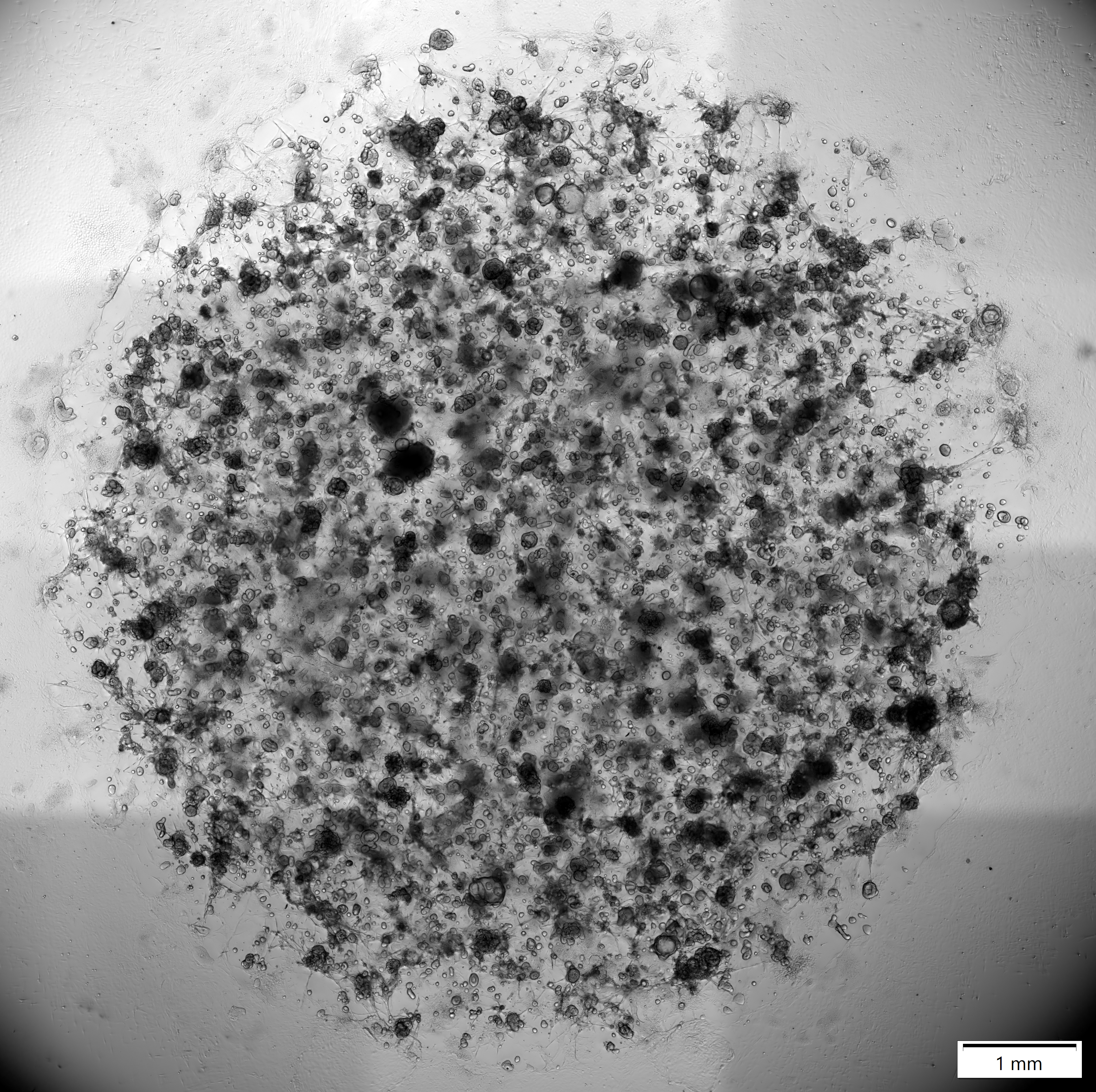

Supplement: Supplementary file 3 — Source data Fig. 1 [file 44318_2025_504_MOESM3_ESM.zip › Figure 1/1A/widefield-P0-hFKOs-after-2w-culture.tif]

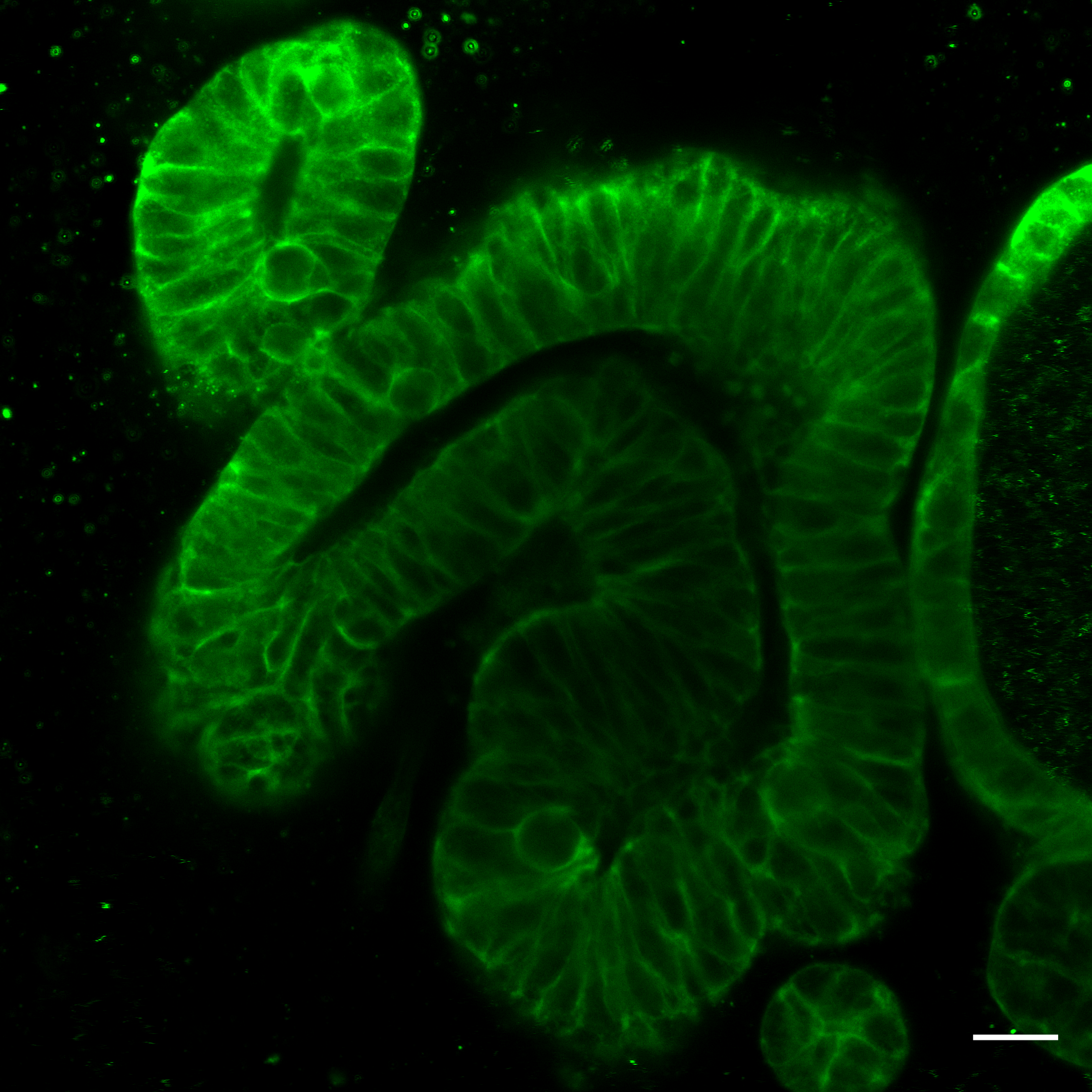

Supplement: Supplementary file 3 — Source data Fig. 1 [file 44318_2025_504_MOESM3_ESM.zip › Figure 1/1B/hFKO-EPCAM-green.tif]

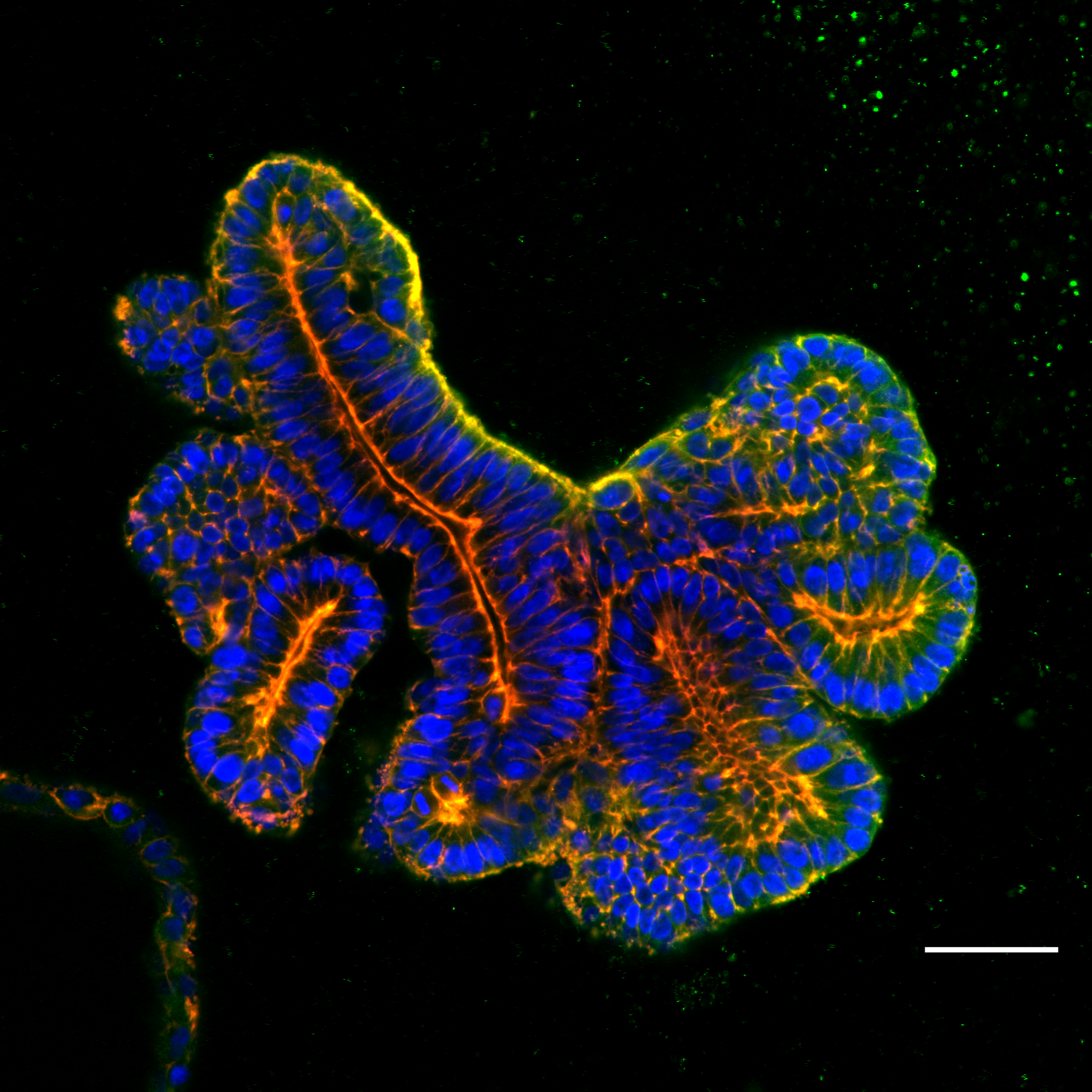

Supplement: Supplementary file 3 — Source data Fig. 1 [file 44318_2025_504_MOESM3_ESM.zip › Figure 1/1B/hFKO-F-ACTIN-red-EPCAM-green-DAPI-blue.tif]

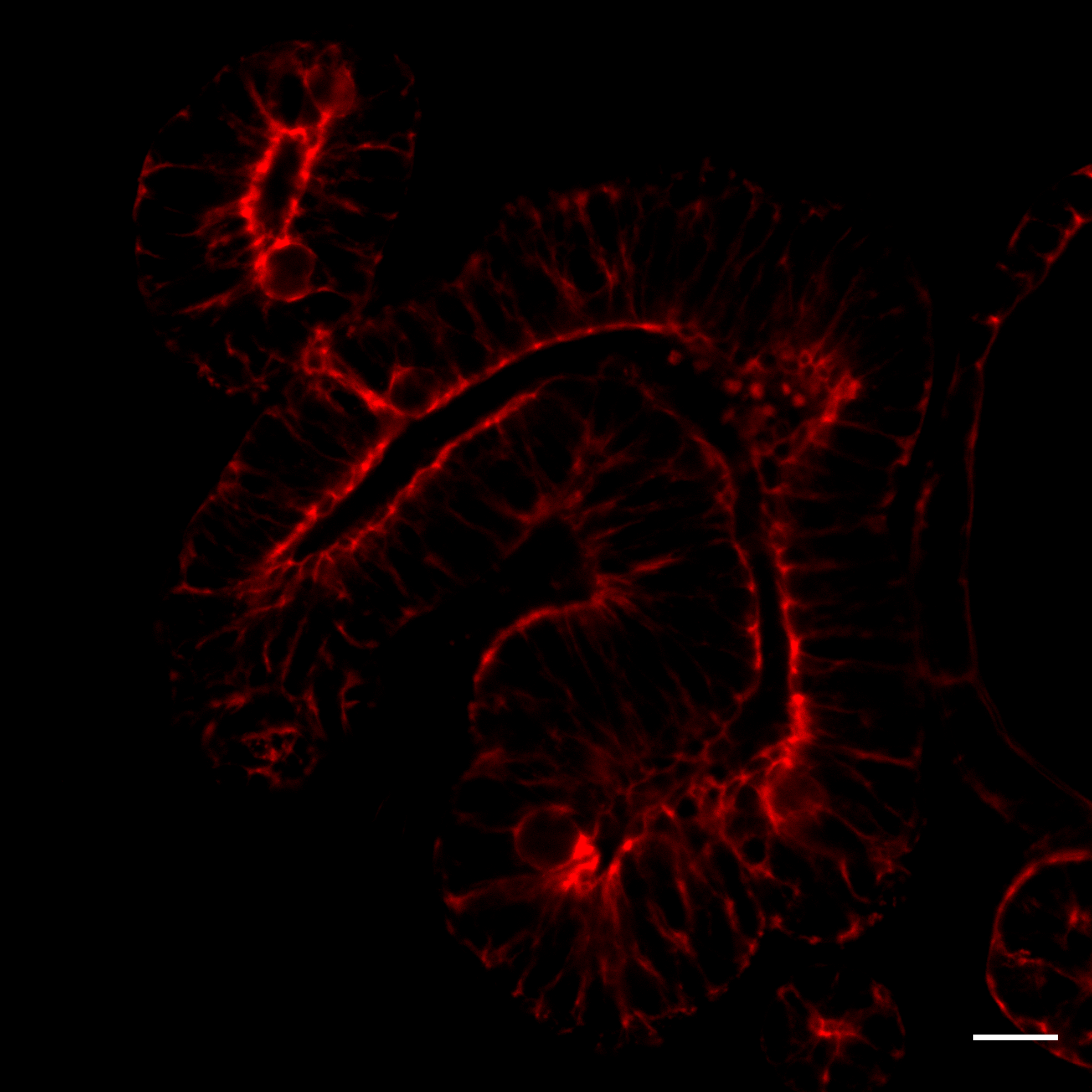

Supplement: Supplementary file 3 — Source data Fig. 1 [file 44318_2025_504_MOESM3_ESM.zip › Figure 1/1B/hFKO-F-ACTIN-red.tif]

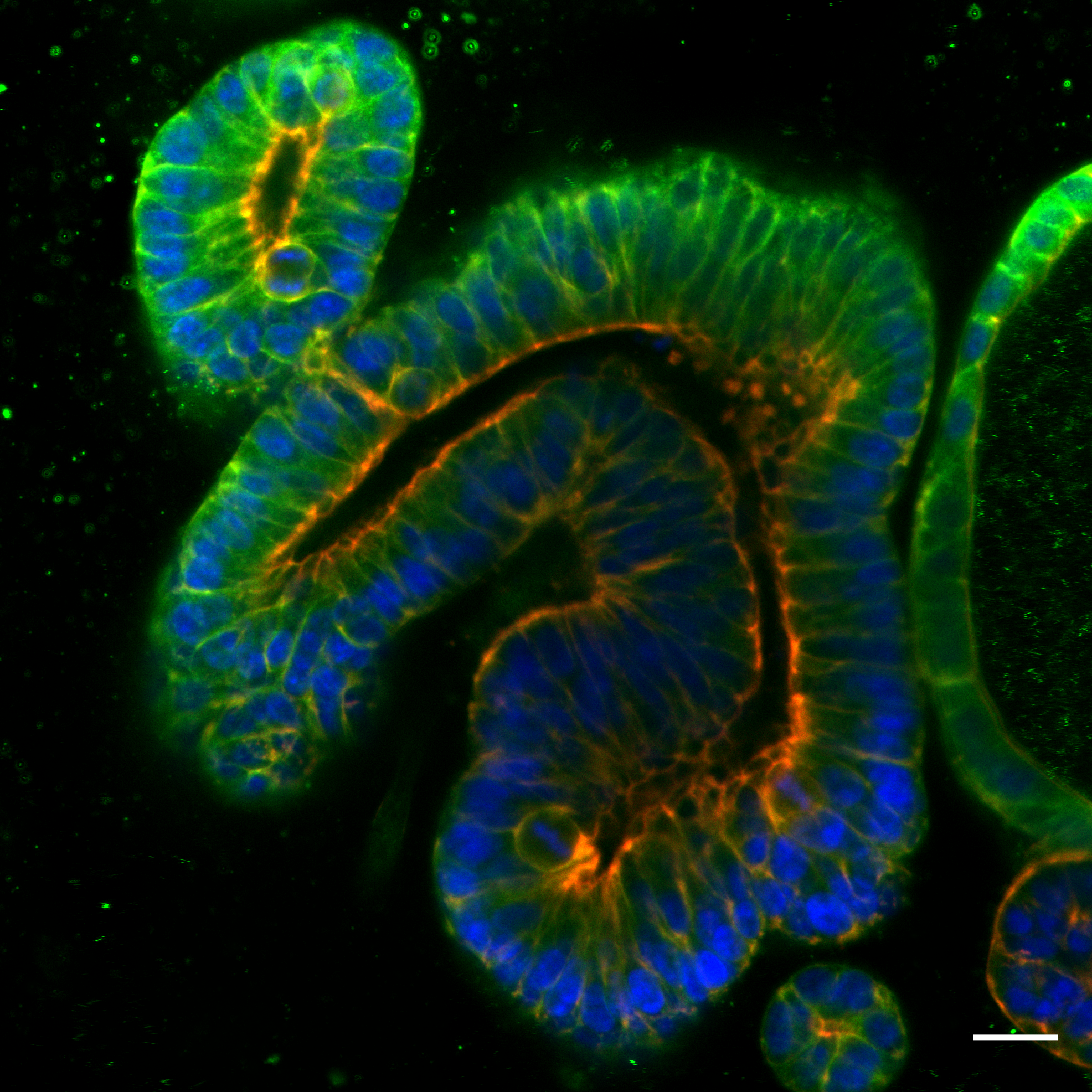

Supplement: Supplementary file 3 — Source data Fig. 1 [file 44318_2025_504_MOESM3_ESM.zip › Figure 1/1B/hFKO-merge.tif]

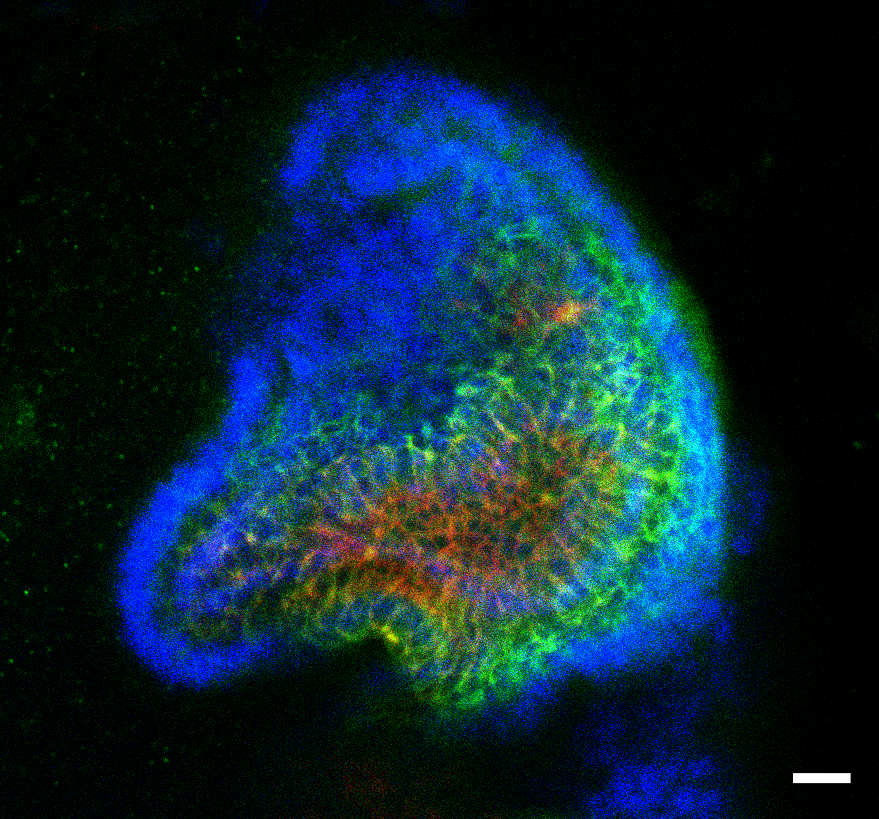

Supplement: Supplementary file 3 — Source data Fig. 1 [file 44318_2025_504_MOESM3_ESM.zip › Figure 1/1C/zstack-1-hFKO-F-ACTIN-red-EPCAM-green-DAPI-blue.tif]

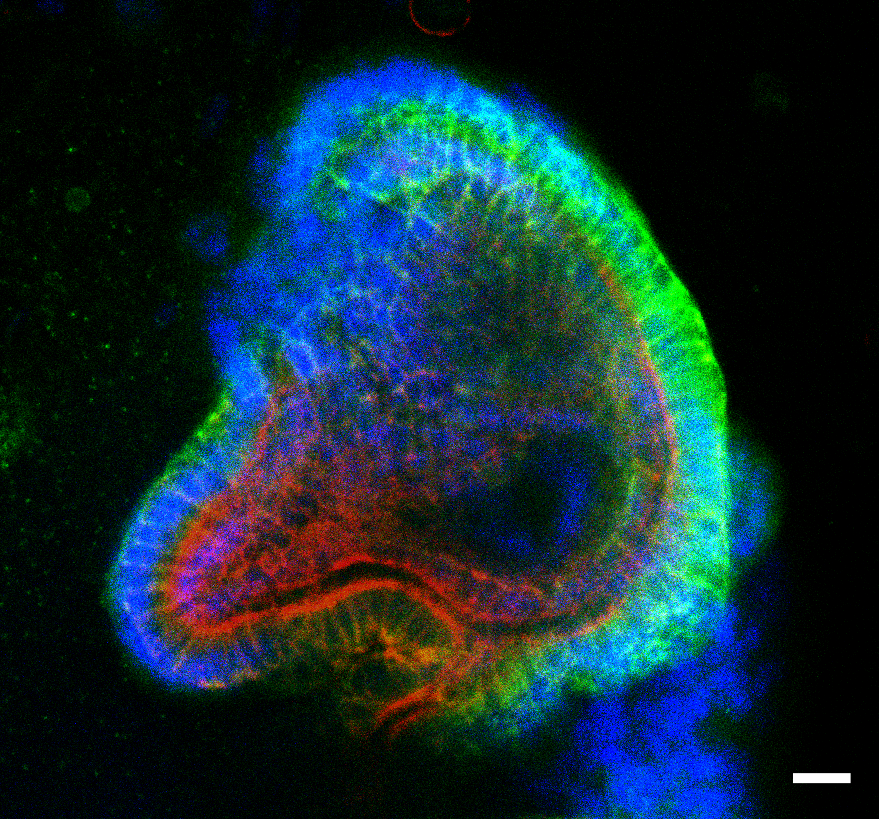

Supplement: Supplementary file 3 — Source data Fig. 1 [file 44318_2025_504_MOESM3_ESM.zip › Figure 1/1C/zstack-2-hFKO-F-ACTIN-red-EPCAM-green-DAPI-blue.tif.tif]

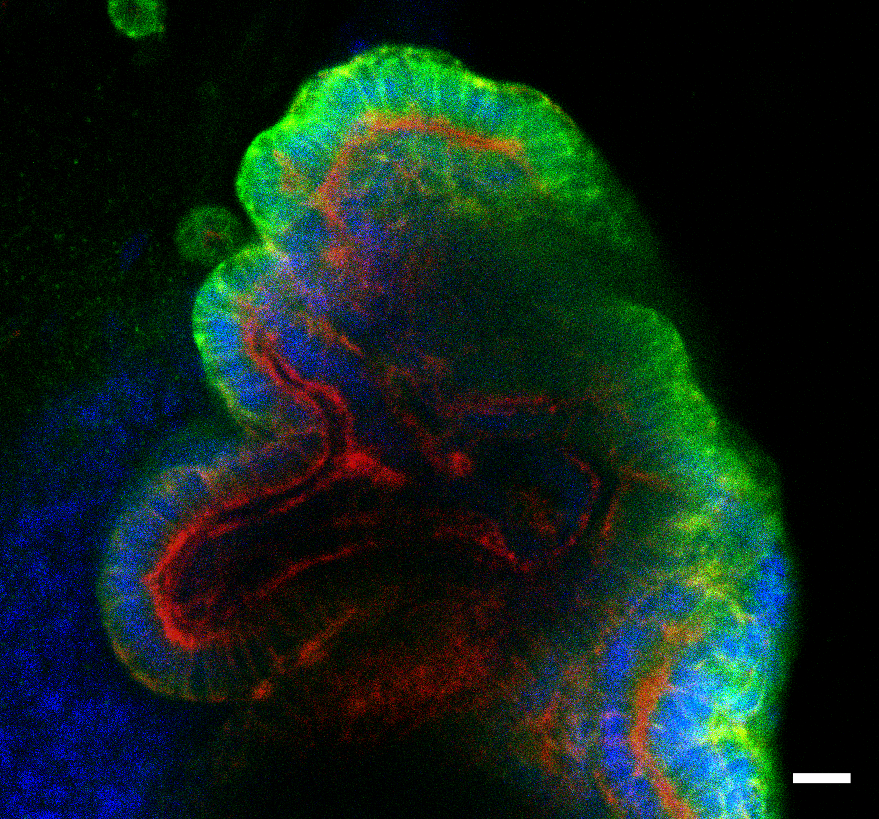

Supplement: Supplementary file 3 — Source data Fig. 1 [file 44318_2025_504_MOESM3_ESM.zip › Figure 1/1C/zstack-3-hFKO-F-ACTIN-red-EPCAM-green-DAPI-blue.tif.tif]

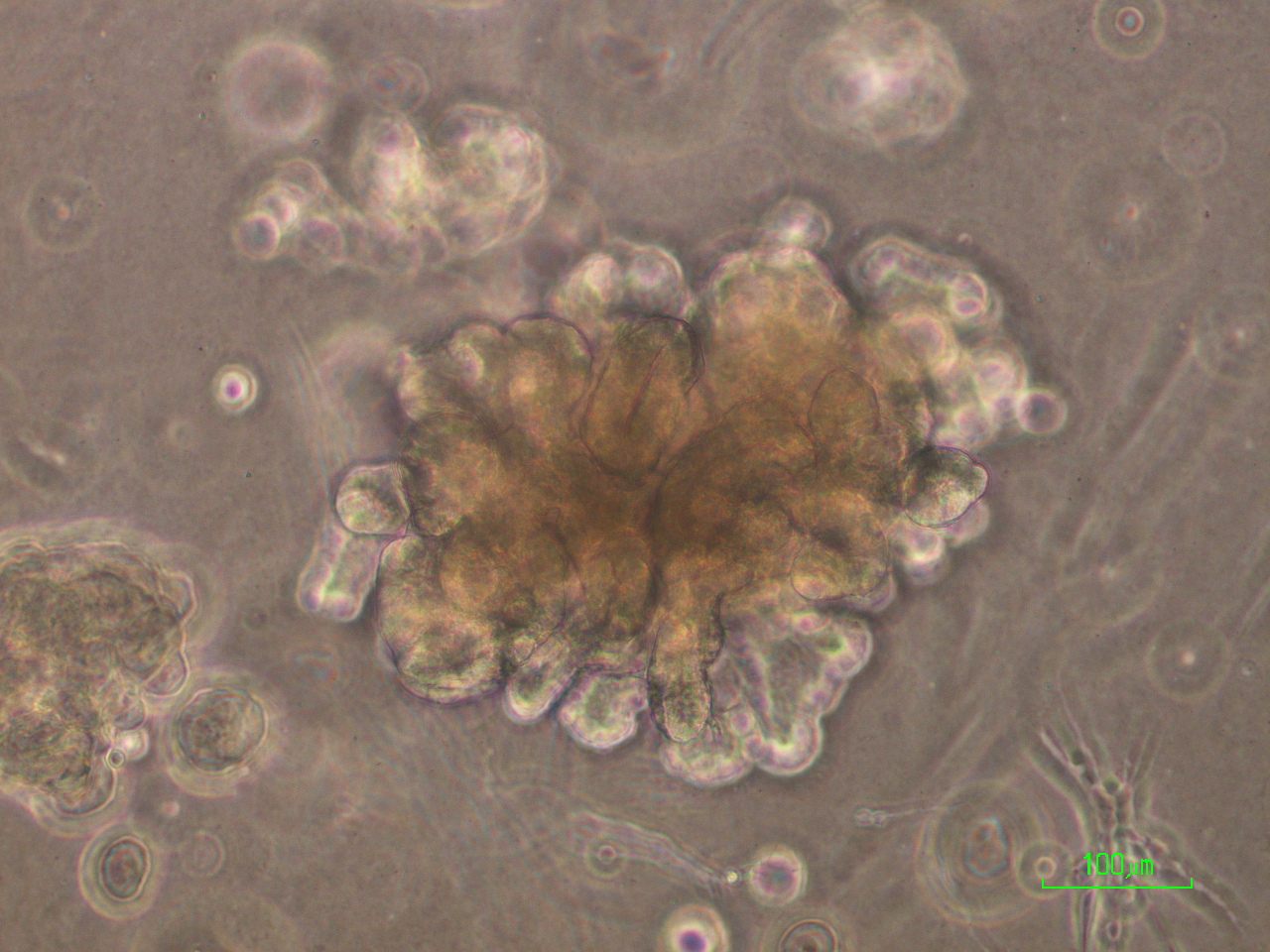

Supplement: Supplementary file 3 — Source data Fig. 1 [file 44318_2025_504_MOESM3_ESM.zip › Figure 1/1D/hFKO-P0.TIF]

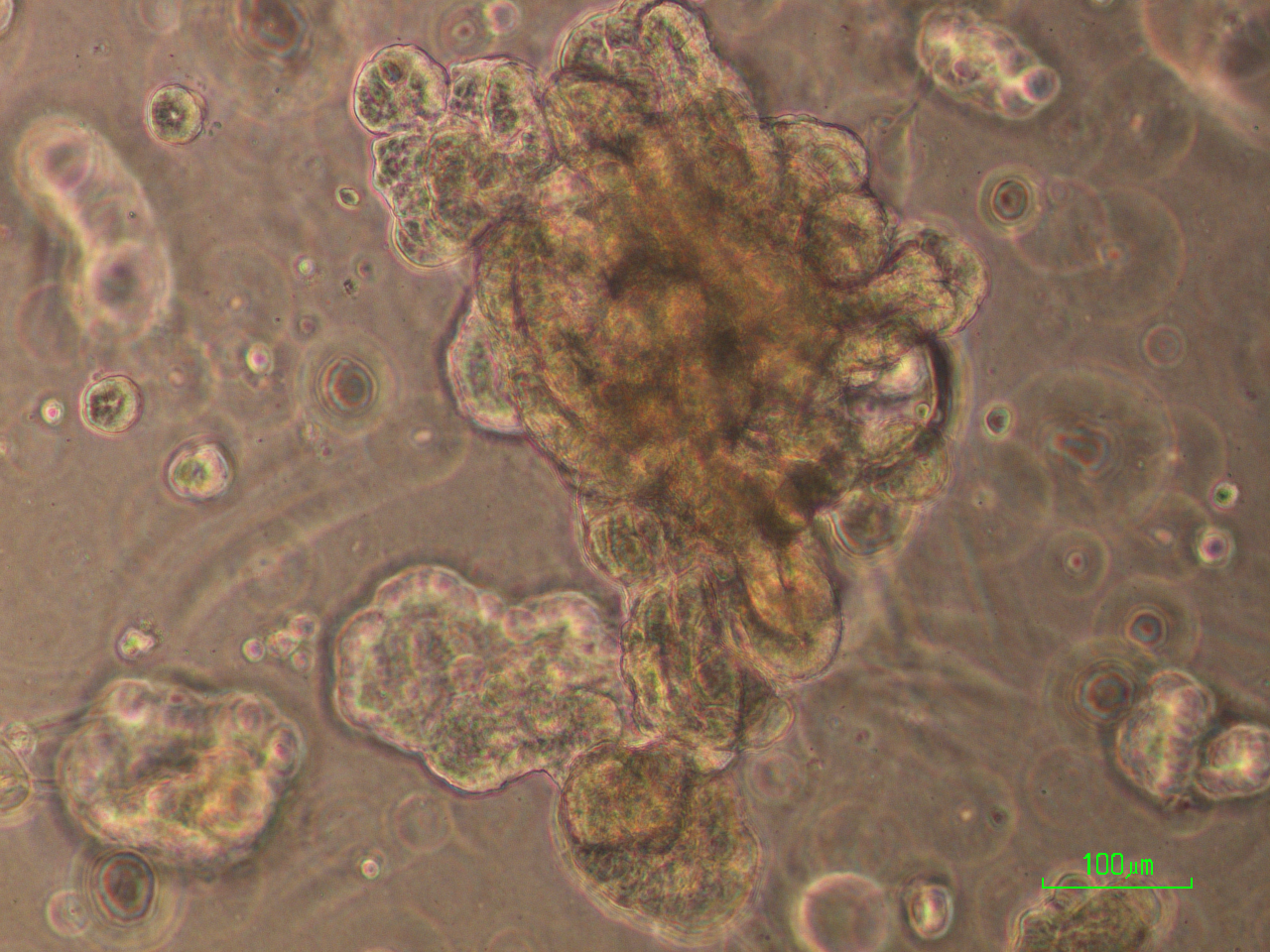

Supplement: Supplementary file 3 — Source data Fig. 1 [file 44318_2025_504_MOESM3_ESM.zip › Figure 1/1D/hFKO-P2.TIF]

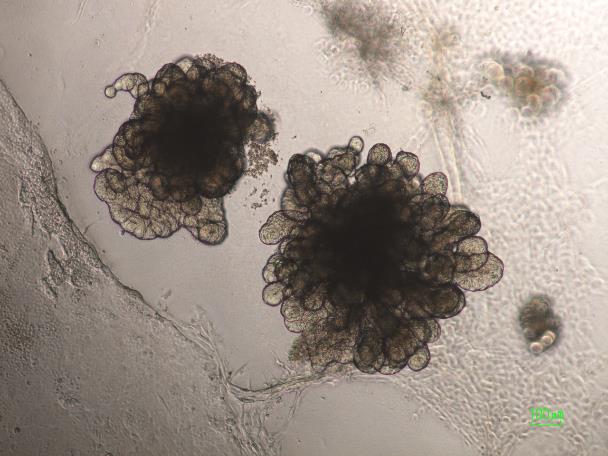

Supplement: Supplementary file 3 — Source data Fig. 1 [file 44318_2025_504_MOESM3_ESM.zip › Figure 1/1D/hFKO-P8.jpeg]

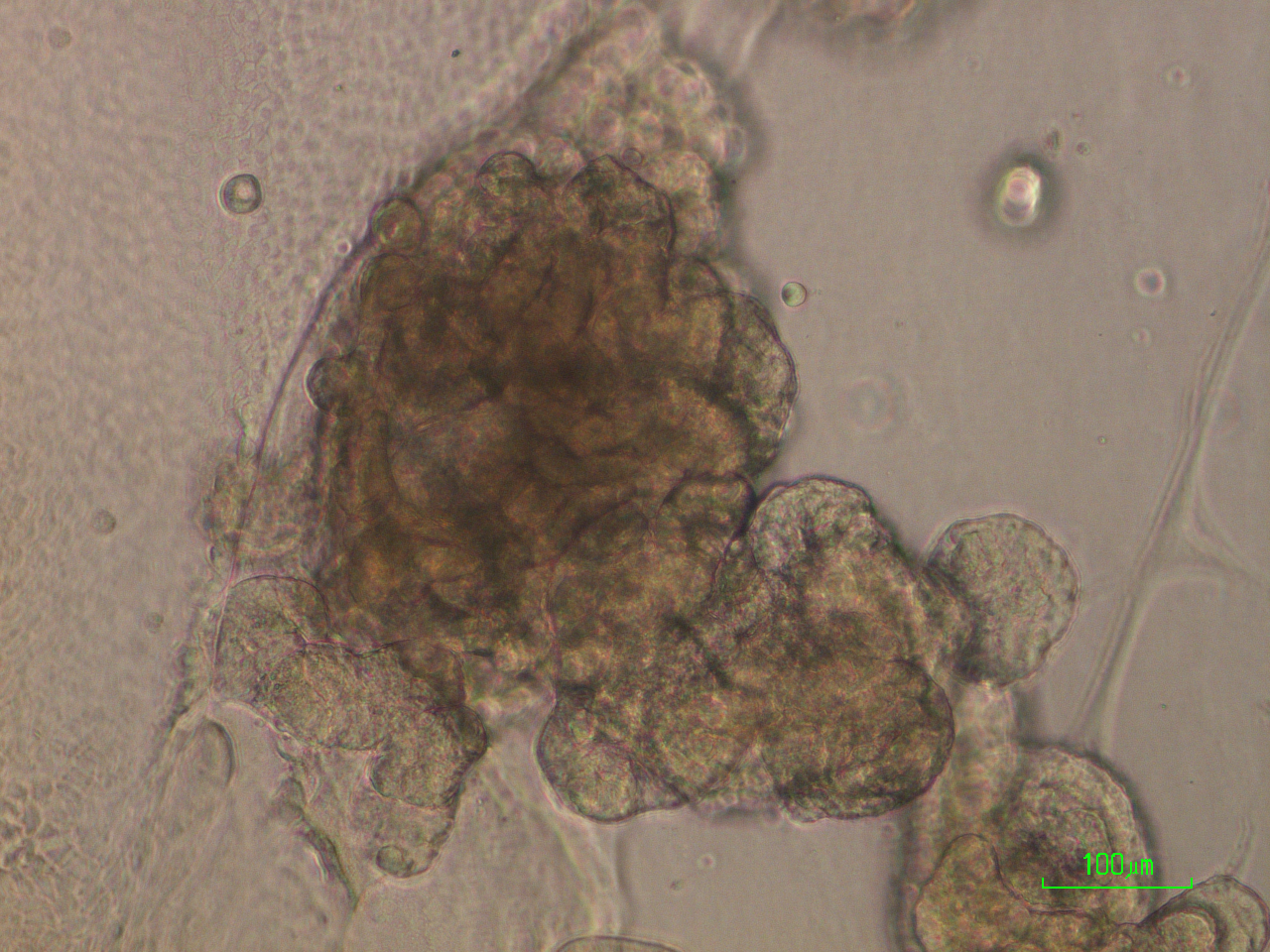

Supplement: Supplementary file 3 — Source data Fig. 1 [file 44318_2025_504_MOESM3_ESM.zip › Figure 1/1D/P0 replicates/hFKO-P0-1.TIF]

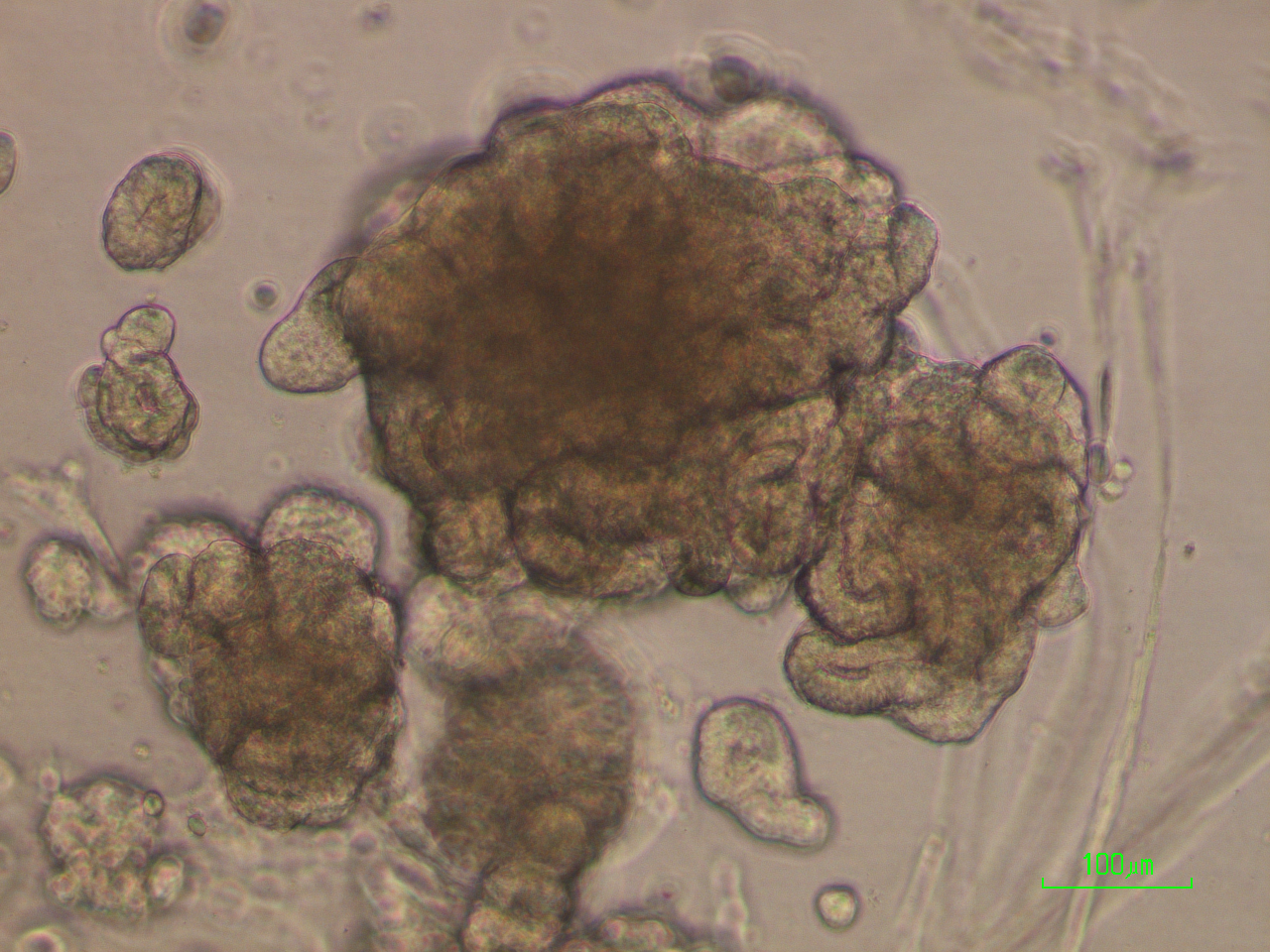

Supplement: Supplementary file 3 — Source data Fig. 1 [file 44318_2025_504_MOESM3_ESM.zip › Figure 1/1D/P0 replicates/hFKO-P0-2.TIF]

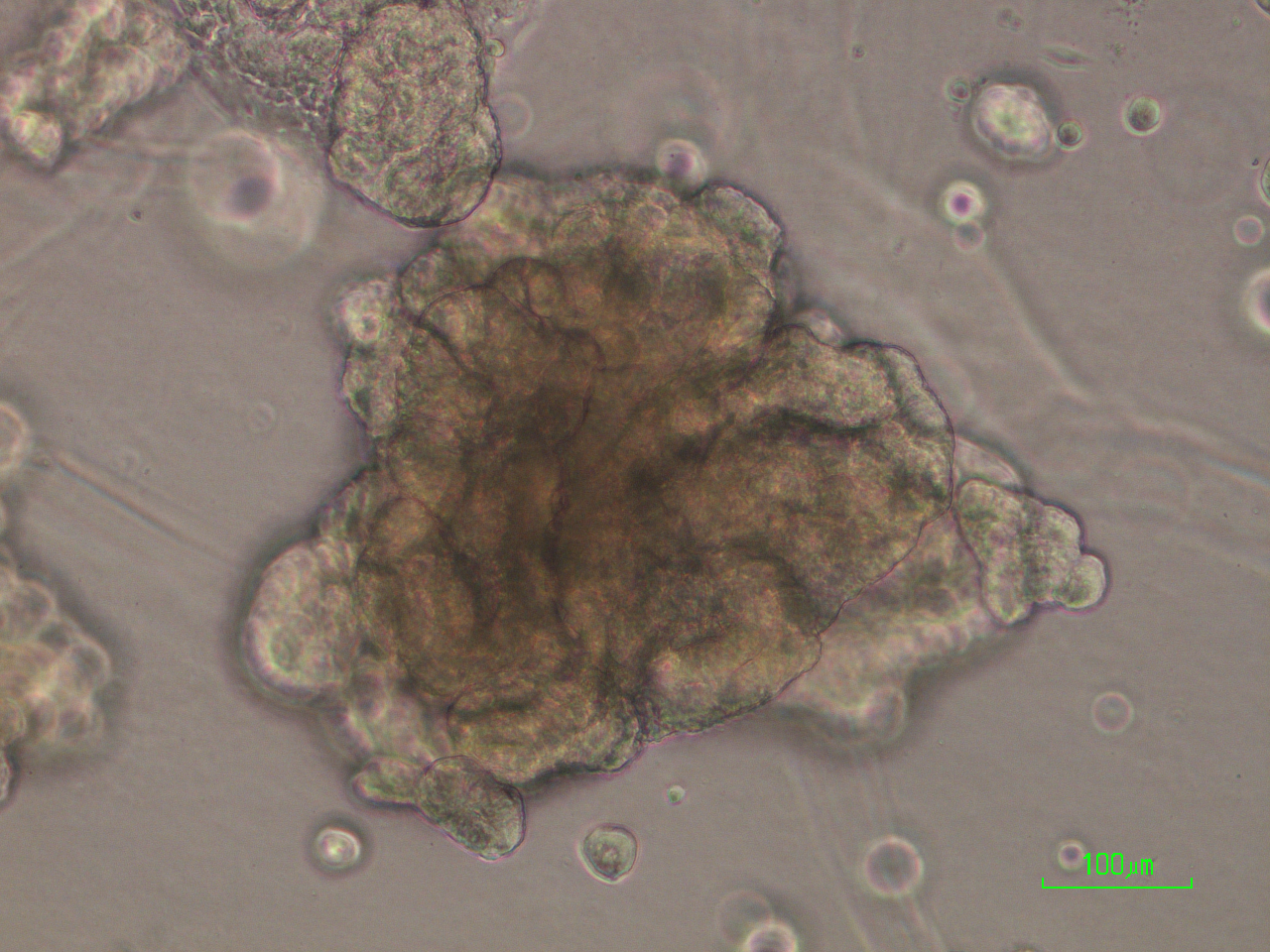

Supplement: Supplementary file 3 — Source data Fig. 1 [file 44318_2025_504_MOESM3_ESM.zip › Figure 1/1D/P0 replicates/hFKO-P0-3.TIF]

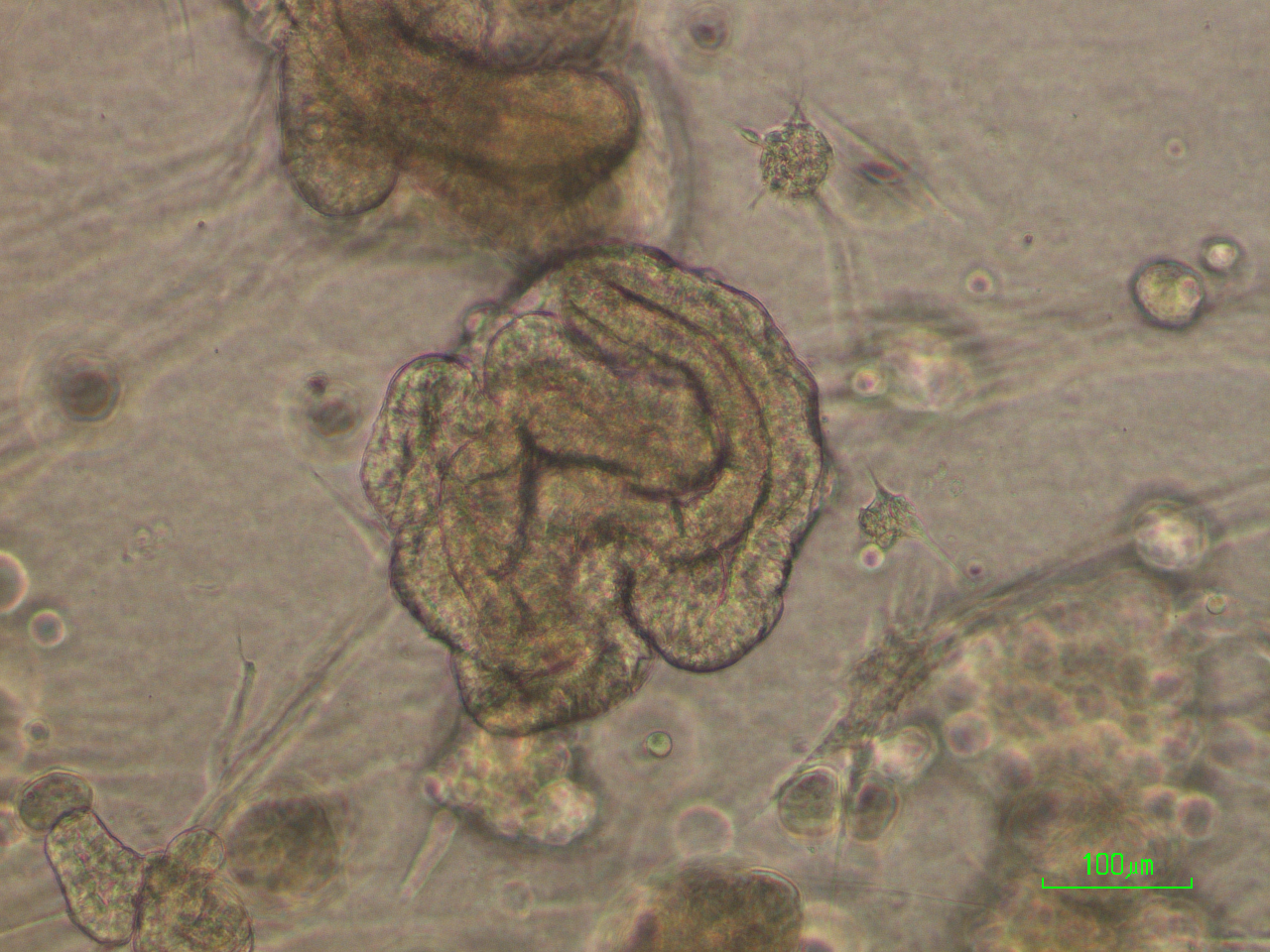

Supplement: Supplementary file 3 — Source data Fig. 1 [file 44318_2025_504_MOESM3_ESM.zip › Figure 1/1D/P0 replicates/hFKO-P0-4.TIF]

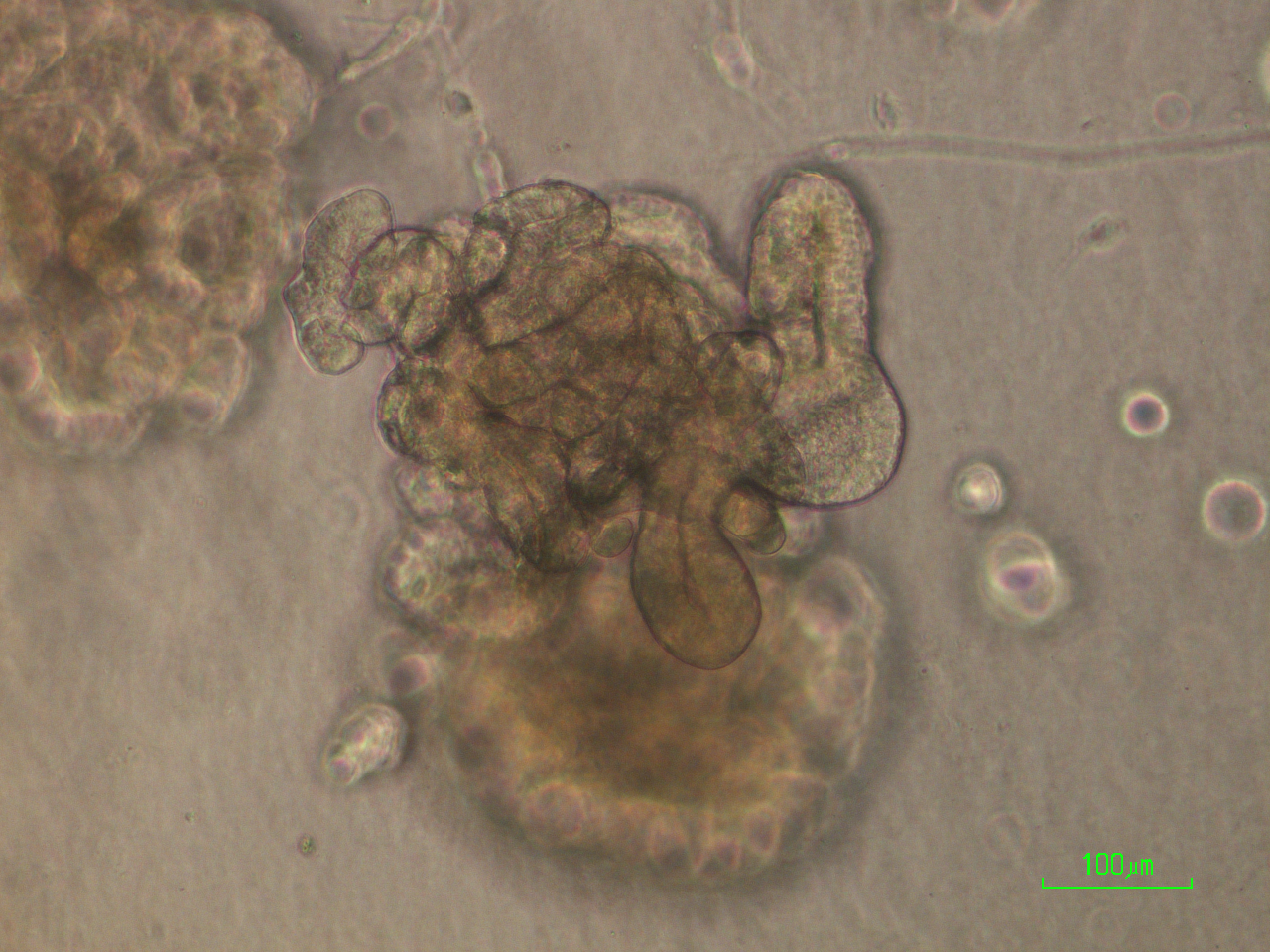

Supplement: Supplementary file 3 — Source data Fig. 1 [file 44318_2025_504_MOESM3_ESM.zip › Figure 1/1D/P0 replicates/hFKO-P0-5.TIF]

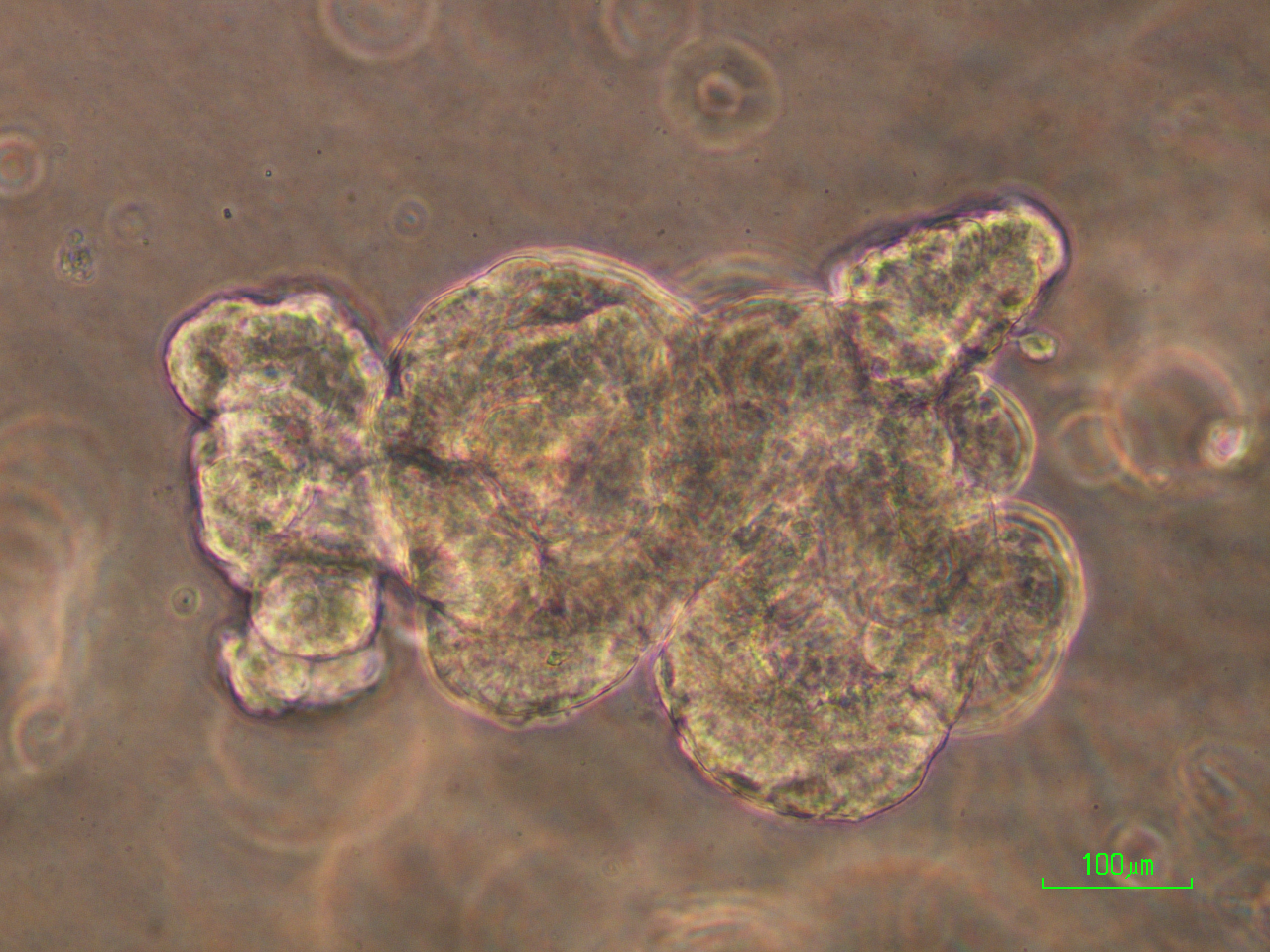

Supplement: Supplementary file 3 — Source data Fig. 1 [file 44318_2025_504_MOESM3_ESM.zip › Figure 1/1D/P2 replicates/hFKO-P2-1.TIF]

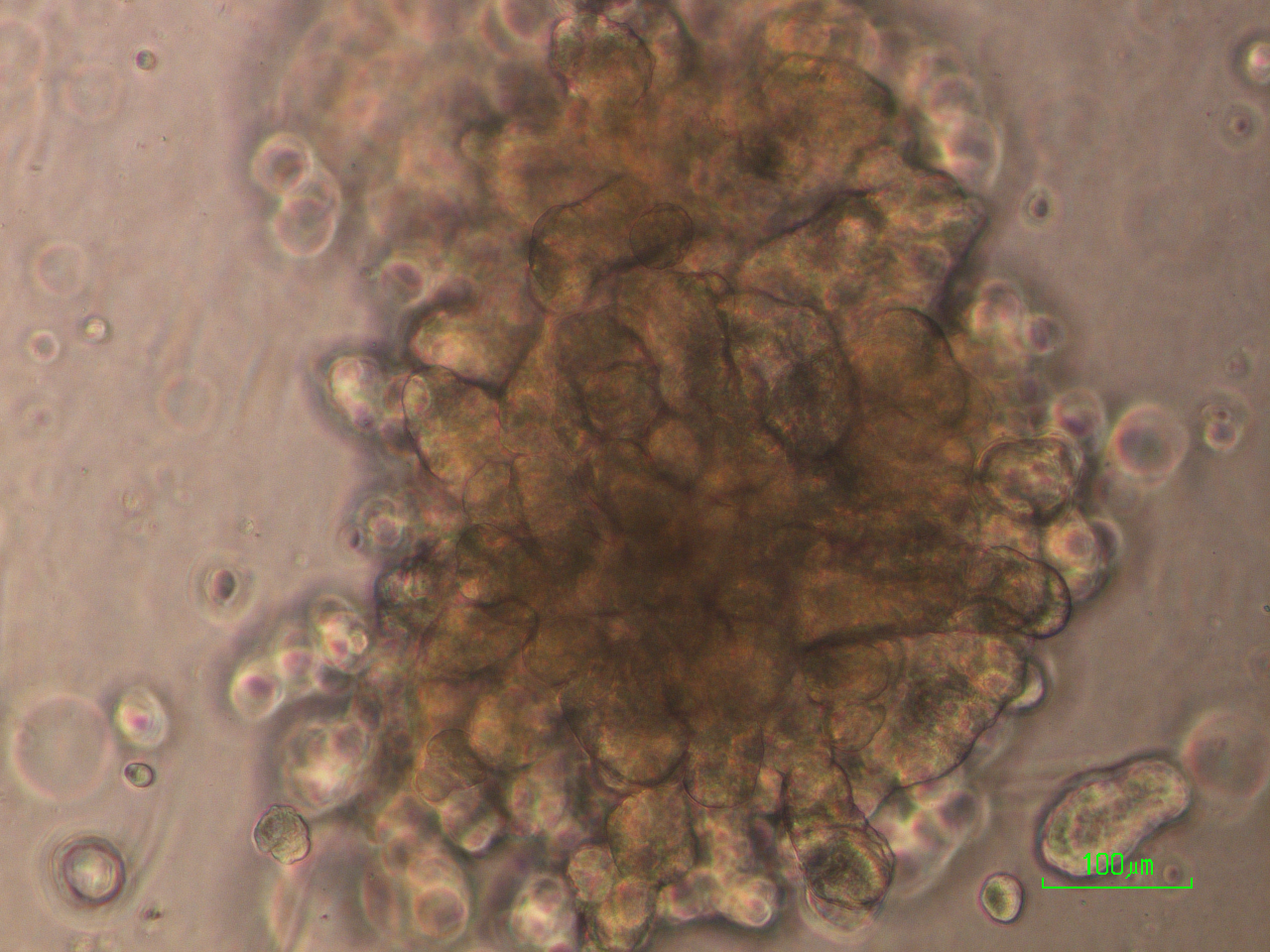

Supplement: Supplementary file 3 — Source data Fig. 1 [file 44318_2025_504_MOESM3_ESM.zip › Figure 1/1D/P2 replicates/hFKO-P2-2.TIF]

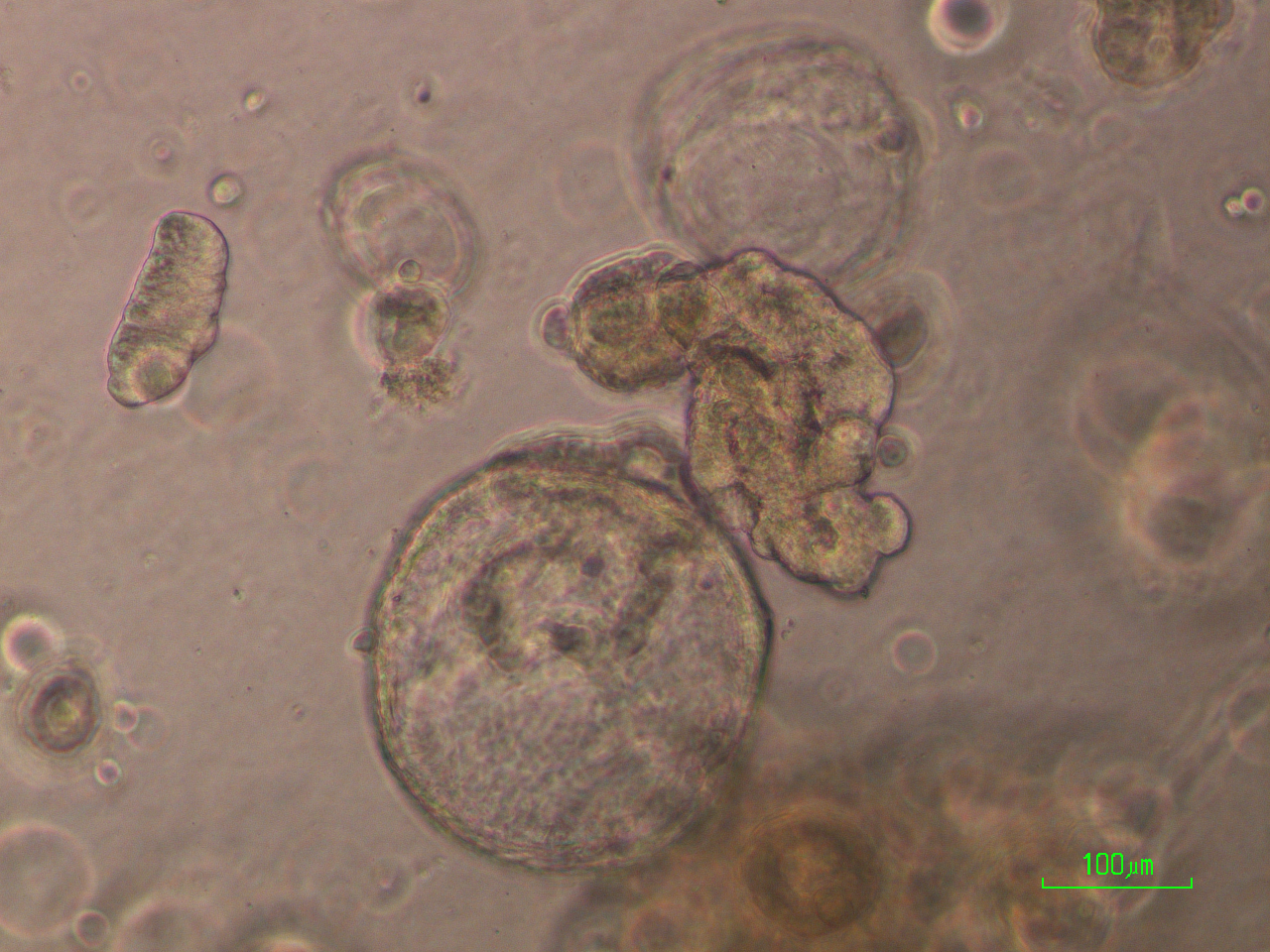

Supplement: Supplementary file 3 — Source data Fig. 1 [file 44318_2025_504_MOESM3_ESM.zip › Figure 1/1D/P2 replicates/hFKO-P2-3.TIF]

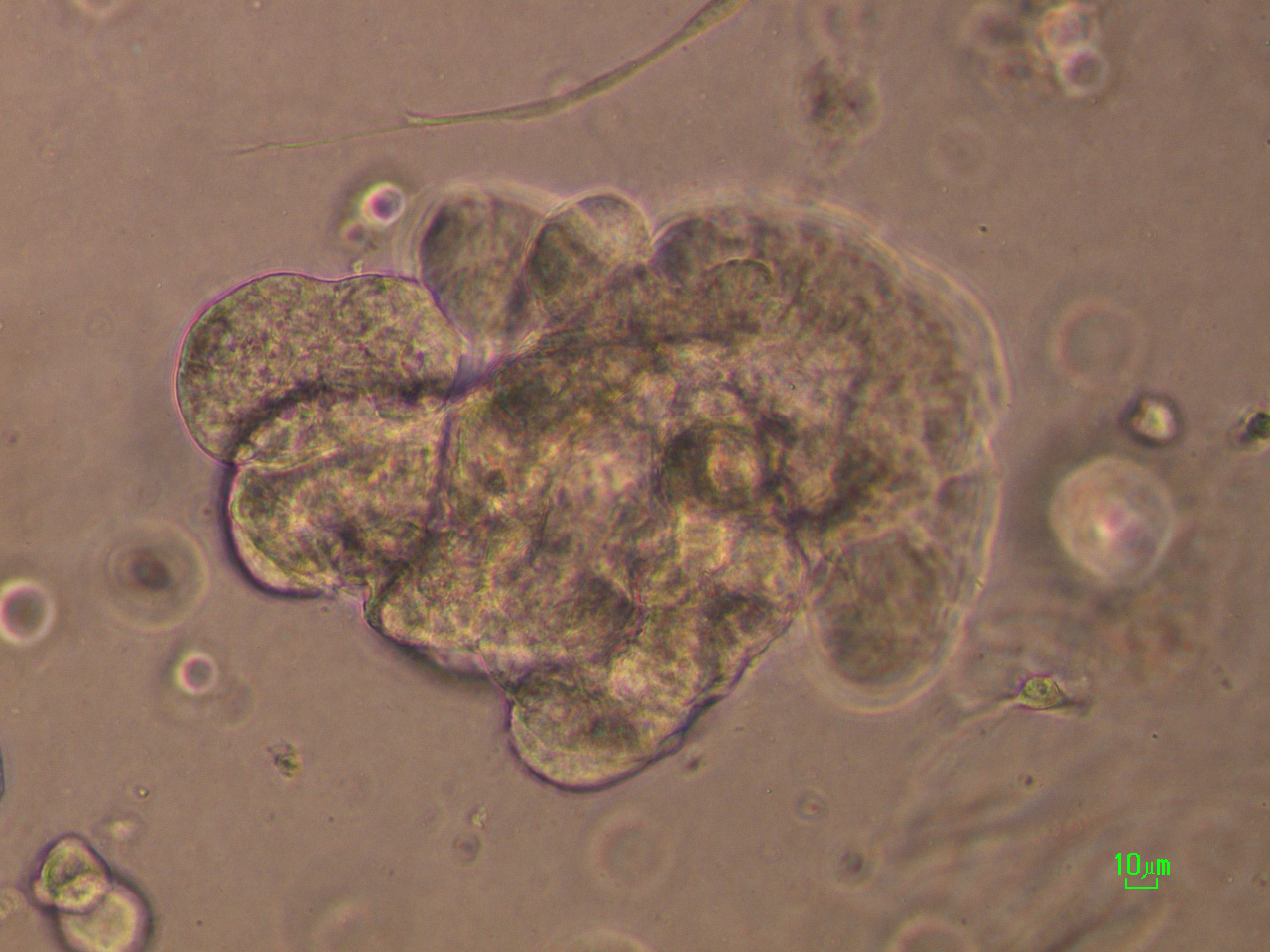

Supplement: Supplementary file 3 — Source data Fig. 1 [file 44318_2025_504_MOESM3_ESM.zip › Figure 1/1D/P2 replicates/hFKO-P2-4.TIF]

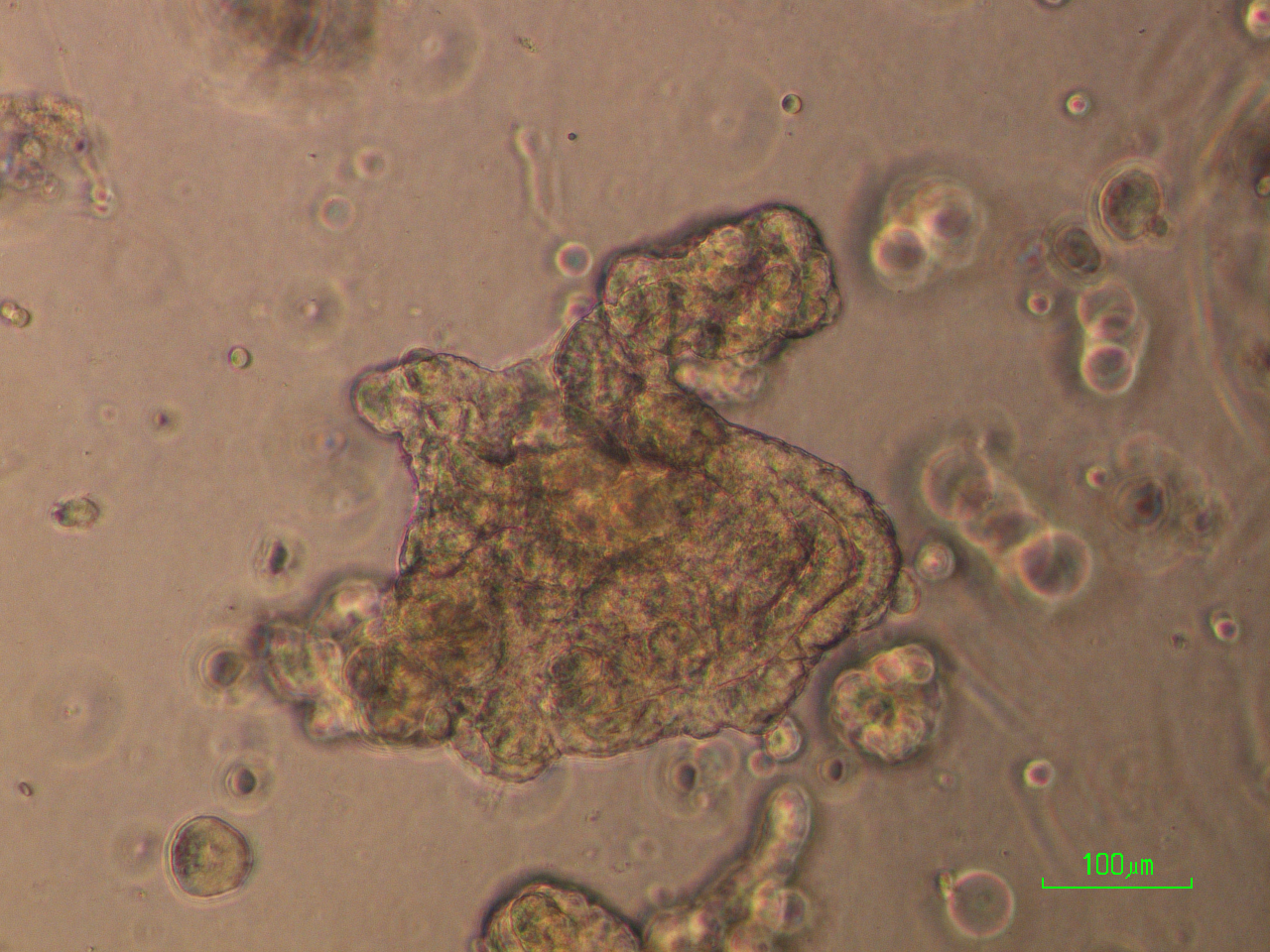

Supplement: Supplementary file 3 — Source data Fig. 1 [file 44318_2025_504_MOESM3_ESM.zip › Figure 1/1D/P2 replicates/hFKO-P2-5.TIF]

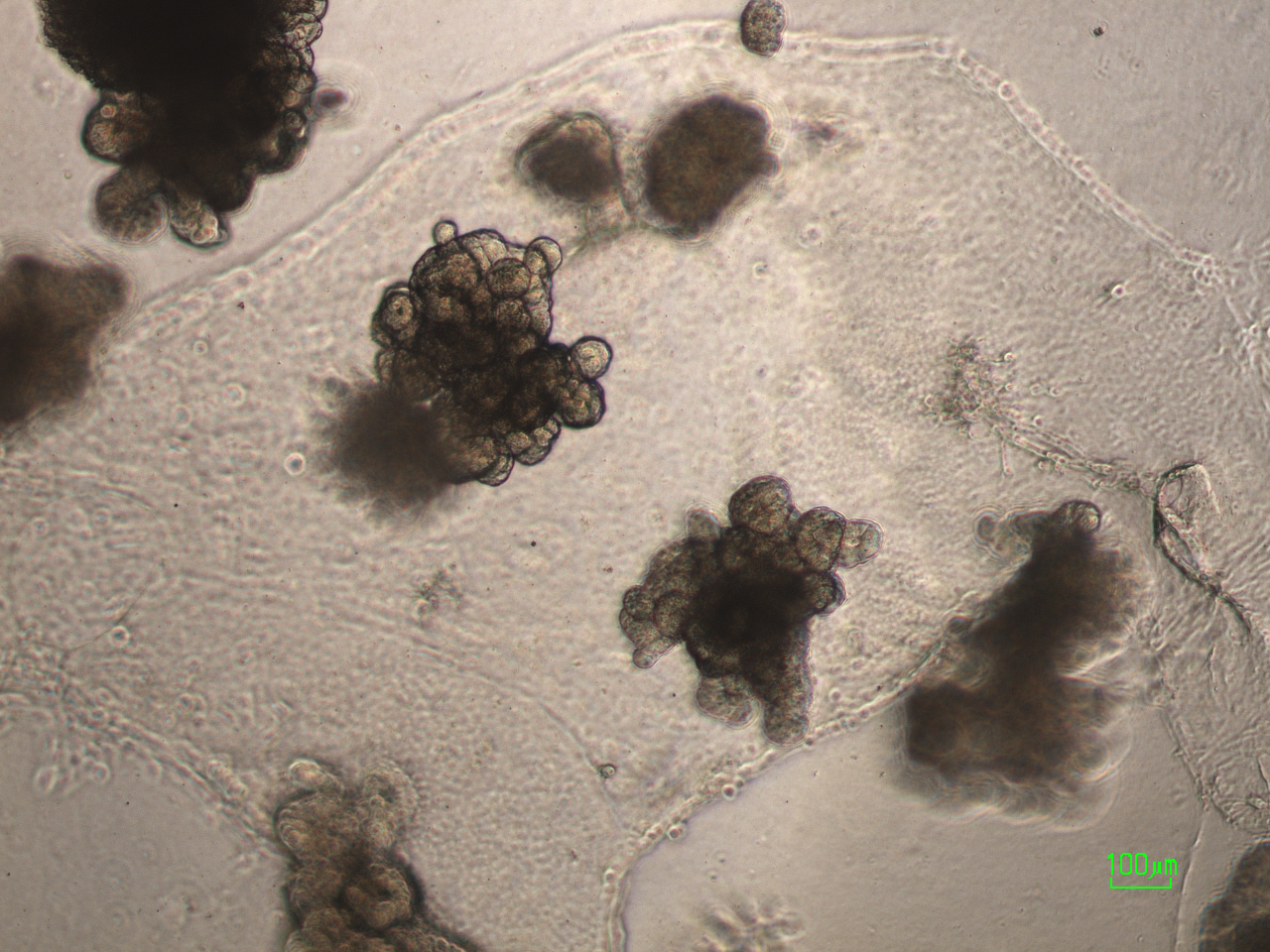

Supplement: Supplementary file 3 — Source data Fig. 1 [file 44318_2025_504_MOESM3_ESM.zip › Figure 1/1D/P8 replicates/hFKO-P8-1.TIF]

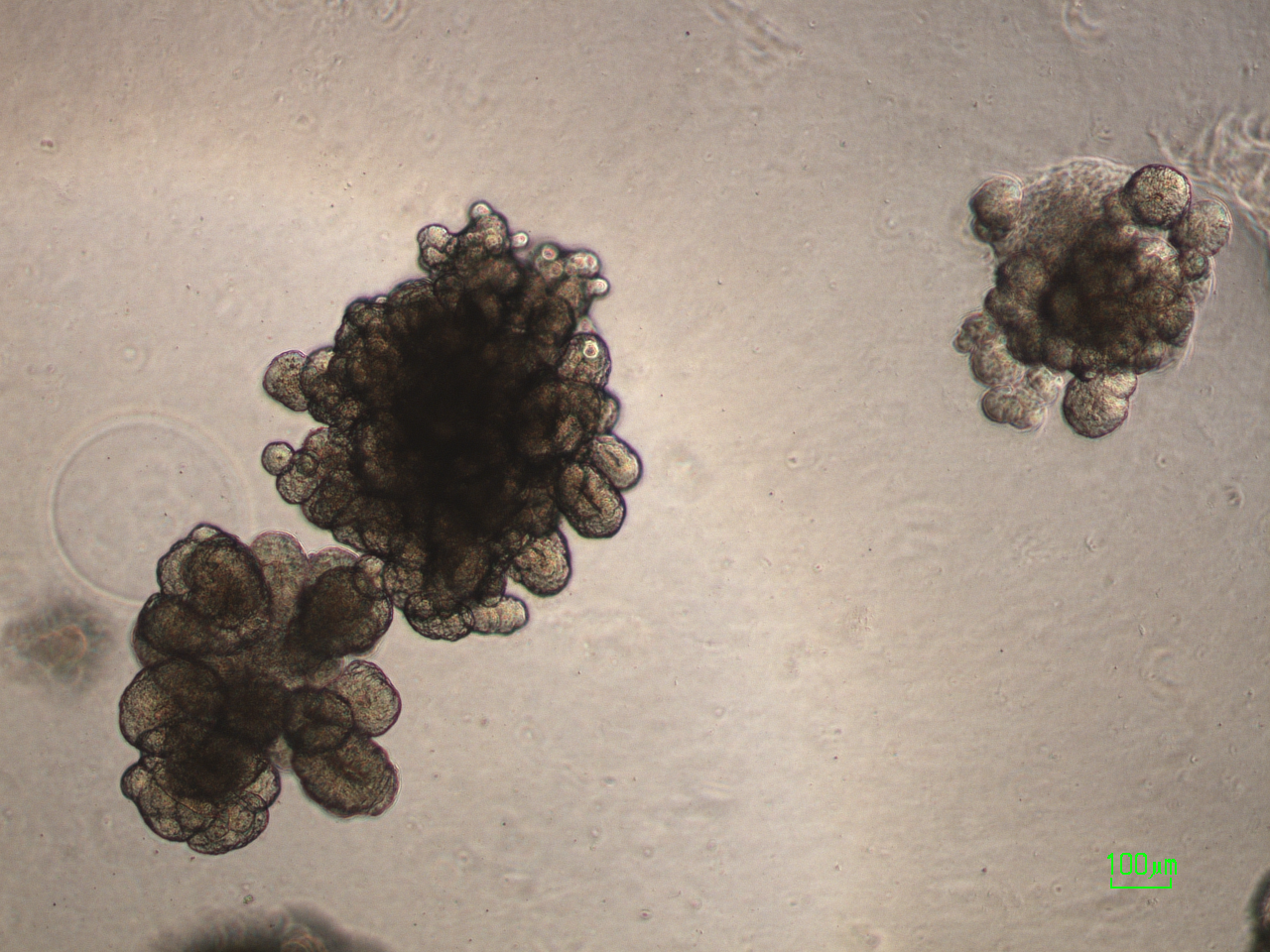

Supplement: Supplementary file 3 — Source data Fig. 1 [file 44318_2025_504_MOESM3_ESM.zip › Figure 1/1D/P8 replicates/hFKO-P8-2.TIF]

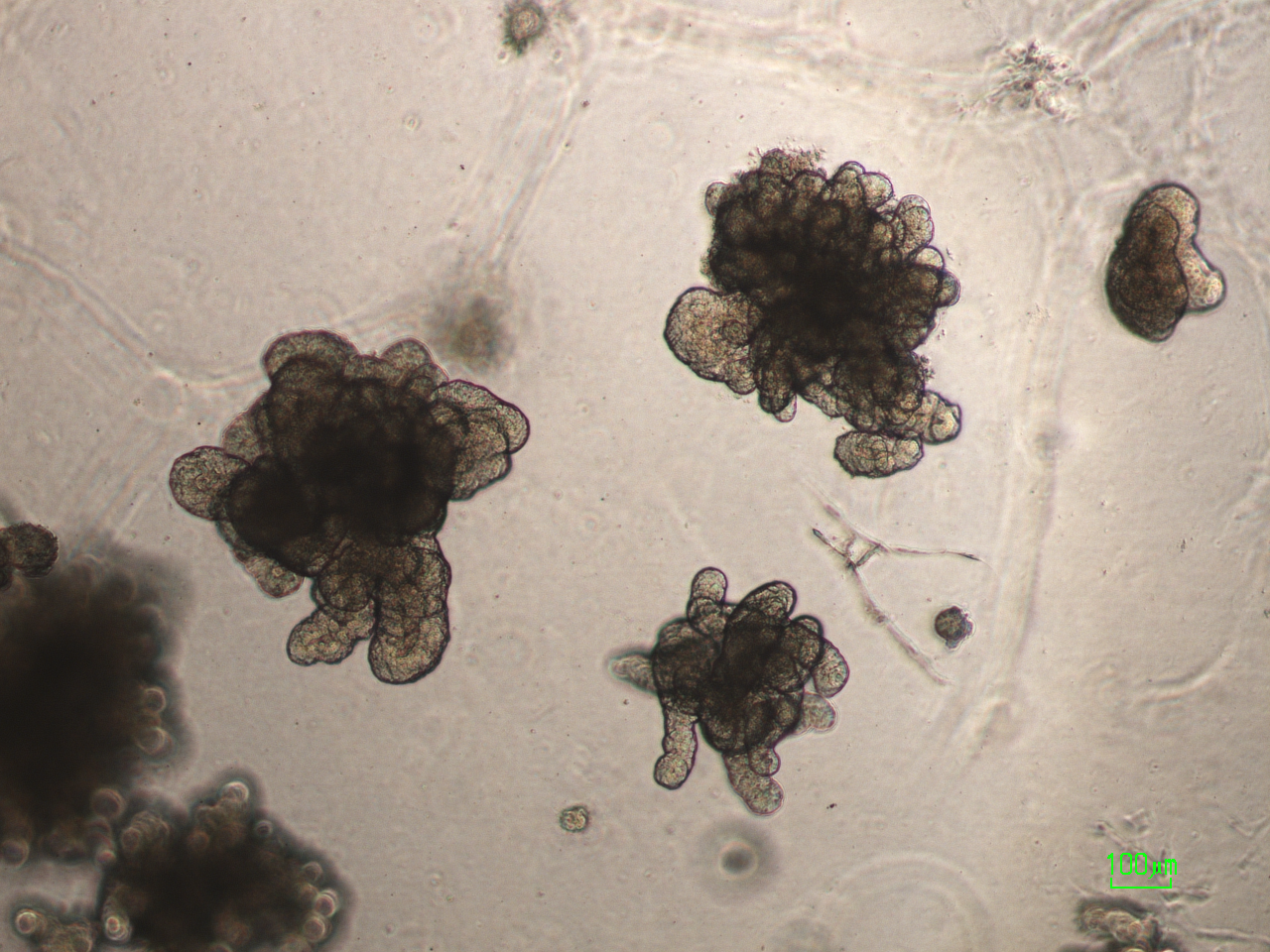

Supplement: Supplementary file 3 — Source data Fig. 1 [file 44318_2025_504_MOESM3_ESM.zip › Figure 1/1D/P8 replicates/hFKO-P8-3.TIF]

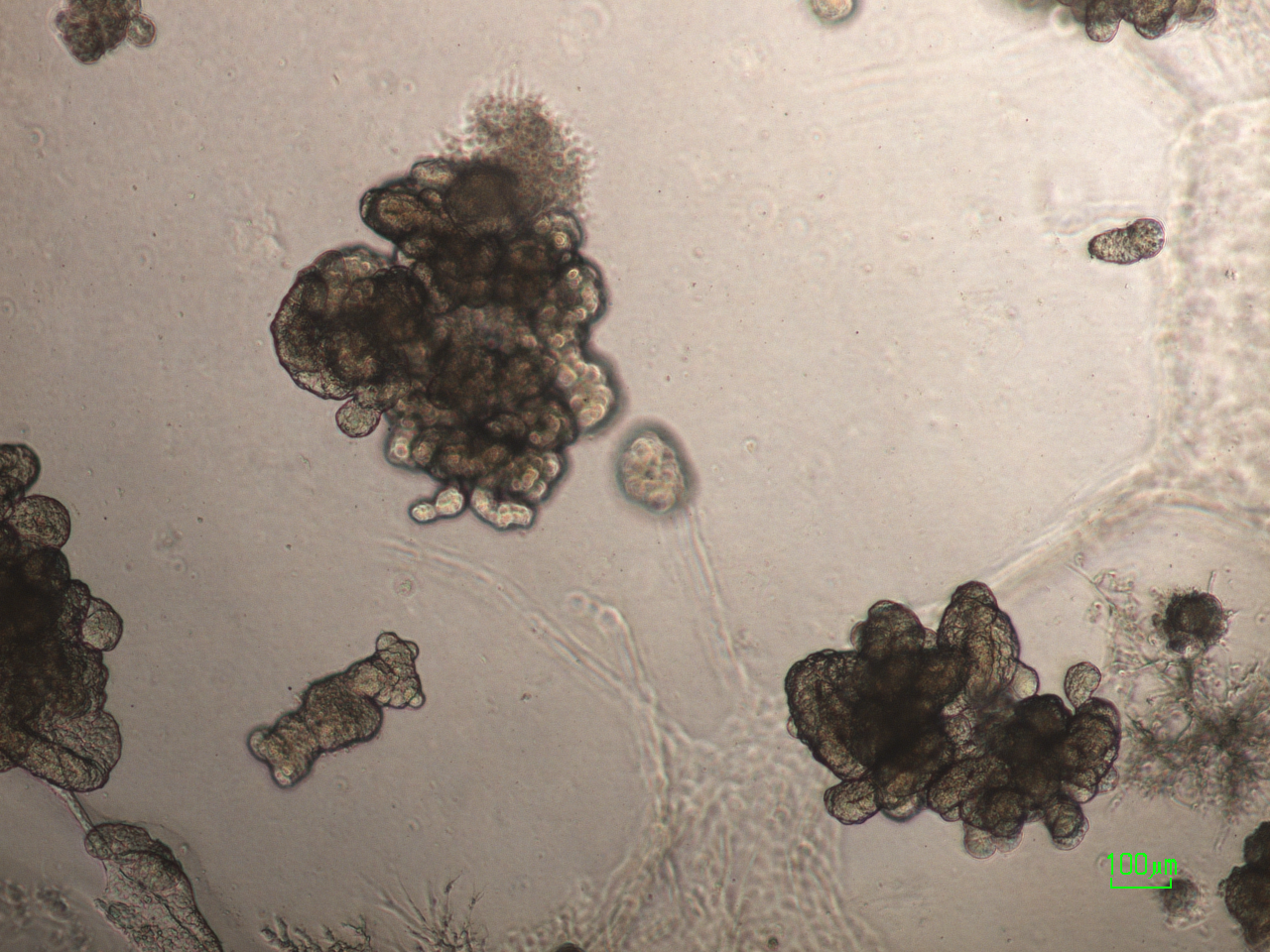

Supplement: Supplementary file 3 — Source data Fig. 1 [file 44318_2025_504_MOESM3_ESM.zip › Figure 1/1D/P8 replicates/hFKO-P8-4.TIF]

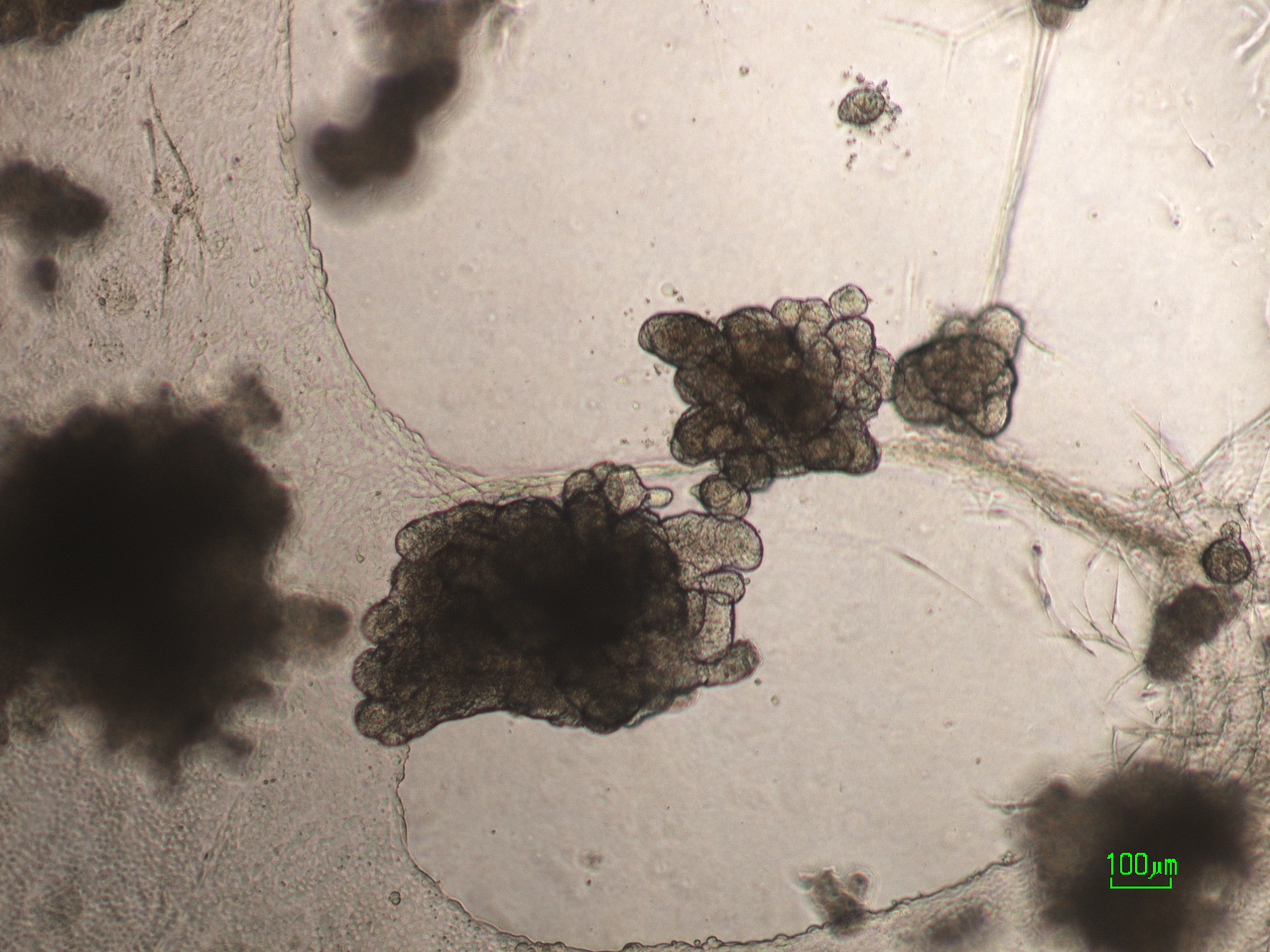

Supplement: Supplementary file 3 — Source data Fig. 1 [file 44318_2025_504_MOESM3_ESM.zip › Figure 1/1D/P8 replicates/hFKO-P8-5.TIF]

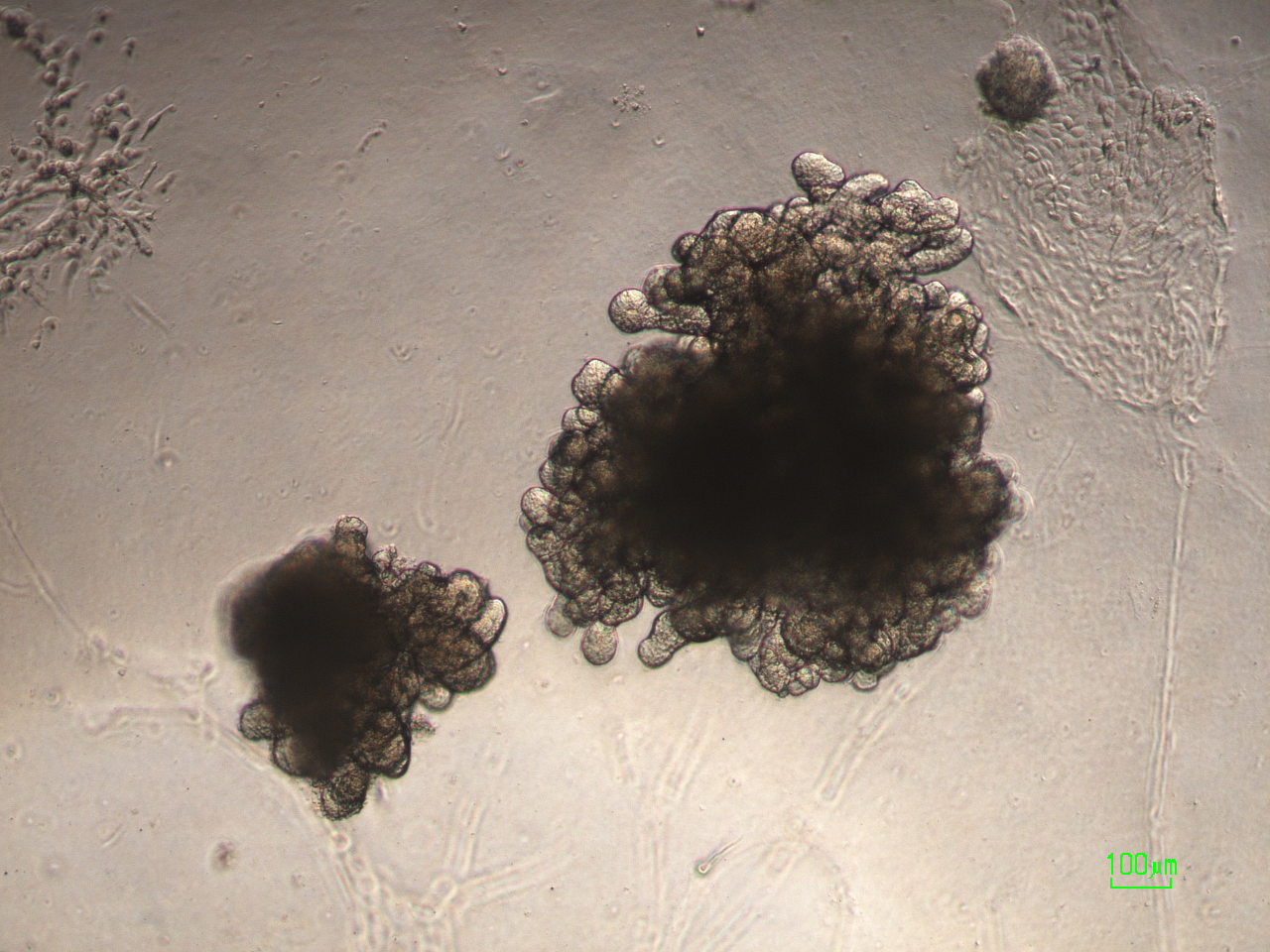

Supplement: Supplementary file 3 — Source data Fig. 1 [file 44318_2025_504_MOESM3_ESM.zip › Figure 1/1D/P8 replicates/hFKO-P8-6.TIF]

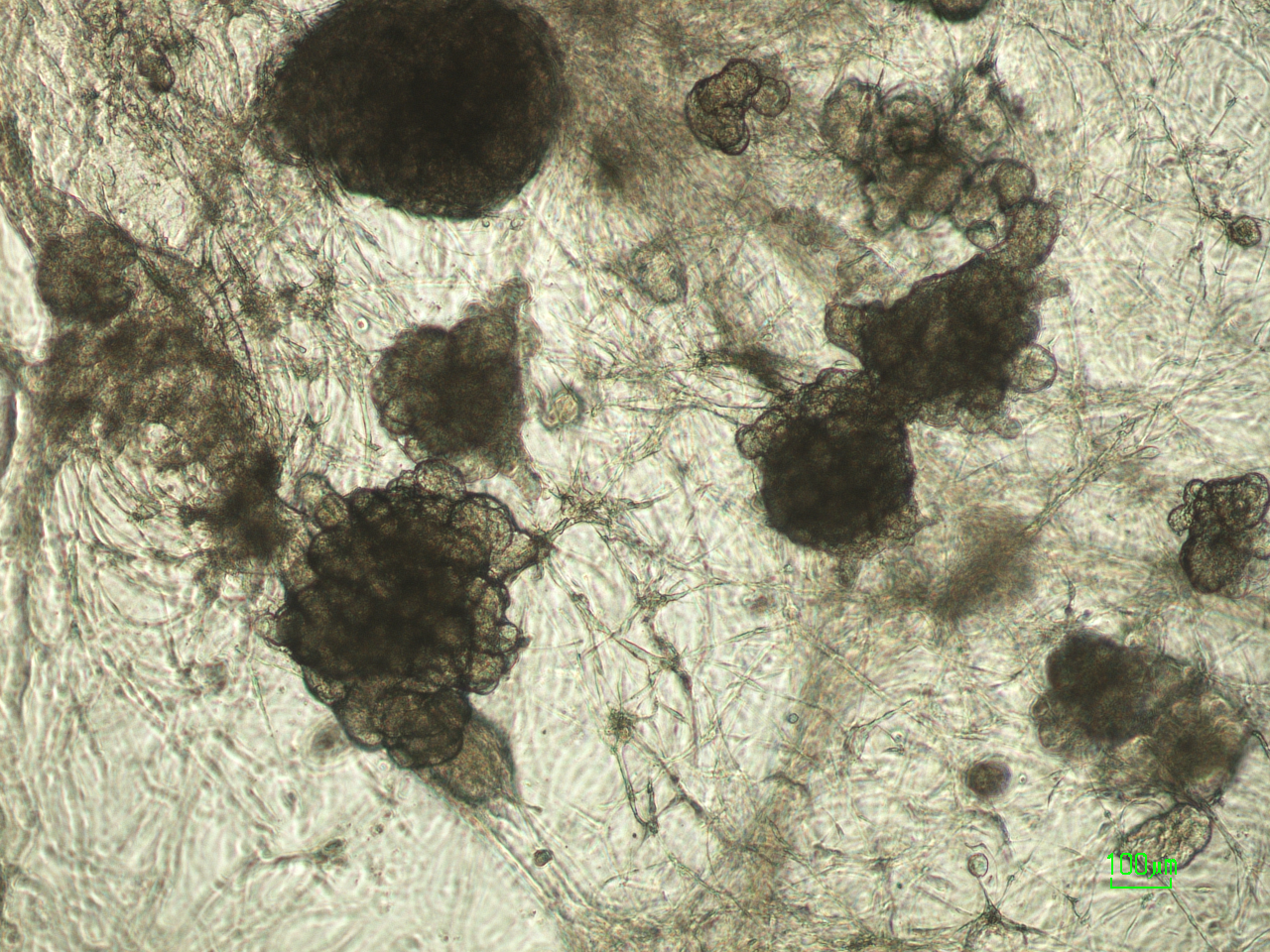

Supplement: Supplementary file 3 — Source data Fig. 1 [file 44318_2025_504_MOESM3_ESM.zip › Figure 1/1E/hFKOs-no-forskolin-treatment.TIF]

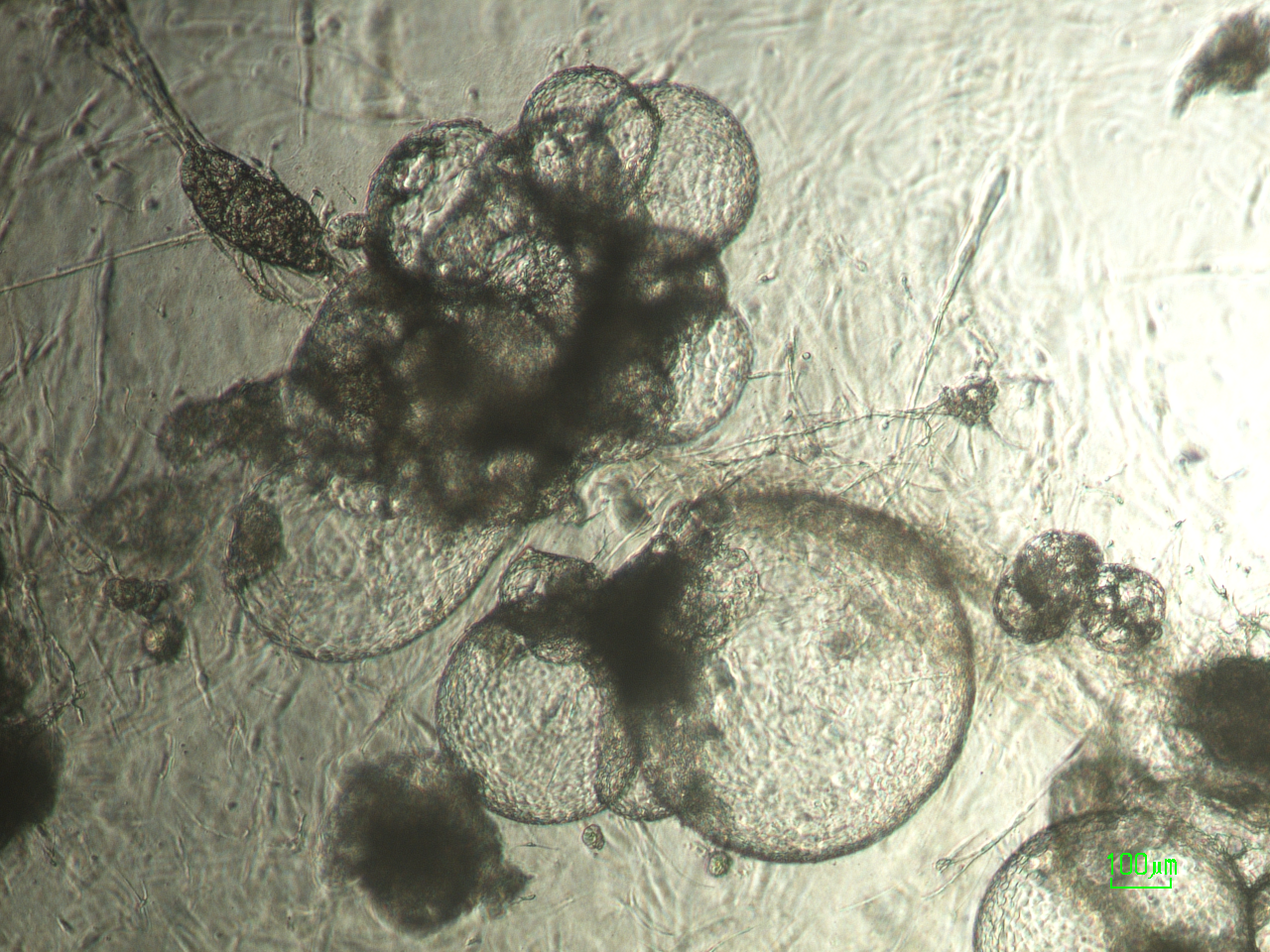

Supplement: Supplementary file 3 — Source data Fig. 1 [file 44318_2025_504_MOESM3_ESM.zip › Figure 1/1E/hFKOs-with-forskolin-treatment.TIF]

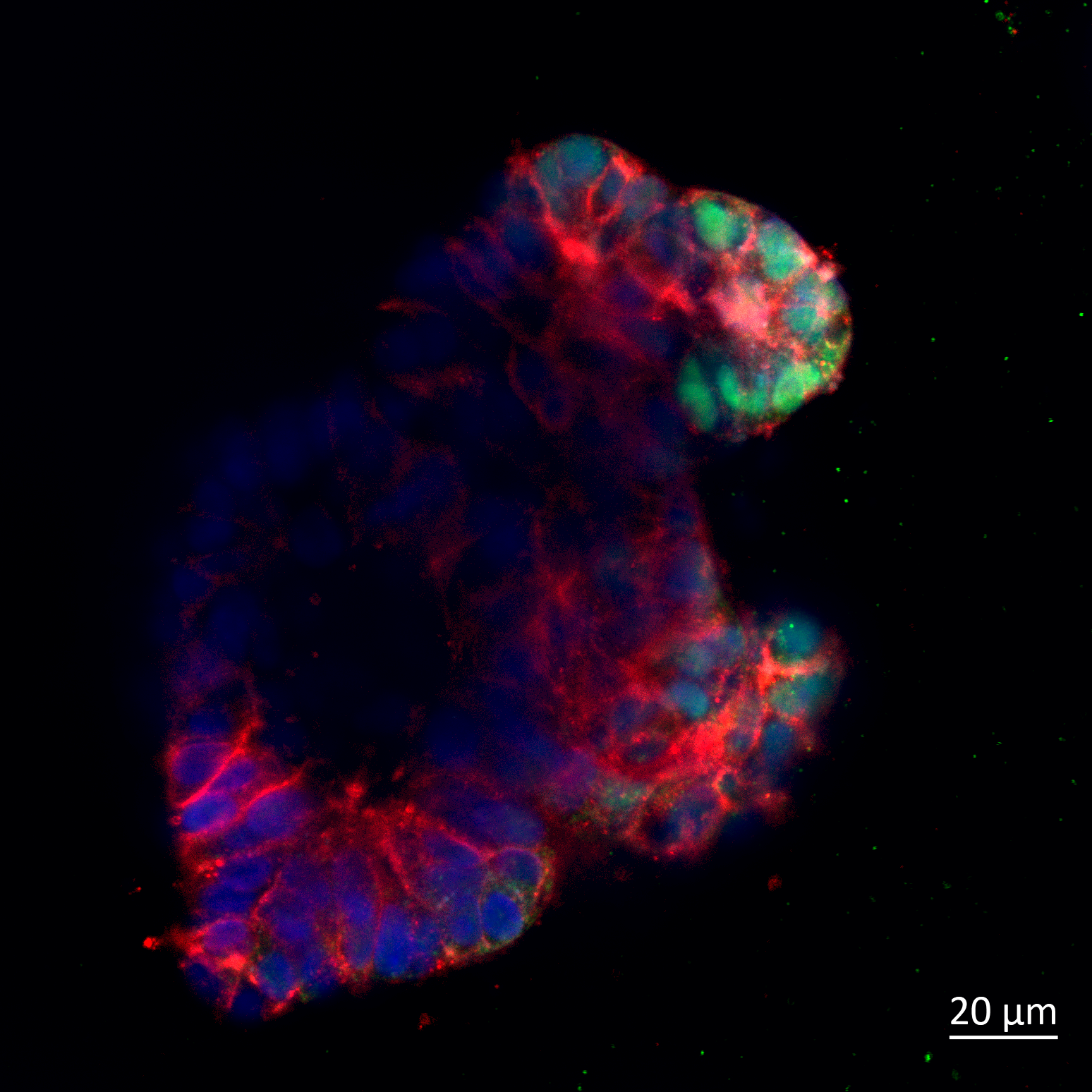

Supplement: Supplementary file 4 — Source data Fig. 2 [file 44318_2025_504_MOESM4_ESM.zip › Figure 2/2A/hFKO-NCAM1-red-SIX2-green-DAPI-blue.tif]

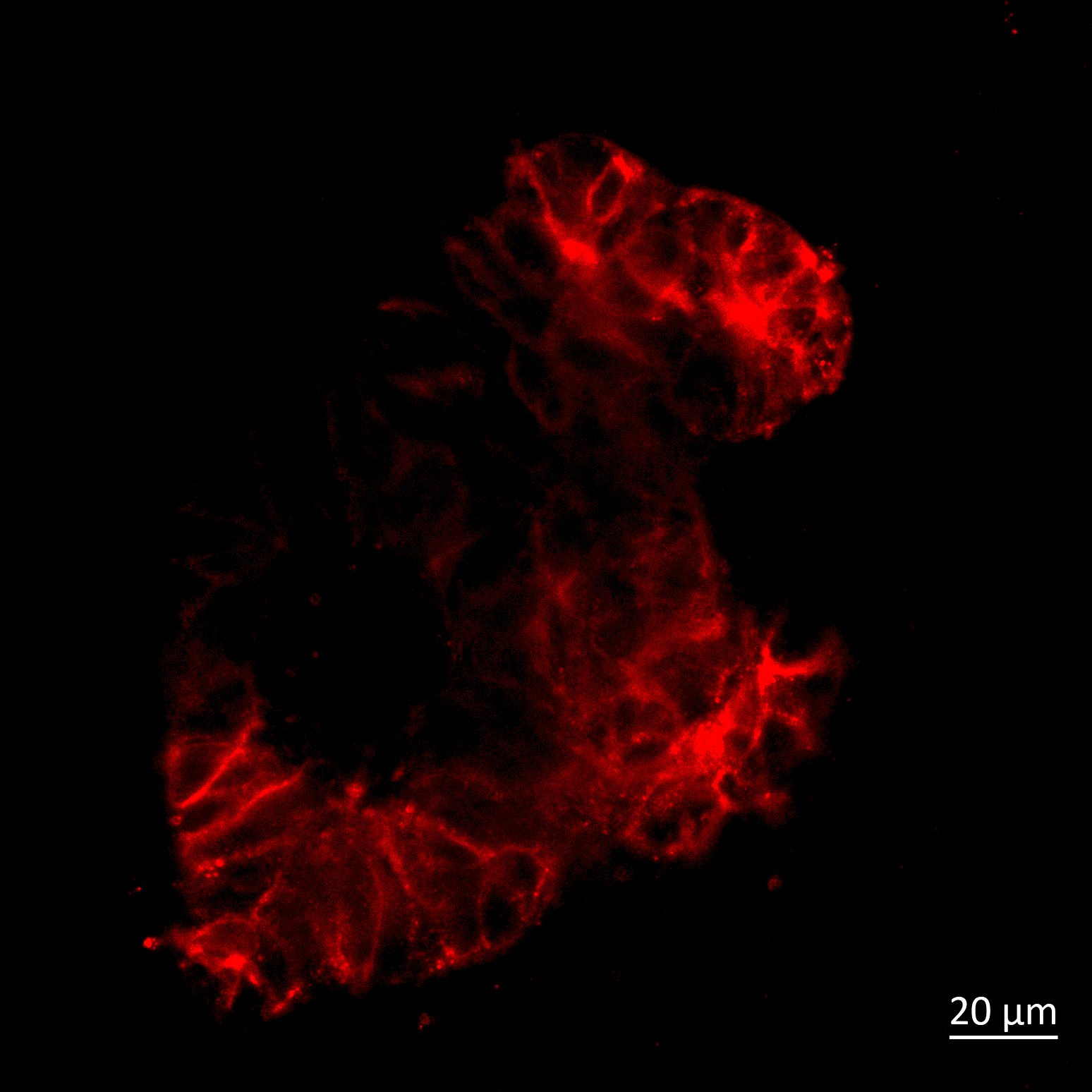

Supplement: Supplementary file 4 — Source data Fig. 2 [file 44318_2025_504_MOESM4_ESM.zip › Figure 2/2A/hFKO-NCAM1-red.tif]

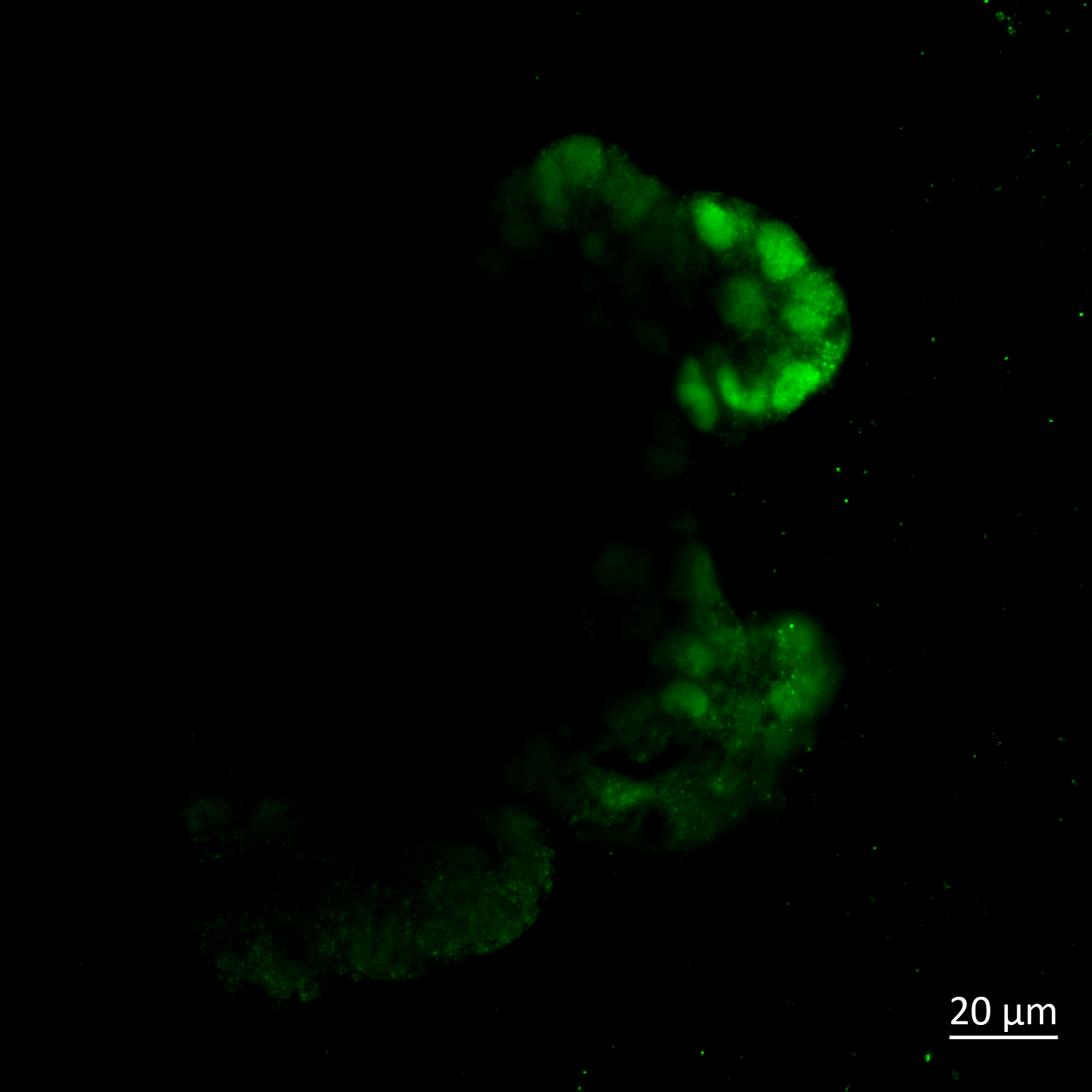

Supplement: Supplementary file 4 — Source data Fig. 2 [file 44318_2025_504_MOESM4_ESM.zip › Figure 2/2A/hFKO-SIX2-green.tif]

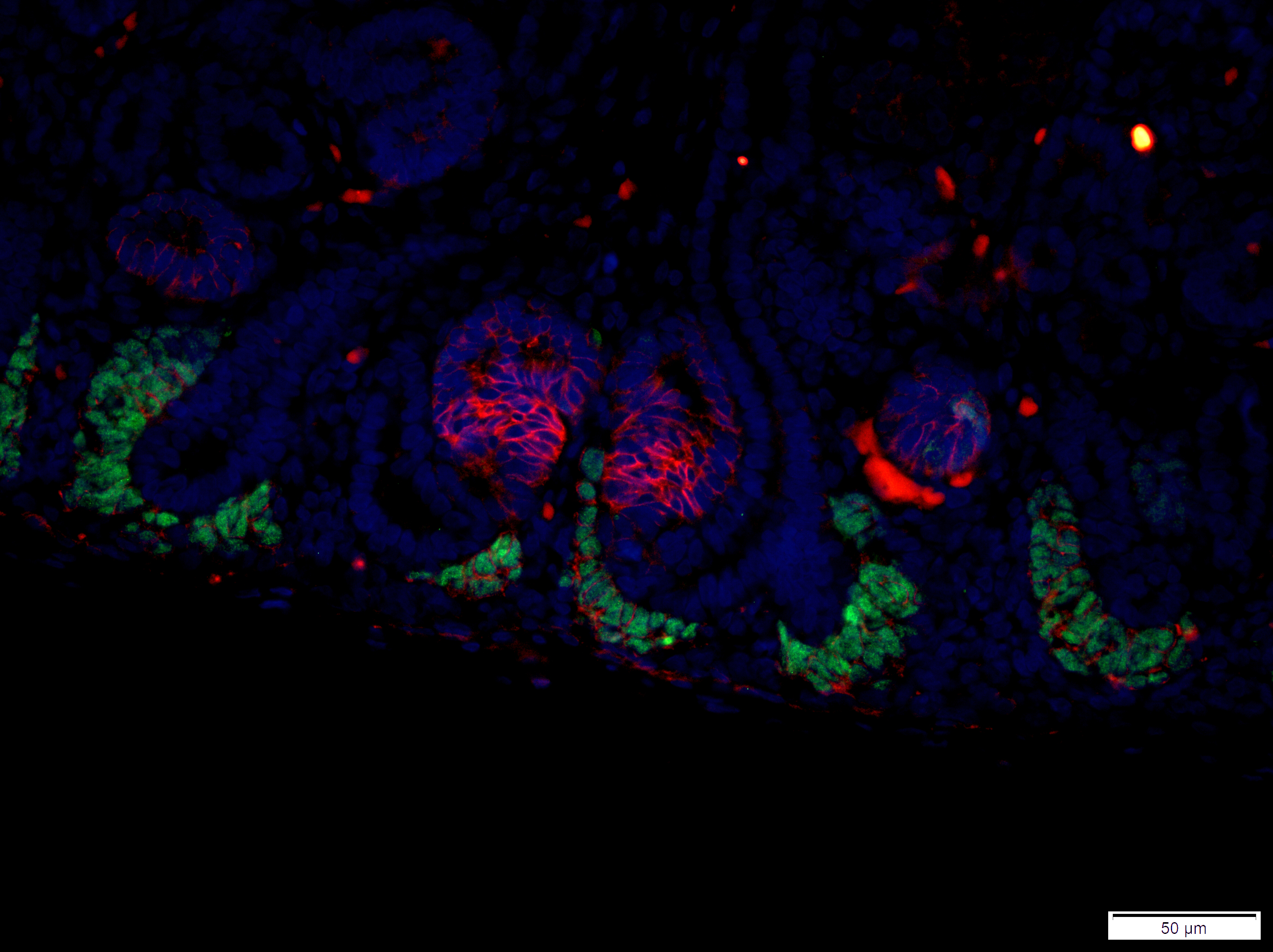

Supplement: Supplementary file 4 — Source data Fig. 2 [file 44318_2025_504_MOESM4_ESM.zip › Figure 2/2A/human-fetal-kidney-slide-NCAM1-red-SIX2-green-DAPI-blue.tiff]

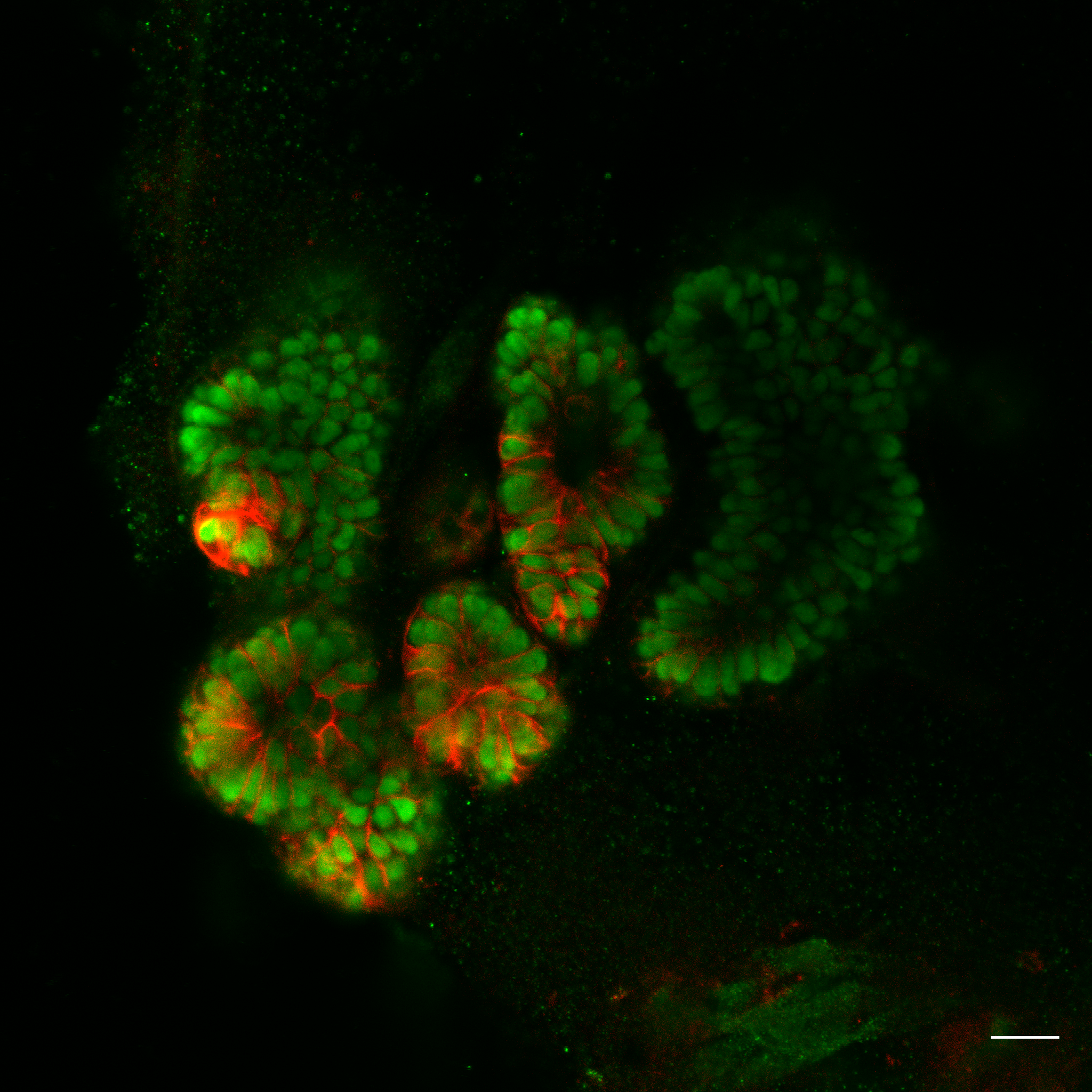

Supplement: Supplementary file 4 — Source data Fig. 2 [file 44318_2025_504_MOESM4_ESM.zip › Figure 2/2B/hFKO-NCAM1-red-PAX2-green-scalebar-20um.tif]

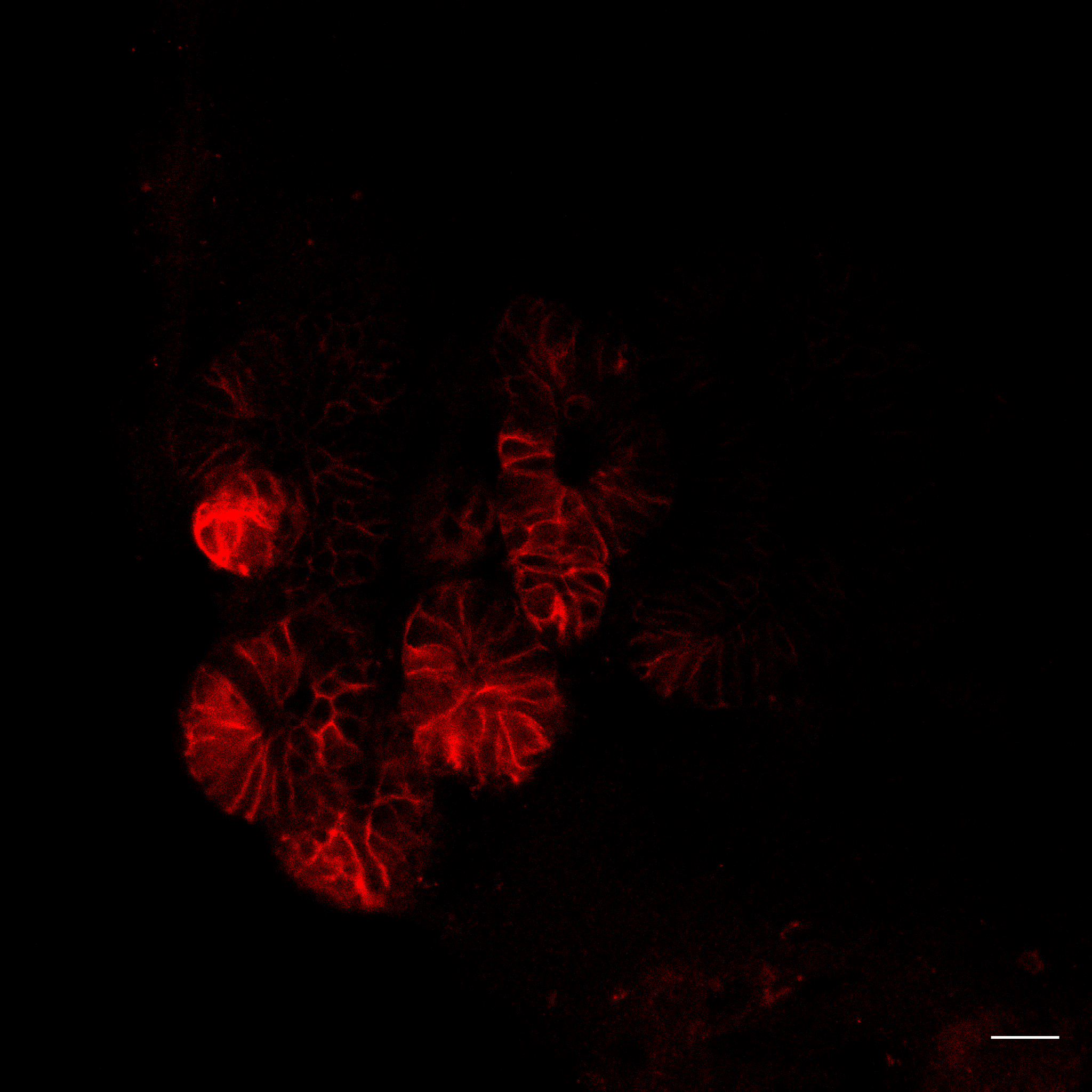

Supplement: Supplementary file 4 — Source data Fig. 2 [file 44318_2025_504_MOESM4_ESM.zip › Figure 2/2B/hFKO-NCAM1-red-scalebar-20um.tif]

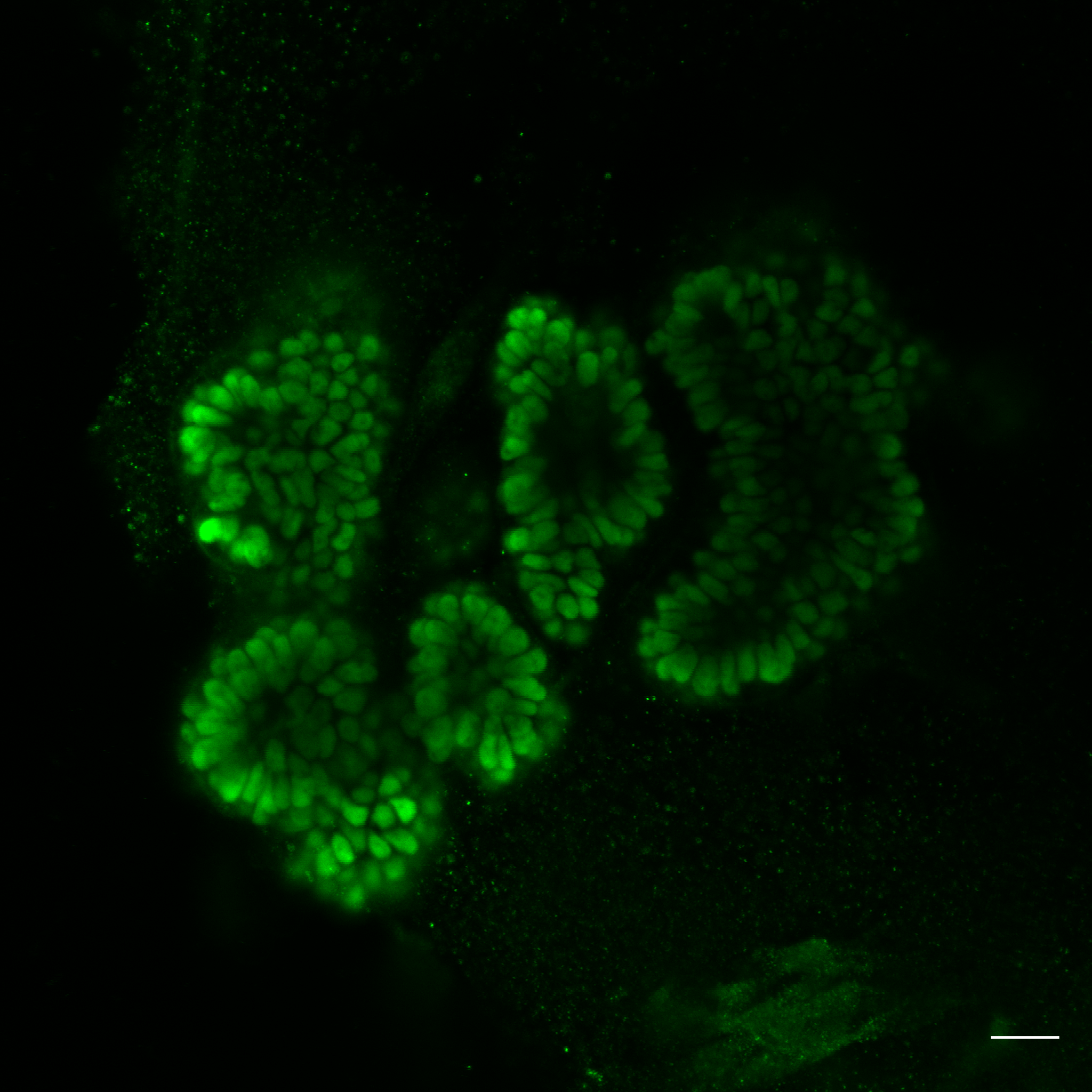

Supplement: Supplementary file 4 — Source data Fig. 2 [file 44318_2025_504_MOESM4_ESM.zip › Figure 2/2B/hFKO-PAX2-green-scalebar-20um.tif]

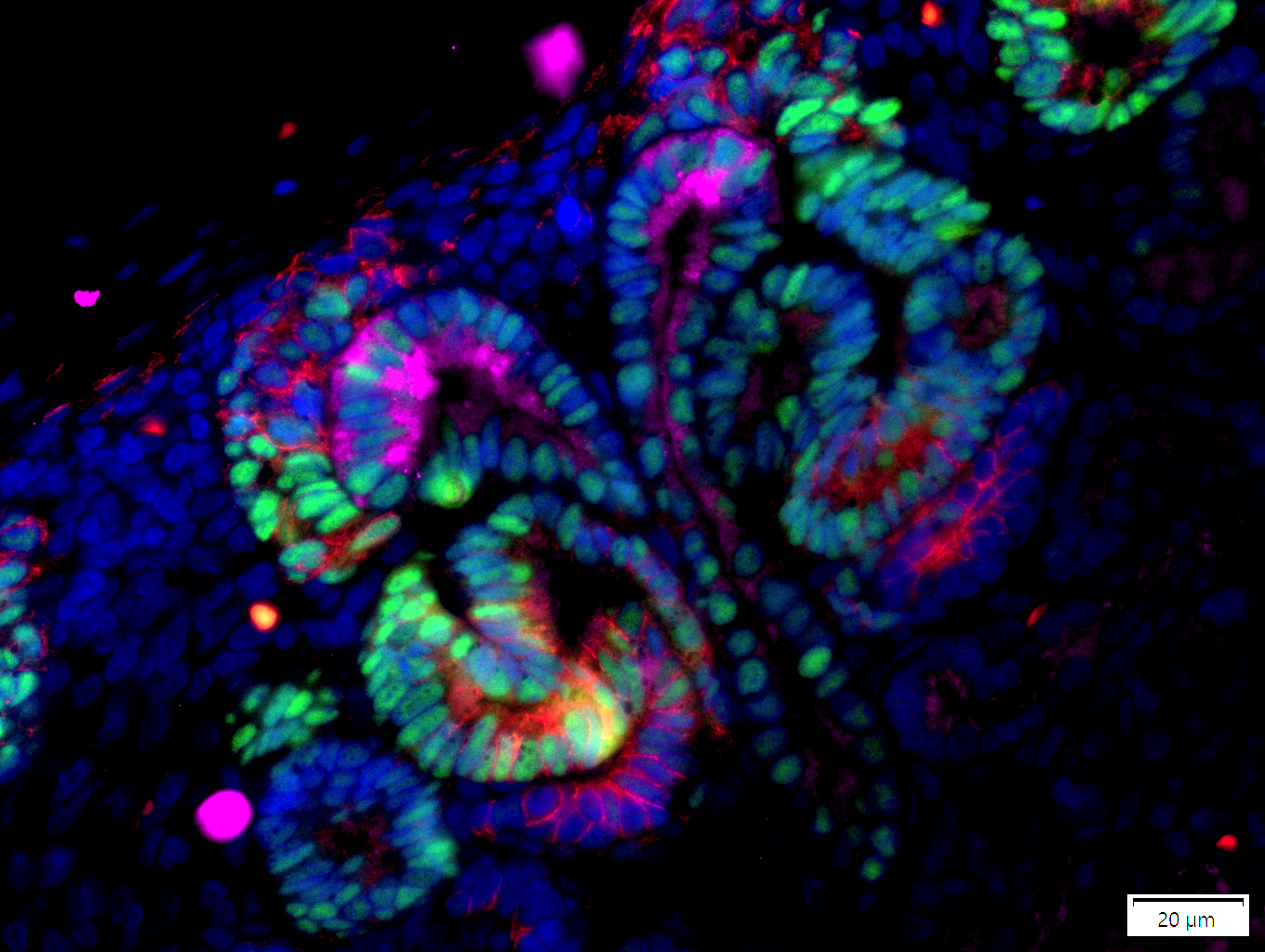

Supplement: Supplementary file 4 — Source data Fig. 2 [file 44318_2025_504_MOESM4_ESM.zip › Figure 2/2B/human-fetal-kidney-slide-NCAM1-red-PAX2-green-DAPI-blue.tif]

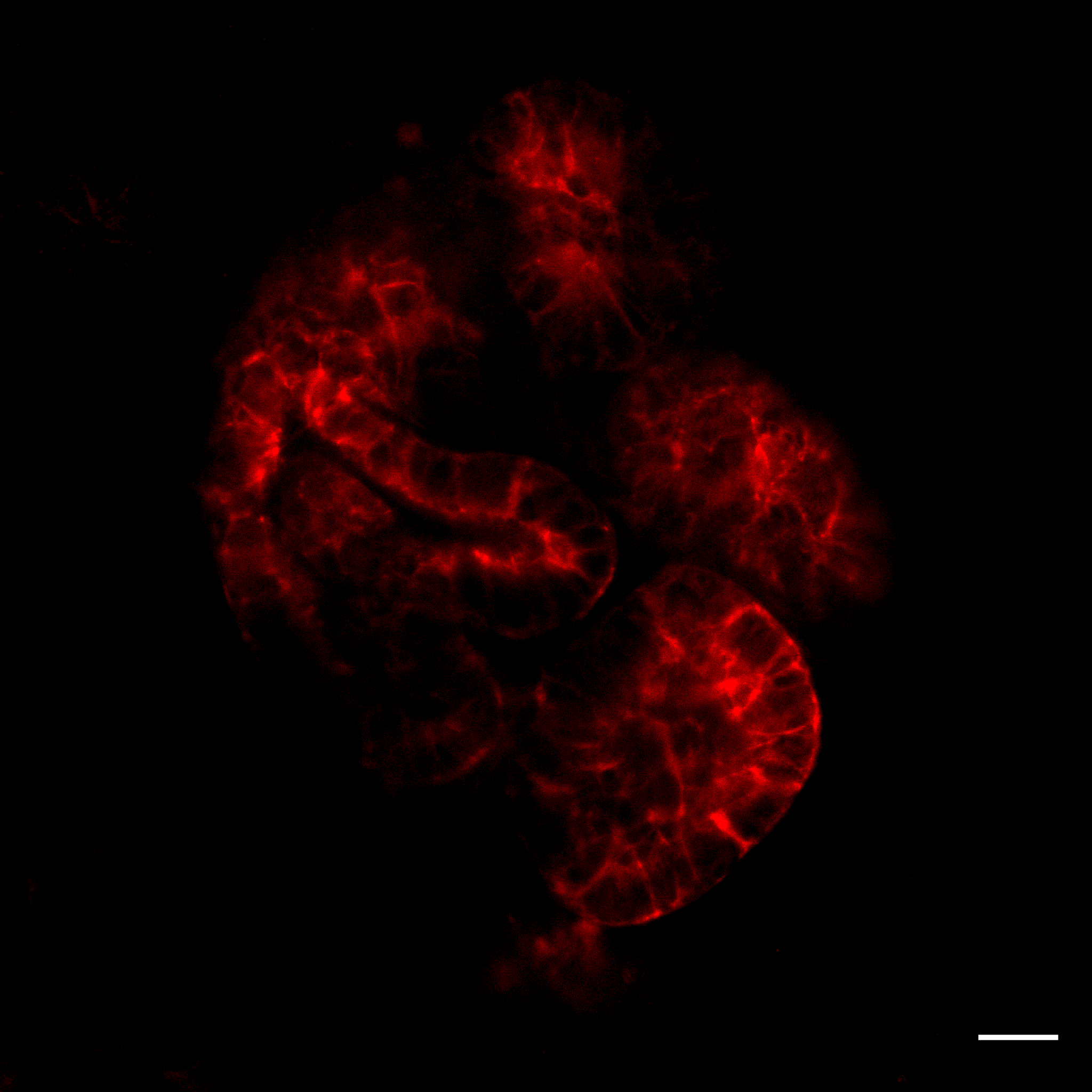

Supplement: Supplementary file 4 — Source data Fig. 2 [file 44318_2025_504_MOESM4_ESM.zip › Figure 2/2C/hFKO-CDH6-red-scalebar-20um.tif]

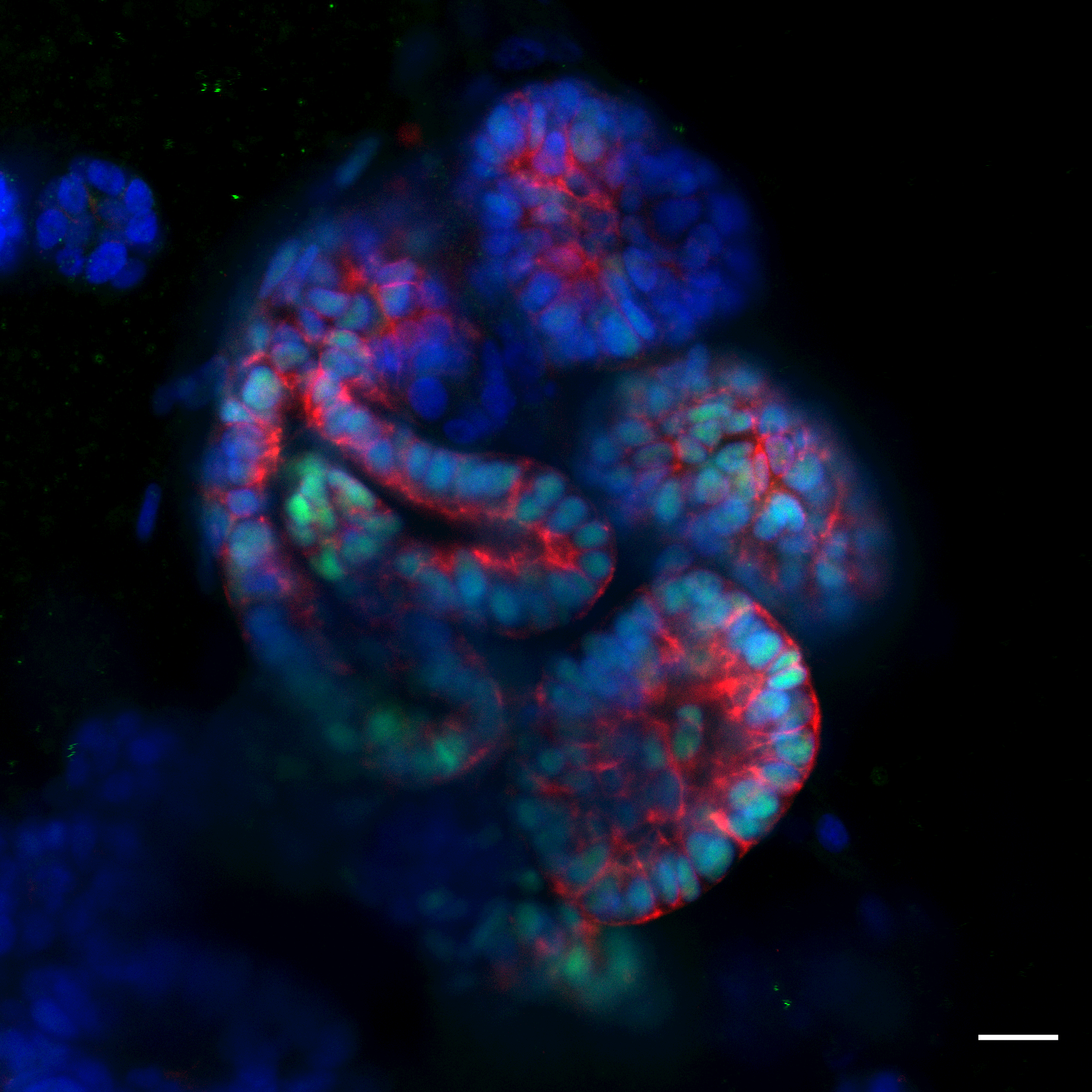

Supplement: Supplementary file 4 — Source data Fig. 2 [file 44318_2025_504_MOESM4_ESM.zip › Figure 2/2C/hFKO-CDH6-red-WT1-green-DAPI-blue-scalebar-20um.tif]

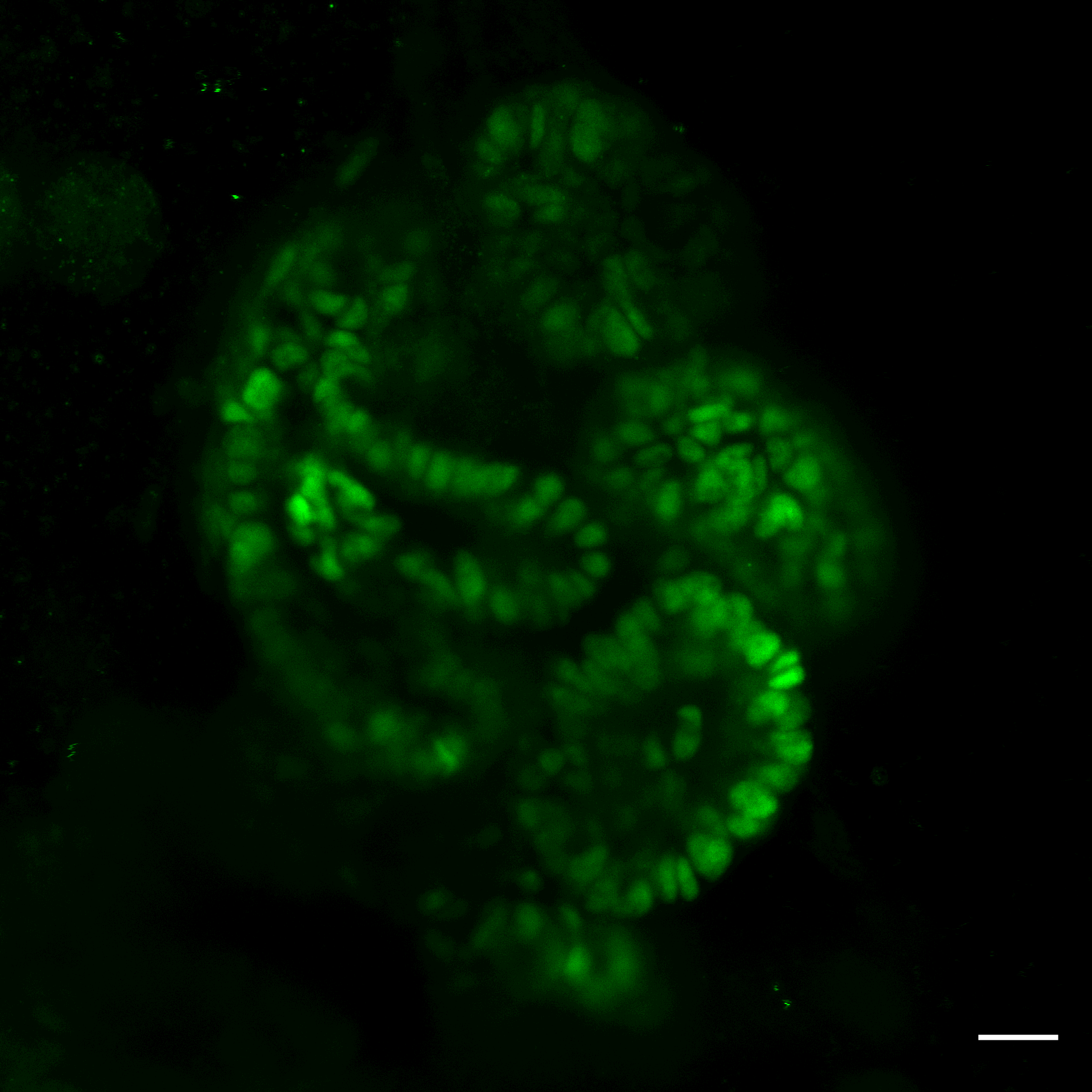

Supplement: Supplementary file 4 — Source data Fig. 2 [file 44318_2025_504_MOESM4_ESM.zip › Figure 2/2C/hFKO-WT1-green-scalebar-20um.tif]

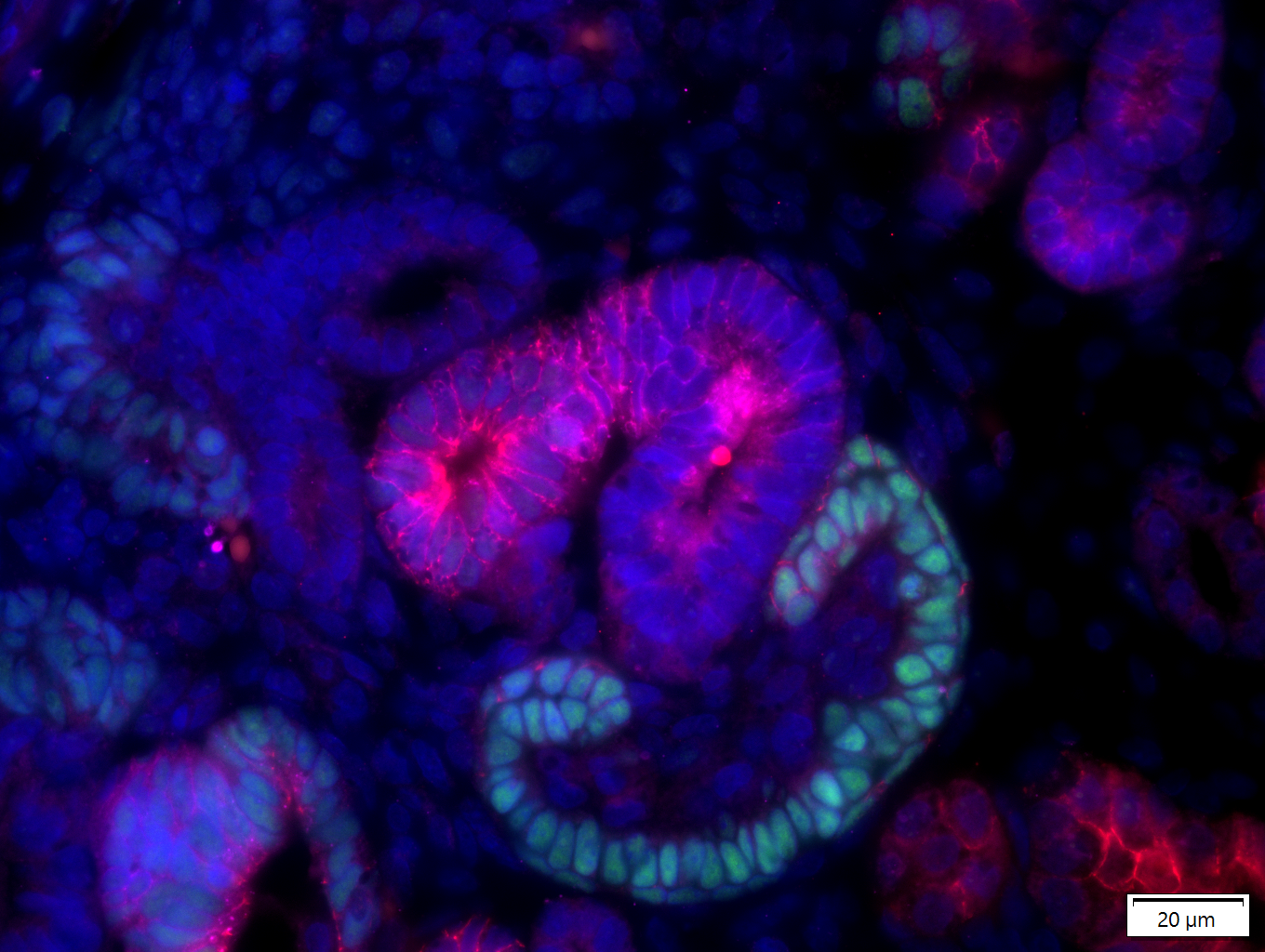

Supplement: Supplementary file 4 — Source data Fig. 2 [file 44318_2025_504_MOESM4_ESM.zip › Figure 2/2C/human-fetal-kidney-slide-CDH6-red-WT1-green-JAG1-purple-DAPI-blue.tif]

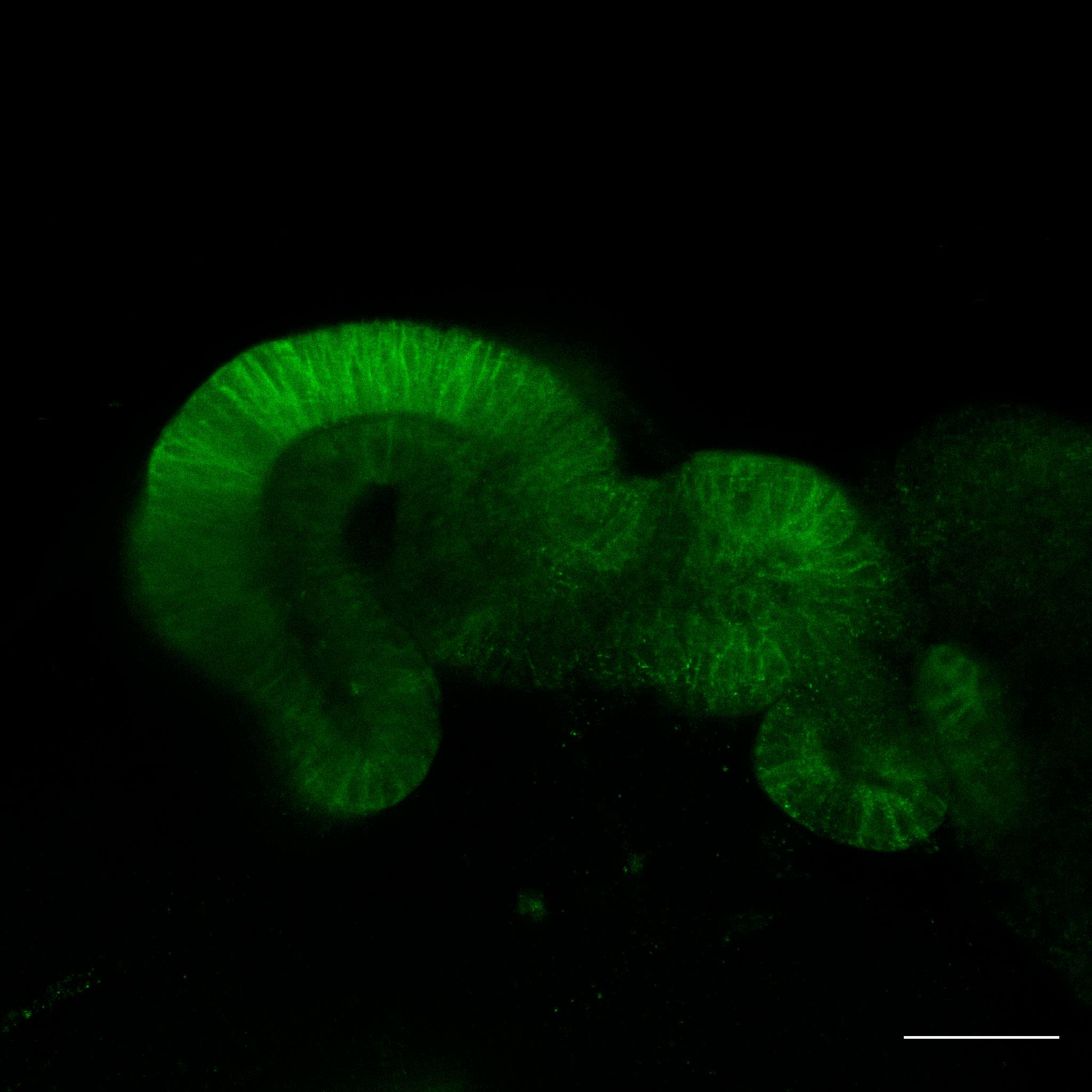

Supplement: Supplementary file 4 — Source data Fig. 2 [file 44318_2025_504_MOESM4_ESM.zip › Figure 2/2D/hFKOs-ECAD-green-scalebar-20um.tif]

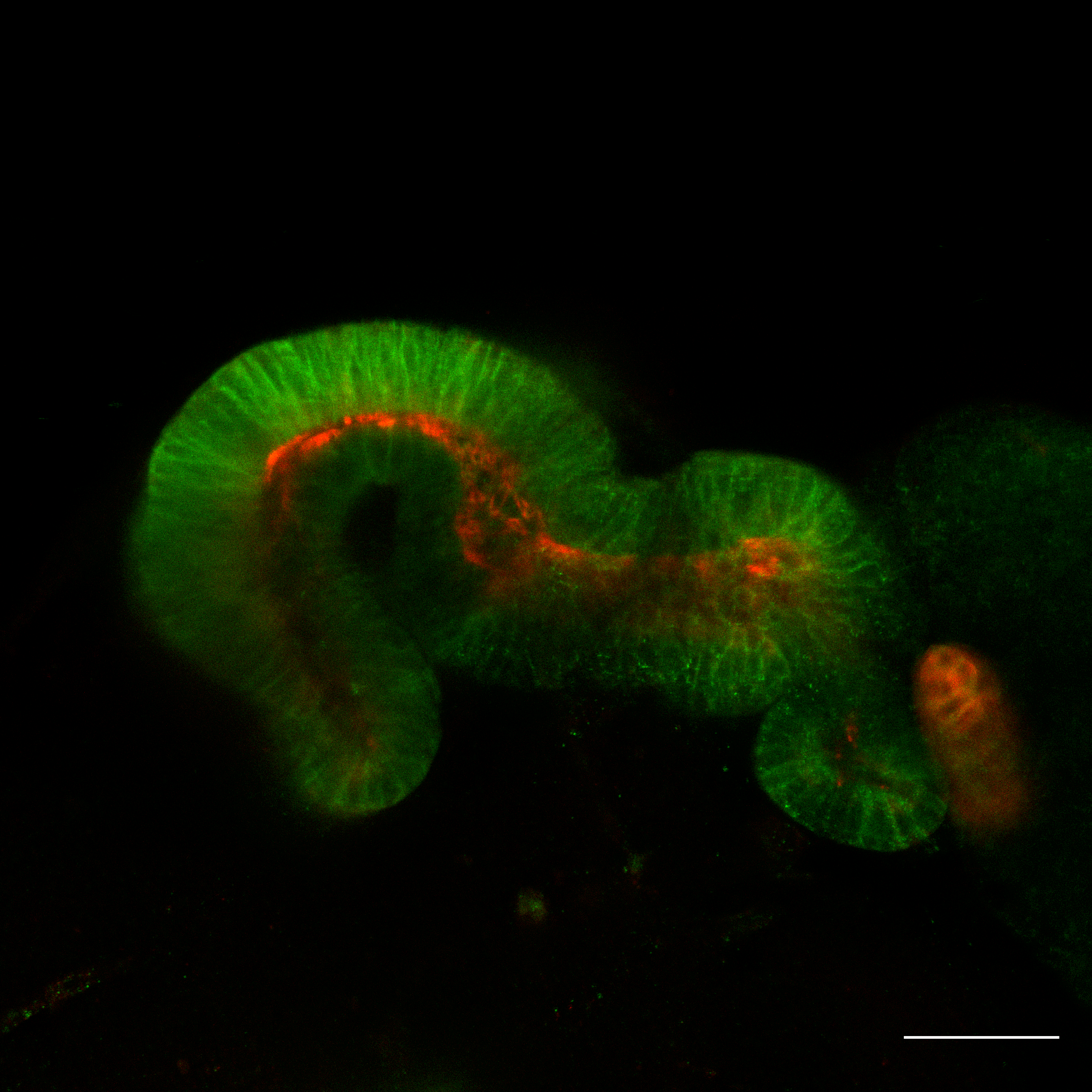

Supplement: Supplementary file 4 — Source data Fig. 2 [file 44318_2025_504_MOESM4_ESM.zip › Figure 2/2D/hFKOs-MUC1-red-ECAD-green-scalebar-20um.tif]

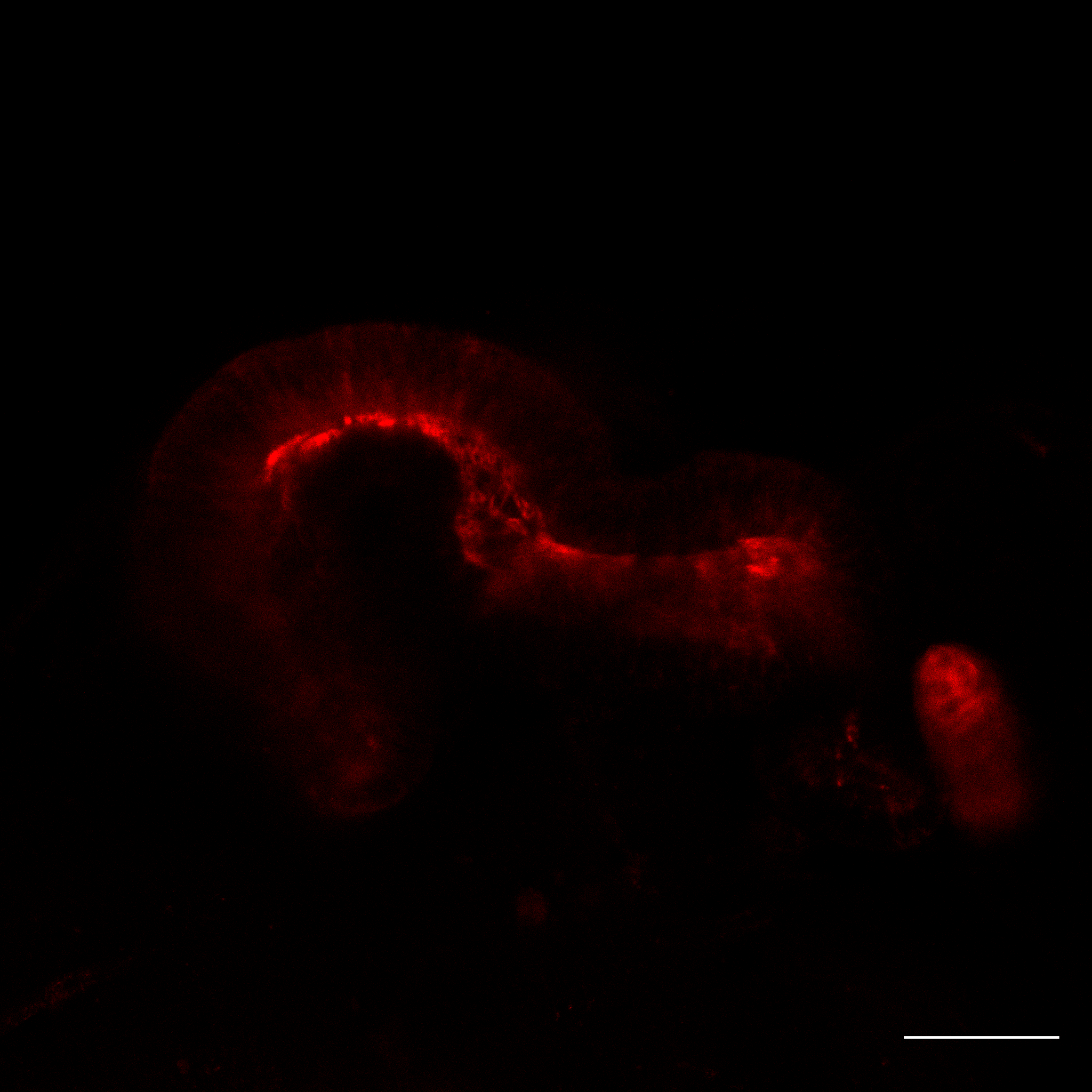

Supplement: Supplementary file 4 — Source data Fig. 2 [file 44318_2025_504_MOESM4_ESM.zip › Figure 2/2D/hFKOs-MUC1-red-scalebar-20um.tif]

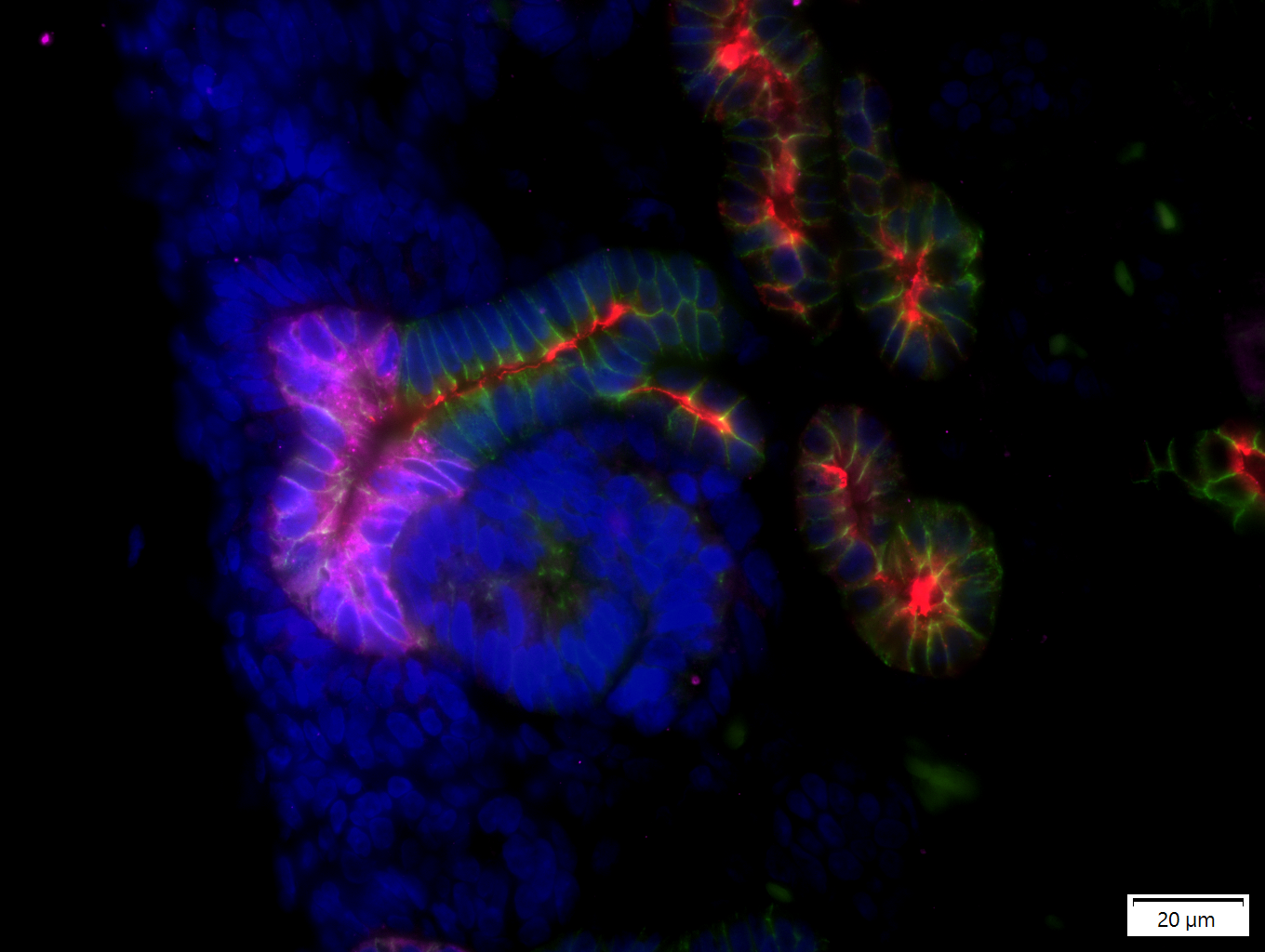

Supplement: Supplementary file 4 — Source data Fig. 2 [file 44318_2025_504_MOESM4_ESM.zip › Figure 2/2D/human-fetal-kidney-slide-CDH6-red-WT1-green-JAG1-purple-DAPI-blue.tif]

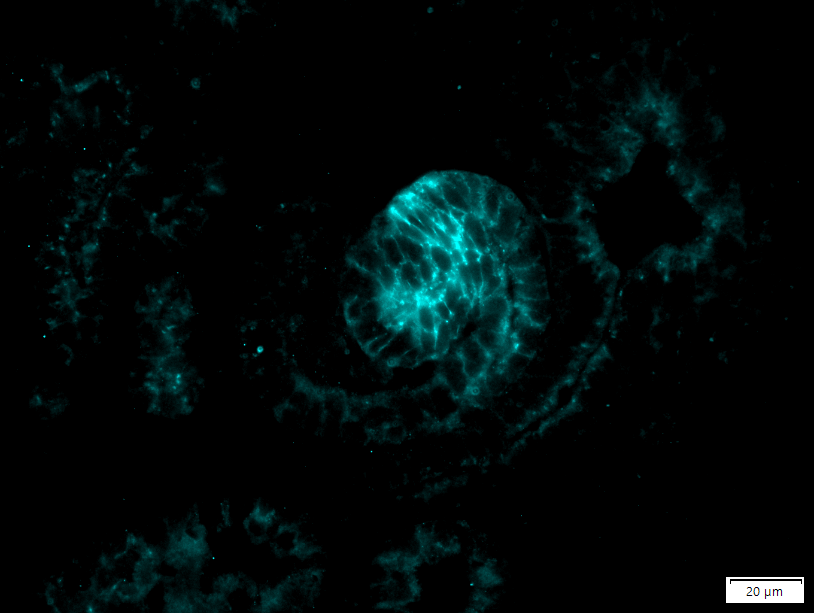

Supplement: Supplementary file 4 — Source data Fig. 2 [file 44318_2025_504_MOESM4_ESM.zip › Figure 2/2E/human-fetal-kidney-slide-JAG1-cyan.tif]

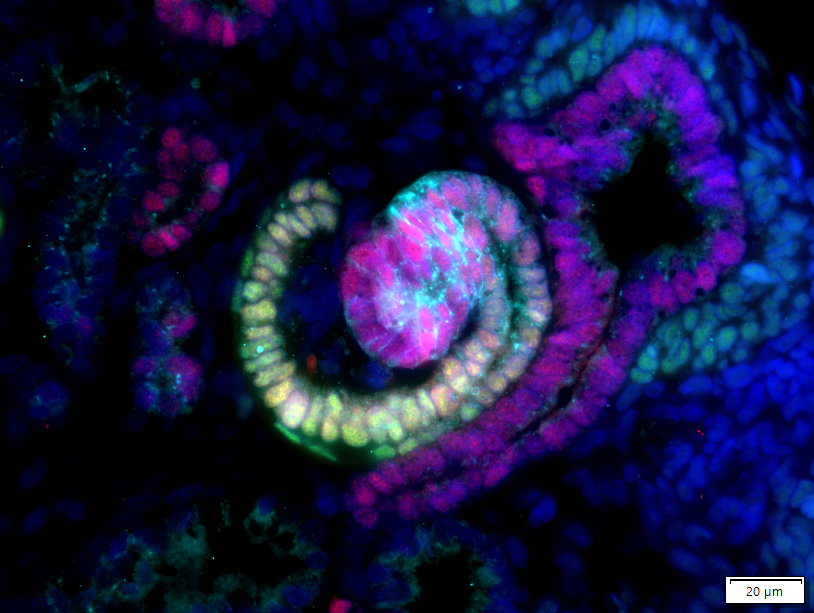

Supplement: Supplementary file 4 — Source data Fig. 2 [file 44318_2025_504_MOESM4_ESM.zip › Figure 2/2E/human-fetal-kidney-slide-LHX1-red-WT1-green-JAG1-cyan-DAPI-blue.tif]

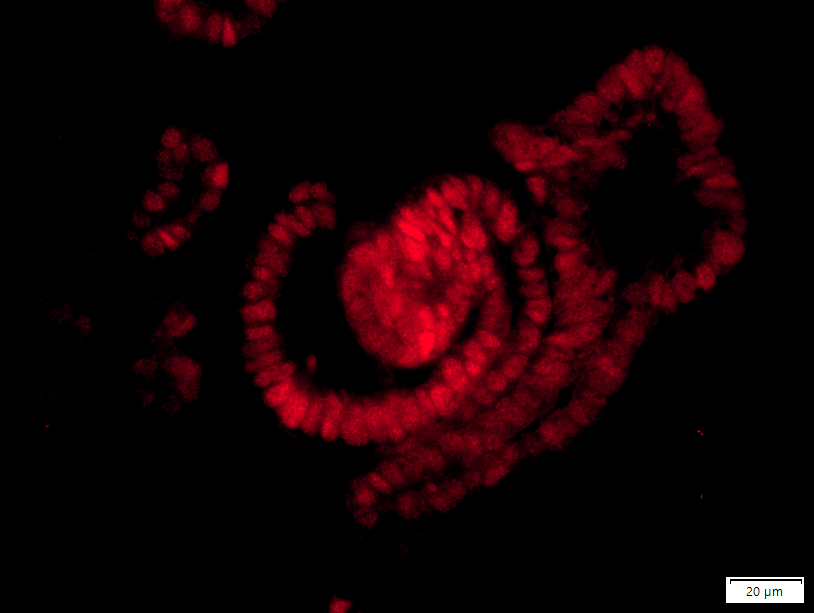

Supplement: Supplementary file 4 — Source data Fig. 2 [file 44318_2025_504_MOESM4_ESM.zip › Figure 2/2E/human-fetal-kidney-slide-LHX1-red.tif]

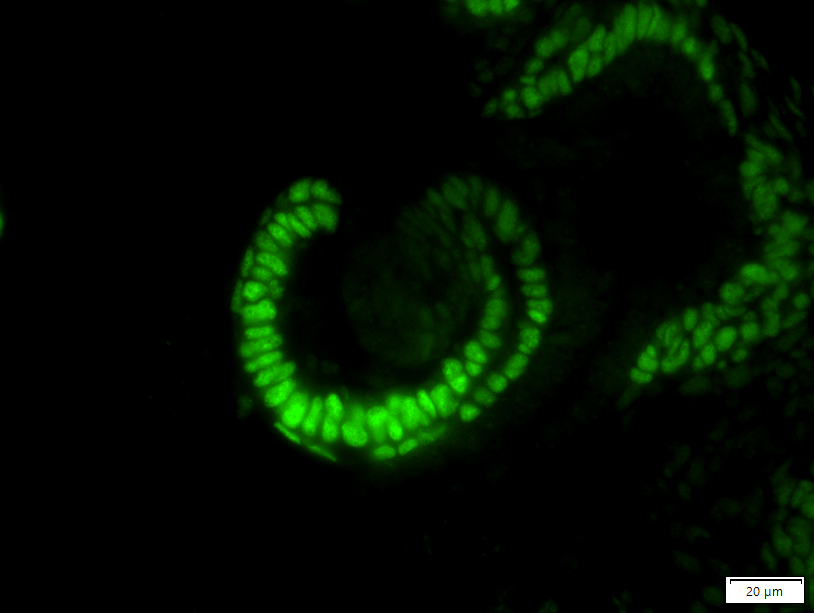

Supplement: Supplementary file 4 — Source data Fig. 2 [file 44318_2025_504_MOESM4_ESM.zip › Figure 2/2E/human-fetal-kidney-slide-WT1-green.tif]

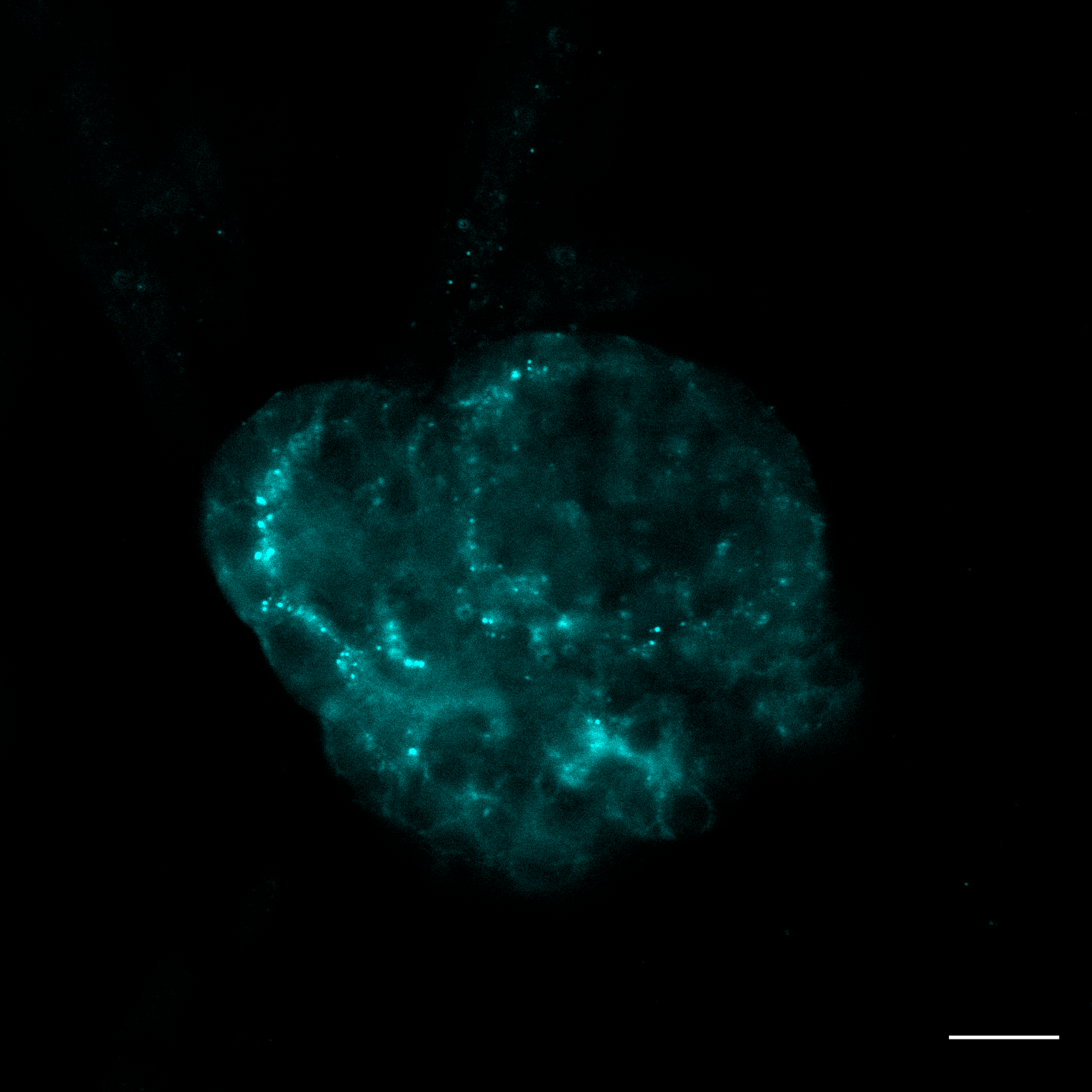

Supplement: Supplementary file 4 — Source data Fig. 2 [file 44318_2025_504_MOESM4_ESM.zip › Figure 2/2F/hFKO-JAG1-cyan-scalebar-20um.tif]

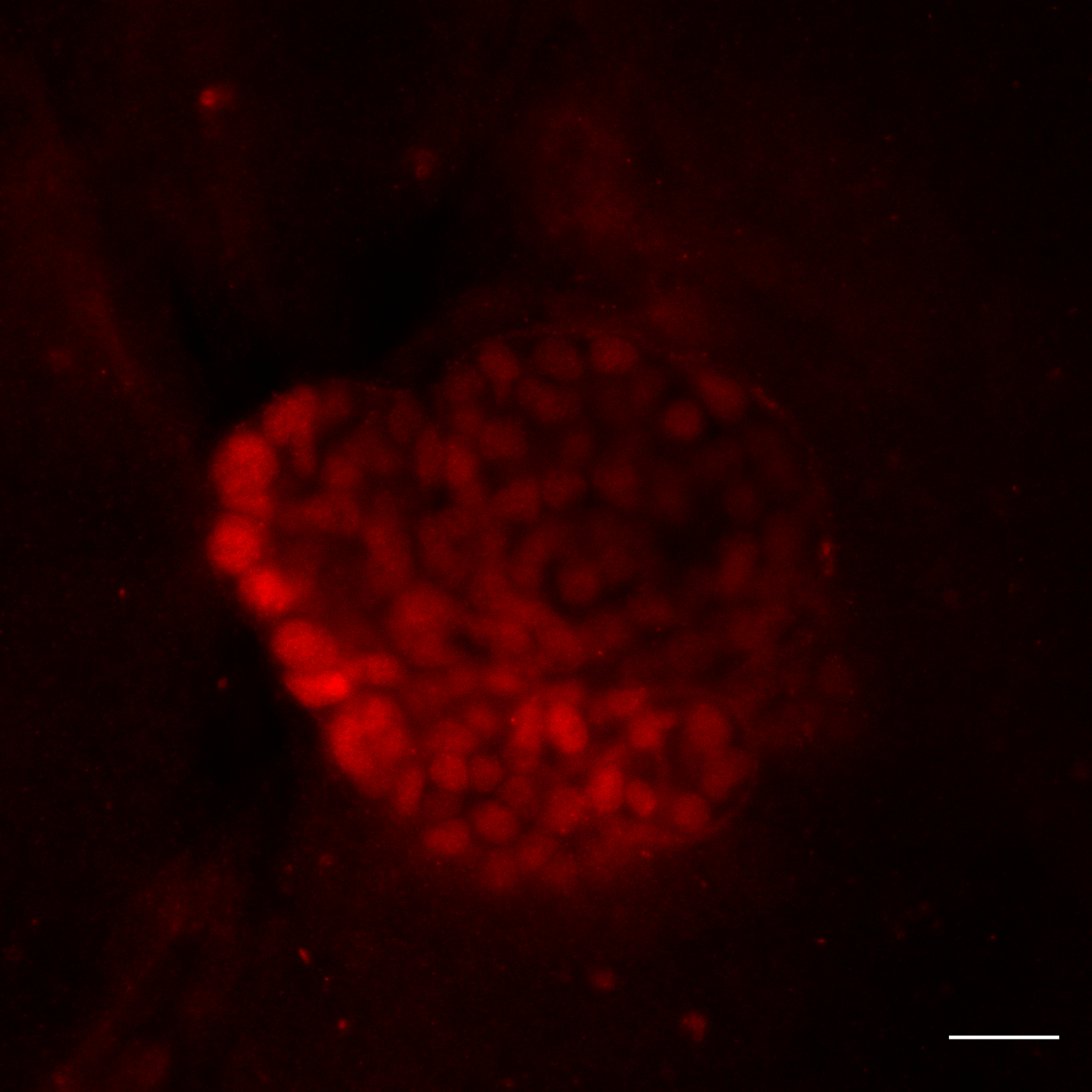

Supplement: Supplementary file 4 — Source data Fig. 2 [file 44318_2025_504_MOESM4_ESM.zip › Figure 2/2F/hFKO-LHX1-red-scalebar-20um.tif]

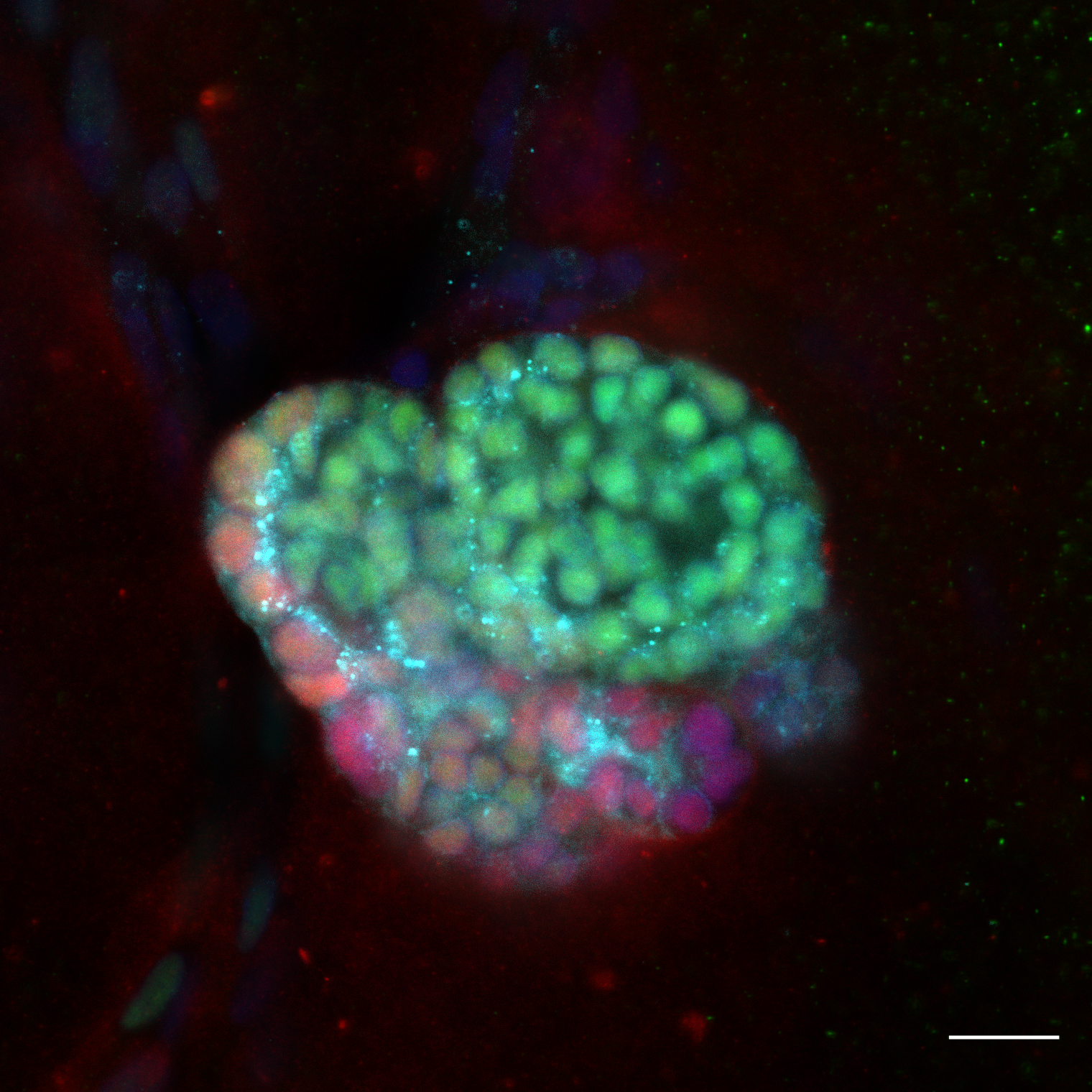

Supplement: Supplementary file 4 — Source data Fig. 2 [file 44318_2025_504_MOESM4_ESM.zip › Figure 2/2F/hFKO-LHX1-red-WT1-green-JAG1-cyan-DAPI-blue-scalebar-20um.tif]

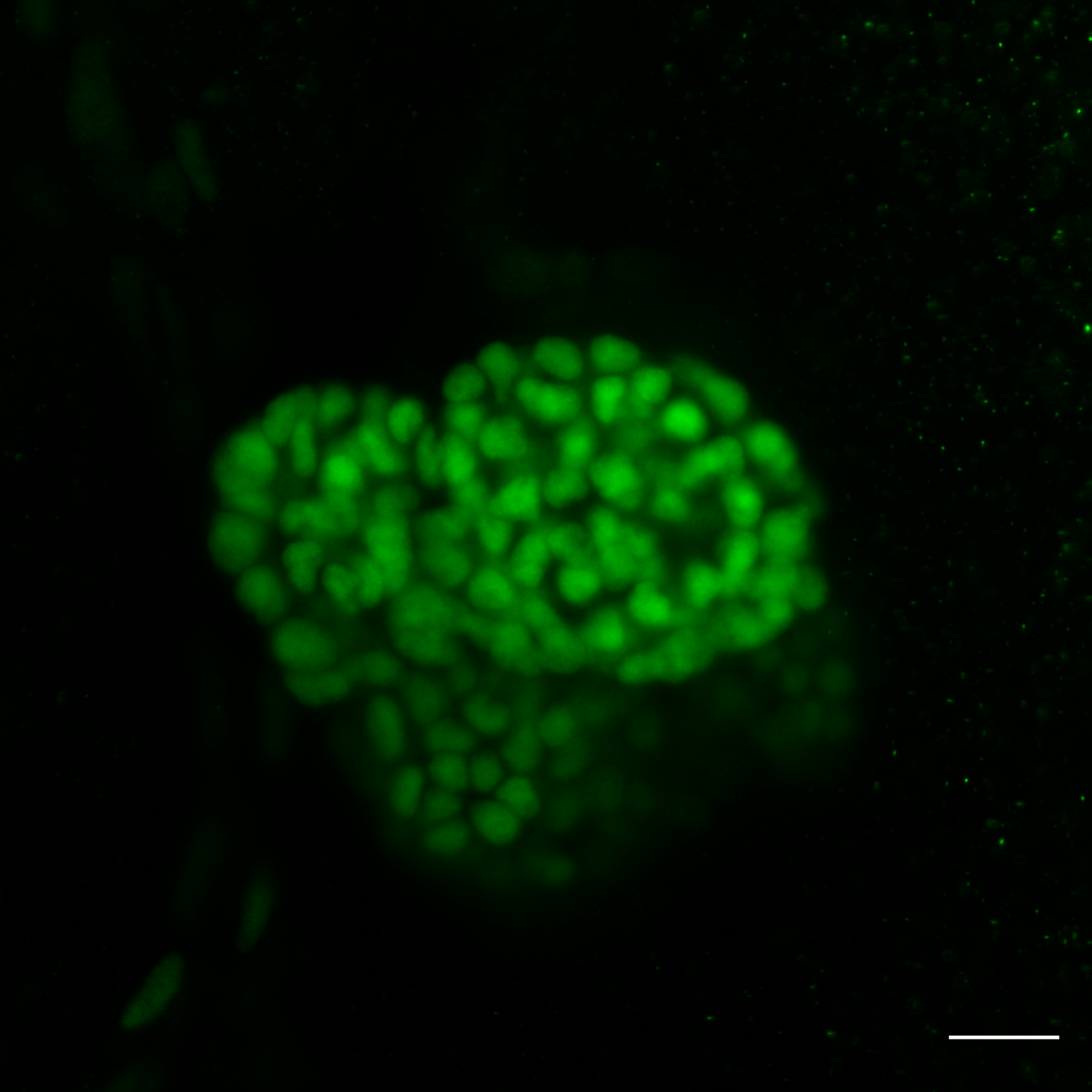

Supplement: Supplementary file 4 — Source data Fig. 2 [file 44318_2025_504_MOESM4_ESM.zip › Figure 2/2F/hFKO-WT1-green-scalebar-20um.tif]

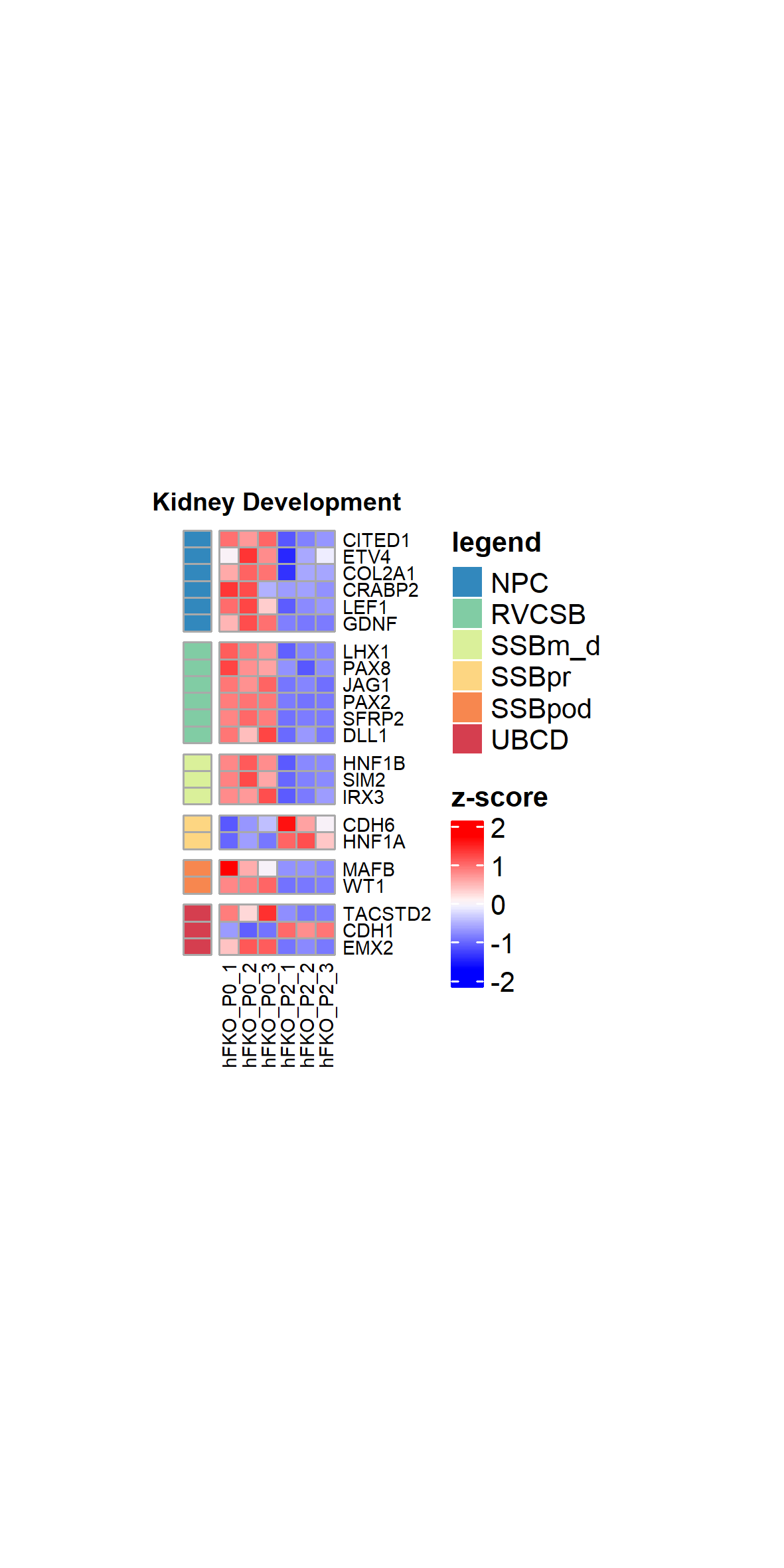

Supplement: Supplementary file 5 — Source data Fig. 3 [file 44318_2025_504_MOESM5_ESM.zip › Figure 3/3A/3A.tiff]

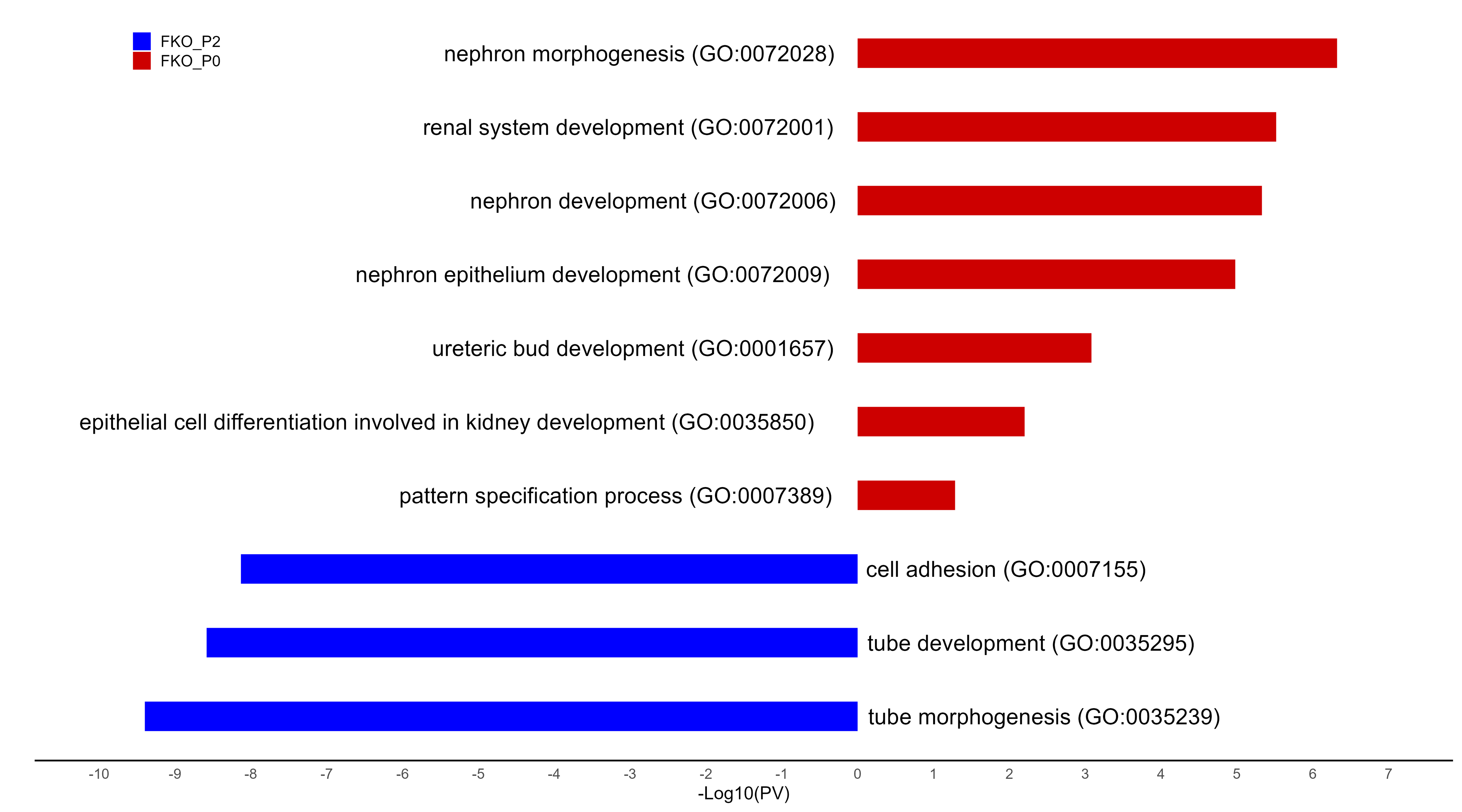

Supplement: Supplementary file 5 — Source data Fig. 3 [file 44318_2025_504_MOESM5_ESM.zip › Figure 3/3B/3B.tiff]

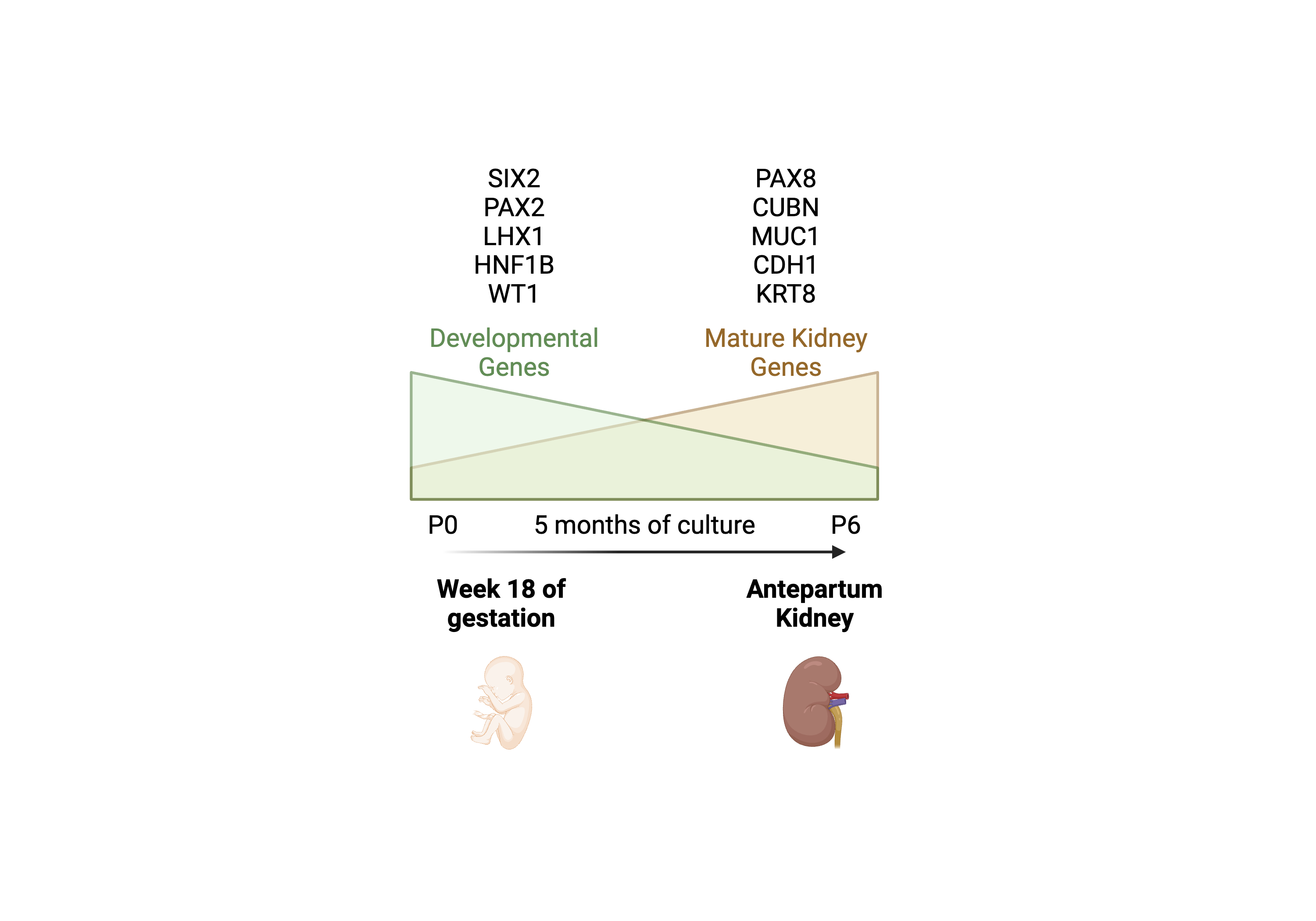

Supplement: Supplementary file 5 — Source data Fig. 3 [file 44318_2025_504_MOESM5_ESM.zip › Figure 3/3C/3C.png]

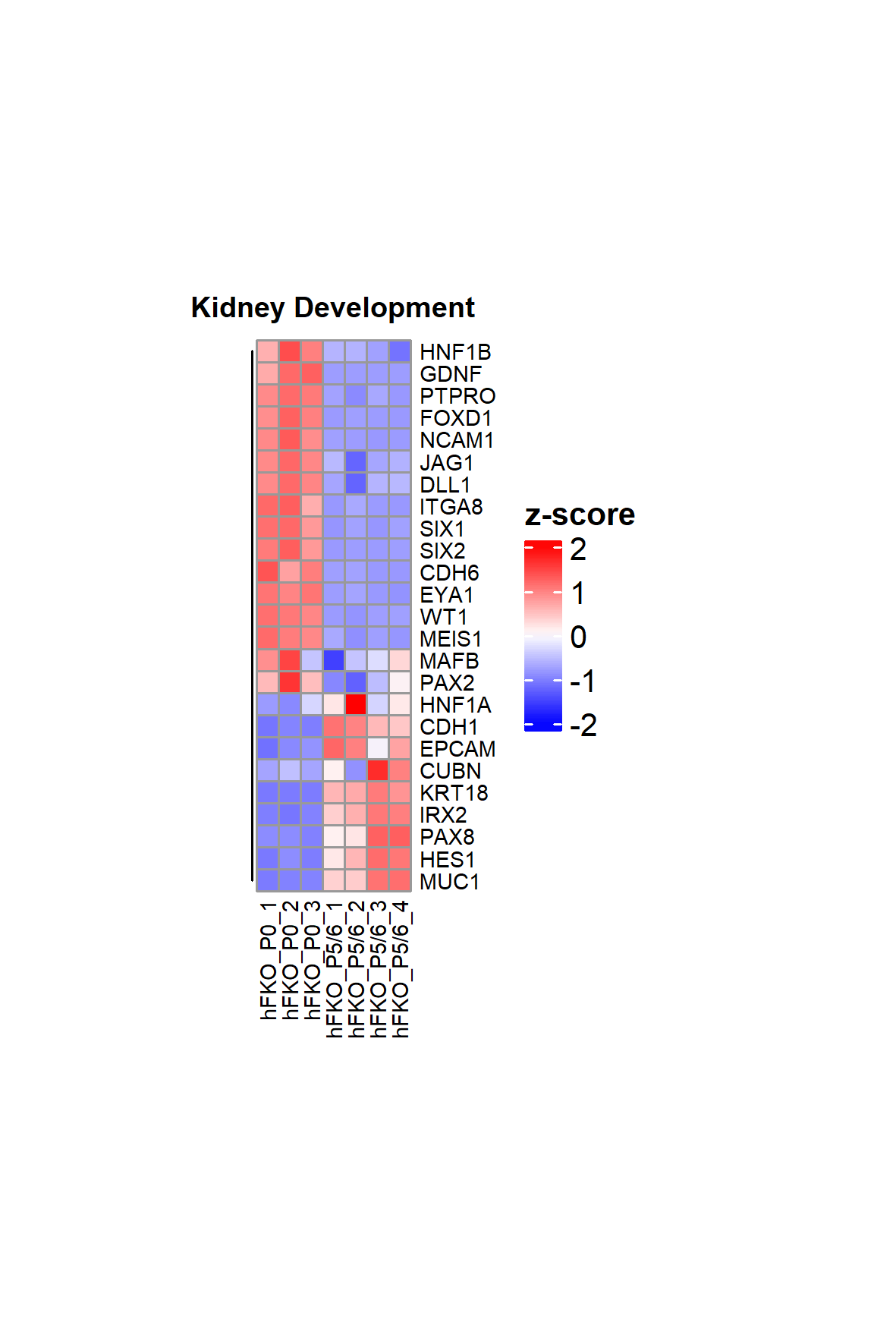

Supplement: Supplementary file 5 — Source data Fig. 3 [file 44318_2025_504_MOESM5_ESM.zip › Figure 3/3D/3D.tiff]

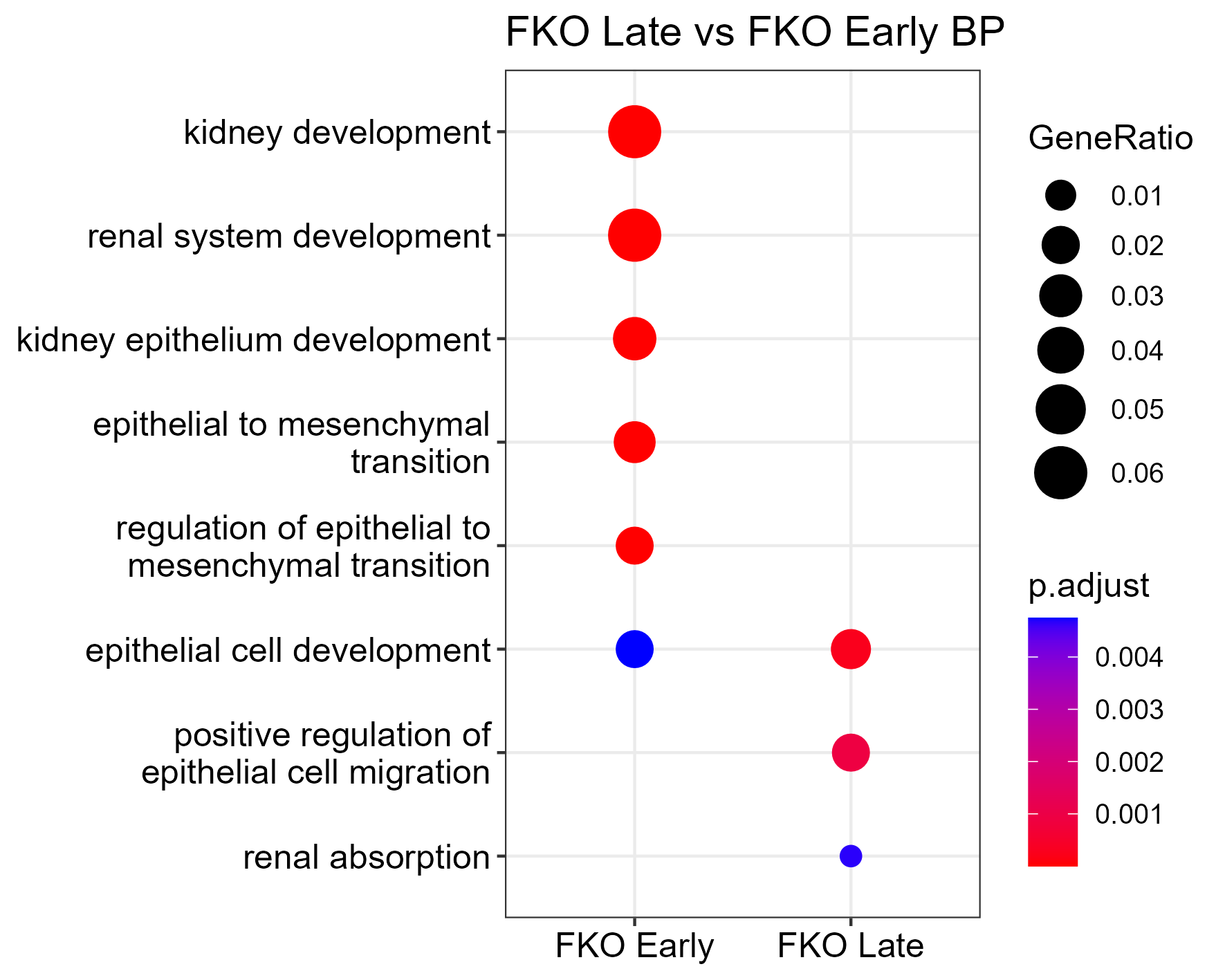

Supplement: Supplementary file 5 — Source data Fig. 3 [file 44318_2025_504_MOESM5_ESM.zip › Figure 3/3E/3E.tiff]

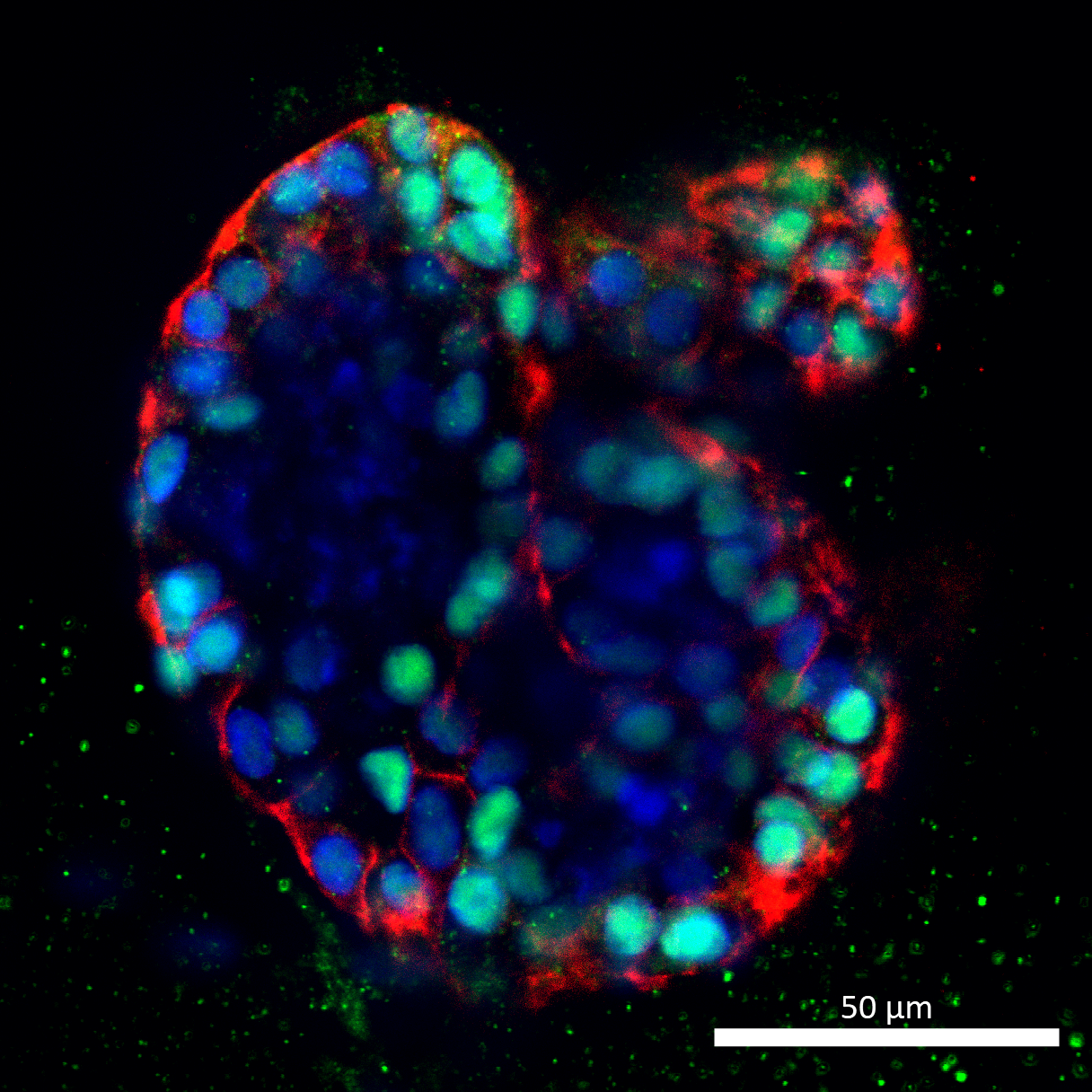

Supplement: Supplementary file 5 — Source data Fig. 3 [file 44318_2025_504_MOESM5_ESM.zip › Figure 3/3F/P2-hFKO-CDH6-red-WT1-green-DAPI-blue.tif]

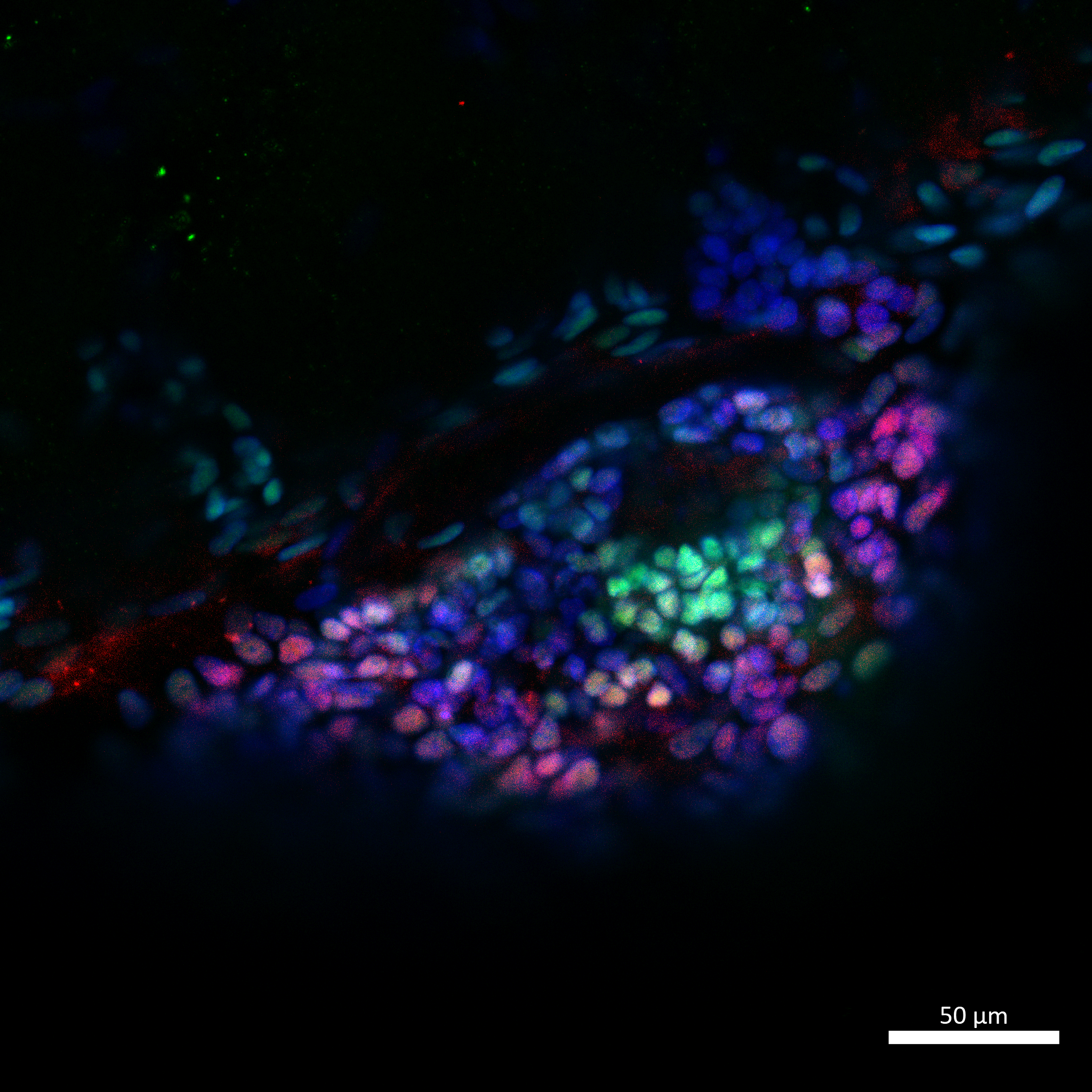

Supplement: Supplementary file 5 — Source data Fig. 3 [file 44318_2025_504_MOESM5_ESM.zip › Figure 3/3F/P2-hFKO-LHX1-red-WT1-green-DAPI-blue.tif]

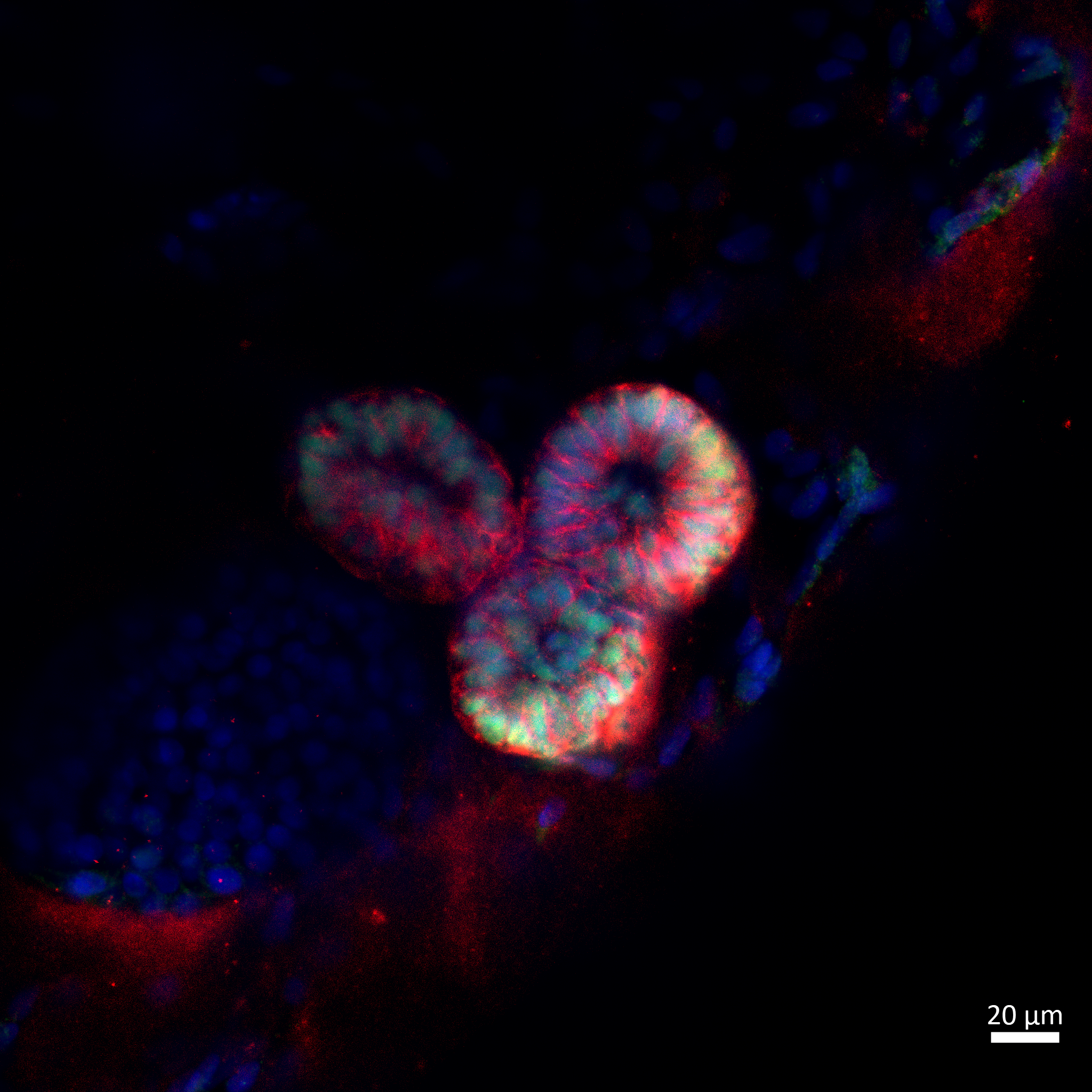

Supplement: Supplementary file 5 — Source data Fig. 3 [file 44318_2025_504_MOESM5_ESM.zip › Figure 3/3F/P2-hFKO-NCAM1-red-PAX2-green-DAPI-blue.tif]

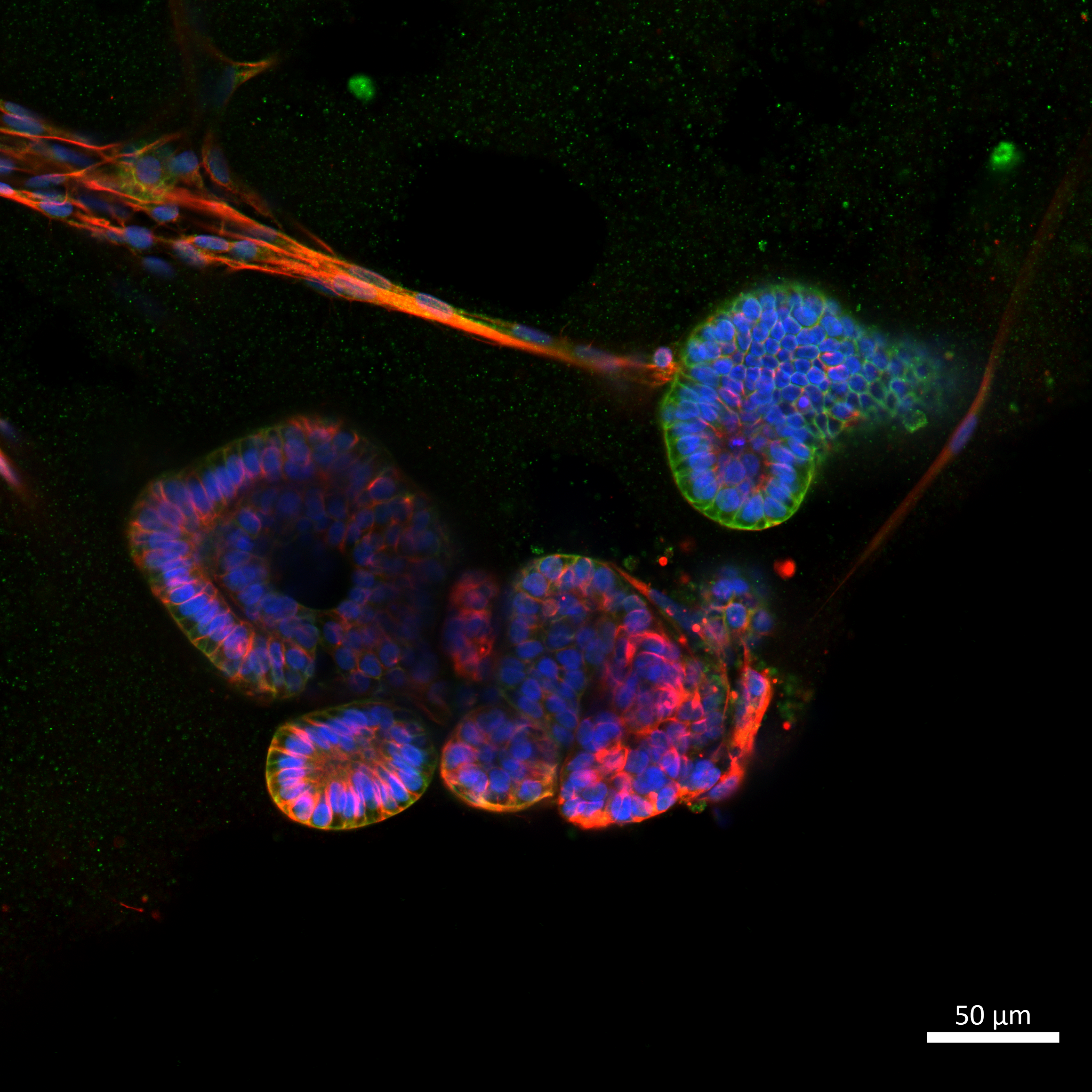

Supplement: Supplementary file 5 — Source data Fig. 3 [file 44318_2025_504_MOESM5_ESM.zip › Figure 3/3F/P2-hFKO-VIMENTIN-red-EPCAM-green-DAPI-blue.tif]

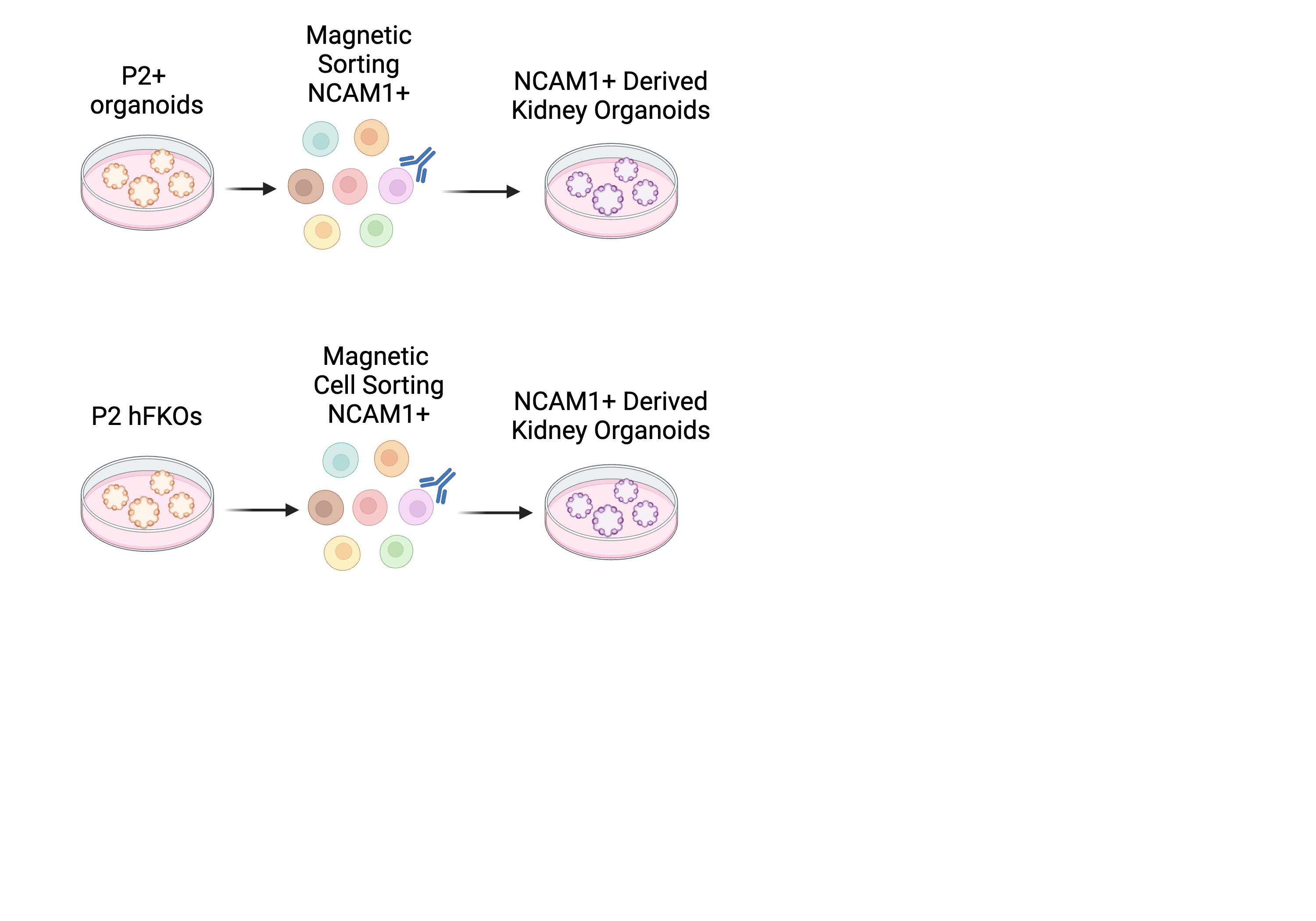

Supplement: Supplementary file 5 — Source data Fig. 3 [file 44318_2025_504_MOESM5_ESM.zip › Figure 3/3G/image105.png]

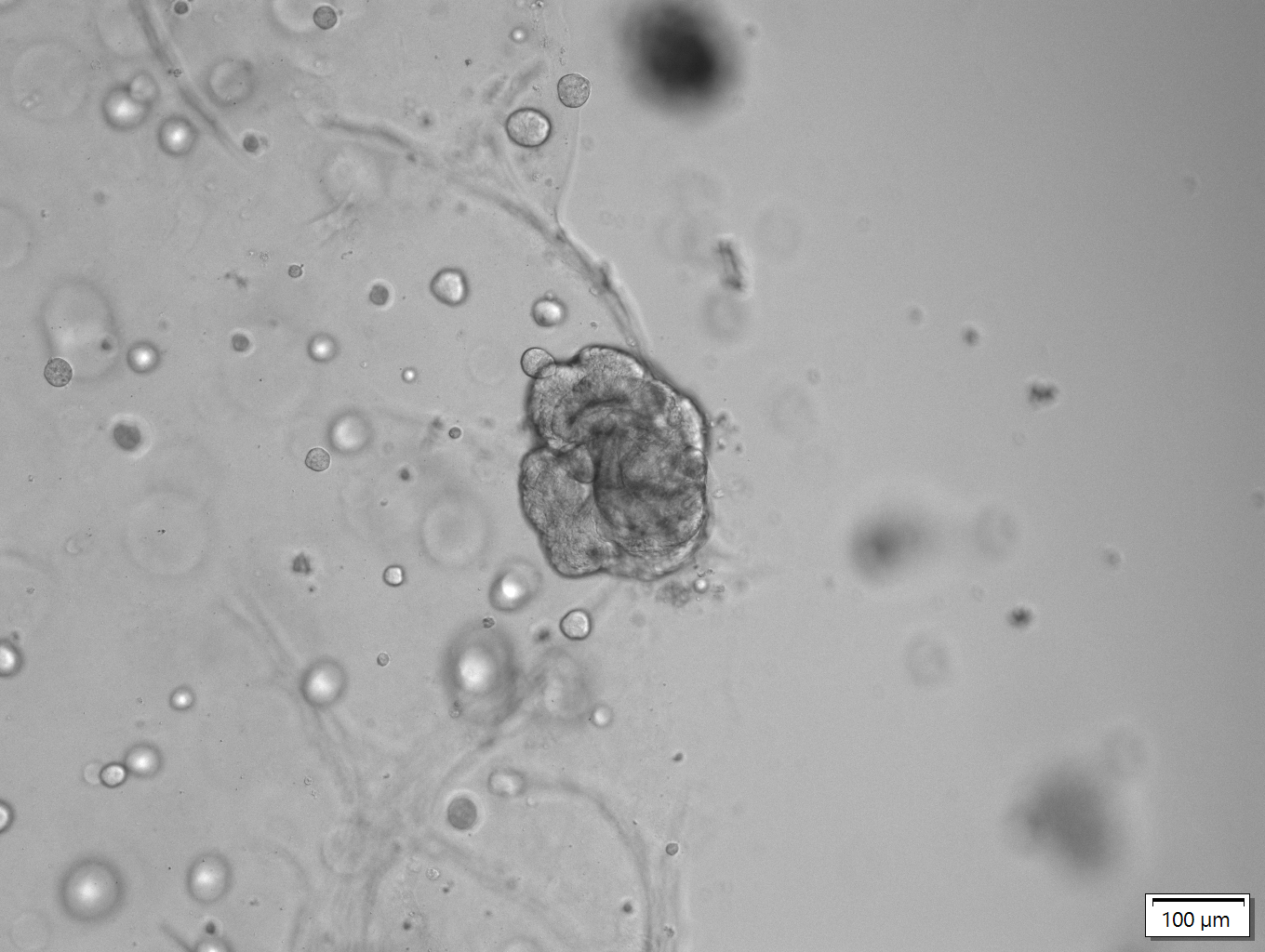

Supplement: Supplementary file 5 — Source data Fig. 3 [file 44318_2025_504_MOESM5_ESM.zip › Figure 3/3H/Brightfield-magnification-hFKOs-derived-from-NCAM1-positive-cells-after-MACS-sorting.tif]

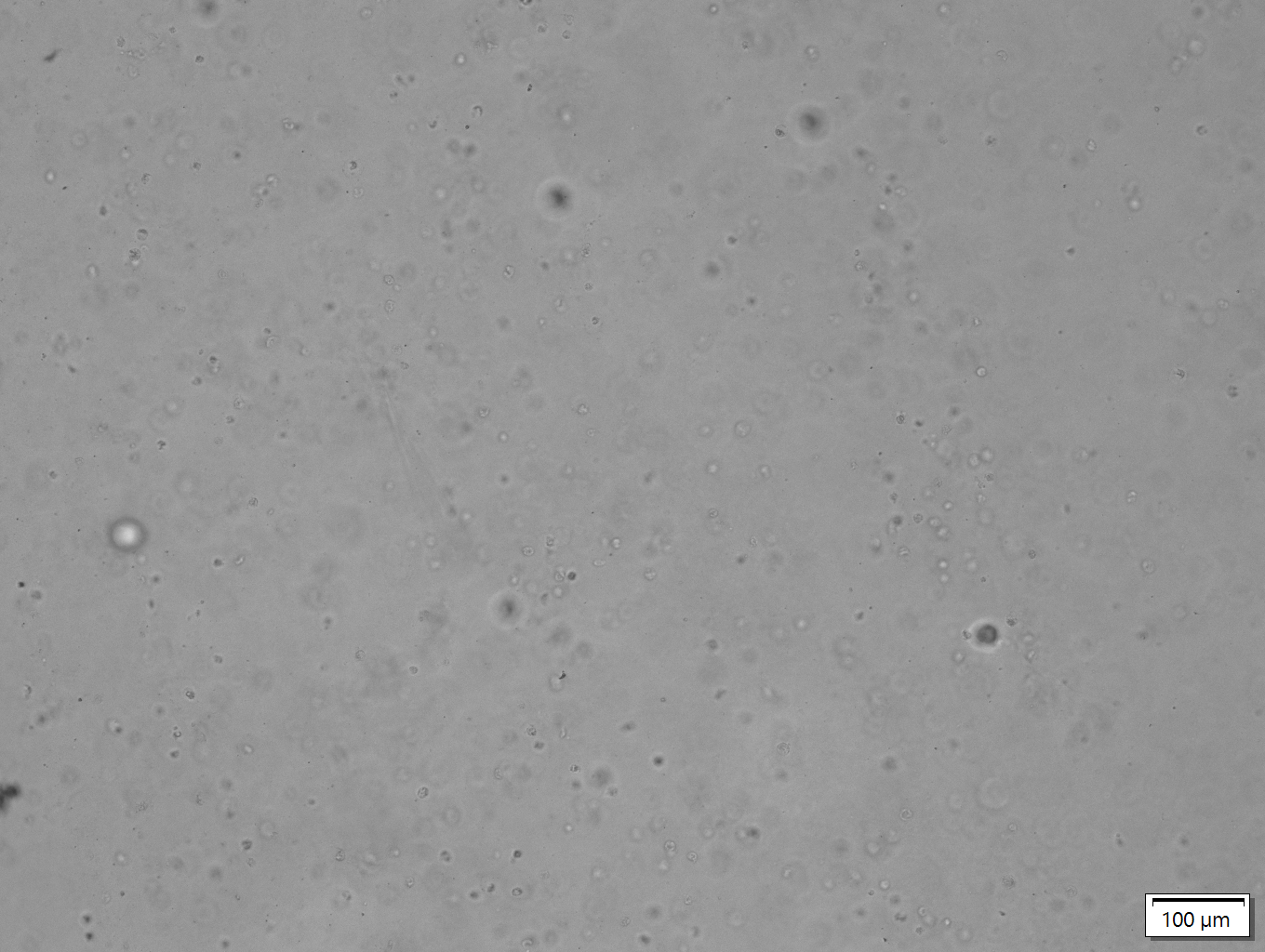

Supplement: Supplementary file 5 — Source data Fig. 3 [file 44318_2025_504_MOESM5_ESM.zip › Figure 3/3H/Brightfield-NCAM1-negative-cells-after-MACS-sorting.tif]

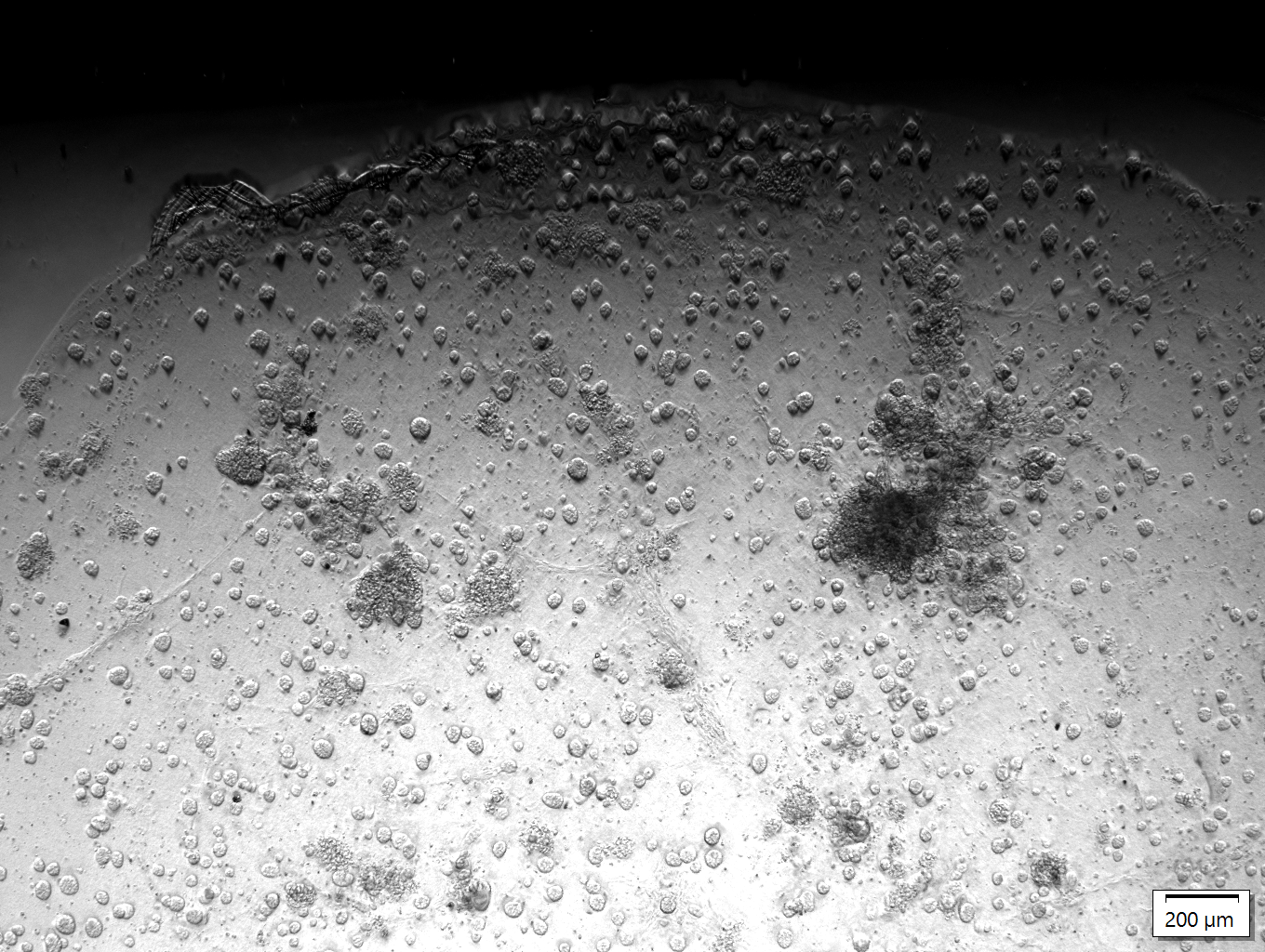

Supplement: Supplementary file 5 — Source data Fig. 3 [file 44318_2025_504_MOESM5_ESM.zip › Figure 3/3H/Widefield-hFKOs-derived-from-NCAM1-positive-cells-after-MACS-sorting.tif]

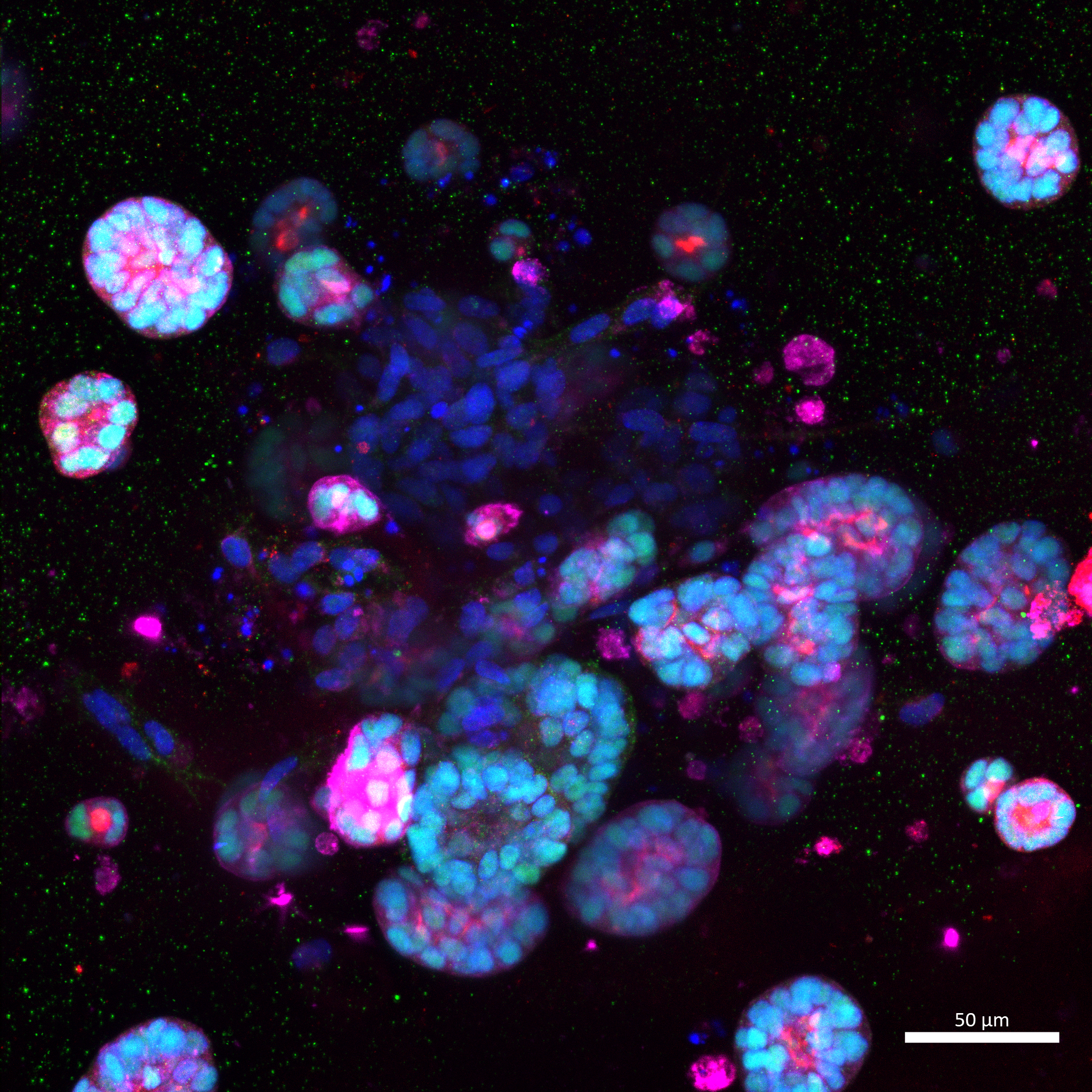

Supplement: Supplementary file 5 — Source data Fig. 3 [file 44318_2025_504_MOESM5_ESM.zip › Figure 3/3I/hFKOs-derived-from-NCAM1-positive-cells-EMA-red-HNF1B-green-LTL-magenta-DAPI-blue.tif]

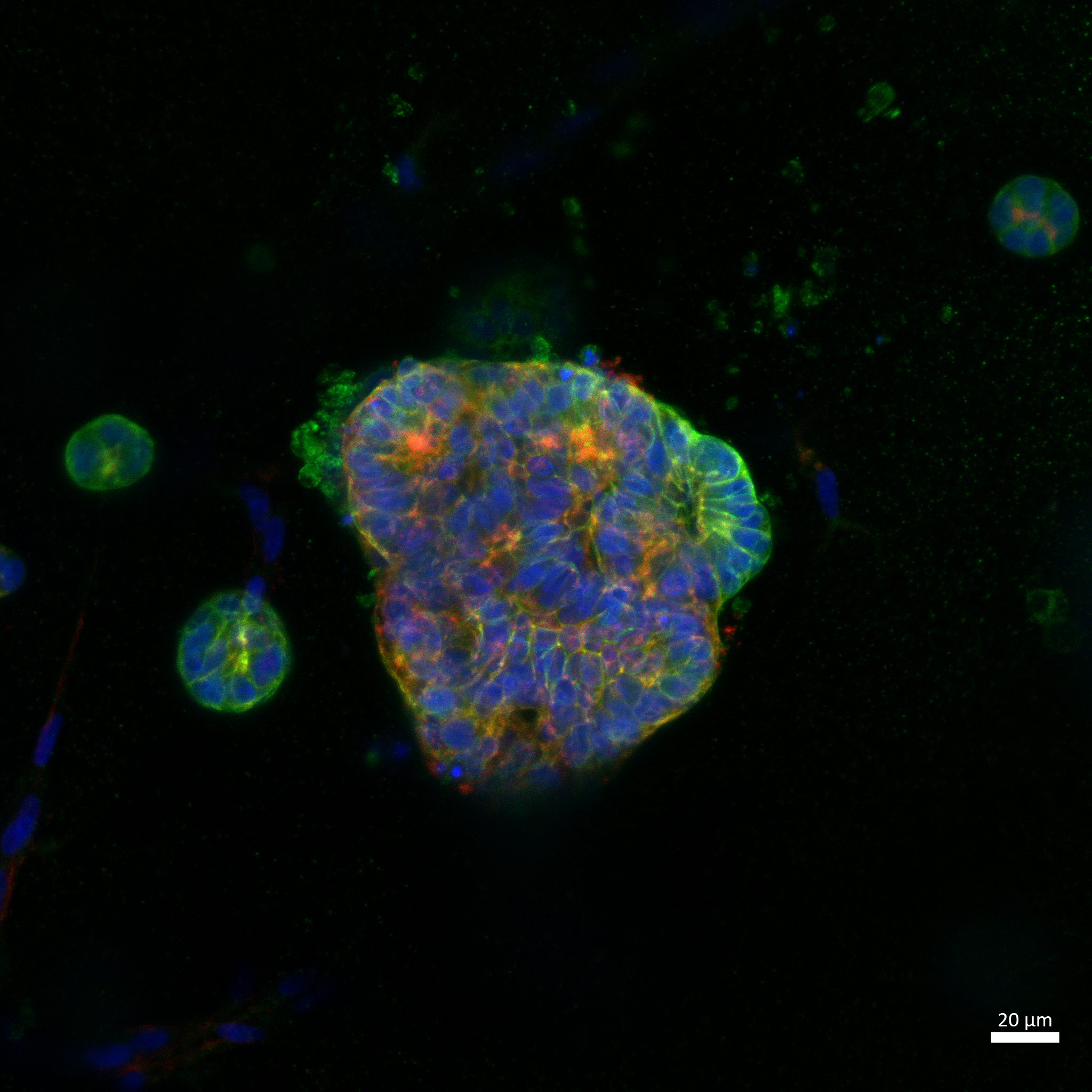

Supplement: Supplementary file 5 — Source data Fig. 3 [file 44318_2025_504_MOESM5_ESM.zip › Figure 3/3I/hFKOs-derived-from-NCAM1-positive-cells-NCAM1-red-EPCAM-green-DAPI-blue.tif]

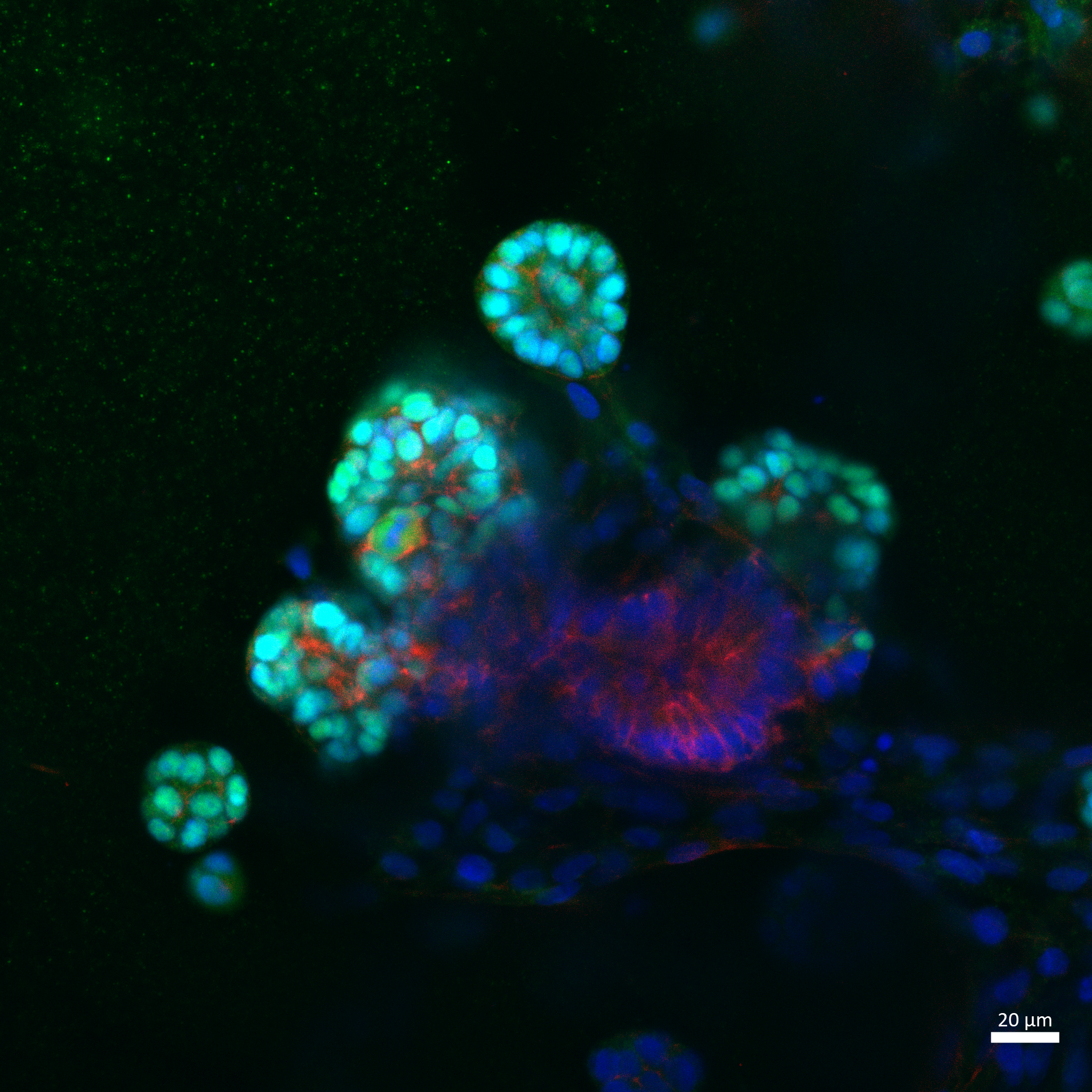

Supplement: Supplementary file 5 — Source data Fig. 3 [file 44318_2025_504_MOESM5_ESM.zip › Figure 3/3I/hFKOs-derived-from-NCAM1-positive-cells-NCAM1-red-PAX2-green-DAPI-blue.tif]

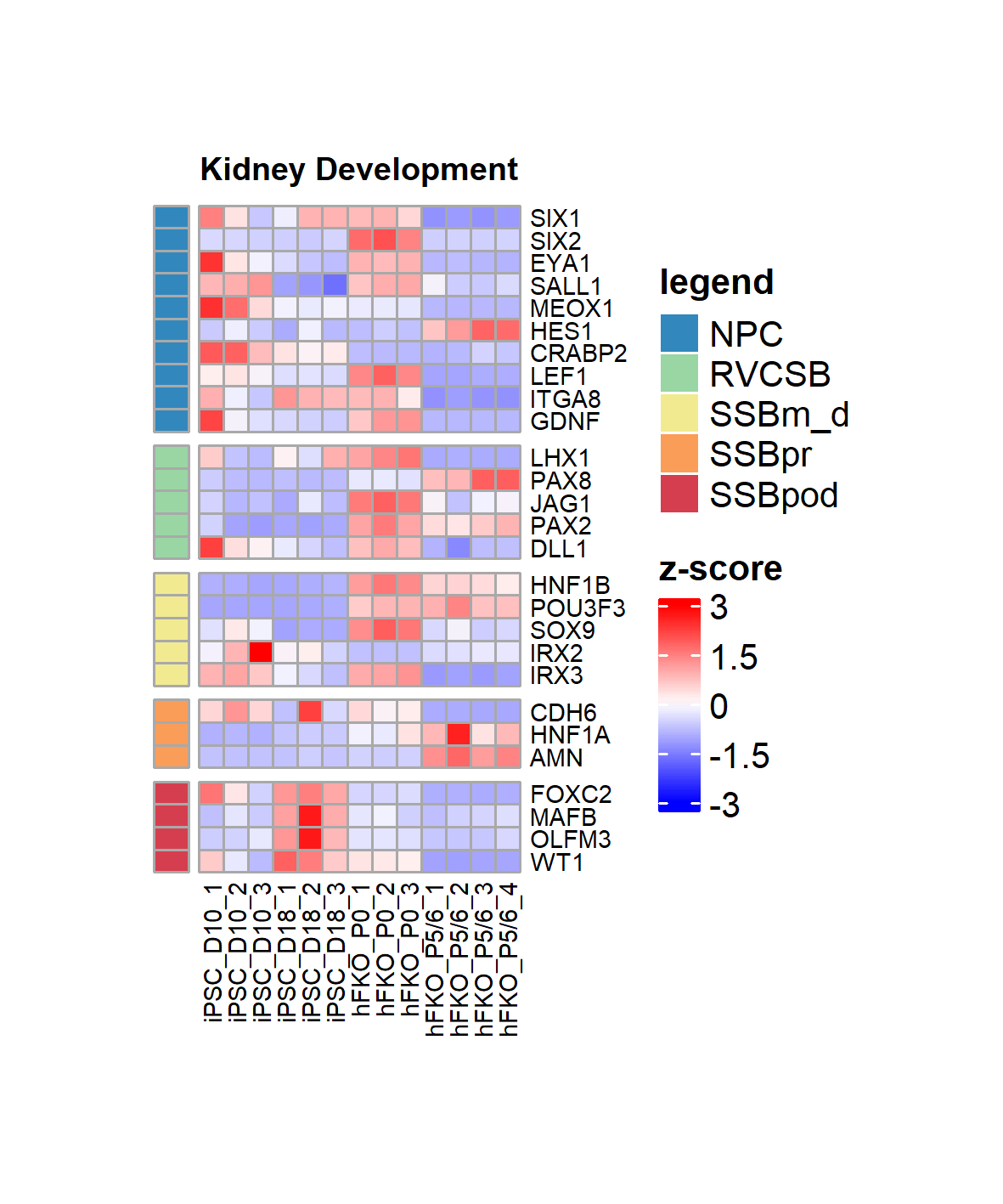

Supplement: Supplementary file 5 — Source data Fig. 3 [file 44318_2025_504_MOESM5_ESM.zip › Figure 3/3J/3J.tiff]

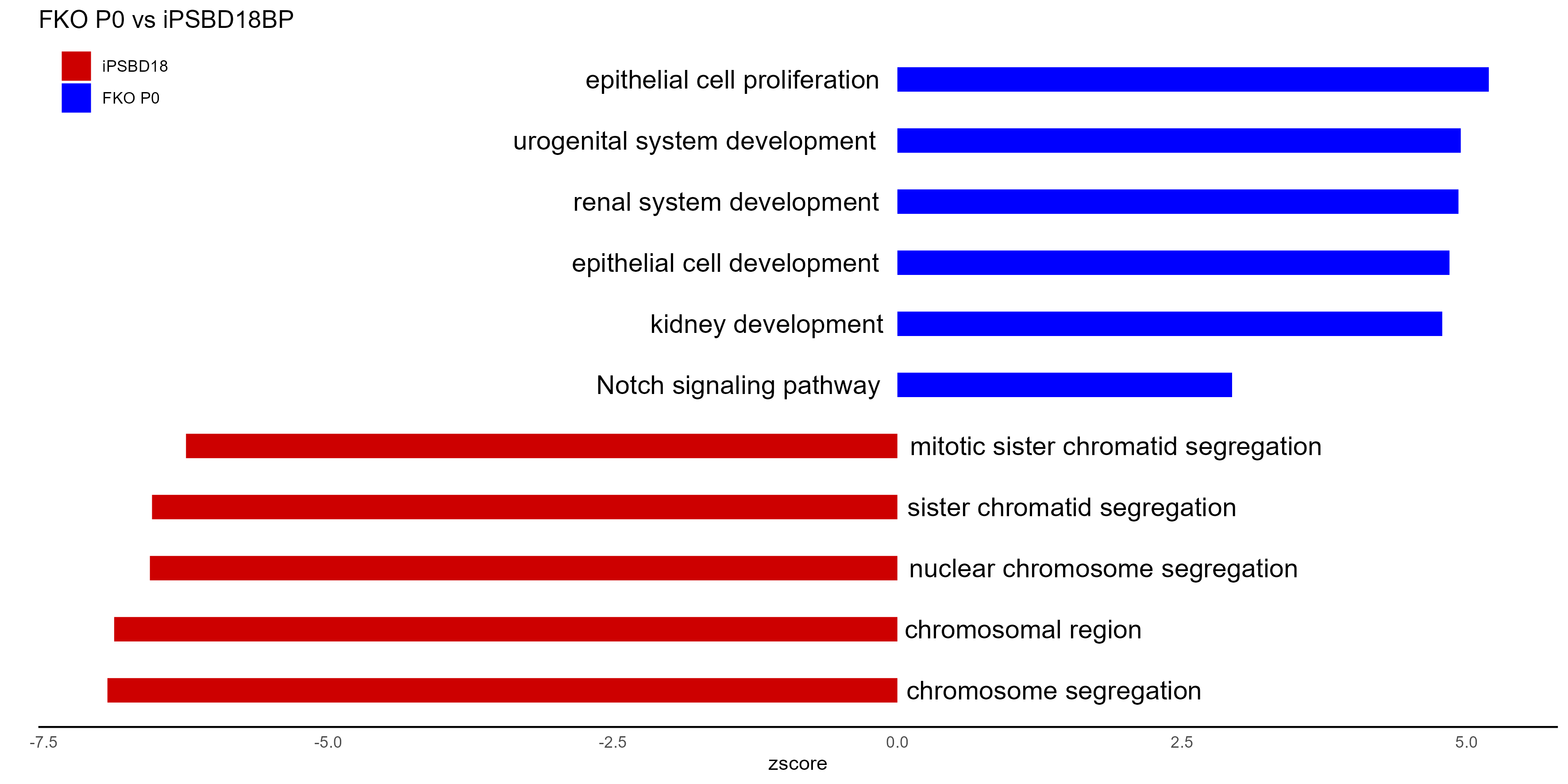

Supplement: Supplementary file 5 — Source data Fig. 3 [file 44318_2025_504_MOESM5_ESM.zip › Figure 3/3K/3K.tiff]

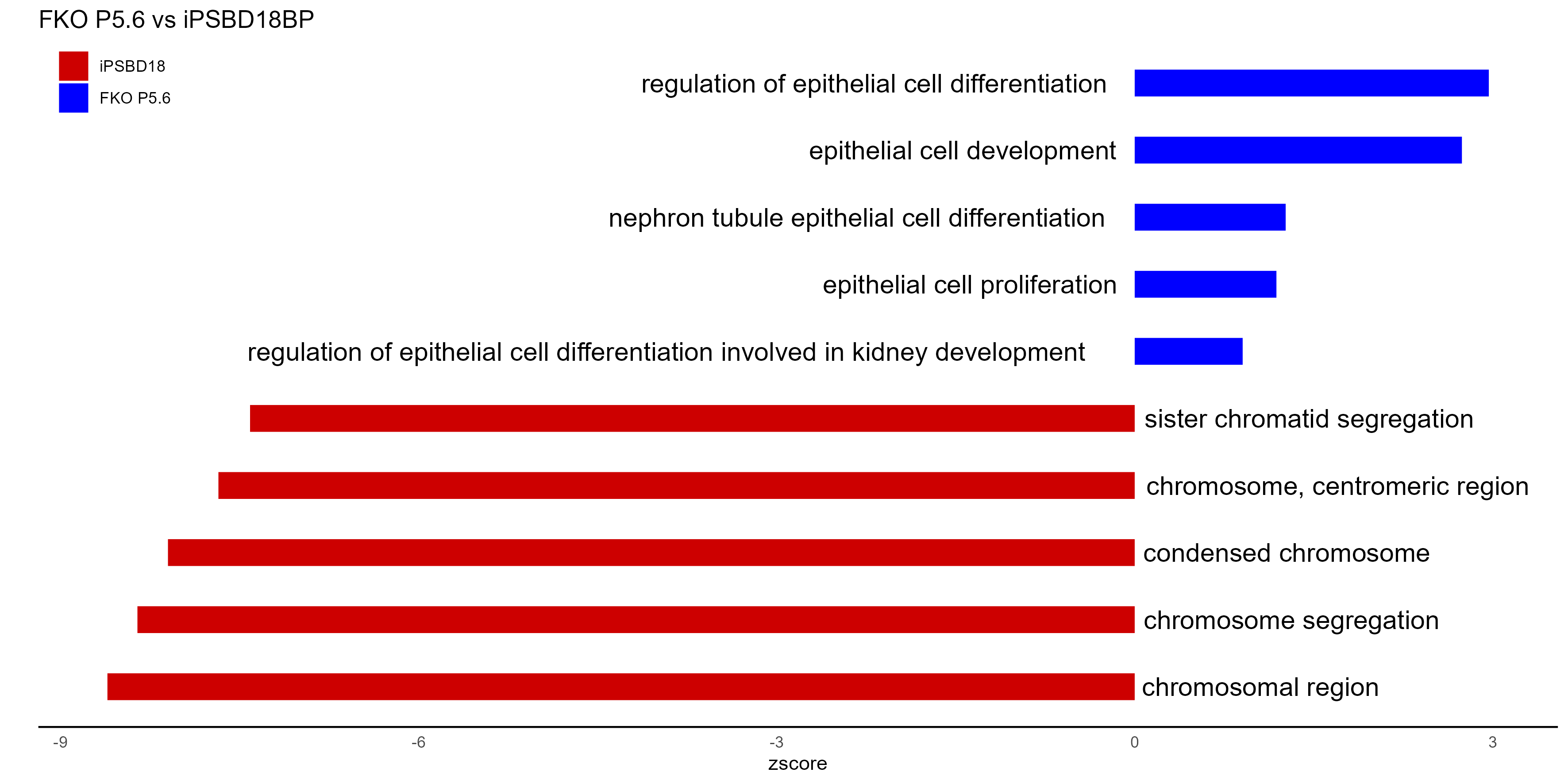

Supplement: Supplementary file 5 — Source data Fig. 3 [file 44318_2025_504_MOESM5_ESM.zip › Figure 3/3L/3L.tiff]

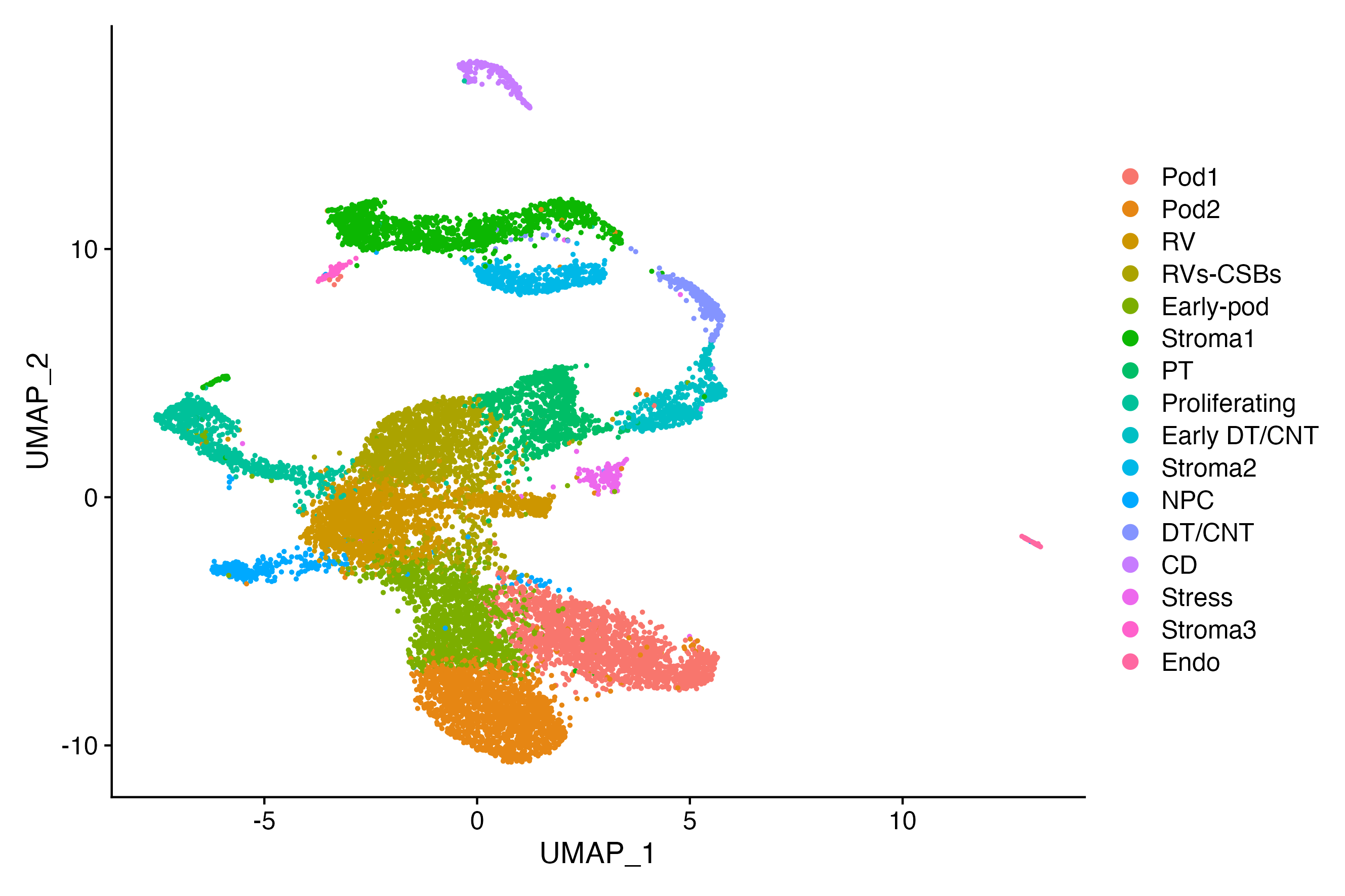

Supplement: Supplementary file 6 — Source data Fig. 4 [file 44318_2025_504_MOESM6_ESM.zip › Figure 4/4A/4A.tiff]

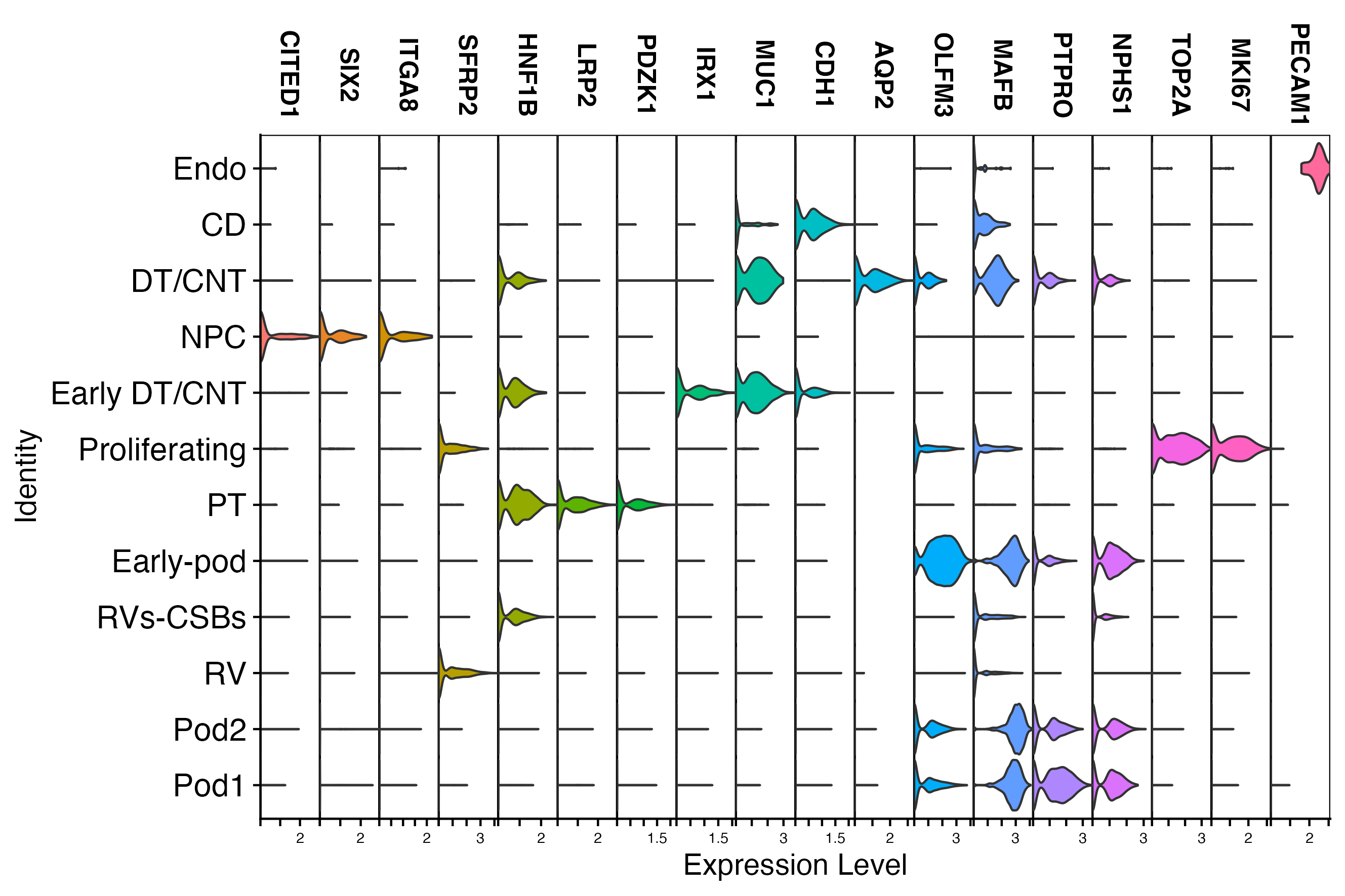

Supplement: Supplementary file 6 — Source data Fig. 4 [file 44318_2025_504_MOESM6_ESM.zip › Figure 4/4B/4B.tiff]

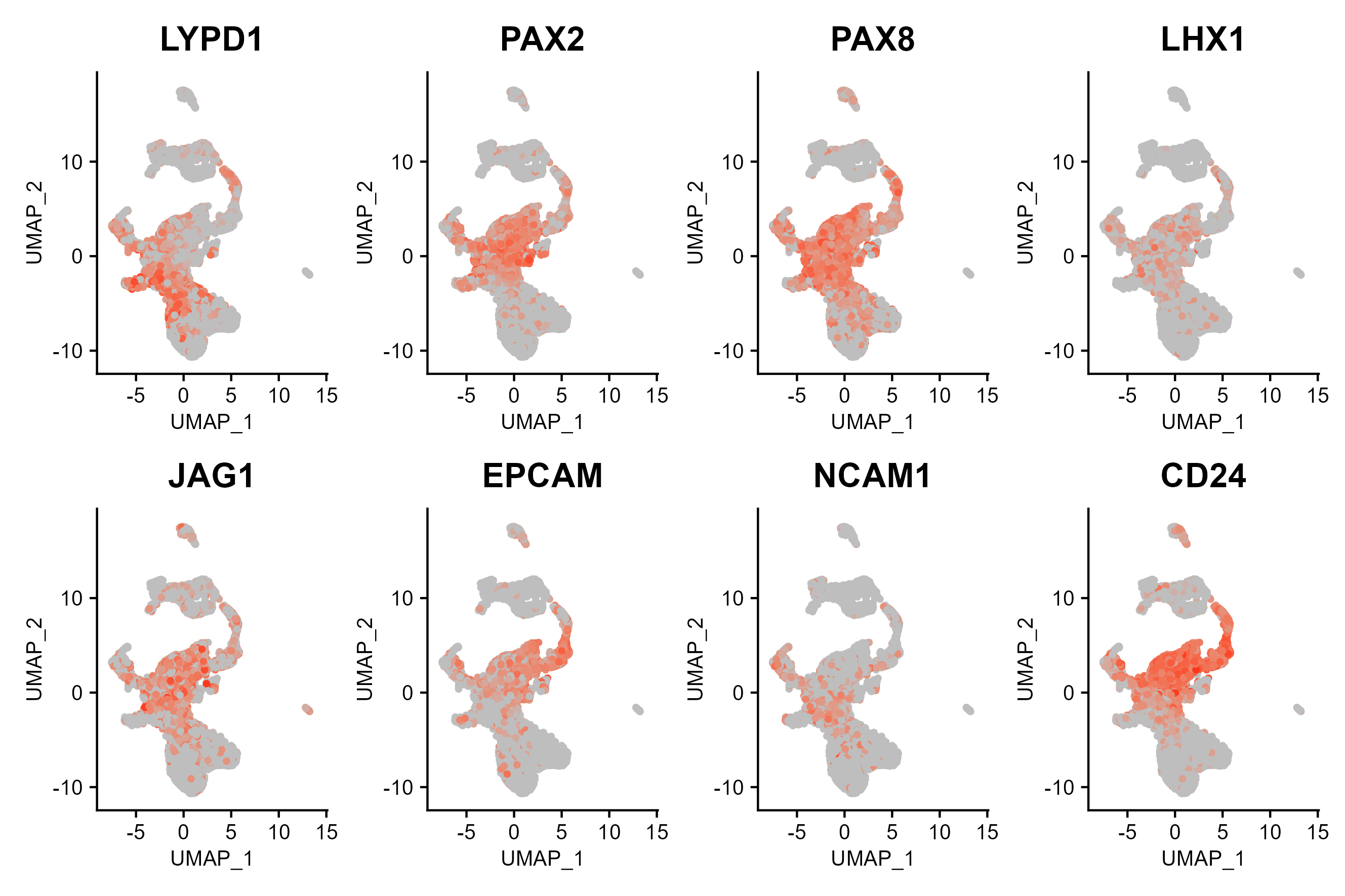

Supplement: Supplementary file 6 — Source data Fig. 4 [file 44318_2025_504_MOESM6_ESM.zip › Figure 4/4C/4C.tiff]

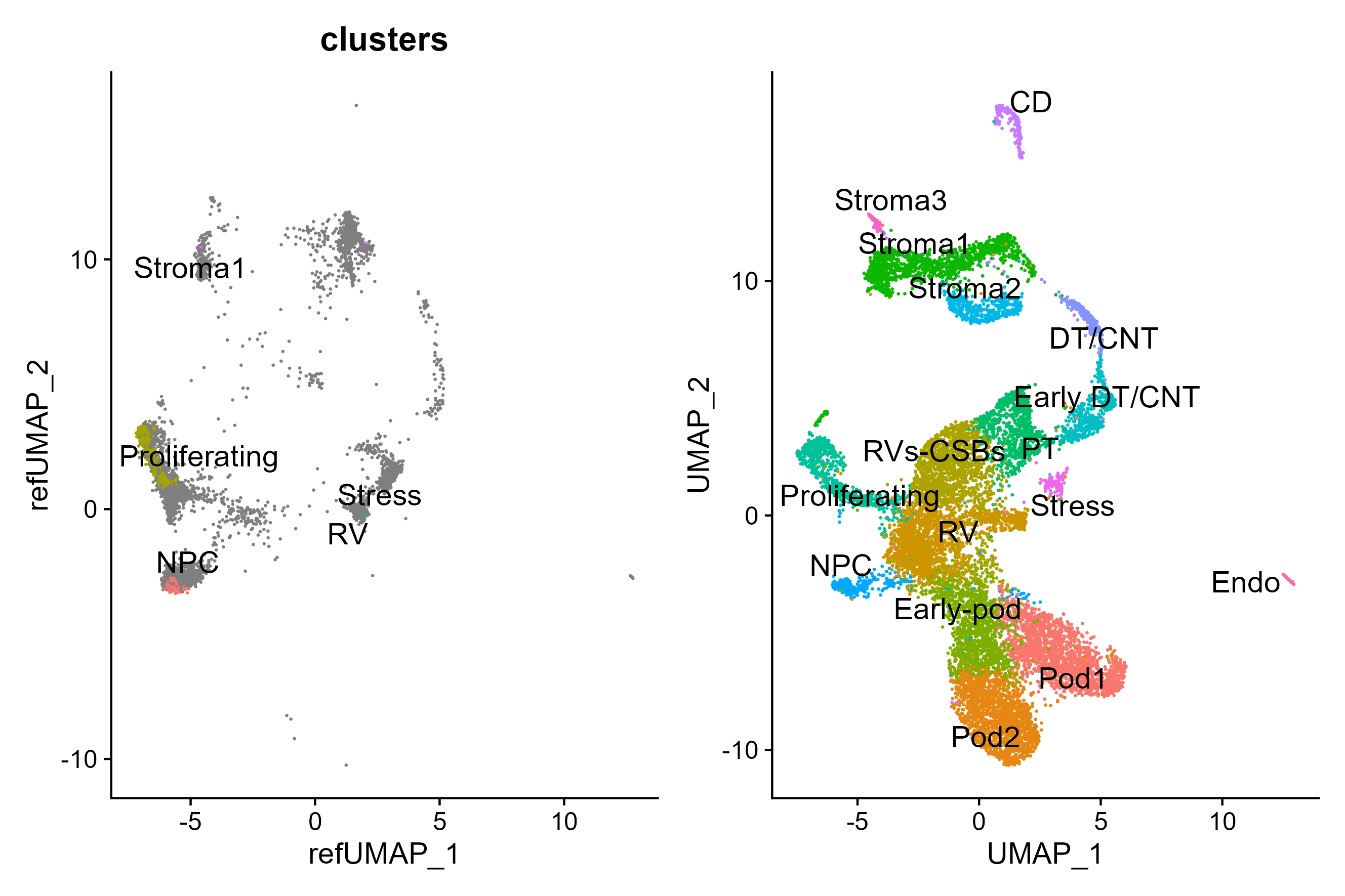

Supplement: Supplementary file 6 — Source data Fig. 4 [file 44318_2025_504_MOESM6_ESM.zip › Figure 4/4D/4D.tiff]

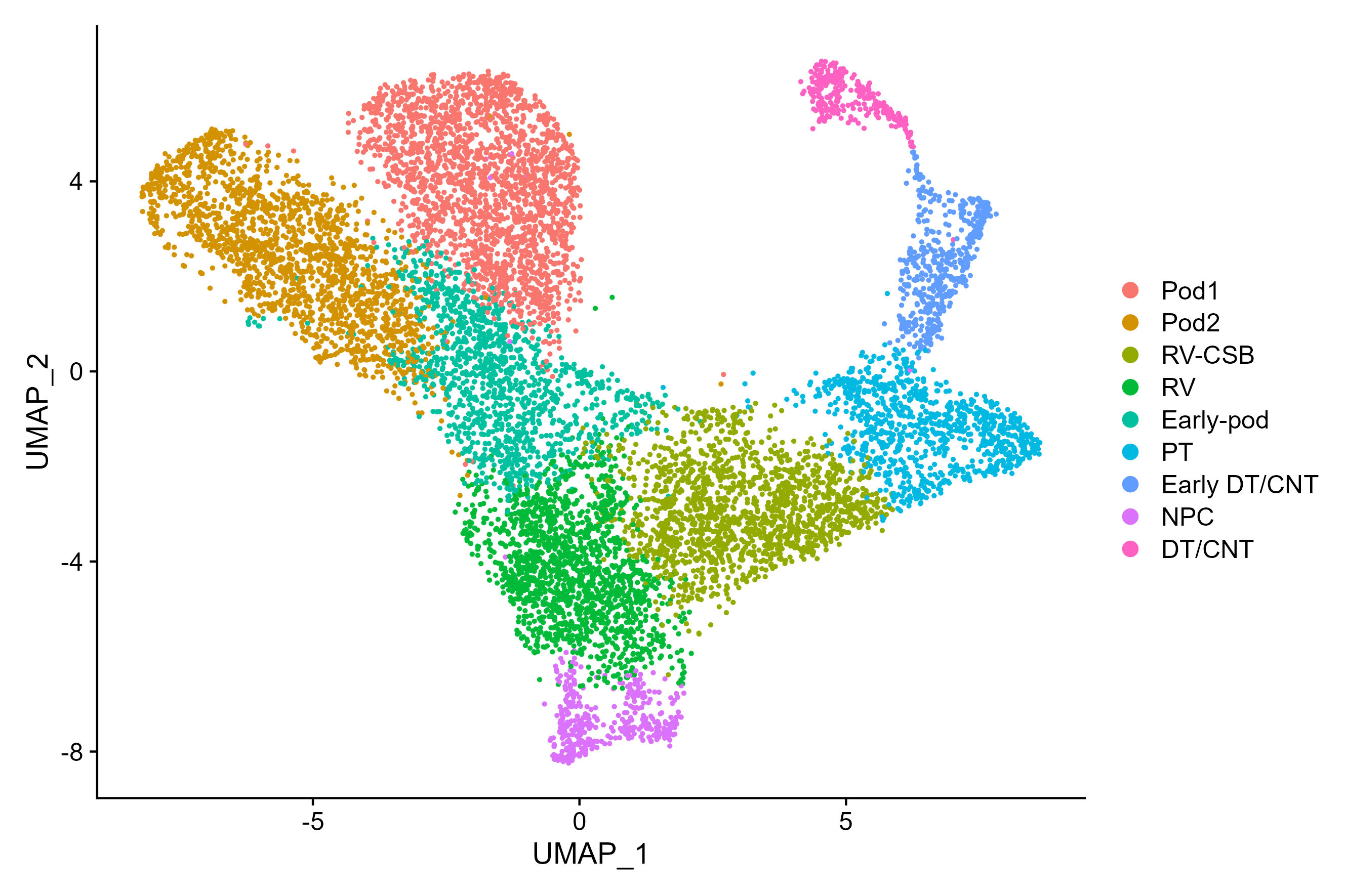

Supplement: Supplementary file 6 — Source data Fig. 4 [file 44318_2025_504_MOESM6_ESM.zip › Figure 4/4E/4E.tiff]

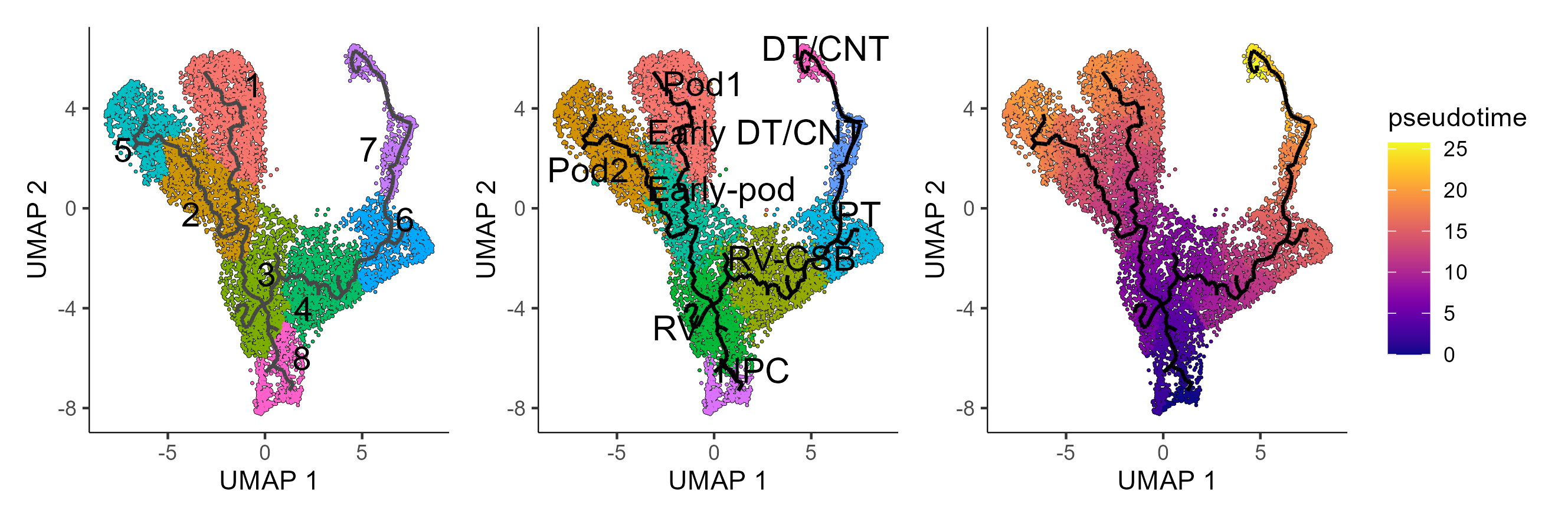

Supplement: Supplementary file 6 — Source data Fig. 4 [file 44318_2025_504_MOESM6_ESM.zip › Figure 4/4F/4F.tiff]

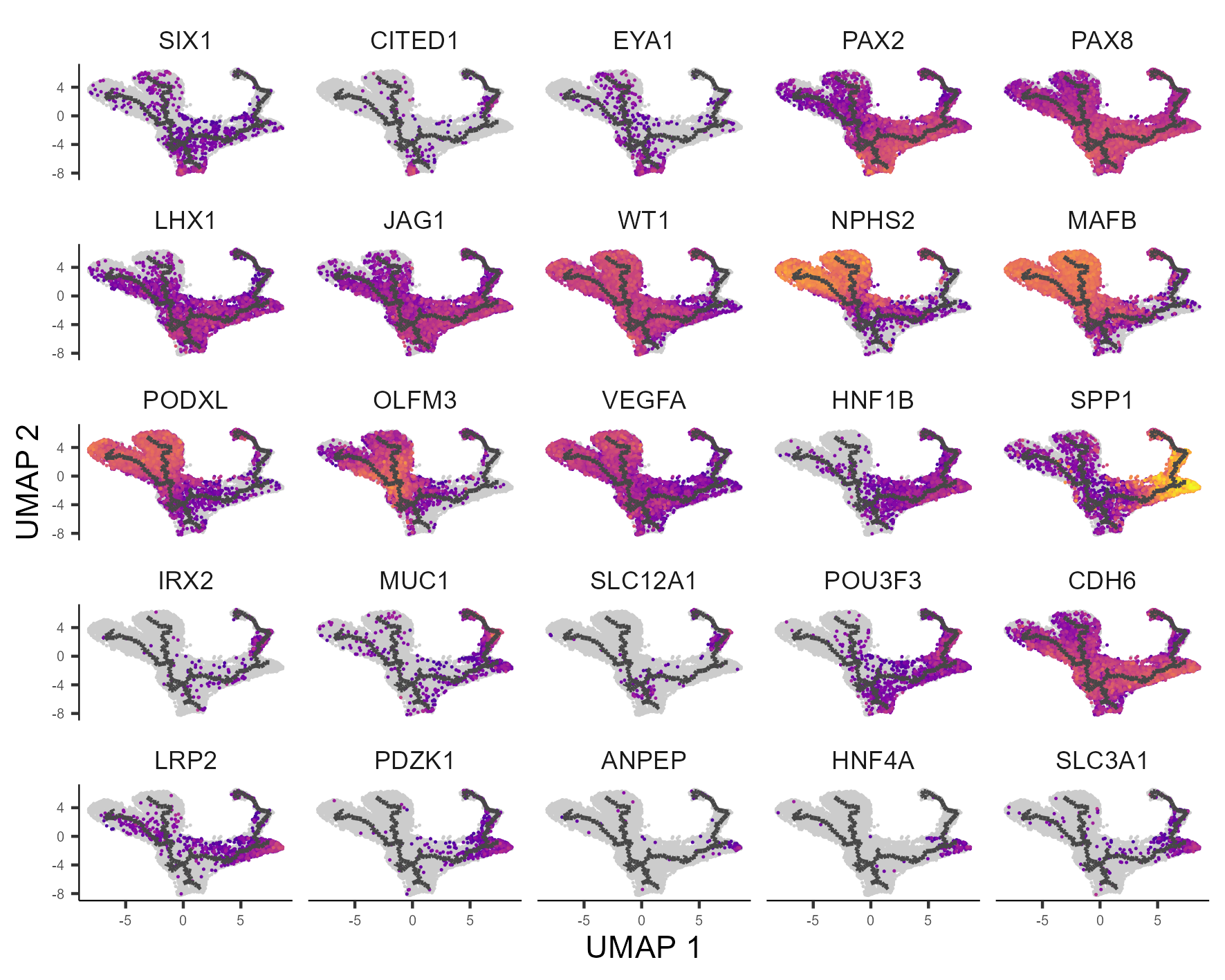

Supplement: Supplementary file 6 — Source data Fig. 4 [file 44318_2025_504_MOESM6_ESM.zip › Figure 4/4G/4G.tiff]

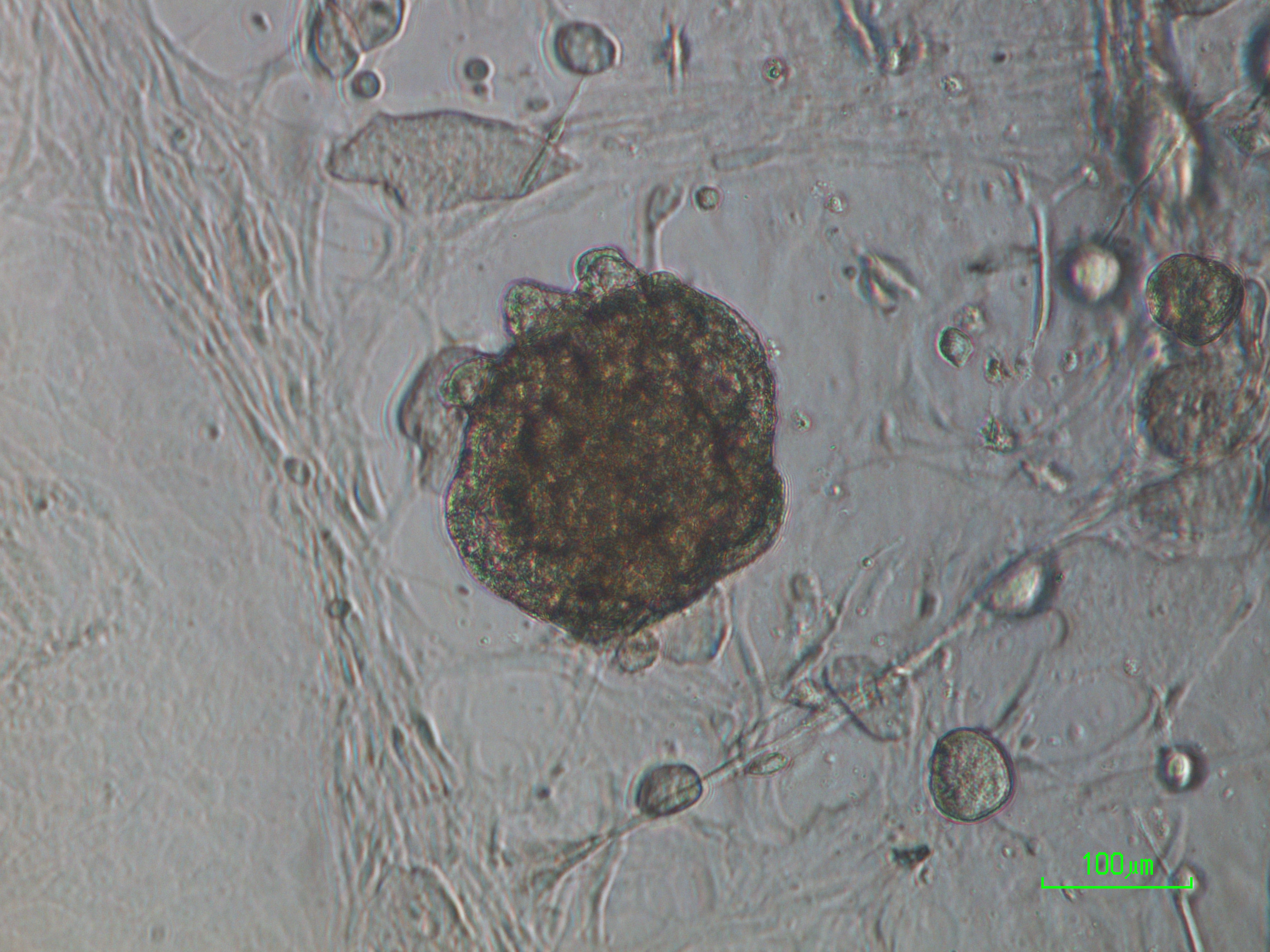

Supplement: Supplementary file 7 — Source data Fig. 5 [file 44318_2025_504_MOESM7_ESM.zip › Figure 5/5A/brightfield-magnification-hFKOs-DAPT-treatment.TIF]

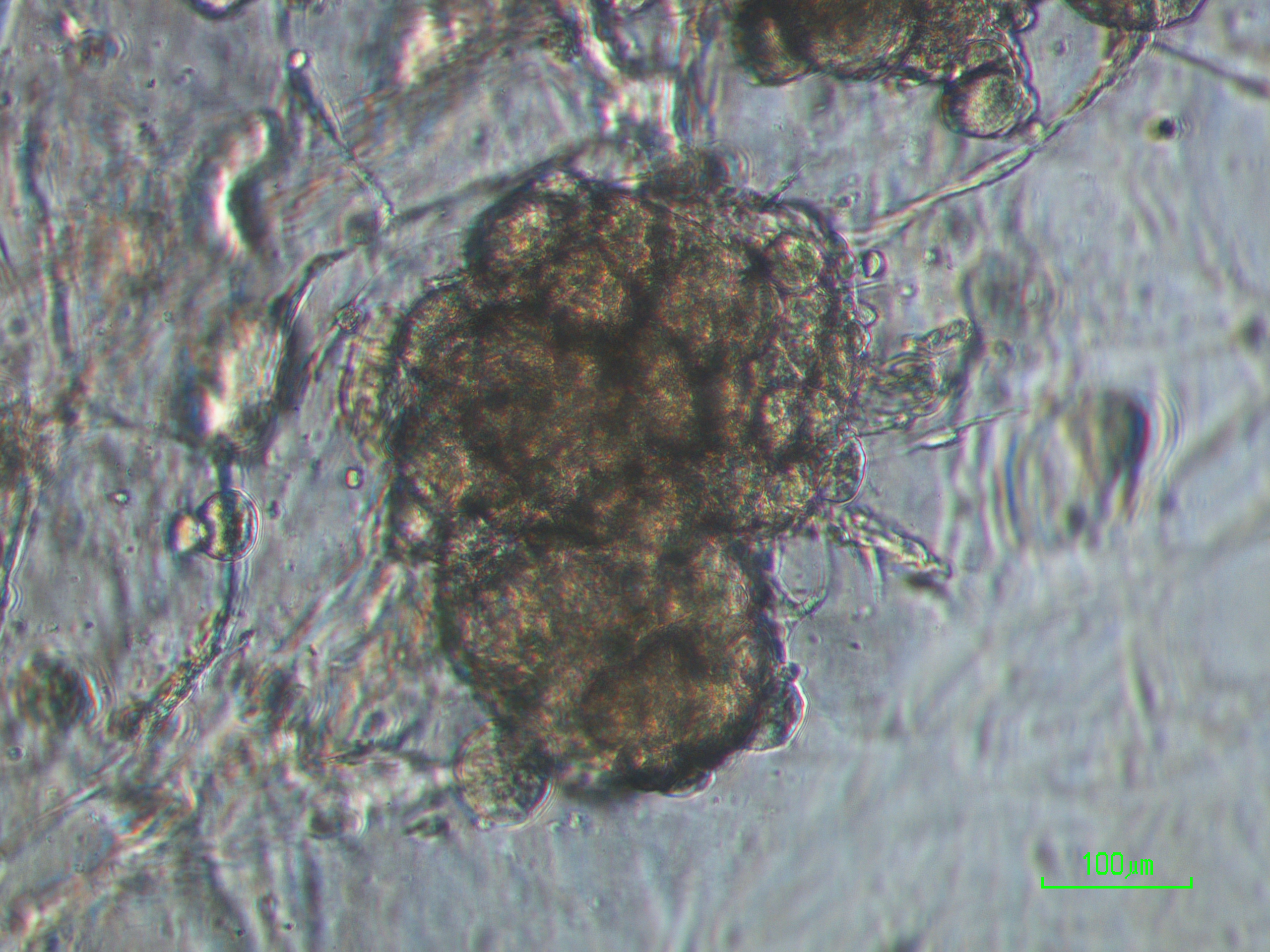

Supplement: Supplementary file 7 — Source data Fig. 5 [file 44318_2025_504_MOESM7_ESM.zip › Figure 5/5A/brightfield-magnification-hFKOs-DMSO-treatment.TIF]

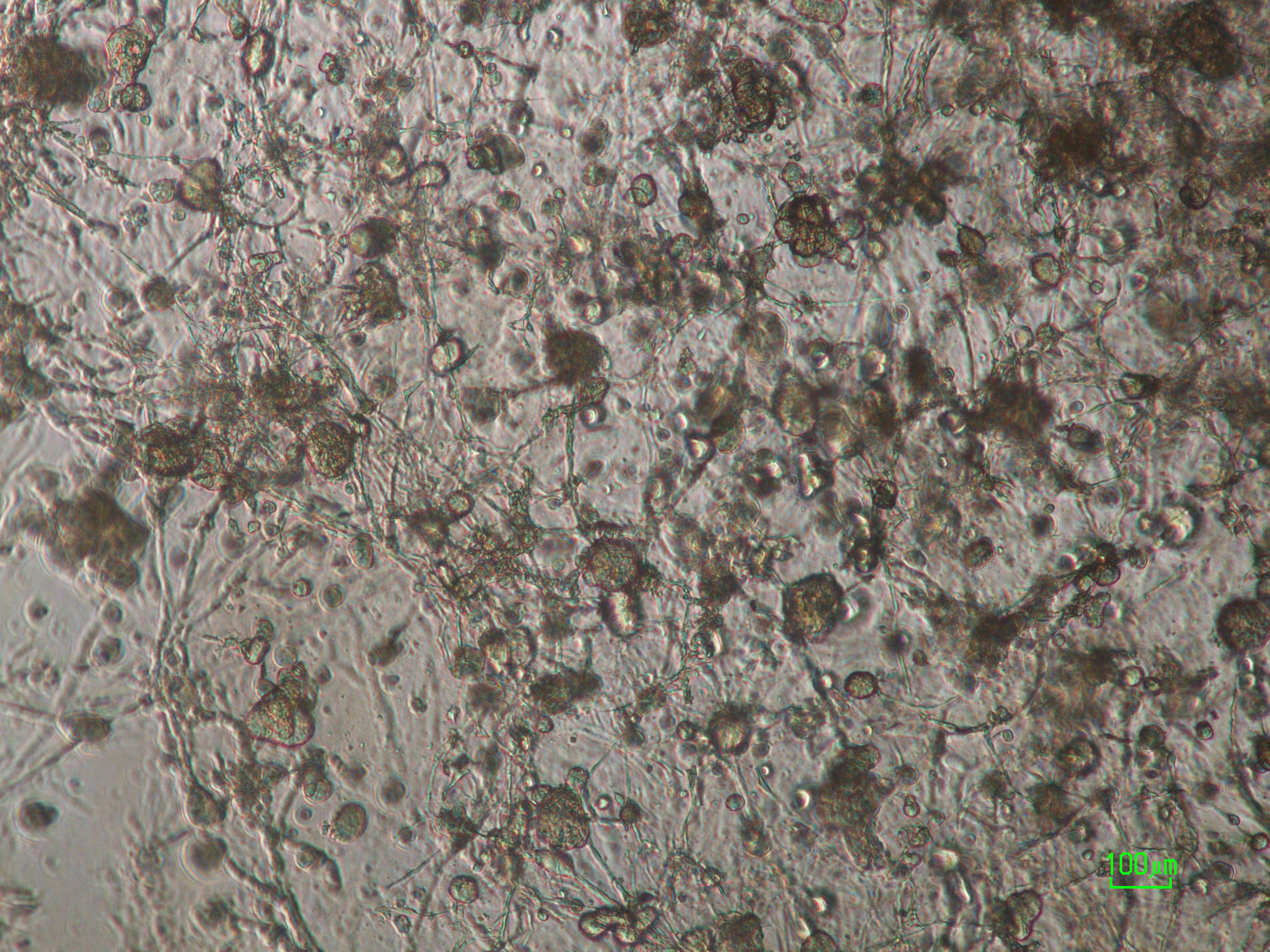

Supplement: Supplementary file 7 — Source data Fig. 5 [file 44318_2025_504_MOESM7_ESM.zip › Figure 5/5A/widefield-hFKOs-DAPT-treatment.TIF]

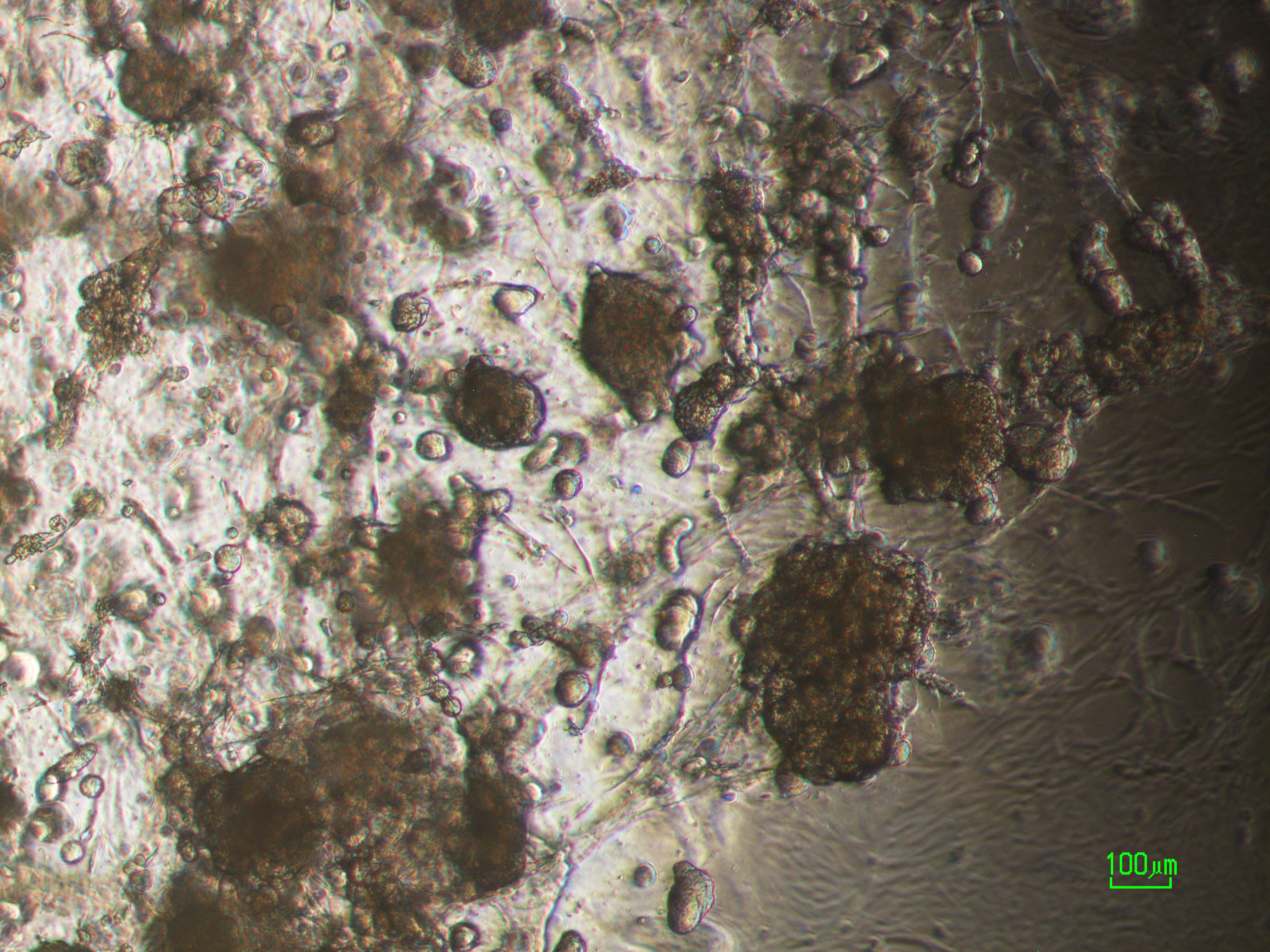

Supplement: Supplementary file 7 — Source data Fig. 5 [file 44318_2025_504_MOESM7_ESM.zip › Figure 5/5A/widefield-hFKOs-DMSO-treatment.TIF]

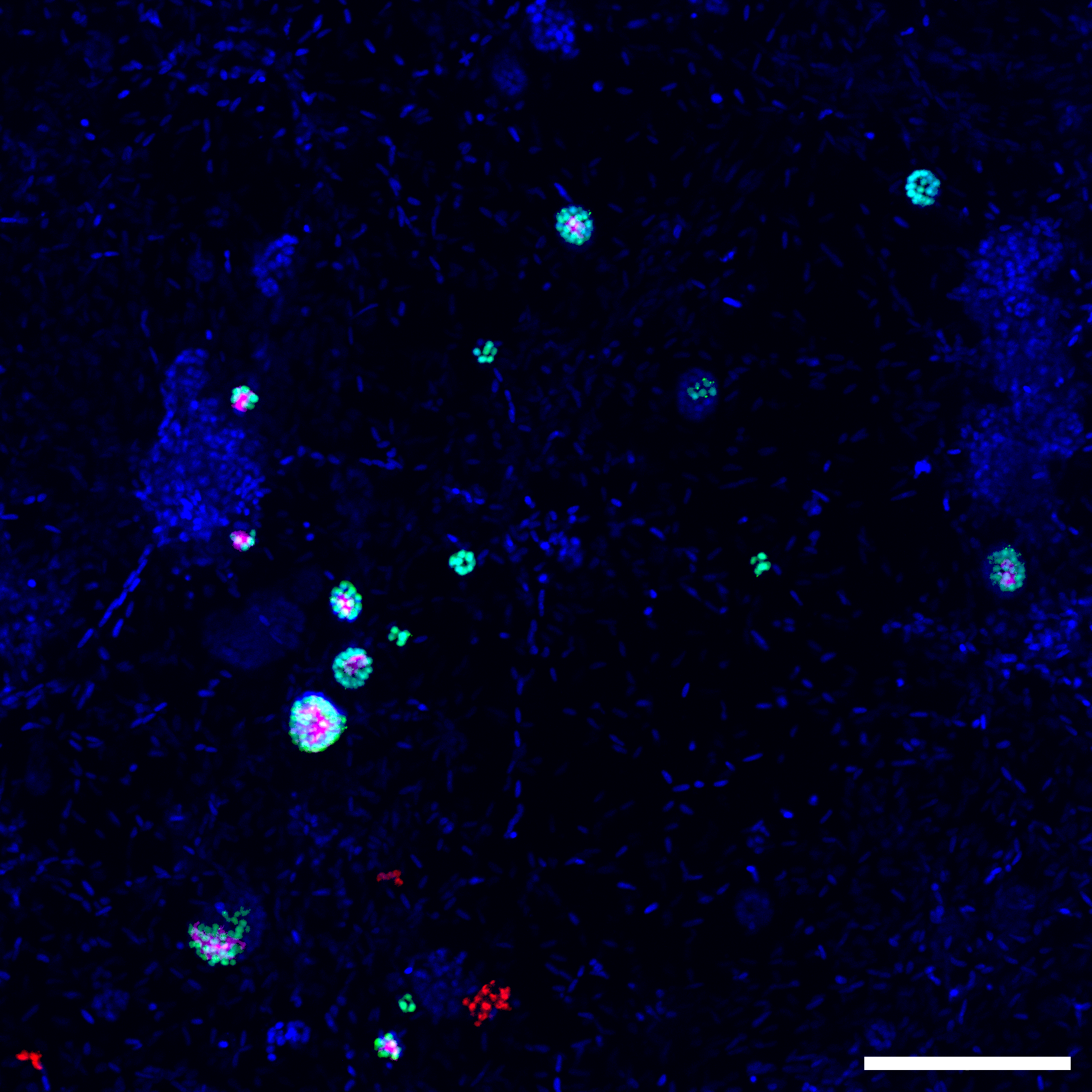

Supplement: Supplementary file 7 — Source data Fig. 5 [file 44318_2025_504_MOESM7_ESM.zip › Figure 5/5C/magnification-droplet-with-hFKOs-DAPT-treatment-CDH6-red-HNF1B-green-LTL-magenta-DAPI-blue-scalebar-200um.tif]

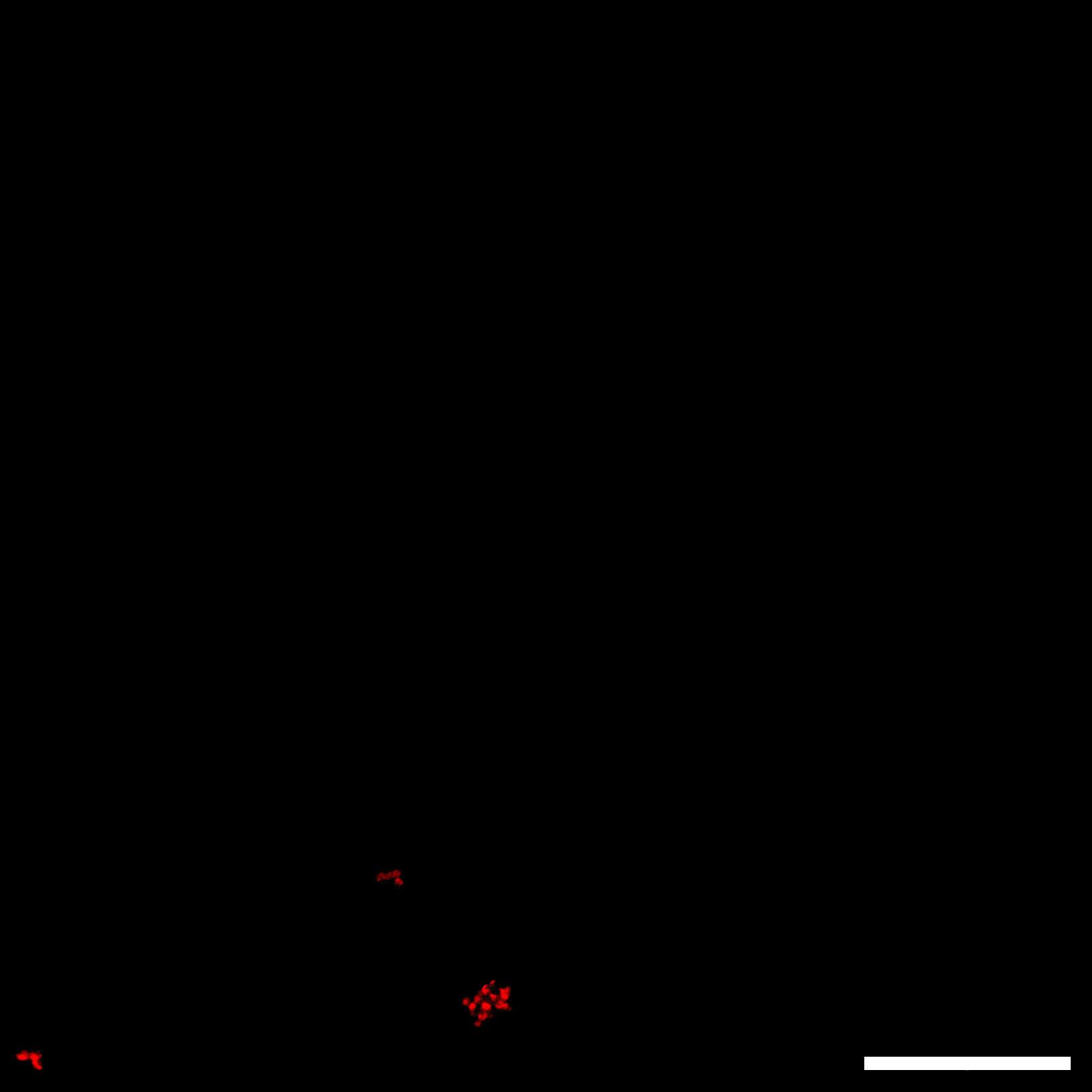

Supplement: Supplementary file 7 — Source data Fig. 5 [file 44318_2025_504_MOESM7_ESM.zip › Figure 5/5C/magnification-droplet-with-hFKOs-DAPT-treatment-CDH6-red-scalebar-200um.tif]

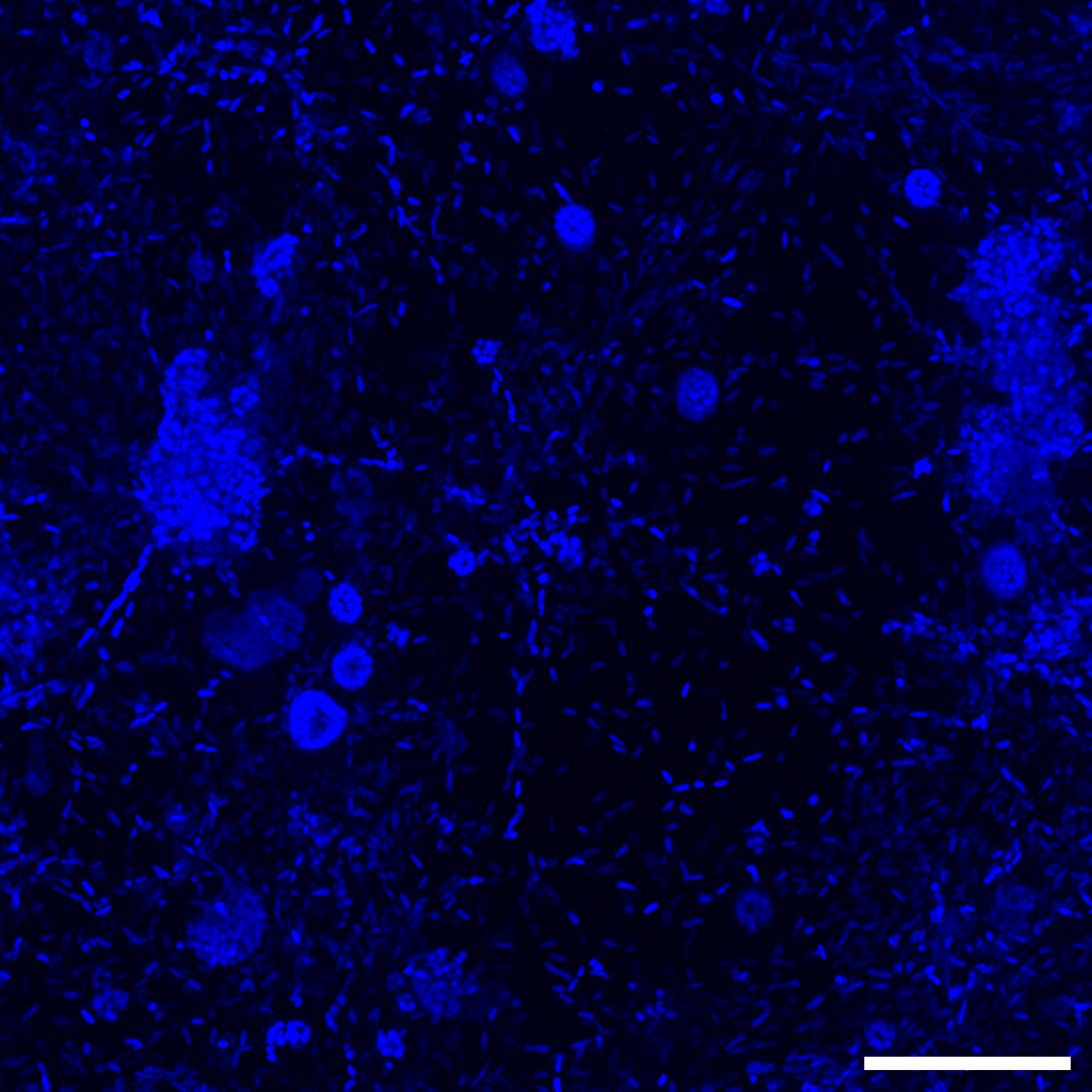

Supplement: Supplementary file 7 — Source data Fig. 5 [file 44318_2025_504_MOESM7_ESM.zip › Figure 5/5C/magnification-droplet-with-hFKOs-DAPT-treatment-DAPI-blue-scalebar-200um.png]

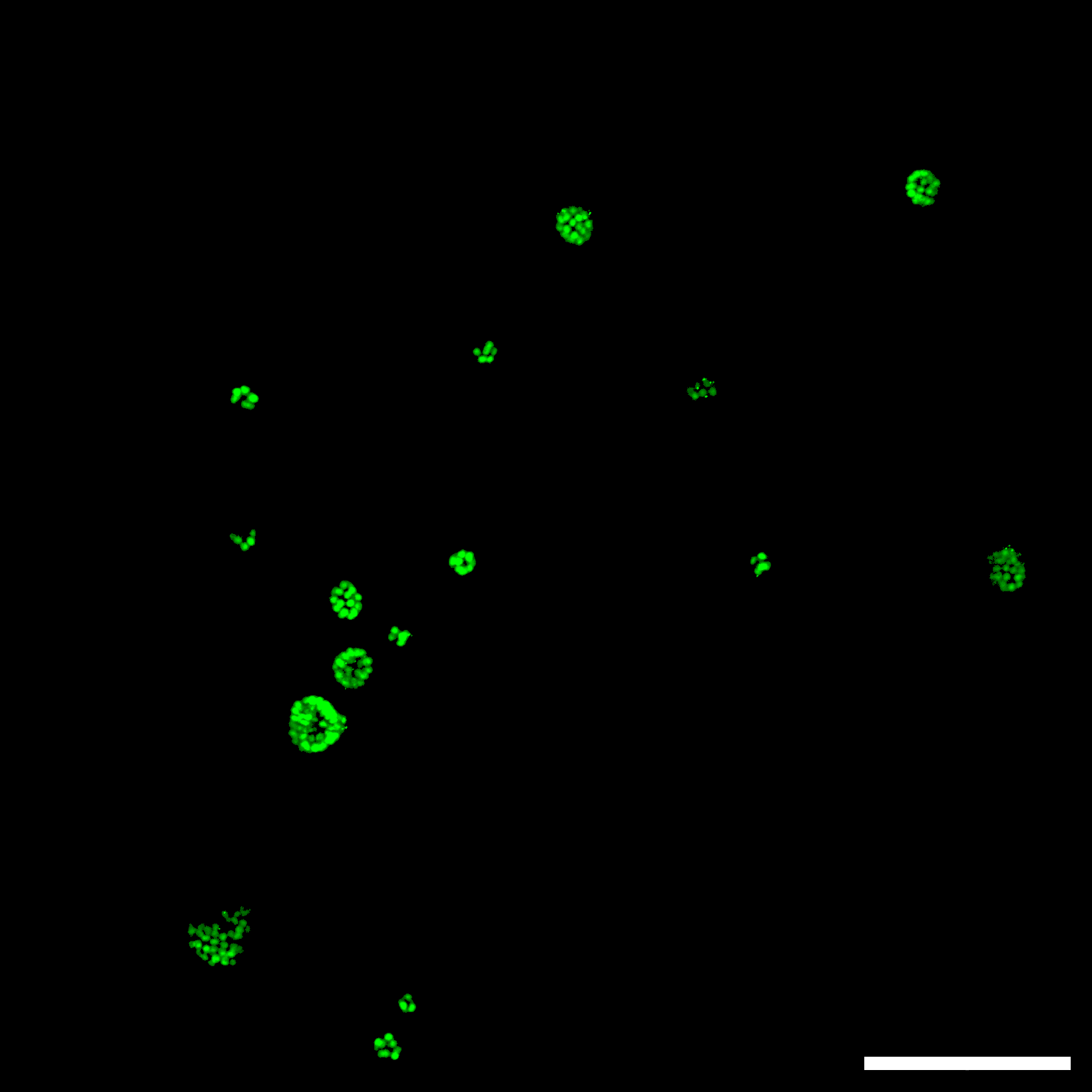

Supplement: Supplementary file 7 — Source data Fig. 5 [file 44318_2025_504_MOESM7_ESM.zip › Figure 5/5C/magnification-droplet-with-hFKOs-DAPT-treatment-HNF1B-green-scalebar-200um.tif]

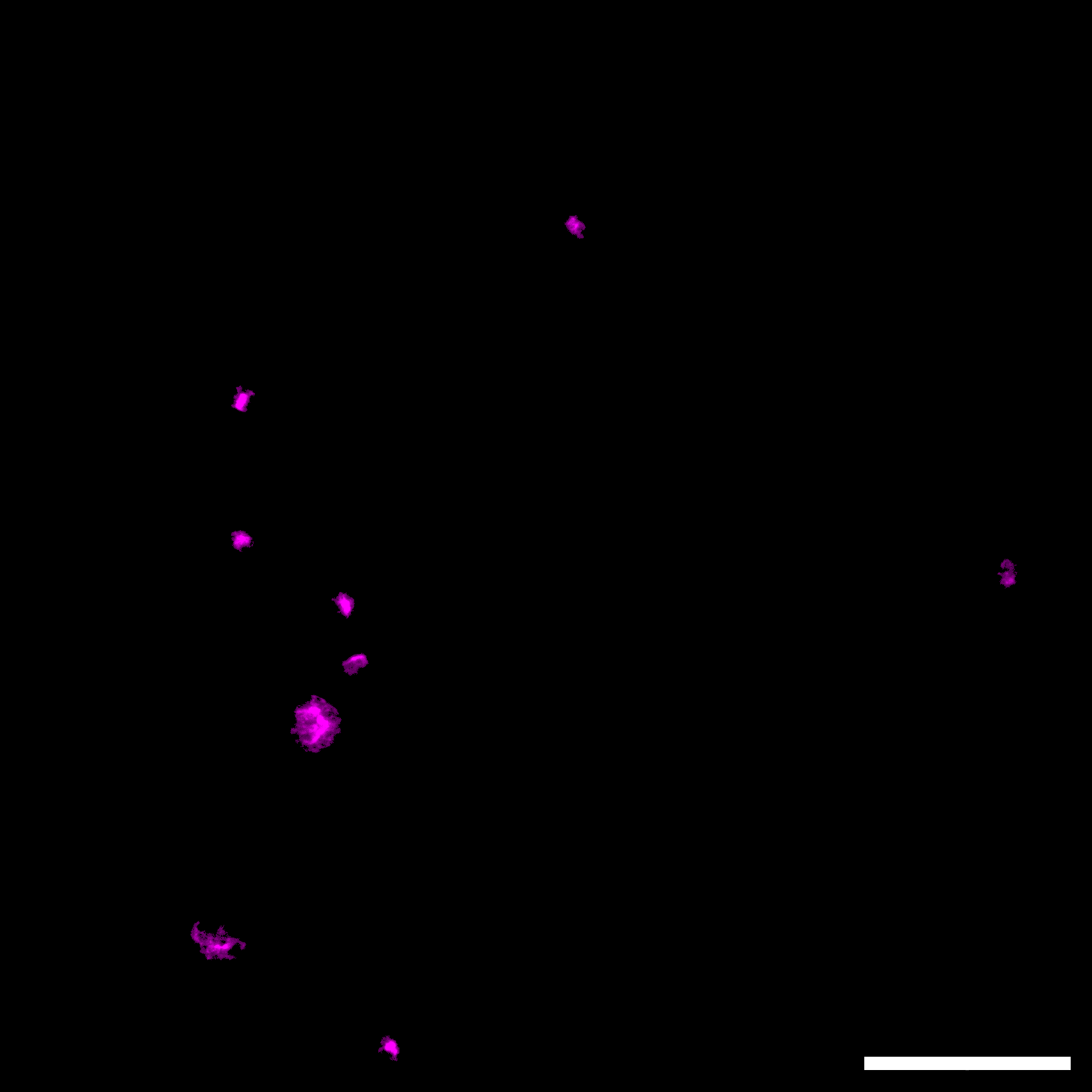

Supplement: Supplementary file 7 — Source data Fig. 5 [file 44318_2025_504_MOESM7_ESM.zip › Figure 5/5C/magnification-droplet-with-hFKOs-DAPT-treatment-LTL-magenta-200um.tif]

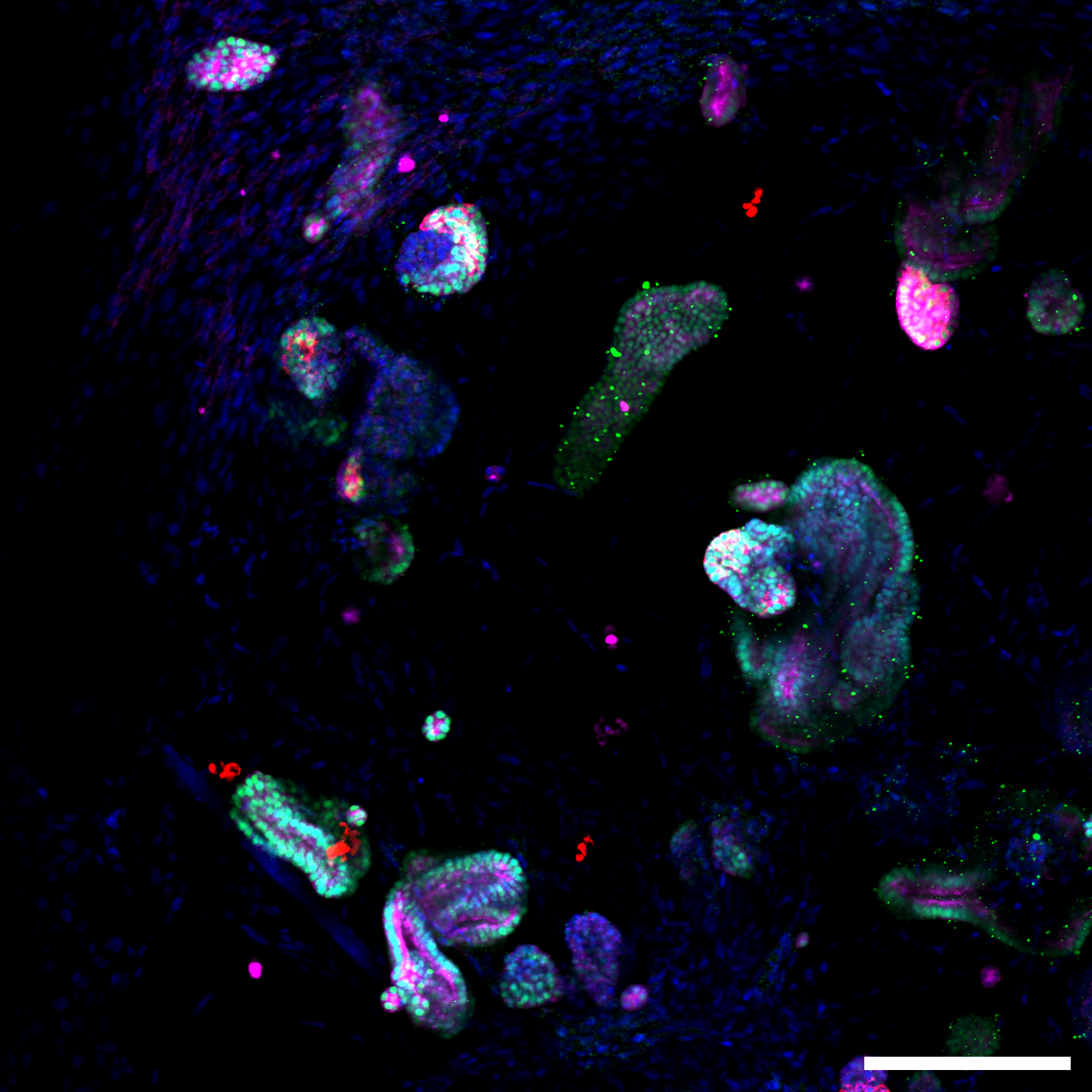

Supplement: Supplementary file 7 — Source data Fig. 5 [file 44318_2025_504_MOESM7_ESM.zip › Figure 5/5C/magnification-droplet-with-hFKOs-DMSO-treatment-CDH6-red-HNF1B-green-LTL-magenta-DAPI-blue-scalebar-200um.tif]

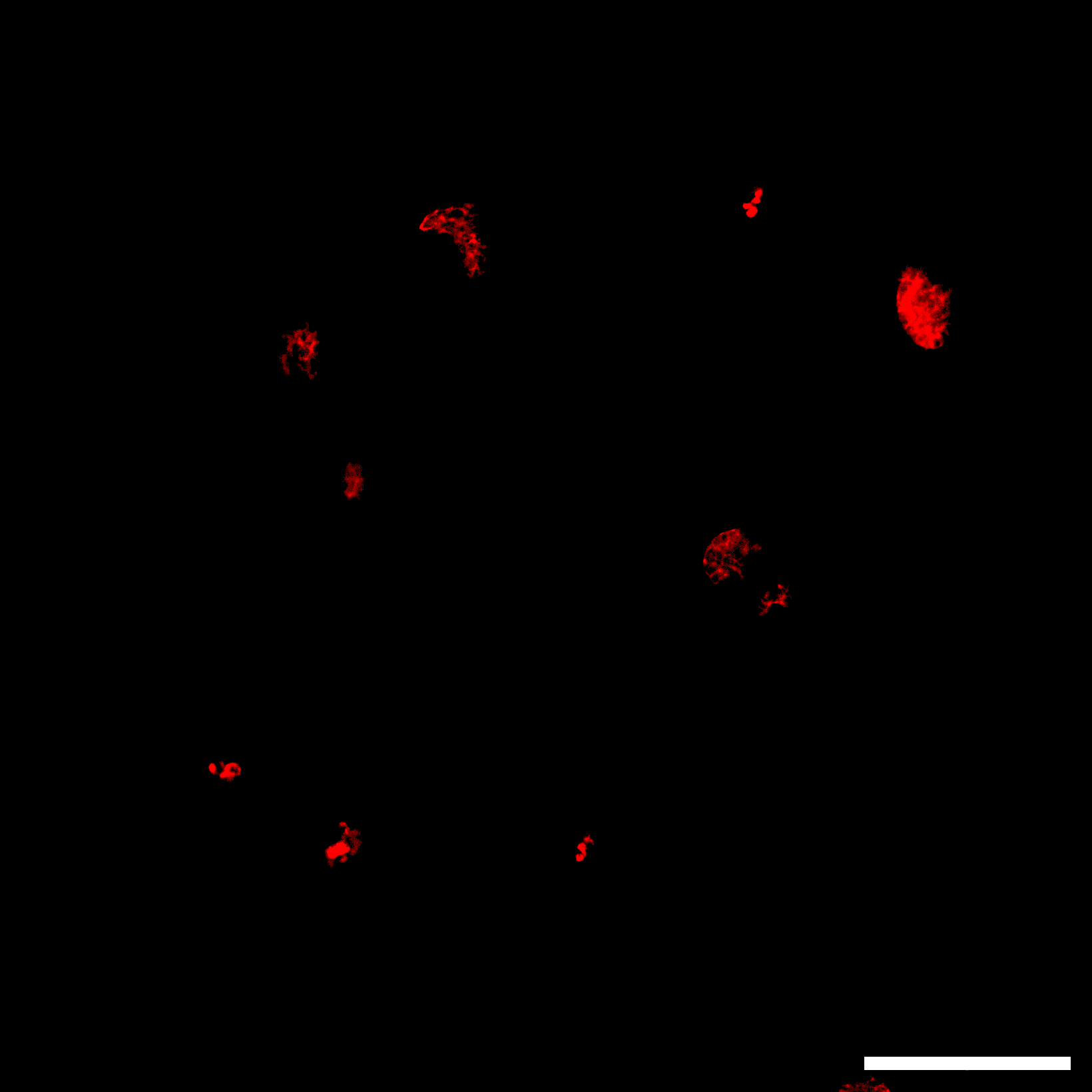

Supplement: Supplementary file 7 — Source data Fig. 5 [file 44318_2025_504_MOESM7_ESM.zip › Figure 5/5C/magnification-droplet-with-hFKOs-DMSO-treatment-CDH6-red-scalebar-200um.tif]

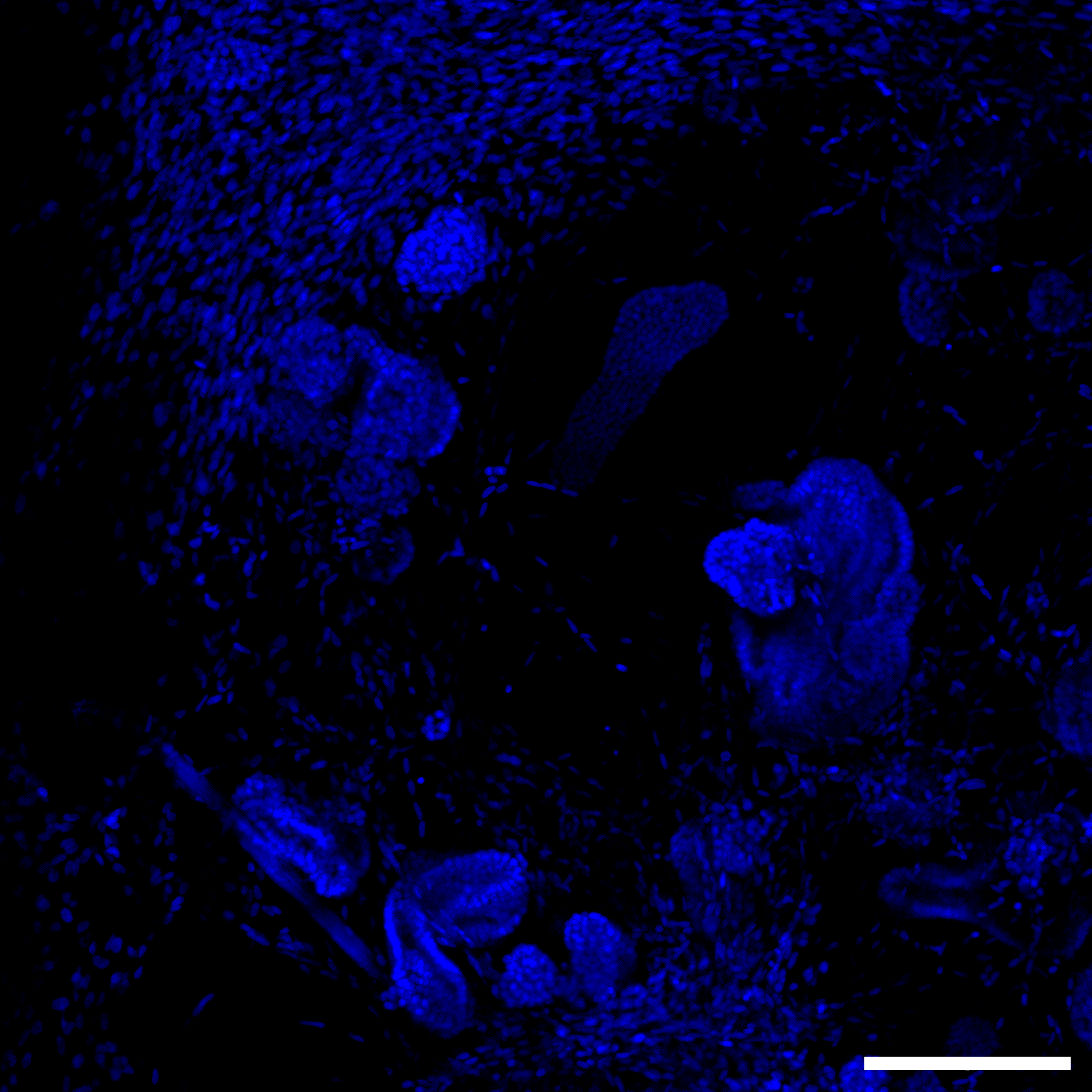

Supplement: Supplementary file 7 — Source data Fig. 5 [file 44318_2025_504_MOESM7_ESM.zip › Figure 5/5C/magnification-droplet-with-hFKOs-DMSO-treatment-DAPI-blue-scalebar-200um.png]

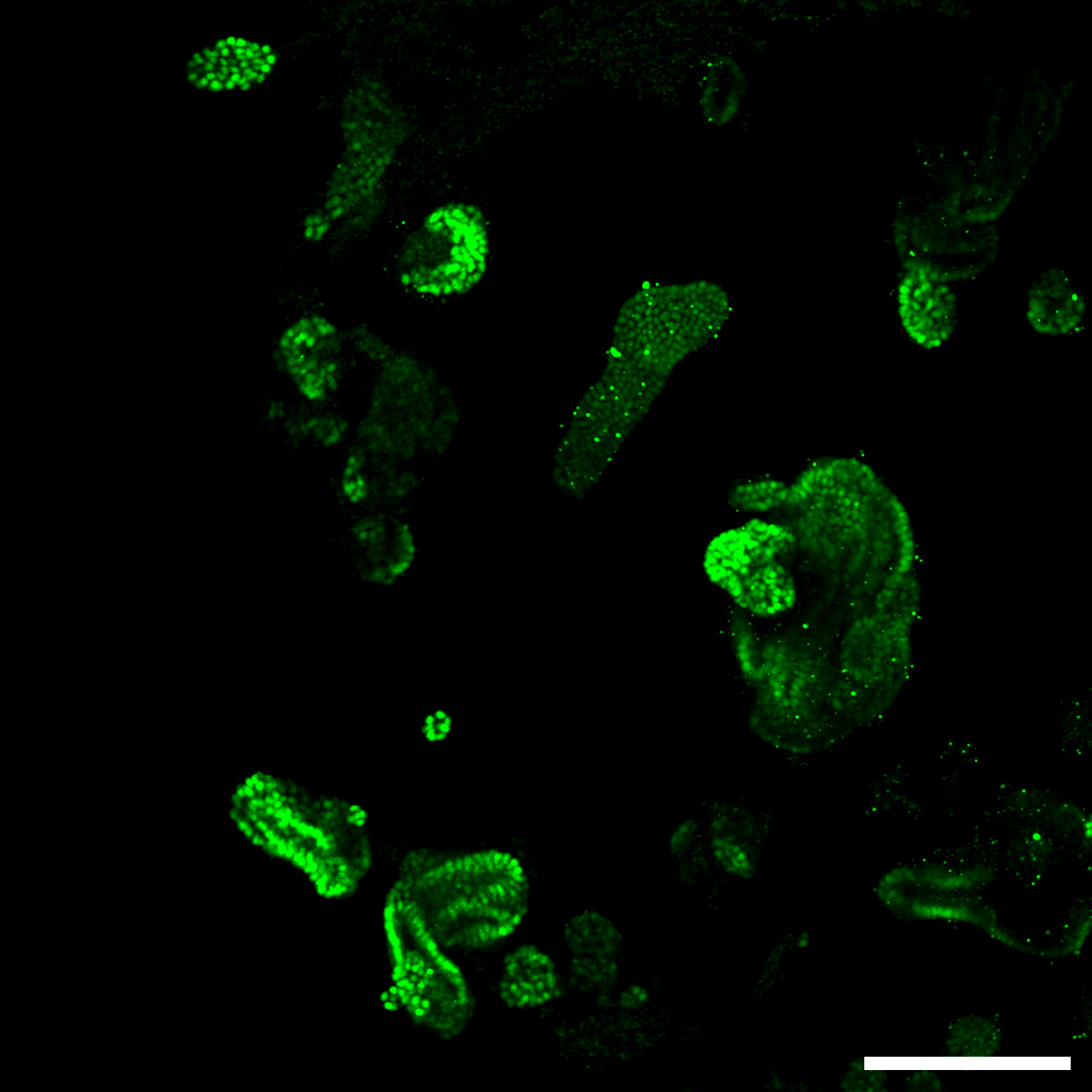

Supplement: Supplementary file 7 — Source data Fig. 5 [file 44318_2025_504_MOESM7_ESM.zip › Figure 5/5C/magnification-droplet-with-hFKOs-DMSO-treatment-HNF1B-green-scalebar-200um.tif]

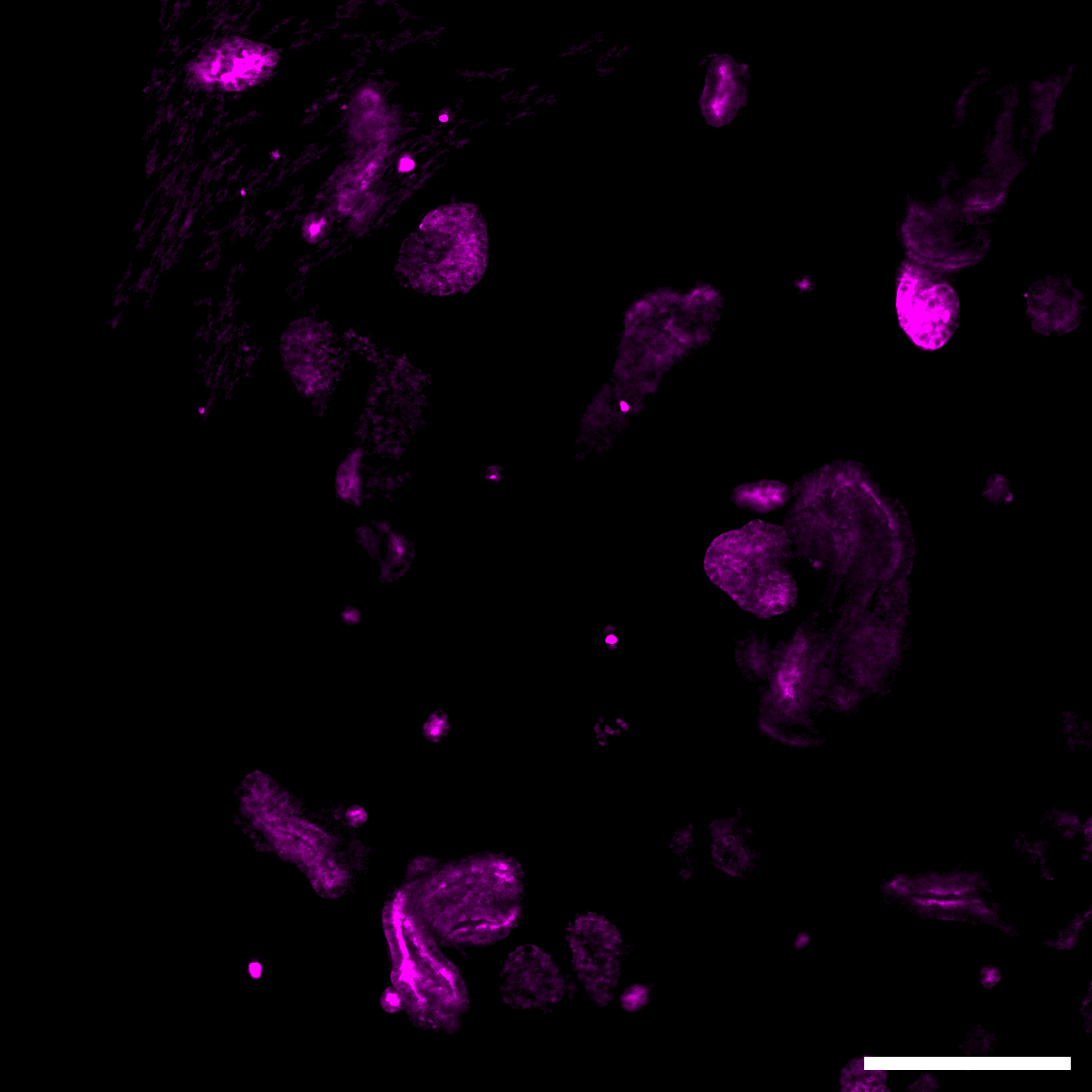

Supplement: Supplementary file 7 — Source data Fig. 5 [file 44318_2025_504_MOESM7_ESM.zip › Figure 5/5C/magnification-droplet-with-hFKOs-DMSO-treatment-LTL-magenta-scalebar-200um.tif]

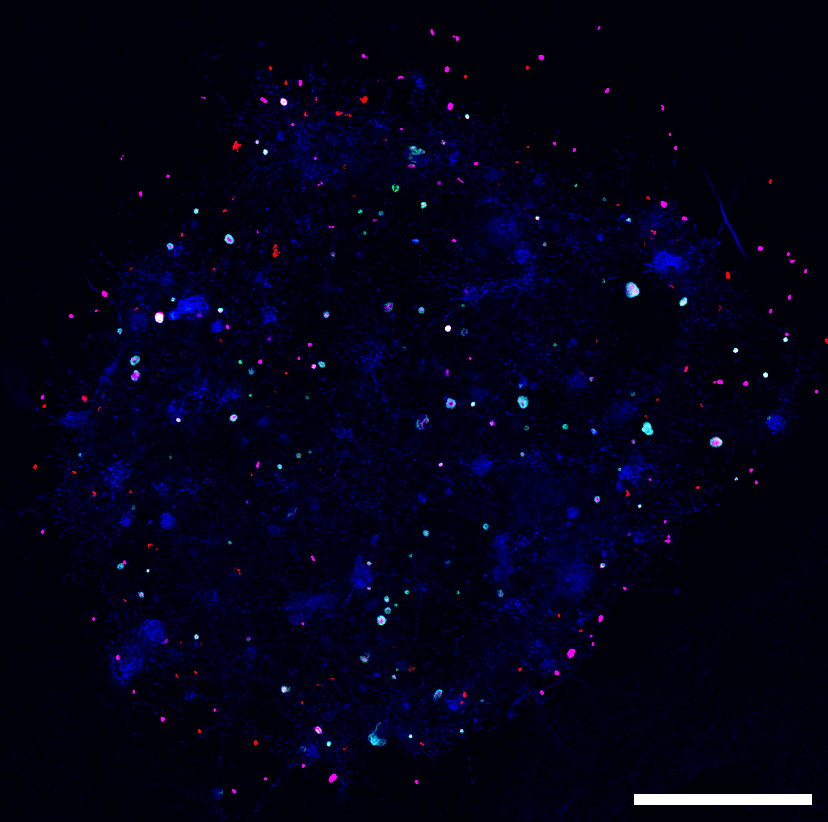

Supplement: Supplementary file 7 — Source data Fig. 5 [file 44318_2025_504_MOESM7_ESM.zip › Figure 5/5C/widefield-droplet-with-hFKOs-DAPT-treatment-CDH6-red-HNF1B-green-LTL-magenta-DAPI-blue-scalebar-1000um.tif]

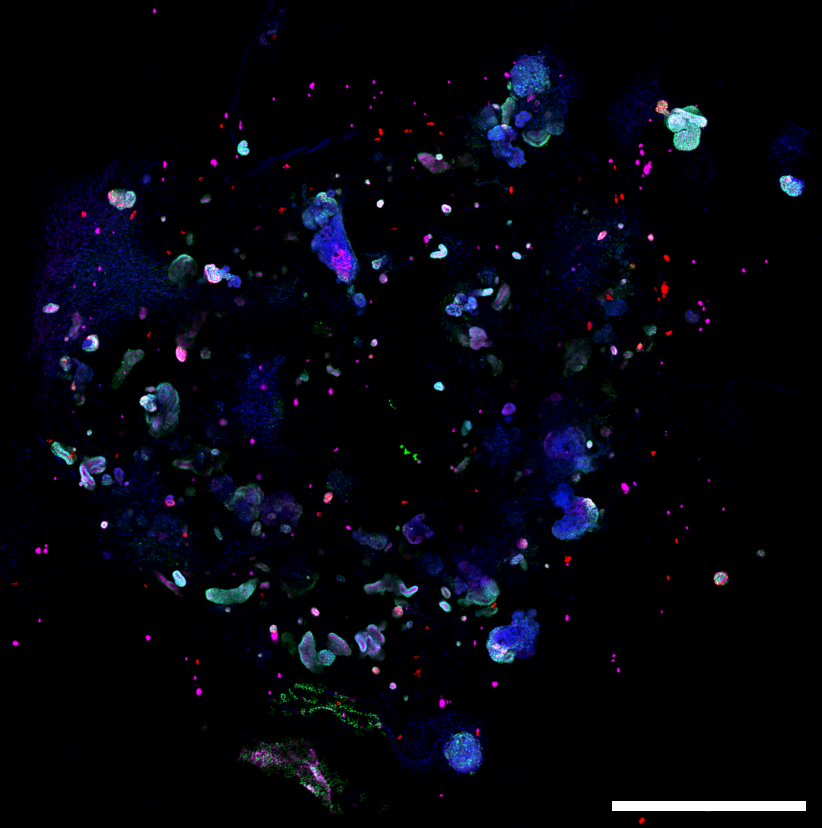

Supplement: Supplementary file 7 — Source data Fig. 5 [file 44318_2025_504_MOESM7_ESM.zip › Figure 5/5C/widefield-droplet-with-hFKOs-DMSO-treatment-CDH6-red-HNF1B-green-LTL-magenta-DAPI-blue-scalebar-1000um.tif]

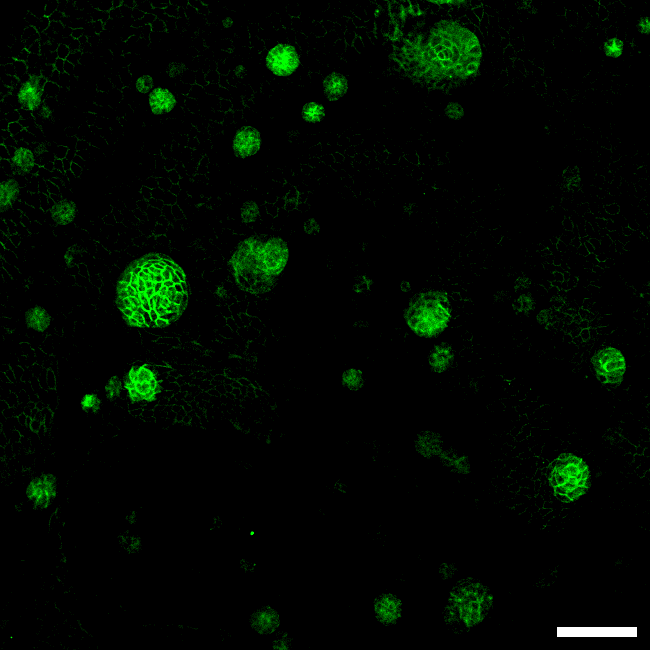

Supplement: Supplementary file 7 — Source data Fig. 5 [file 44318_2025_504_MOESM7_ESM.zip › Figure 5/5D/magnification-droplet-with-hFKOs-DAPT-treatment-ECAD-green-scalebar-100um.tif]

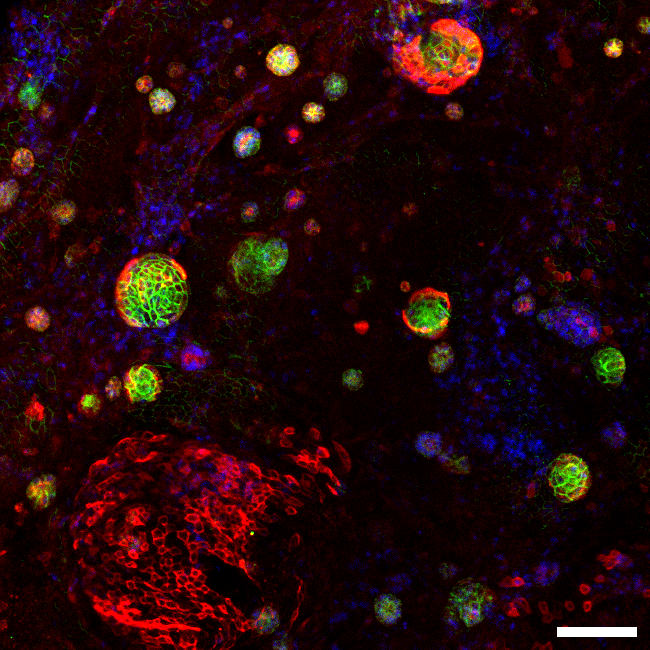

Supplement: Supplementary file 7 — Source data Fig. 5 [file 44318_2025_504_MOESM7_ESM.zip › Figure 5/5D/magnification-droplet-with-hFKOs-DAPT-treatment-EMA-red-ECAD-green-DAPI-blue-scalebar-100um.tif]

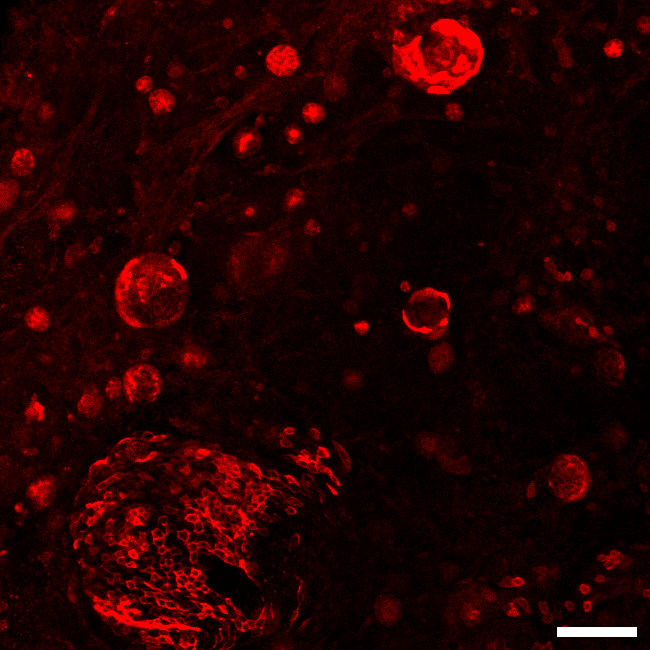

Supplement: Supplementary file 7 — Source data Fig. 5 [file 44318_2025_504_MOESM7_ESM.zip › Figure 5/5D/magnification-droplet-with-hFKOs-DAPT-treatment-EMA-red-scalebar-100um.tif]
