# Supplementary figures and images for: Human fetal kidney organoids model early human nephrogenesis and Notch-driven cell fate (part 2 of 2)
Source: EMBO J. 2025 Jul 21;44(17):4681–719. doi: 10.1038/s44318-025-00504-2 (PMC12402132; doi:10.1038/s44318-025-00504-2)

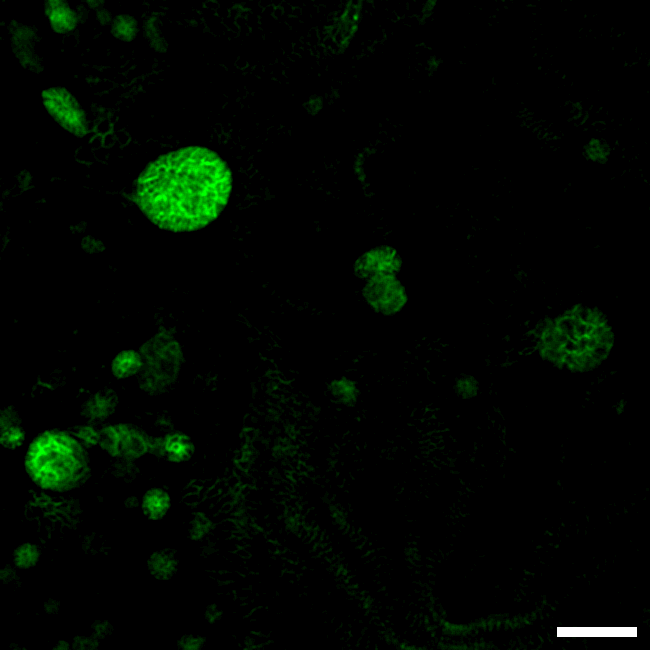

Supplement: Supplementary file 7 — Source data Fig. 5 [file 44318_2025_504_MOESM7_ESM.zip › Figure 5/5D/magnification-droplet-with-hFKOs-DMSO-treatment-ECAD-green-scalebar-200um.tif]

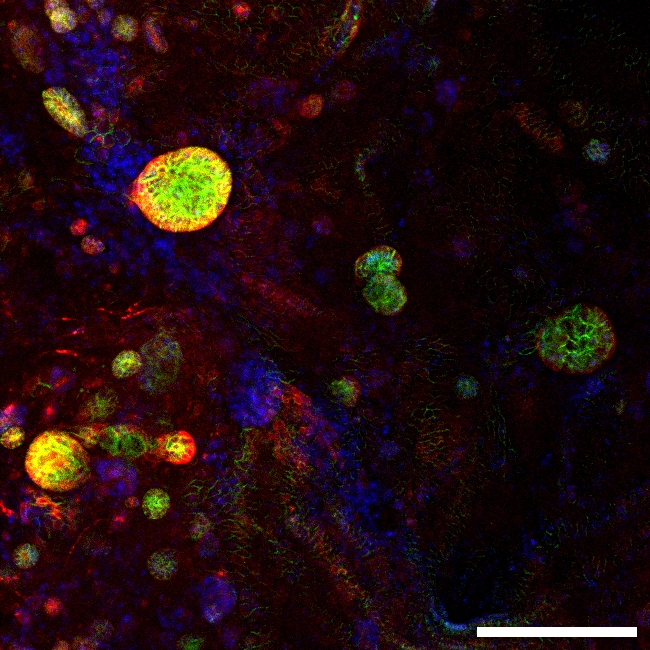

Supplement: Supplementary file 7 — Source data Fig. 5 [file 44318_2025_504_MOESM7_ESM.zip › Figure 5/5D/magnification-droplet-with-hFKOs-DMSO-treatment-EMA-red-ECAD-green-DAPI-blue-scalebar-200um.tif]

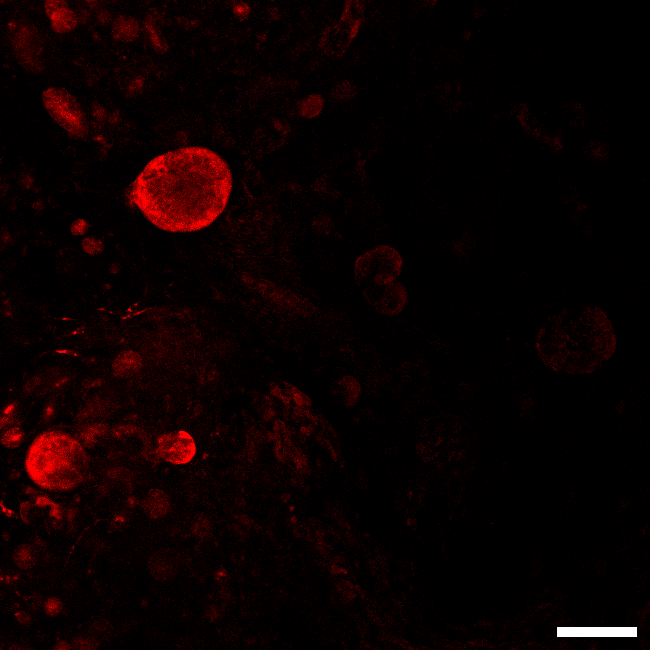

Supplement: Supplementary file 7 — Source data Fig. 5 [file 44318_2025_504_MOESM7_ESM.zip › Figure 5/5D/magnification-droplet-with-hFKOs-DMSO-treatment-EMA-red-scalebar-200um.tif]

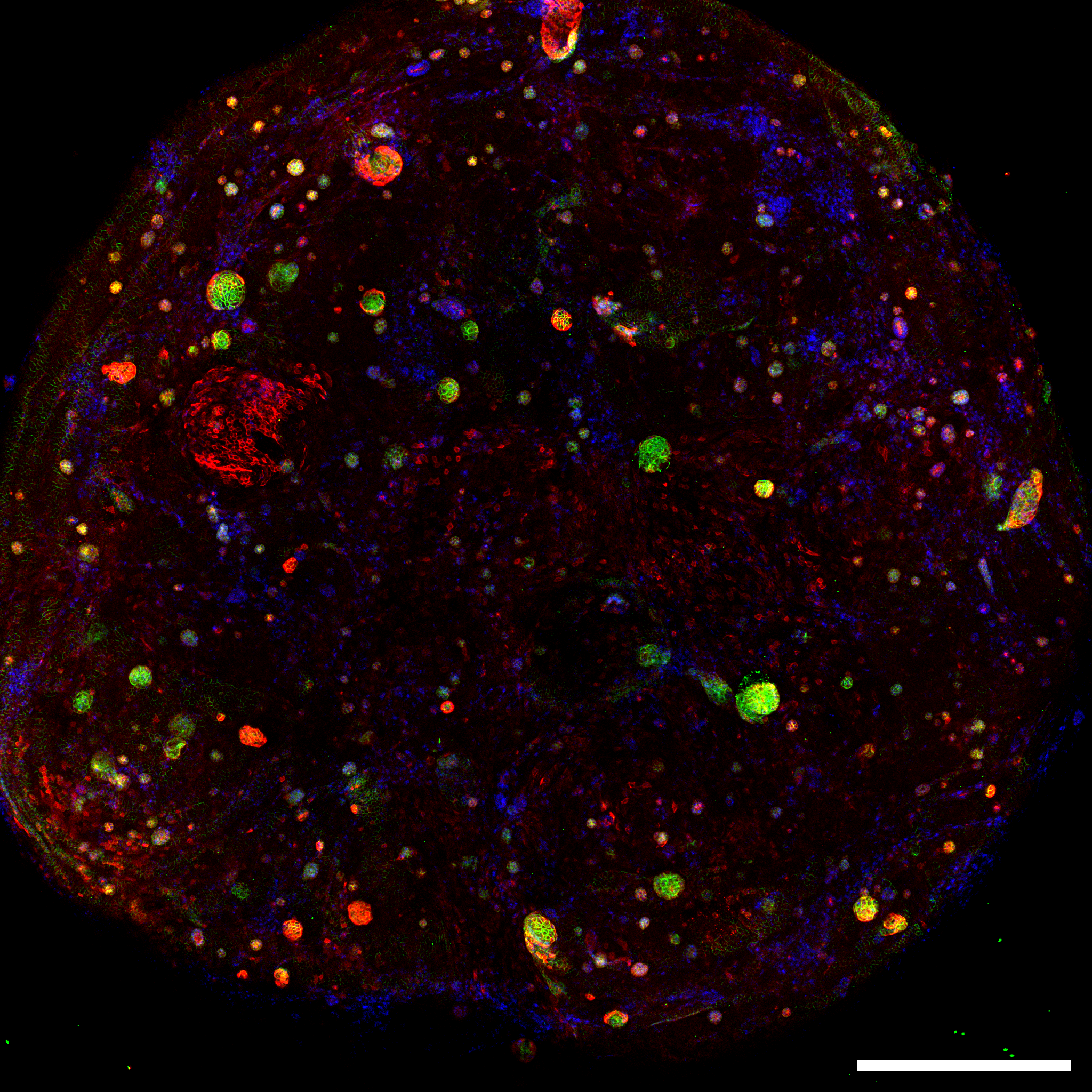

Supplement: Supplementary file 7 — Source data Fig. 5 [file 44318_2025_504_MOESM7_ESM.zip › Figure 5/5D/widefield-droplet-with-hFKOs-DAPT-treatment-EMA-red-ECAD-green-DAPI-blue-scalebar-500um.tif]

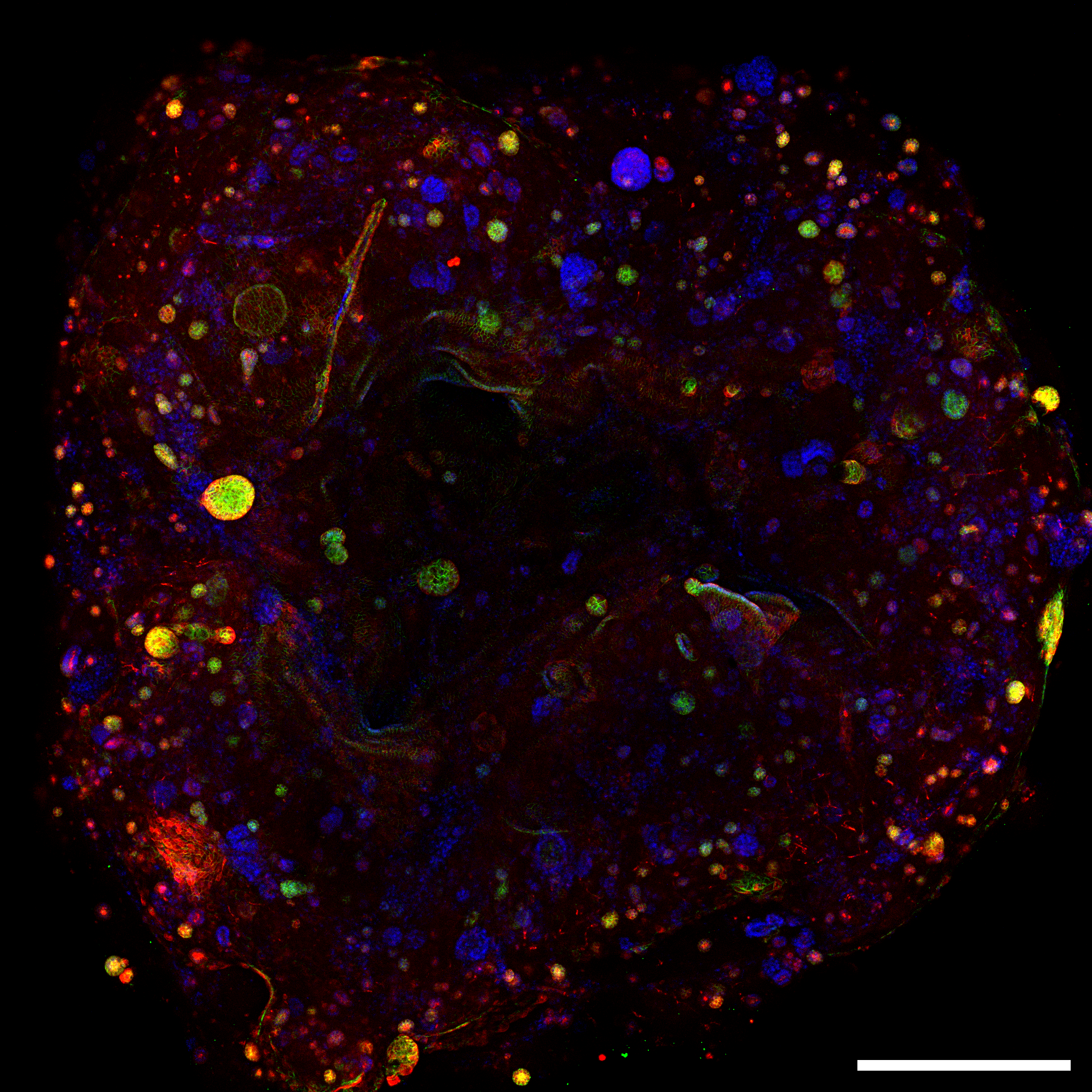

Supplement: Supplementary file 7 — Source data Fig. 5 [file 44318_2025_504_MOESM7_ESM.zip › Figure 5/5D/widefield-droplet-with-hFKOs-DMSO-treatment-EMA-red-ECAD-green-DAPI-blue-scalebar-500um.tif]

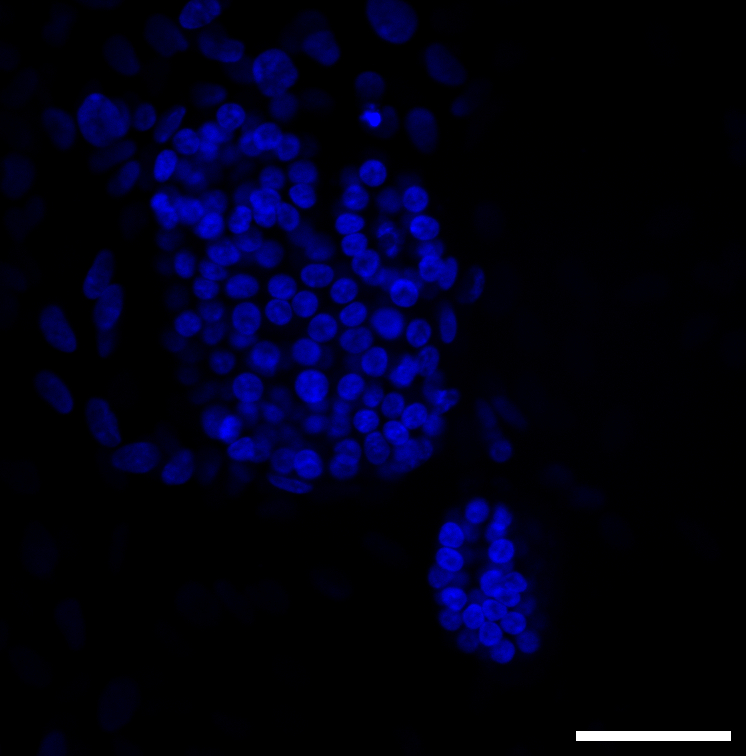

Supplement: Supplementary file 7 — Source data Fig. 5 [file 44318_2025_504_MOESM7_ESM.zip › Figure 5/5E/hFKO-DMSO-treated-DAPI-blue-scalebar-100um.tif]

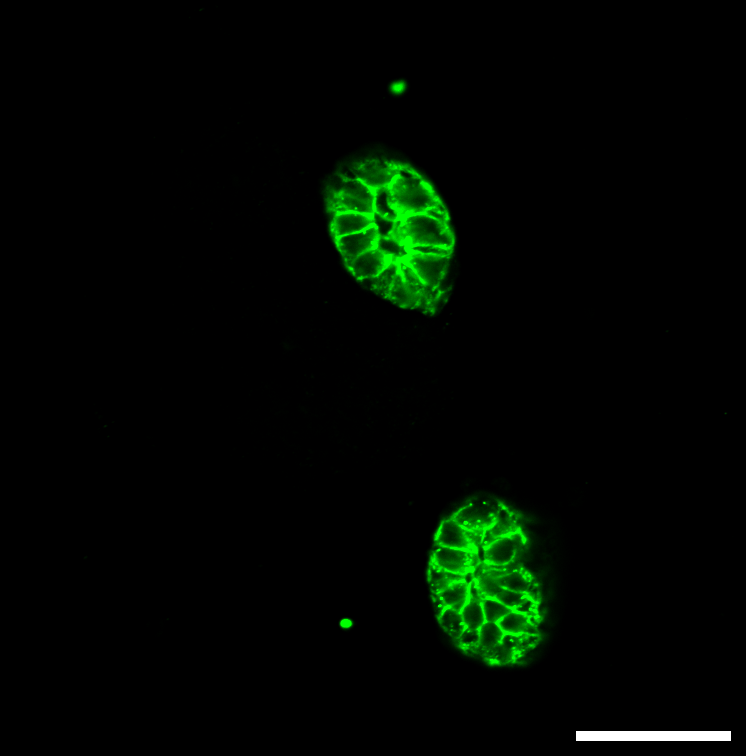

Supplement: Supplementary file 7 — Source data Fig. 5 [file 44318_2025_504_MOESM7_ESM.zip › Figure 5/5E/hFKO-DMSO-treated-ECAD-green-scalebar-100um.tif]

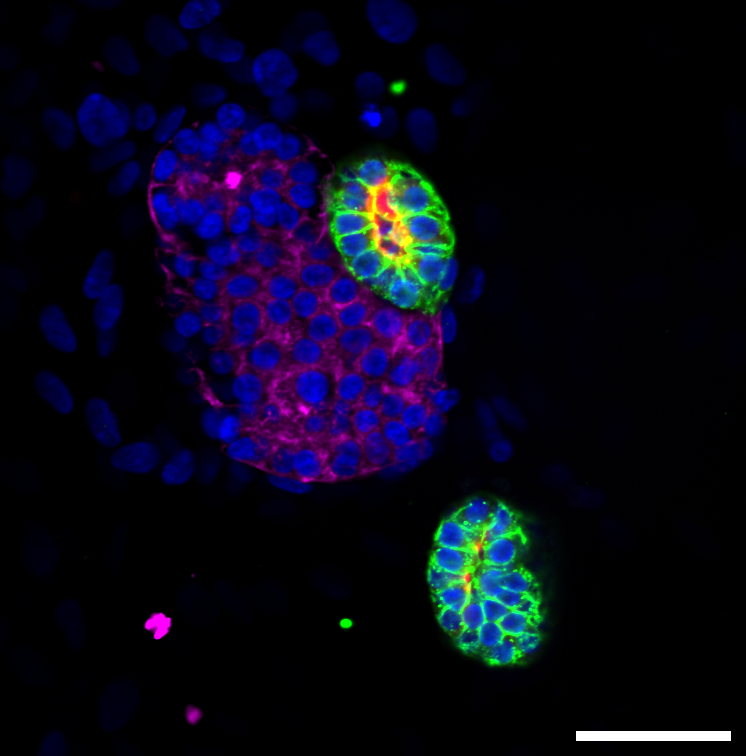

Supplement: Supplementary file 7 — Source data Fig. 5 [file 44318_2025_504_MOESM7_ESM.zip › Figure 5/5E/hFKO-DMSO-treated-EMA-red-ECAD-green-LTL-magenta-DAPI-blue-scalebar-100um.tif]

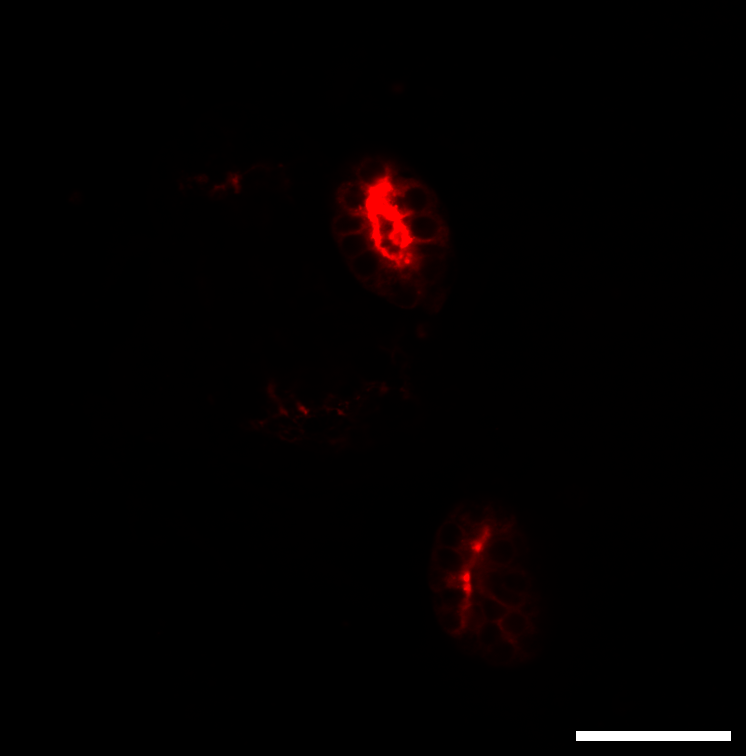

Supplement: Supplementary file 7 — Source data Fig. 5 [file 44318_2025_504_MOESM7_ESM.zip › Figure 5/5E/hFKO-DMSO-treated-EMA-red-scalebar-100um.tif]

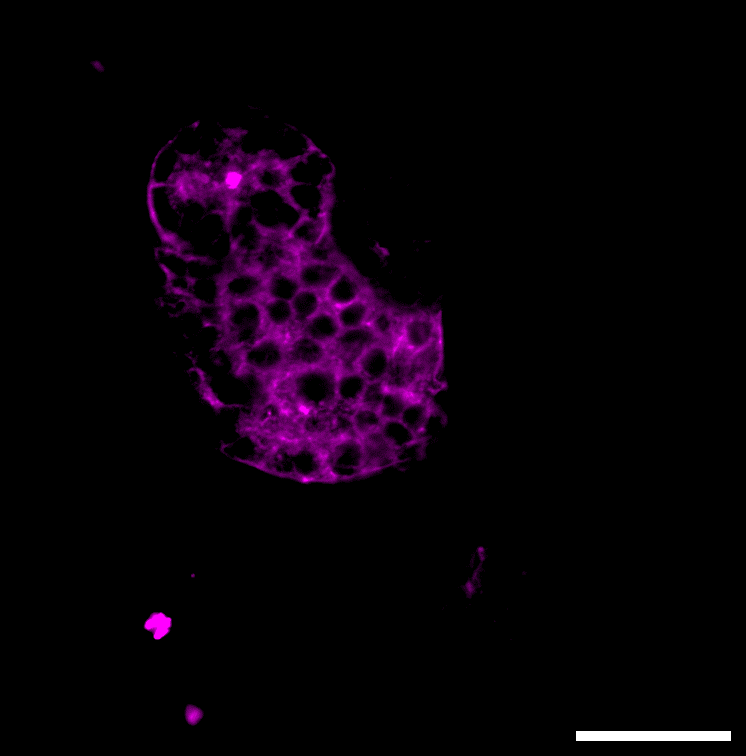

Supplement: Supplementary file 7 — Source data Fig. 5 [file 44318_2025_504_MOESM7_ESM.zip › Figure 5/5E/hFKO-DMSO-treated-LTL-magenta-scalebar-100um.png]

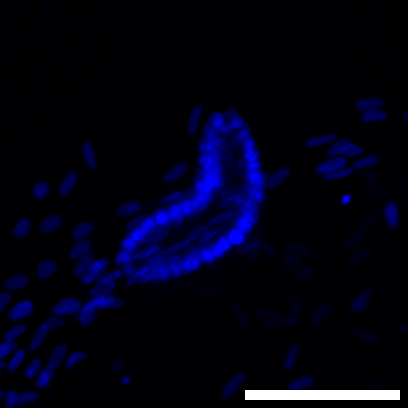

Supplement: Supplementary file 7 — Source data Fig. 5 [file 44318_2025_504_MOESM7_ESM.zip › Figure 5/5F/hFKO-DAPT-treated-DAPI-blue-scalebar-100um.tif]

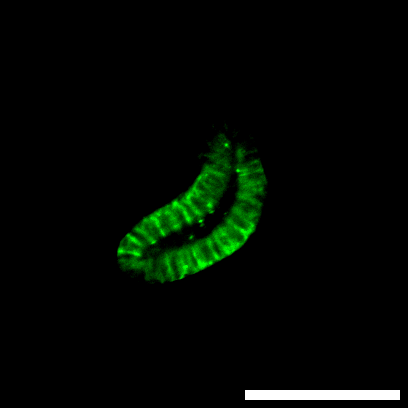

Supplement: Supplementary file 7 — Source data Fig. 5 [file 44318_2025_504_MOESM7_ESM.zip › Figure 5/5F/hFKO-DAPT-treated-ECAD-green-scalebar-100um.tif]

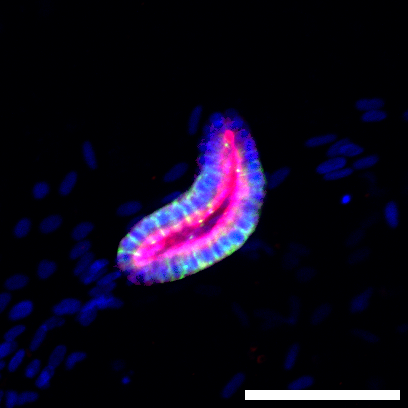

Supplement: Supplementary file 7 — Source data Fig. 5 [file 44318_2025_504_MOESM7_ESM.zip › Figure 5/5F/hFKO-DAPT-treated-EMA-red-ECAD-green-LTL-magenta-DAPI-blue-scalebar-100um.tif]

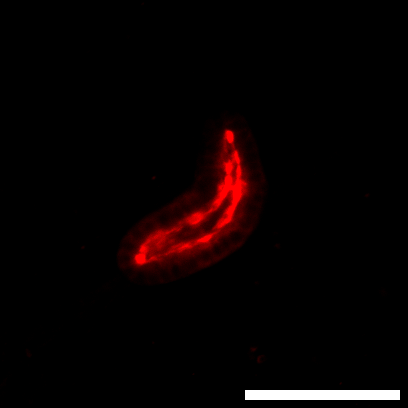

Supplement: Supplementary file 7 — Source data Fig. 5 [file 44318_2025_504_MOESM7_ESM.zip › Figure 5/5F/hFKO-DAPT-treated-EMA-red-scalebar-100um.tif]

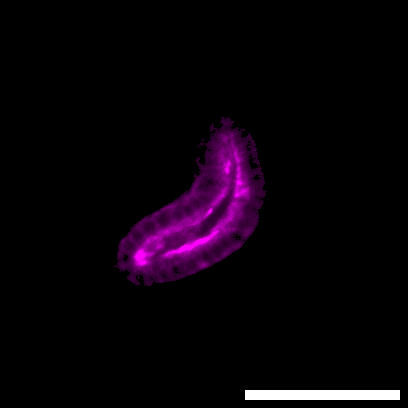

Supplement: Supplementary file 7 — Source data Fig. 5 [file 44318_2025_504_MOESM7_ESM.zip › Figure 5/5F/hFKO-DAPT-treated-LTL-magenta-scalebar-100um.tif]

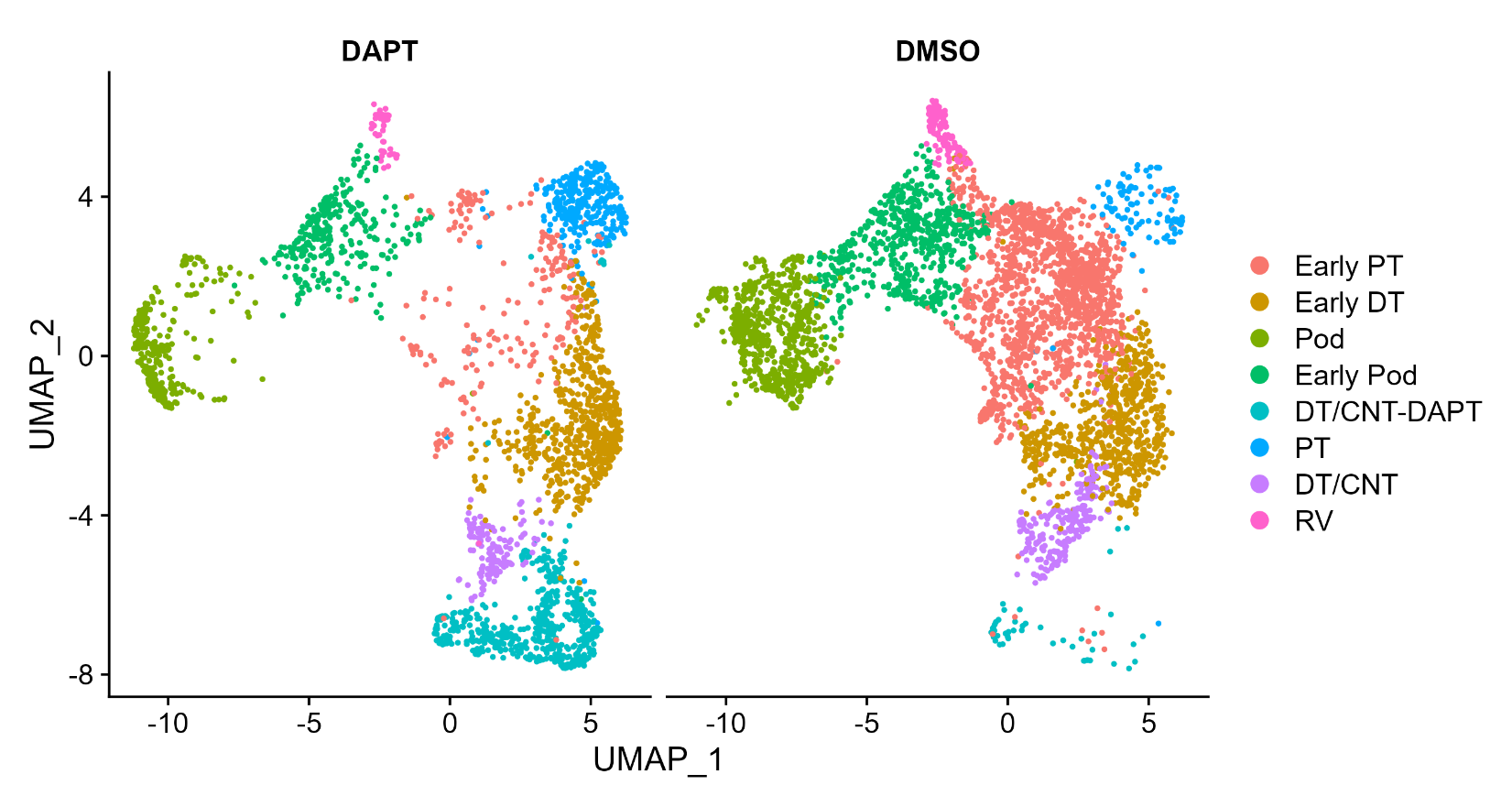

Supplement: Supplementary file 7 — Source data Fig. 5 [file 44318_2025_504_MOESM7_ESM.zip › Figure 5/5G/5G.tiff]

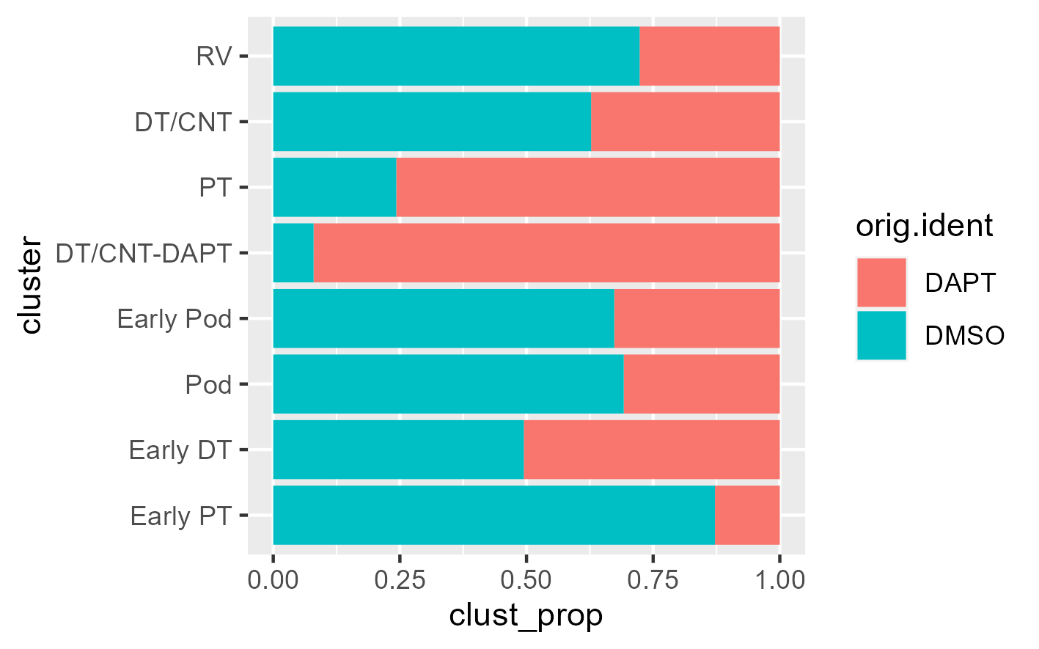

Supplement: Supplementary file 7 — Source data Fig. 5 [file 44318_2025_504_MOESM7_ESM.zip › Figure 5/5H/5H.tiff]

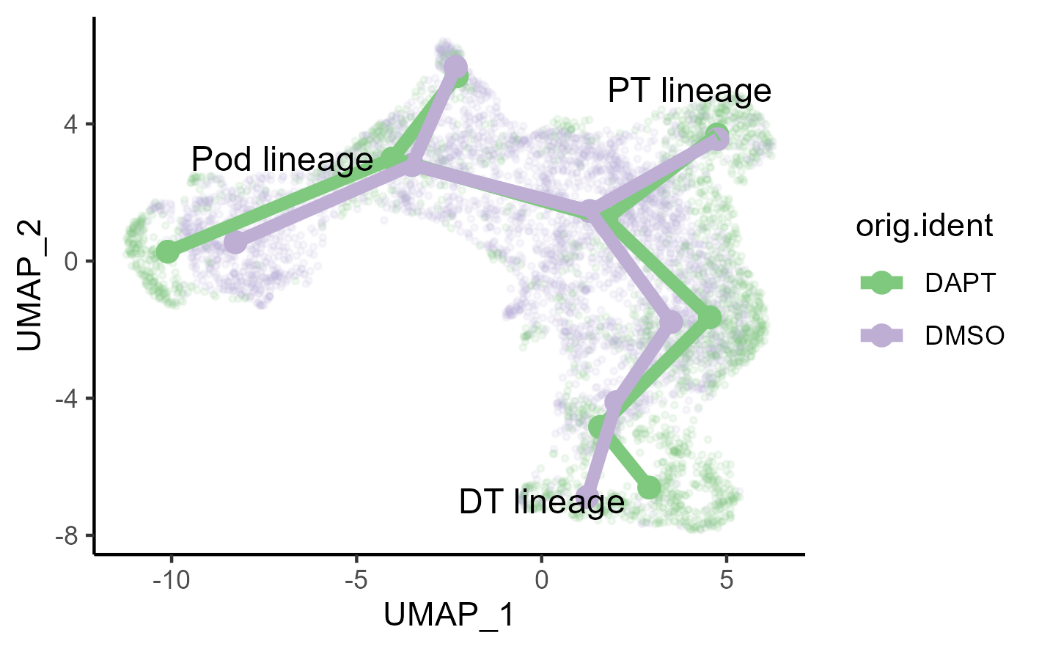

Supplement: Supplementary file 7 — Source data Fig. 5 [file 44318_2025_504_MOESM7_ESM.zip › Figure 5/5I/5I.tiff]

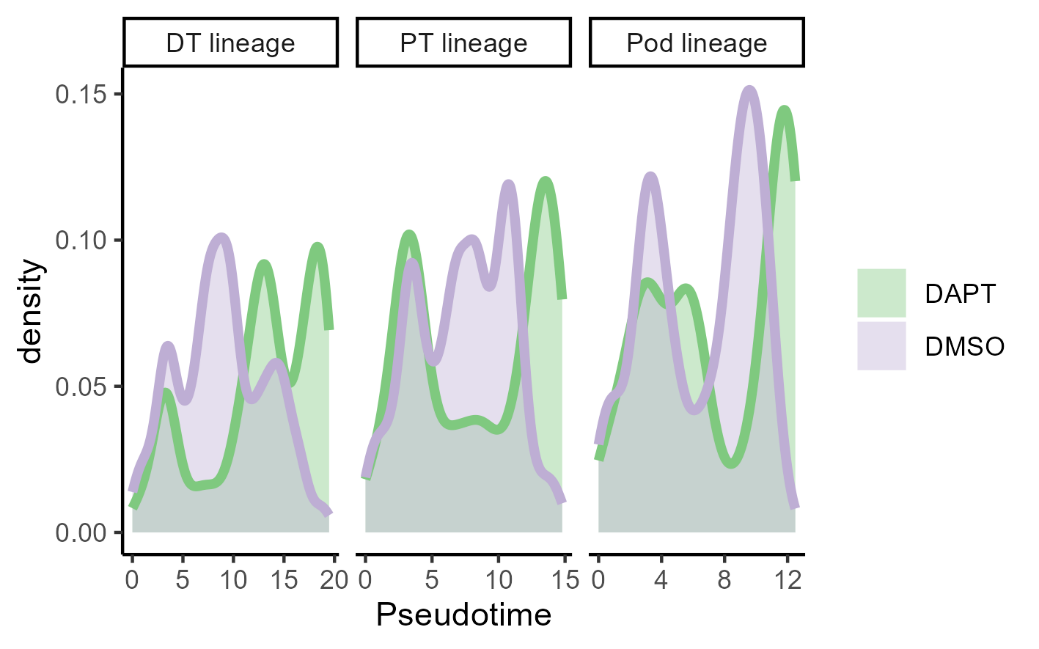

Supplement: Supplementary file 7 — Source data Fig. 5 [file 44318_2025_504_MOESM7_ESM.zip › Figure 5/5J/5J-a.tiff]

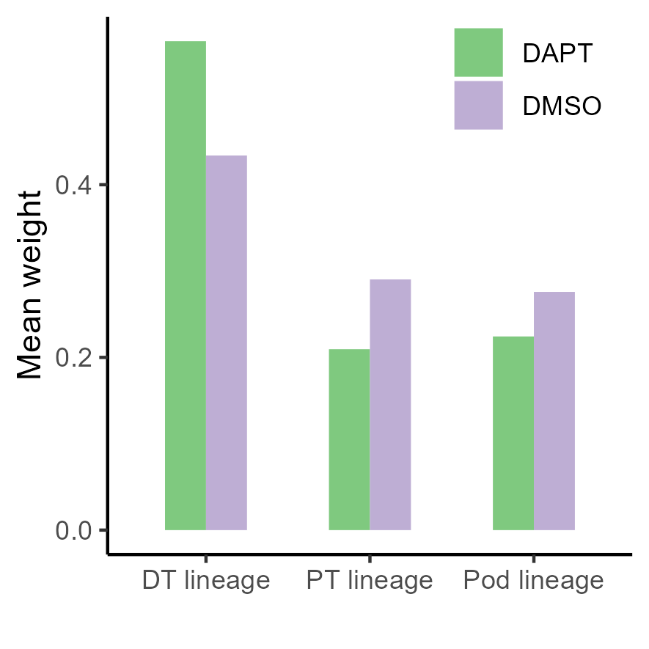

Supplement: Supplementary file 7 — Source data Fig. 5 [file 44318_2025_504_MOESM7_ESM.zip › Figure 5/5J/5J-b.tiff]
