# Supplementary material for: Human fetal kidney organoids model early human nephrogenesis and Notch-driven cell fate
Source: EMBO J. 2025 Jul 21;44(17):4681–719. doi: 10.1038/s44318-025-00504-2 (PMC12402132; doi:10.1038/s44318-025-00504-2)
Supplement: Supplementary file 8 — Expanded View Figures [file 44318_2025_504_MOESM8_ESM.pdf]

# Expanded View Figures

**Figure EV1. human fetal kidney organoid (hFKO) phenotype under various culture medium.**

(**A**) Left: schematic representation of the different phenotypes derived from each medium. hNPSR medium forms hFKOs that are complex and convoluted, while AKOM medium drives a cystic phenotype. Middle panel: hFKOs under hNPSR form complex, convoluted structures (dashed white lines). Right: hFKOs under AKOM show a simple cystic phenotype, similar to that of adult kidney-derived tubuloids. Scale bars, 100 µm. (**B**) Representative widefield image of a BME droplet containing hFKOs with convoluted phenotype under hNPSR. Scale bars, 500 µm. (**C**) Representative widefield image of a BME droplet containing hFKOs showcasing a cystic phenotype under AKOM. Scale bars, 500 µm. (**D**) Electron microscopy of hFKOs. Left: Microvilli (black arrows) are present in the apical lumen of hFKOs, along with basement membrane (BM). Middle: Tight junctions (white arrows) and lumens with microvilli (dashed white arrow) between cells in hFKOs. Left: Primary cilia (black arrow), often present in proximal and distal tubules as well as collecting ducts in the mature kidney. (**E**) hFKOs contain KI-67$^{+}$ cells which allow their long-term proliferation. qRT-PCR of P0 and P6 hFKOs reveal increased *CDH1* expression and maintenance of *EPCAM* expression, suggesting a stable renal epithelial phenotype. $n = 3$; expression levels in P0 were used to normalize data. Data were calculated as average ± SD. *$P < 0.05$., immunostaining of P2 and P6 hFKOs confirm the maintenance of an epithelial identity. Scale bars, 20 µm (top right), 50 µm (bottom panels). (**F**) Not all hFKOs swell under forskolin treatment since the culture is enriched with early tubular epithelial cells, which putatively do not possess the correct channels for water absorption. Scale bars, 500 µm.

 © The Author(s)

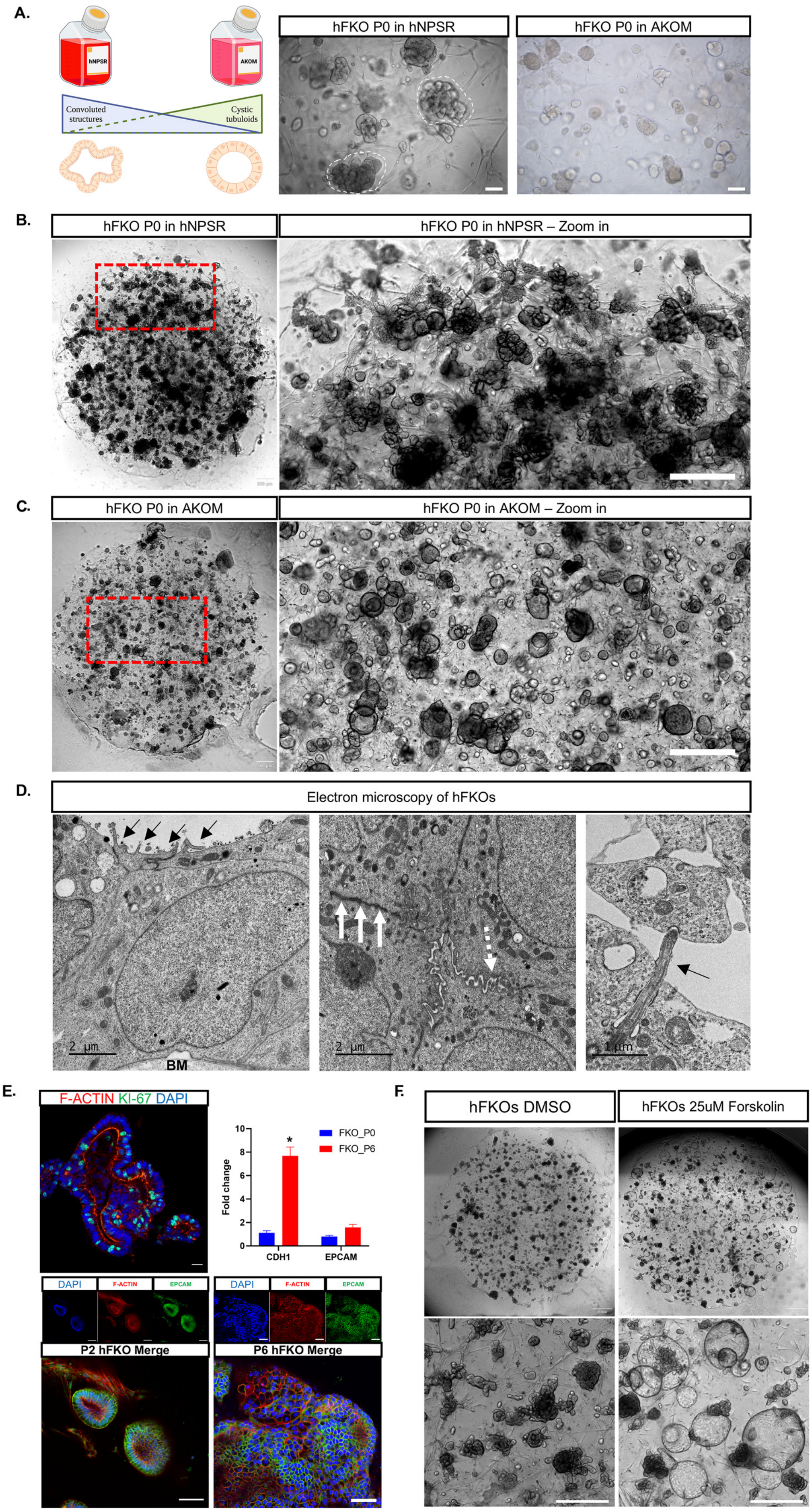
A.
hNPSR
AKOM
Convoluted structures
Cystic tubuloids
hFKO P0 in hNPSR
hFKO P0 in AKOM
B.
hFKO P0 in hNPSR
hFKO P0 in hNPSR – Zoom in
C.
hFKO P0 in AKOM
hFKO P0 in AKOM – Zoom in
D.
Electron microscopy of hFKOs
2 µm
BM
2 µm
1 µm
E.
F-ACTIN KI-67 DAPI
Fold change
FKO_P0
FKO_P6
CDH1
EPCAM
DAPI
F-ACTIN
EPCAM
P2 hFKO Merge
P6 hFKO Merge
F.
hFKOs DMSO
hFKOs 25uM Forskolin

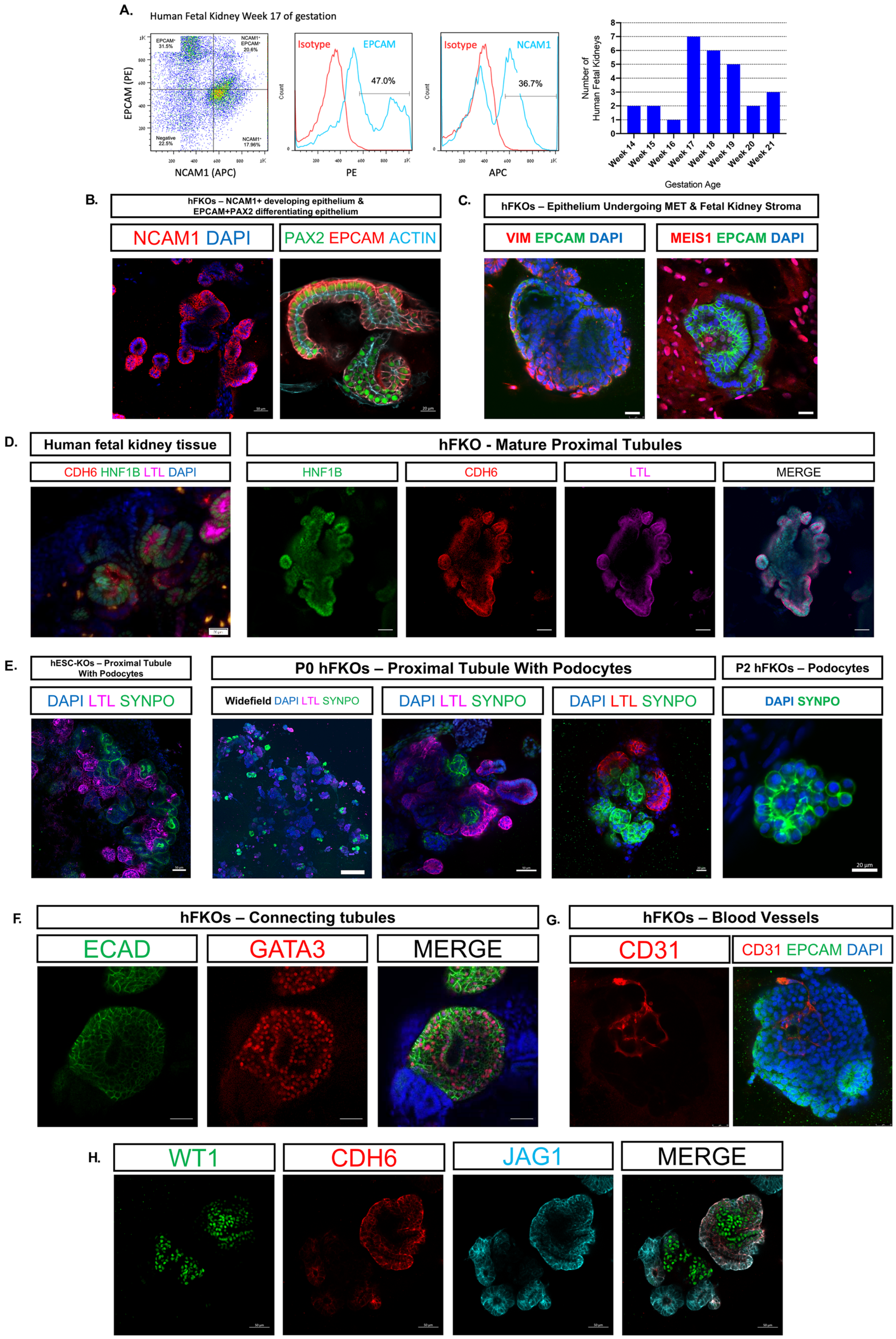
A.
Human Fetal Kidney Week 17 of gestation
EPCAM (PE)
NCAM1 (APC)
EPCAM+ 31.5%
NCAM1+ EPCAM+ 20.6%
Negative 22.5%
NCAM1+ 17.96%
Isotype
EPCAM
47.0%
Count
PE
Isotype
NCAM1
36.7%
Count
APC
Number of Human Fetal Kidneys
Week 14
Week 15
Week 16
Week 17
Week 18
Week 19
Week 20
Week 21
Gestation Age
B.
hFKOs – NCAM1+ developing epithelium & EPCAM+PAX2 differentiating epithelium
NCAM1 DAPI
PAX2 EPCAM ACTIN
C.
hFKOs – Epithelium Undergoing MET & Fetal Kidney Stroma
VIM EPCAM DAPI
MEIS1 EPCAM DAPI
D.
Human fetal kidney tissue
CDH6 HNF1B LTL DAPI
hFKO - Mature Proximal Tubules
HNF1B
CDH6
LTL
MERGE
E.
hESC-KOs – Proximal Tubule With Podocytes
DAPI LTL SYNPO
P0 hFKOs – Proximal Tubule With Podocytes
Widefield DAPI LTL SYNPO
DAPI LTL SYNPO
DAPI LTL SYNPO
P2 hFKOs – Podocytes
DAPI SYNPO
20 µm
F.
hFKOs – Connecting tubules
ECAD
GATA3
MERGE
G.
hFKOs – Blood Vessels
CD31
CD31 EPCAM DAPI
H.
WT1
CDH6
JAG1
MERGE

 © The Author(s)

**Figure EV2. Developmental hierarchy is preserved in hFKOs.**

(**A**) Flow cytometry analysis of composition of freshly dissociated hFKs containing NCAM1$^+$EPCAM$^-$ early epithelial progenitors as well as NCAM1$^+$EPCAM$^+$ committed early differentiated epithelium. Distribution of gestational age of hFKs received for research; weeks 17–19 were the most common. (**B**) Morphological difference between NCAM1$^+$PAX2$^+$ early developing nephrons and EPCAM$^+$ mature differentiated epithelial. NCAM1$^+$ structures are aggregates, while EPCAM$^+$ structures are more convoluted and tubular in nature. (**C**) hFKOs with EPCAM$^+$VIM$^+$ cells undergoing mesenchymal-to-epithelial transition (MET) and also MEIS1$^+$ fetal kidney stromal cells surrounding the epithelial structures. (**D**) Mature proximal tubule structures in hFKOs expressing HNF1B, CDH6, and LTL. (**E**) Left, human embryonic stem cell-derived kidney organoids stained for LTL and synaptopodin (SYNPO). Middle, P0 hFKOs with podocytes expressing SYNPO accompanied by LTL$^+$ proximal tubules. Left, P2 hFKOs expressing SYNPO. (**F**) ECAD$^+$GATA3$^+$ connecting tubules in hFKOs. (**G**) CD31$^+$ endothelial cells form small blood vessels in EPCAM$^+$ hFKOs. (**h**) hFKOs consist of multiple lineages, including WT1$^+$CDH6$^+$ proximal tubules and JAG1$^+$ medial and distal lineages.

© The Author(s)

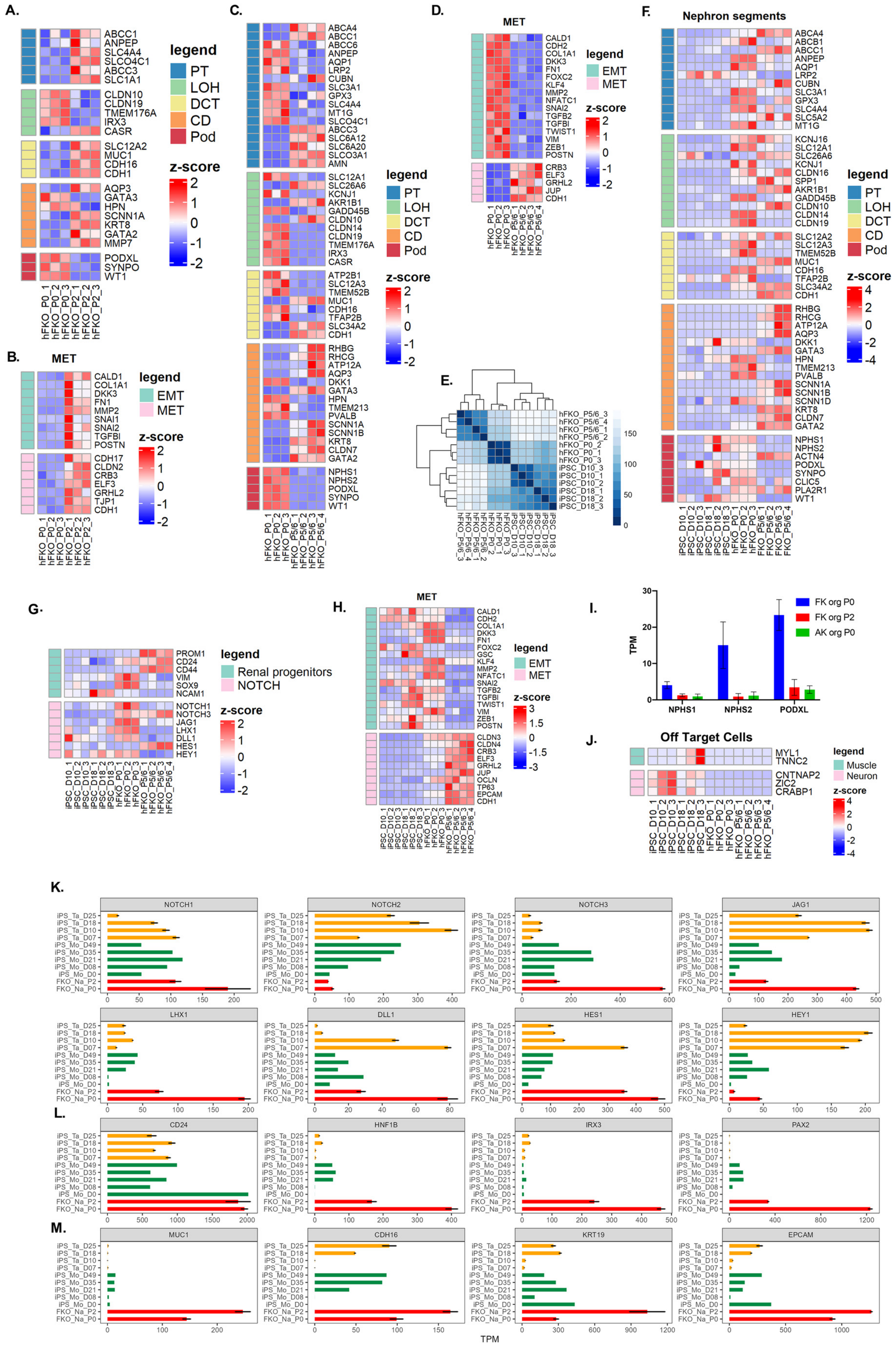
A.
ABCC1
ANPEP
SLC4A4
SLCO4C1
ABCC3
SLC1A1
CLDN10
CLDN19
TMEM176A
IRX3
CASR
SLC12A2
MUC1
CDH16
CDH1
AQP3
GATA3
HPN
SCNN1A
KRT8
GATA2
MMP7
PODXL
SYNPO
WT1
legend
PT
LOH
DCT
CD
Pod
z-score
B.
MET
legend
EMT
MET
C.
D.
MET
E.
F.
Nephron segments
G.
Renal progenitors
NOTCH
H.
I.
TPM
FK org P0
FK org P2
AK org P0
NPHS1
NPHS2
PODXL
J.
Off Target Cells
MYL1
TNNC2
CNTNAP2
ZIC2
CRABP1
Muscle
Neuron
K.
NOTCH1
NOTCH2
NOTCH3
JAG1
LHX1
DLL1
HES1
HEY1
L.
CD24
HNF1B
IRX3
PAX2
M.
MUC1
CDH16
KRT19
EPCAM
TPM

 © The Author(s)

**Figure EV3. Bulk RNA sequencing of hFKOs shows superior expression of kidney development genes.**

(**A**) Nephron segment markers increase in P2 of hFKOs, indicating maturation process in vitro. (**B**) EMT and MET markers indicate a dual process mesenchymal proliferation and epithelial transition, suiting the self-renewal of NPCs and differentiation of early nephrons. (**C**) Heatmap comparing the expression of segment-specific markers between early (P0) and late (P5/P6) hFKOs. (**D**). Comparison of expression of EMT and MET markers in P0 and P6 hFKOs. (**E**) Distance plot of human fetal kidney organoids (hFKO), and iPSC-derived kidney organoids (iPSC). (**F**) Nephron segment markers of P0 and P6 hFKOs vs D10 and D18 iPSC-derived kidney organoids (iPSC-KOs). (**G**) Renal progenitor and Notch signaling pathways related genes expression in P0 and P6 hFKOs vs D10 and D18 iPSC-KOs. (**H**) Comparison of expression of EMT and MET markers in P0 and P6 hFKOs vs D10 and D18 iPSC-KOs. (**I**) Podocyte marker expression in P0 hFKOs versus P0 Adult kidney organoids (AK org). hFKOs contain all nephron segments in comparison to AKOs which contain only the tubular segments. (**J**) Comparison of off-target marker expression between iPSC-KOs and hFKOs, expression of muscles markers, MYL1 and TNNC2 and neuron markers CNTNAP2, CRABP1 and ZIC2 are higher in iPSC-KOs. (**K**) Key Notch markers expression (in transcripts per million, TPM) in P0 and P2 hFKOs versus other prominent hPSC-KO differentiation protocols, Morizane et al (Mo) and Takasato et al (Ta), at various timepoints (Mo: day 8, 21,35 and 49. Ta: day 7, 10, 18 and 25). Notch1, Notch3, JAG1, LHX1, DLL1 and HES1 are expressed on par with hPSC-KOs or expressed higher in hFKOs. (**L**) Expression of key epithelial progenitor markers such as CD24, HFN1B, IRX3 and PAX2 in hFKOs in comparison to other hPSC-KO protocols, HNF1B, IRX3 and PAX2 are expressed higher in hFKOs, even in P2, indicating that the hFKO culture retains a population of epithelial progenitors even after 8 weeks of culture. In comparison to hFKOs, PAX2 and HNF1B are quickly downregulated in hPSC-KOs. (**M**) Expression of nephron epithelium markers, MUC1, CDH16, KRT19 and EPCAM, is increased in P2 hFKOs, indicating differentiation and maturation processes similar to the native kidney.

© The Author(s)

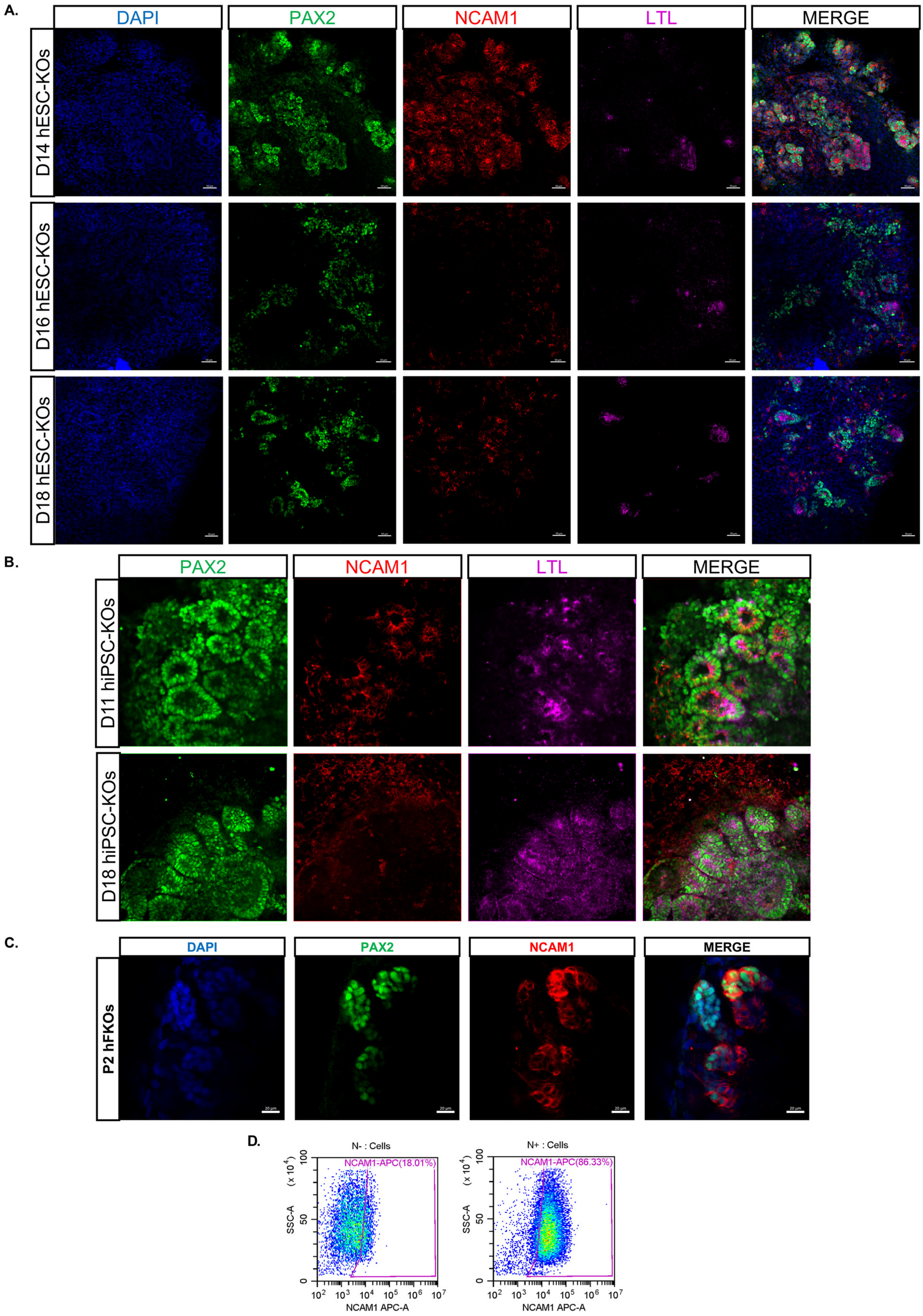
A.
DAPI
PAX2
NCAM1
LTL
MERGE
D14 hESC-KOs
D16 hESC-KOs
D18 hESC-KOs
B.
PAX2
NCAM1
LTL
MERGE
D11 hiPSC-KOs
D18 hiPSC-KOs
C.
DAPI
PAX2
NCAM1
MERGE
P2 hFKOs
20 μm
D.
N- : Cells
NCAM1-APC(18.01%)
N+ : Cells
NCAM1-APC(86.33%)
SSC-A (x 10^4)
0
50
100
10^2 10^3 10^4 10^5 10^6 10^7
NCAM1 APC-A

 © The Author(s)

**Figure EV4. Nephron progenitor markers NCAM1 and PAX2 are less abundant as hPSC-derived kidney organoids mature.**

(**A**) Immunofluorescent imaging of human embryonic stem cell-derived kidney organoids (hESC-KOs), expressing many NCAM1+ and PAX2+ structures, containing nephron progenitor cells at Day 14 of differentiation. These structures become less abundant as the culture progresses, even after an additional two days (D16). As the differentiation progresses, PAX2+ cells become organized into tubular structures and decrease in number. scale bar 50 μm. (**B**) IF imaging of hiPSC-derived kidney organoids. NPC populations co-expressing NCAM1 + PAX2+ diminish as the organoid differentiates and NCAM1 cells become unorganized and are more abundant in the stroma of the organoid. (**C**) in P2 hFKOs, after 8 weeks of culture, NCAM1 + PAX2+ co-expression is evident in epithelial progenitors. Scale bar 20 μm. (**D**) Flow cytometry of positive and negative fractions of NCAM1+ after magnetic sorting.

© The Author(s) 

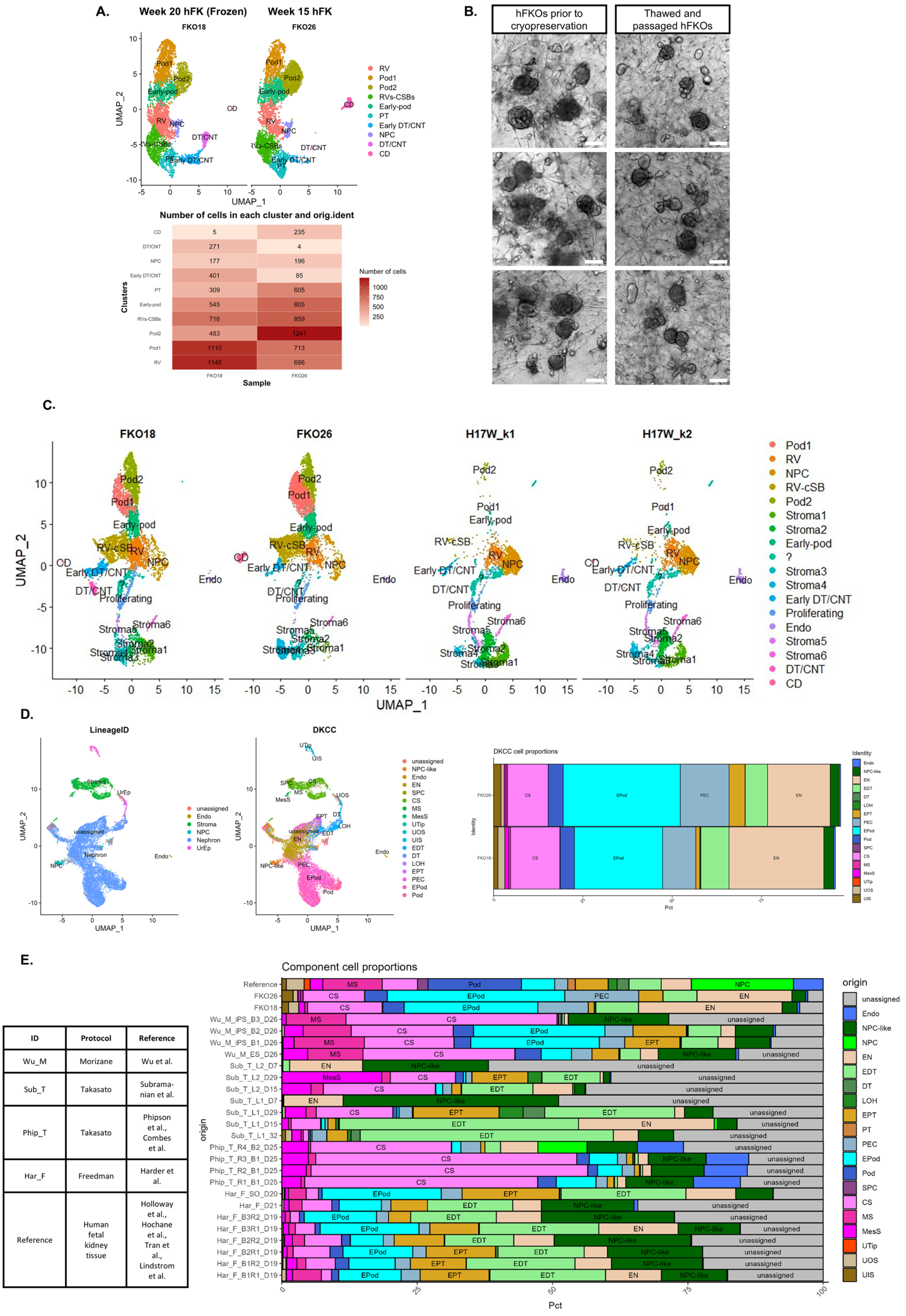


| ID | Protocol | Reference |
|---|---|---|
| Wu_M | Morizane | Wu et al. |
| Sub_T | Takasato | Subramanian et al. |
| Phip_T | Takasato | Phipson et al., Combes et al. |
| Har_F | Freedman | Harder et al. |
| Reference | Human fetal kidney tissue | Holloway et al., Hochane et al., Tran et al., Lindstrom et al. |

 © The Author(s)

**Figure EV5. Single-cell RNA sequencing of hFKOs reveals developmental processes similar to those of native fetal kidney.**

(**A**) UMAP of each hFKO sample used in the scRNA-seq dataset after nephrogenic sub-setting. The two samples exhibit similarity in clusters, even though one hFKO sample originated from thawed hFK cells. Right: Table depicting number of cells in each cluster. (**B**) hFKO cells cryopreserved compared to hFKO cells after thawing and continued culture, no significant change of morphology was detected. (**C**) Comparison of hFKOs scRNA-seq dataset to human fetal kidney cortex dataset, matching NPCs and RVs. (**D**) Unbiased classification of hFKOs clusters with the DevKidCC tool. Clusters are classified similarly to initial classification made using markers depicted in the literature. (**E**) Comparison of nephrogenic clusters and their proportions between hFKOs, human fetal kidney tissue, and kidney organoids derived from PSCs using prominent organoid differentiation protocols. ID, protocols, and references used to produce the plot are depicted in the table at left. Origin annotations: (UTip), outer stalk (UOS), inner stalk (UIS), stromal progenitor cells (SPC), cortical stroma (CS), medullary stroma (MS), mesangial cells (MesS), endothelium (Endo), nephron progenitor cells (NPC), early nephron (EN), early distal tubule (EDT), distal tubule (DT), Loop of Henle (LOH), early proximal tubule (EPT), proximal tubule (PT), parietal epithelial cells (PEC), early podocytes (EPod) and podocytes (Pod).

© The Author(s)

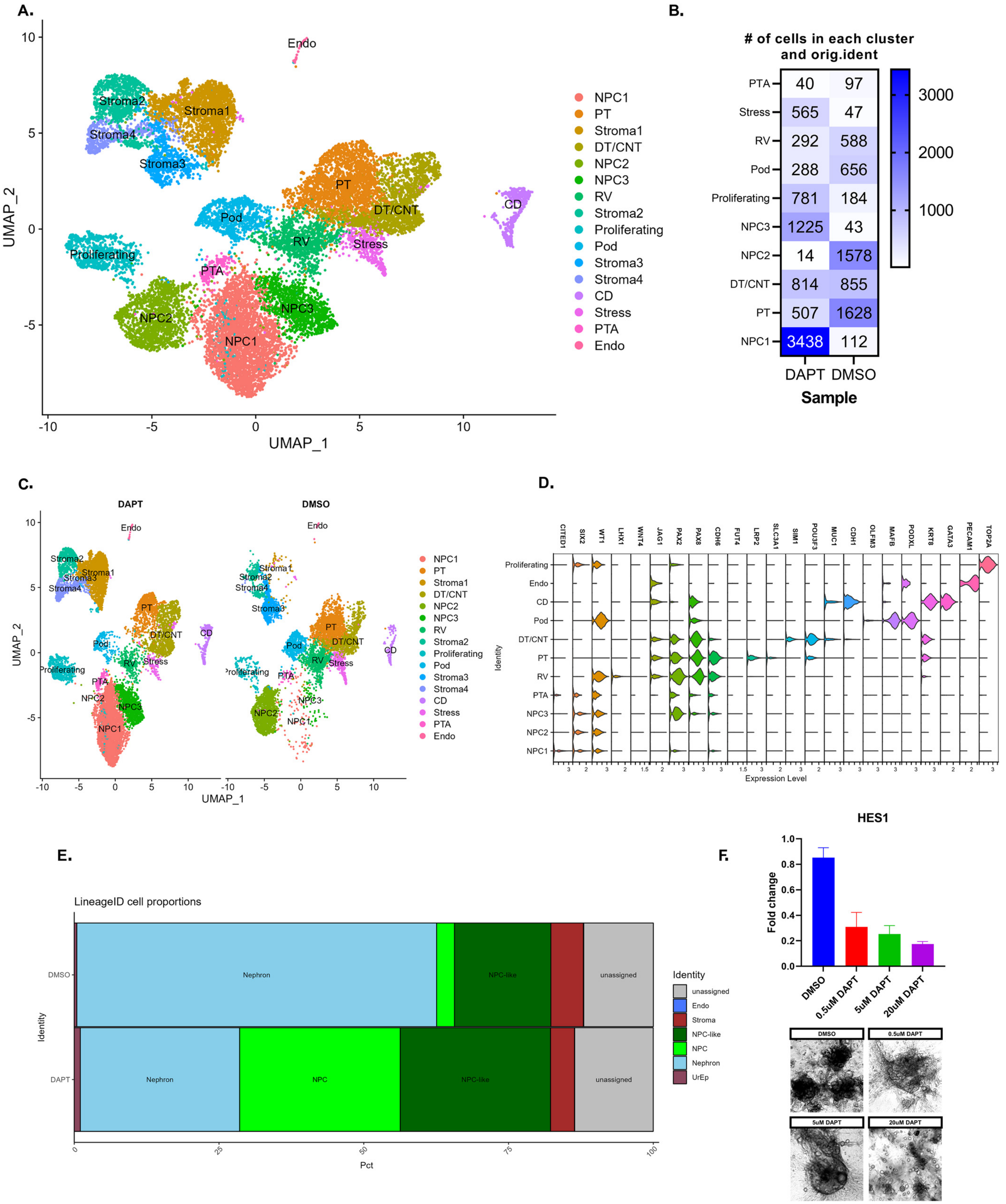
A.
UMAP_1
UMAP_2
Endo
Stroma2
Stroma1
Stroma4
Stroma3
PT
DT/CNT
Pod
RV
Stress
Proliferating
PTA
NPC3
NPC2
NPC1
CD
NPC1
PT
Stroma1
DT/CNT
NPC2
NPC3
RV
Stroma2
Proliferating
Pod
Stroma3
Stroma4
CD
Stress
PTA
Endo
B.
# of cells in each cluster and orig.ident
PTA 40 97
Stress 565 47
RV 292 588
Pod 288 656
Proliferating 781 184
NPC3 1225 43
NPC2 14 1578
DT/CNT 814 855
PT 507 1628
NPC1 3438 112
DAPT DMSO
Sample
3000
2000
1000
C.
DAPT
DMSO
UMAP_1
UMAP_2
D.
CITED1 SIX2 WT1 LHX1 WNT4 JAG1 PAX2 PAX8 CDH6 FUT4 LRP2 SLC3A1 SIM1 POU3F3 MUC1 CDH1 OLFM3 MAFB PODXL KRT8 GATA3 PECAM1 TOP2A
Proliferating
Endo
CD
Pod
DT/CNT
PT
RV
PTA
NPC3
NPC2
NPC1
Identity
Expression Level
E.
LineageID cell proportions
DMSO
DAPT
Nephron
NPC
NPC-like
unassigned
Identity
Pct
Identity
unassigned
Endo
Stroma
NPC-like
NPC
Nephron
UrEp
F.
HES1
Fold change
DMSO
0.5uM DAPT
5uM DAPT
20uM DAPT
DMSO
0.5uM DAPT
5uM DAPT
20uM DAPT


© The Author(s)

**Figure EV6. Proximal tubule lineage is hampered under Notch inhibition in hFKOs.**

(**A**) UMAP of DAPT and control hFKOs clusters. NPC, nephron progenitors; RV, renal vesicles; Pod, podocytes; DT/CNT, distal/connecting tubule; PT, proximal tubule; PTA, pre-tubular aggregates; endo, endothelial cells, CD, collecting ducts. (**B**) Each treatment leads to different proportions of cells in each cluster, an increased NPC population under DAPT inhibition. and decreased PT and RV cells. (**C**) UMAP split by sample identity, DAPT and control. hFKOs under DAPT treatment have more NPCs, which are retained in a progenitor state under Notch inhibitions. (**D**) Violin plots of the expression of markers used in classifying clusters. (**E**) Unbiased classification of clusters in hFKOs under DAPT inhibition and control (DMSO) with the DevKidCC tool. (**F**) DAPT inhibits HES1 expression in a dose-dependent manner, as the concentration of DAPT increases, the fold change in HES1 expression decreases. Morphological changes, such as reduction in organoid complexity, begin to be evident at concentrations of 5 µM DAPT while no significant change in expression is observed in higher concentrations.

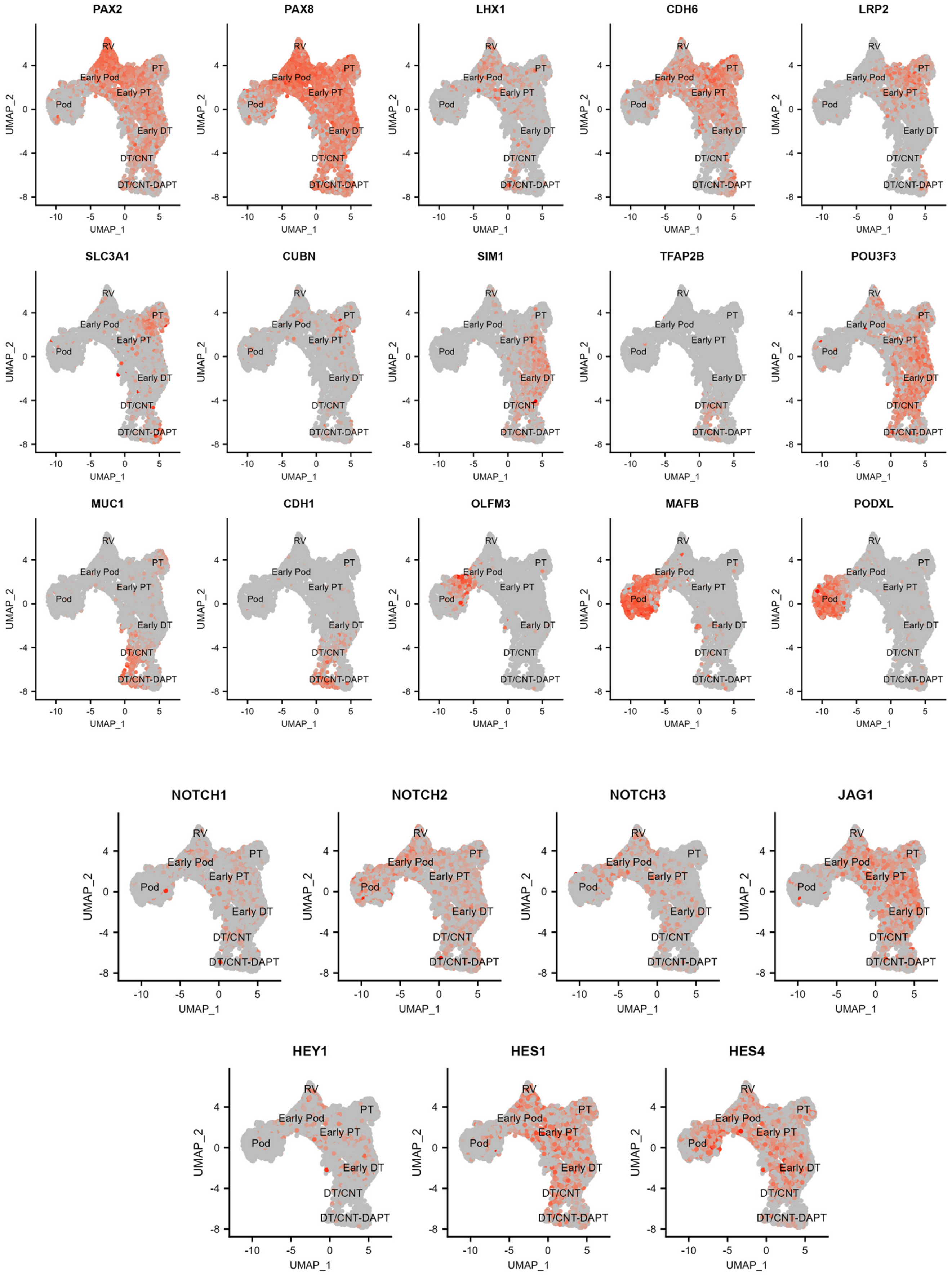
PAX2
PAX8
LHX1
CDH6
LRP2
SLC3A1
CUBN
SIM1
TFAP2B
POU3F3
MUC1
CDH1
OLFM3
MAFB
PODXL
NOTCH1
NOTCH2
NOTCH3
JAG1
HEY1
HES1
HES4
RV
Early Pod
PT
Early PT
Pod
Early DT
DT/CNT
DT/CNT-DAPT
UMAP_1
UMAP_2

 © The Author(s)

**Figure EV7. Proximal tubule lineage is hampered under Notch inhibition in hFKOs.**

Feature plots of key lineage markers used to infer differentiation states: for example, *TFAP2B* and *POU3F3* are marker of early distal lineage and *CDH1* and *MUC1* are mature distal tubule markers, *CDH6* is a distal early proximal tubule marker, and *LRP2* and *SLC3A1* are mature proximal tubule markers.

© The Author(s)

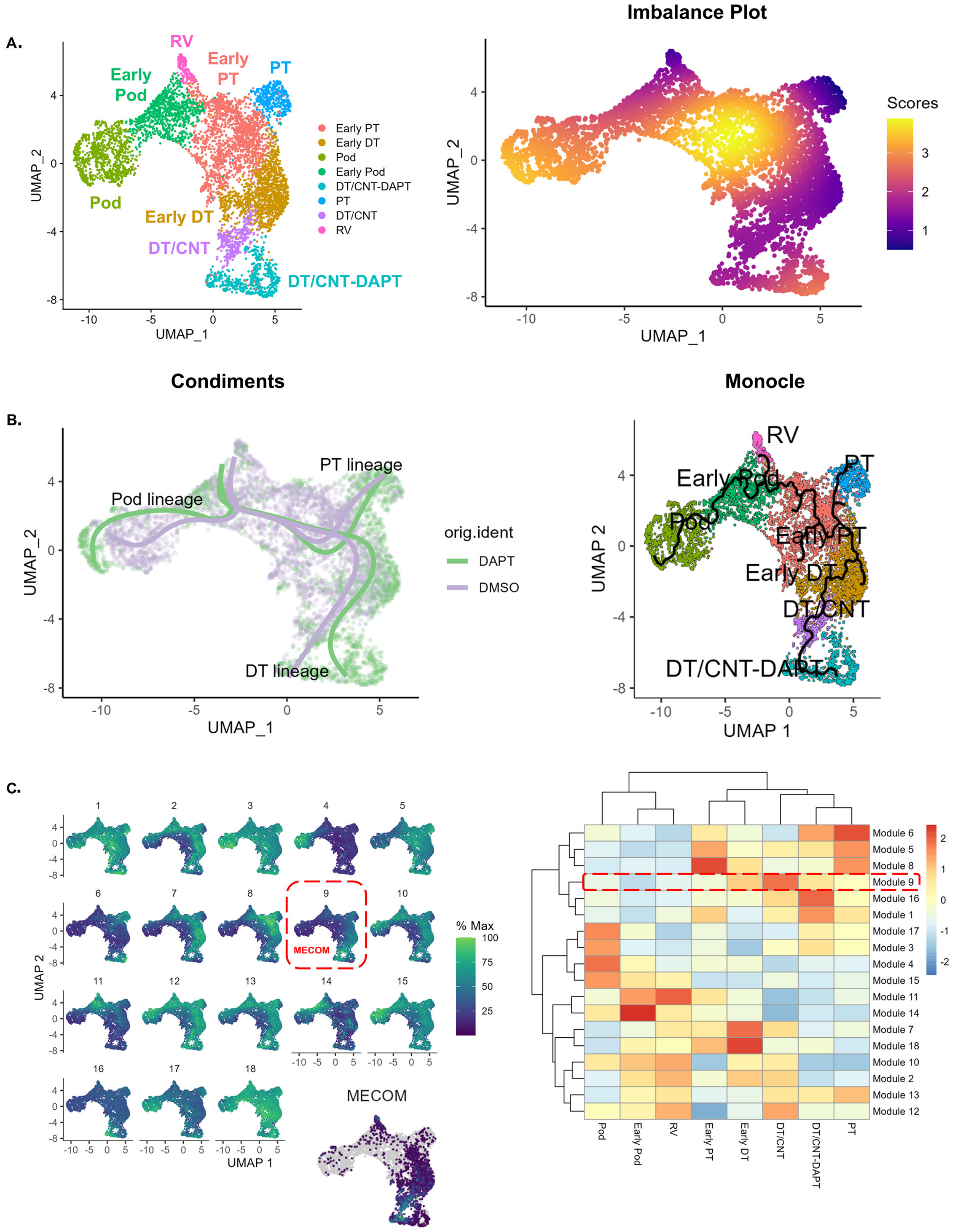
A.
RV
Early Pod
Early PT
PT
Pod
Early DT
DT/CNT
DT/CNT-DAPT
Early PT
Early DT
Pod
Early Pod
DT/CNT-DAPT
PT
DT/CNT
RV
UMAP_1
UMAP_2
Imbalance Plot
Scores
B.
Condiments
PT lineage
Pod lineage
DT lineage
orig.ident
DAPT
DMSO
Monocle
UMAP 1
UMAP 2
C.
MECOM
% Max
Module 6
Module 5
Module 8
Module 9
Module 16
Module 1
Module 17
Module 3
Module 4
Module 15
Module 11
Module 14
Module 7
Module 18
Module 10
Module 2
Module 13
Module 12
Pod
Early Pod
RV
Early PT
Early DT
DT/CNT
DT/CNT-DAPT
PT

 © The Author(s)

**Figure EV8. Proximal tubule lineage is hampered under Notch inhibition in hFKOs.**

(**A**) scRNA-seq of hFKOs under Notch inhibition (DAPT) and control (DMSO), and UMAP of the nephrogenic compartment. RV, renal vesicle; PT, proximal tubule; Pod, podocyte; DT, distal tubule; DT/CNT, distal tubule/connecting tubule; DT/CNT-DAPT, distal tubule/connecting tubule enriched under Notch inhibition. Imbalance plot depicting the area in the UMAP with the highest amount of mismatch between control and DAPT, area containing mostly the early PT cluster. (**B**) Comparison between pseudotime plot from the condiments package and monocle, leading to similar patterns of trajectory inference. (**C**) Breakdown of trajectory into modules, making it possible to scan for DE genes in certain patterns. Module 9 depicts a pattern of bypass whereby early distal cells circumvent Notch inhibition, putatively through the influence of MECOM.

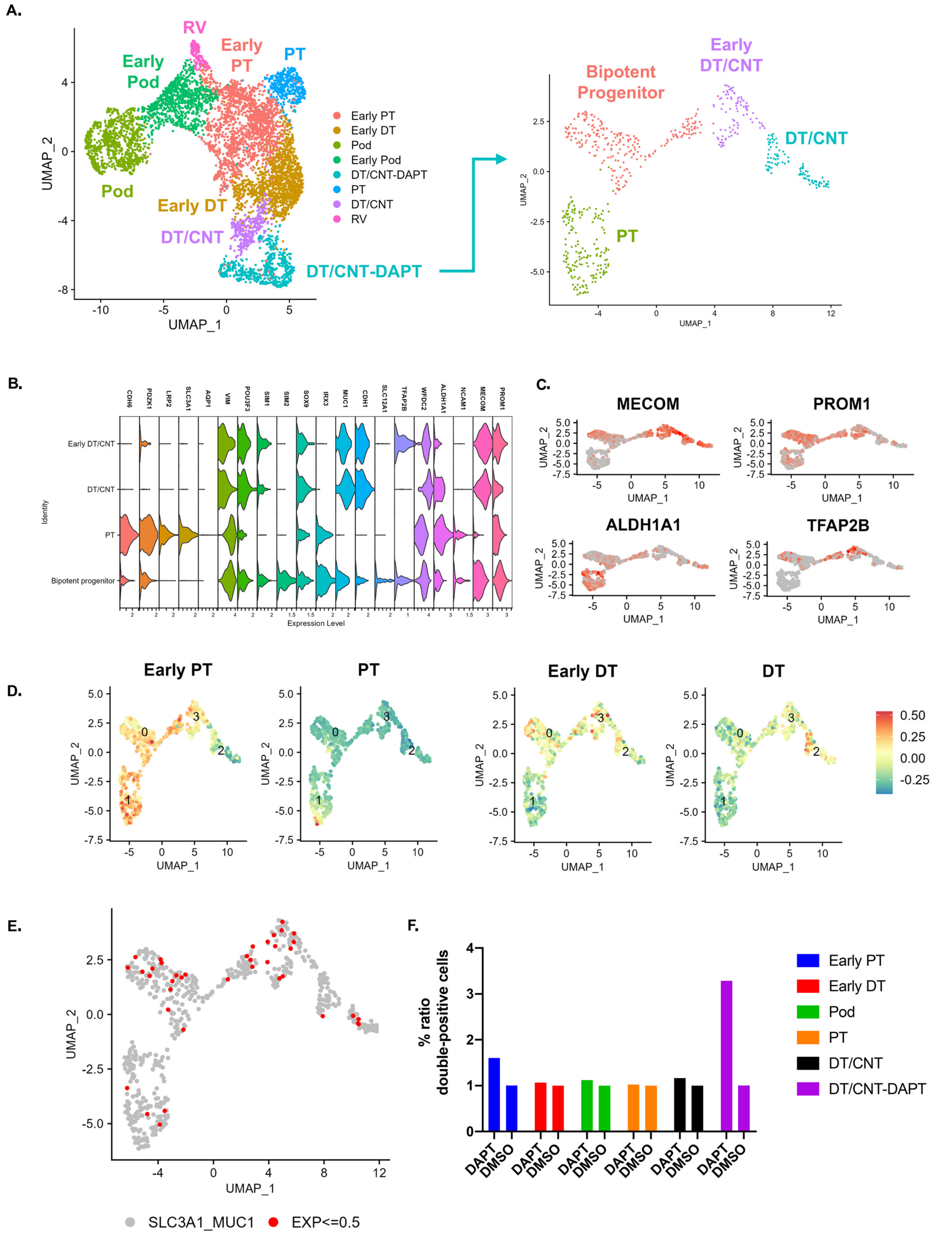
A.
RV
Early Pod
Early PT
PT
Pod
Early DT
DT/CNT
DT/CNT-DAPT
UMAP_1
UMAP_2
Early PT
Early DT
Pod
Early Pod
DT/CNT-DAPT
PT
DT/CNT
RV
Bipotent Progenitor
Early DT/CNT
DT/CNT
PT
B.
CDH6
PDZK1
LRP2
SLC3A1
AQP1
VIM
POU3F3
SIM1
SIM2
SOX9
IRX3
MUC1
CDH1
SLC12A1
TFAP2B
WFDC2
ALDH1A1
NCAM1
MECOM
PROM1
Early DT/CNT
DT/CNT
PT
Bipotent progenitor
Identity
Expression Level
C.
MECOM
PROM1
ALDH1A1
TFAP2B
D.
Early PT
PT
Early DT
DT
0.50
0.25
0.00
-0.25
E.
SLC3A1_MUC1
EXP<=0.5
F.
% ratio double-positive cells
DAPT
DMSO
Early PT
Early DT
Pod
PT
DT/CNT
DT/CNT-DAPT

 © The Author(s)

**Figure EV9. Proximal tubule lineage is hampered under Notch inhibition in hFKOs.**

(**A**) Sub-setting and re-clustering of the distal tubule/connecting tubule enriched under Notch inhibition (DT/CNT-DAPT) reveals four clusters: bipotent progenitors, early DT/CNT, DT/CNT, and PT. (**B**) Violin plots of the expression of key markers used to characterize the four sub-clusters in the DT/CNT-DAPT cluster. (**C**) Feature plot depicting expression patterns of the distal markers *MECOM* and *TFAP2B*, localizing in the distal clusters; *ALDH1A1*, localizing in the PT cluster; and *PROM1* (*CD133*), expressed in all clusters. (**D**) Feature plot expression heatmap of genes characterizing each lineage, Early PT (*CDH6*, *HNF1A*, *IGFBP7*, etc.), PT (*SLC3A1*, *SLC4A4*, *SLC3A1*, etc.), early DT (*POU3F3*, *SOX9*, *IRX2*, etc.), and DT (*SLC12A3*, *MUC1*, *CALB1*, etc.) (**E**) Double-positive cells detected in the DT/CNT-DAPT cluster, expressing proximal marker *SLC3A1* and distal marker *MUC1;* threshold is set to above 0.5. (**F**) Percent ratio bar plot of double-positive cells expressing *SLC3A1* and *MUC1* in the various clusters.
